# Targeted knock-in of *NCF1* cDNA into the *NCF2* locus leads to myeloid phenotypic correction of p47^*phox*^-deficient chronic granulomatous disease

**DOI:** 10.1016/j.omtn.2024.102229

**Published:** 2024-05-24

**Authors:** Kah Mun Siow, Merve Güngör, Dominik Wrona, Federica Raimondi, Oleksandr Pastukhov, Panagiotis Tsapogas, Timon Menzi, Michael Schmitz, Péter István Kulcsár, Gerald Schwank, Ansgar Schulz, Martin Jinek, Ute Modlich, Ulrich Siler, Janine Reichenbach

**Affiliations:** 1 Division of Gene and Cell Therapy, Institute for Regenerative Medicine, University of Zurich, Schlieren, 8952 Zurich, Switzerland; 2 Department of Biochemistry, University of Zurich, 8057 Zurich, Switzerland; 3 Institute of Pharmacology and Toxicology, University of Zurich, 8057 Zurich, Switzerland; 4 Department of Pediatrics, University Medical Center Ulm, 89075 Ulm, Germany; 5 School of Life Sciences, Institute for Pharma Technology, University of Applied Sciences and Arts Northwestern Switzerland, 4132 Muttenz, Switzerland; 6 Department of Somatic Gene Therapy, University Children’s Hospital Zurich, 8032 Zurich, Switzerland; 7 Center for Applied Biotechnology and Molecular Medicine (CABMM), University of Zurich, 8057 Zurich, Switzerland

**Keywords:** MT: RNA/DNA Editing, CRISPR-Cas9, HDR-mediated knock-in, hematopoietic stem cells, *NCF2*, *NCF1*, gene editing, gene therapy, chronic granulomatous disease, adeno-associated viral vectors, integration-deficient lentiviral vectors

## Abstract

p47^*phox*^-deficient chronic granulomatous disease (p47-CGD) is a primary immunodeficiency caused by mutations in the neutrophil cytosolic factor 1 (*NCF1*) gene, resulting in defective NADPH oxidase function in phagocytes. Due to its complex genomic context, the *NCF1* locus is not suited for safe gene editing with current genome editing technologies. Therefore, we developed a targeted *NCF1* coding sequence knock-in by CRISPR-Cas9 ribonucleoprotein and viral vector template delivery, to restore p47^*phox*^ expression under the control of the endogenous *NCF2* locus. *NCF2* encodes for p67^*phox*^, an NADPH oxidase subunit that closely interacts with p47^*phox*^ and is predominantly expressed in myeloid cells. This approach restored p47^*phox*^ expression and NADPH oxidase function in p47-CGD patient hematopoietic stem and progenitor cells (HSPCs) and in p47^*phox*^-deficient mouse HSPCs, with the transgene expression following a myeloid differentiation pattern. Adeno-associated viral vectors performed favorably over integration-deficient lentiviral vectors for template delivery, with fewer off-target integrations and higher correction efficacy in HSPCs. Such myeloid-directed gene editing is promising for clinical CGD gene therapy, as it leads to the co-expression of p47^*phox*^ and p67^*phox*^, ensuring spatiotemporal and near-physiological transgene expression in myeloid cells.

## Graphical abstract

**Figure:**
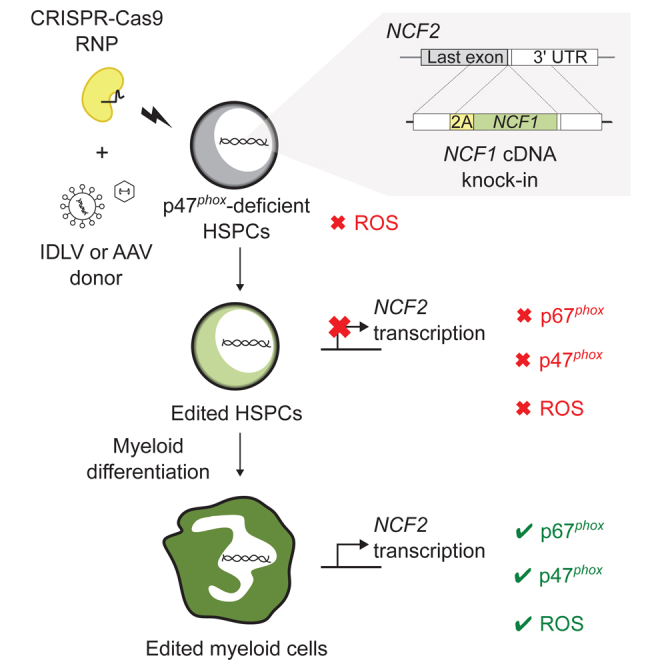

## Teaser

Reichenbach and colleagues present a CRISPR-based knock-in approach to express p47^*phox*^ under the control of an endogenous gene, *NCF2*, to treat p47^*phox*^-deficient CGD. Such an approach has the advantage of allowing a spatiotemporal and near-physiological transgene expression that follows a myeloid differentiation pattern in the edited cells.

## Introduction

Chronic granulomatous disease (CGD) is a group of primary immunodeficiency disorders characterized by defective respiratory bursts in phagocytes, which are caused by mutations in genes encoding subunits of the nicotinamide adenine dinucleotide phosphate (NADPH) oxidase, including p47^*phox*^ and p67^*phox*^.^1^^,^^2^ The resulting impaired NADPH oxidase function leads to the loss of reactive oxygen species (ROS) production, accounting for life-threatening bacterial and fungal infections, as well as hyperinflammation in affected patients.^3^^,^^4^ A potentially curative treatment for CGD is the transplantation of genetically modified autologous hematopoietic stem and progenitor cells (HSPCs). Several research groups have explored various *ex vivo* gene therapy approaches, including retroviral gene addition,^5^^,^^6^^,^^7^ direct mutation targeting with designer nucleases,^8^^,^^9^ safe harbor targeting,^10^^,^^11^^,^^12^^,^^13^ exon replacement,^14^ or minigene insertion,^15^ to correct different genetic subtypes of CGD.

In this study, we focused on p47^*phox*^-deficient CGD (p47-CGD), which in the majority of patients is caused by a two-nucleotide deletion (ΔGT) in the neutrophil cytosolic factor 1 (*NCF1*) gene.^16^ The mutated gene is colocalized on the same chromosome with two homologous pseudogenes, *NCF1B* and *NCF1C*, which display a high degree of sequence similarity to *NCF1*, and also carry the ΔGT sequence.^17^^,^^18^ Consequently, gene editing approaches that target the ΔGT sequence can simultaneously induce multiple double-strand breaks (DSBs) in the *NCF1* loci, ultimately leading to chromosomal aberrations, which could limit the potential clinical application of such strategies.^8^^,^^19^

To avoid such adverse outcomes, we developed an alternative gene editing approach using CRISPR-Cas9 and homology-directed repair (HDR)-mediated knock-in, inserting a promoter-less *NCF1* cDNA into the 3′ end of the endogenous *NCF2* gene. This gene has been selected as the target for *NCF1* knock-in, as the encoded p67^*phox*^ protein is predominantly expressed in myeloid cells,^20^^,^^21^^,^^22^ and exists as a tight complex with p47^*phox*^ in the resting state and in the activated NADPH oxidase.^23^^,^^24^ To ensure spatiotemporal regulation of the transgenic p47^*phox*^, we coupled both subunits into a single reading frame using the 2A co-expression system,^25^^,^^26^ limiting the p47^*phox*^ expression to the myeloid compartment of the hematopoietic system.

## Results

### Knock-in of *NCF1* cDNA into the *NCF2* locus restores p47^*phox*^ expression in p47^*phox*^-deficient cells

**Figure 1:**
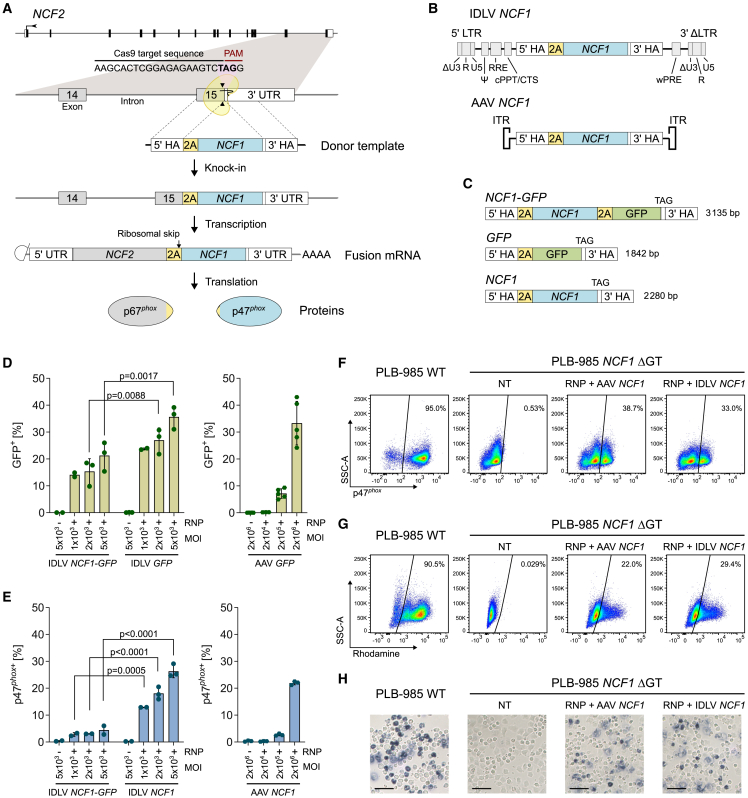
Targeted knock-in of *NCF1* cDNA into the *NCF2* locus restores p47^*phox*^ expression and ROS production in PLB-985 *NCF1* ΔGT cells (A) Schematic diagram of the knock-in strategy targeted at the translational stop codon of the *NCF2* gene. The stop codon (TAG in bold) was targeted by the gRNA for cleavage (arrowheads) by Cas9. Gray boxes indicate exons of *NCF2*. PAM, protospacer adjacent motif; TAG in bold, translational stop codon; HA, homology arm; 2A, FMDV 2A oligopeptide; *NCF1*, *NCF1* codon-optimized full-length cDNA; UTR, 3′ untranslated region; AAAA, poly(A) tail. (B) Types of vectors carrying the IDLV *NCF1* and AAV *NCF1* donor template. LTR, long terminal repeat; ΔU3, self-inactivating untranslated 3ʹ; R, repeat region; U5, untranslated 5ʹ; Ψ, psi; RRE, Rev response element; CPPT/CTS, central polypurine tract/central termination sequence; wPRE, woodchuck hepatitis virus post-transcriptional regulatory element; ITR, inverted terminal repeat of AAVs. (C) Design of donor templates denoted with their respective names and constructs’ length (bp). GFP, green fluorescence protein; TAG, translational stop codon. (D) Flow cytometry analysis of GFP expression in the CD11b-positive population upon granulocytic differentiation of PLB-985 *NCF1* ΔGT cells, treated with CRISPR-Cas9 RNP and IDLV *NCF1*-*GFP*, IDLV *GFP*, or AAV *GFP* with increasing MOI. (E) Flow cytometry analysis of p47^*phox*^ expression in the CD11b-positive population upon granulocytic differentiation of PLB-985 *NCF1* ΔGT cells, treated with CRISPR-Cas9 RNP and IDLV *NCF1*-*GFP*, IDLV *NCF1*, or AAV *NCF1* with increasing MOI. MOI is denoted as LP/cell for IDLVs and vg/cell for AAVs. Gating strategies and relative MFI of (D) and (E) are shown in Figures S2 and S3, respectively; *n* = 2–5, data are shown as mean ± SD. Statistical analysis in (D) and (E) was performed with two-way ANOVA followed by Sidak’s multiple comparisons test. (F) Flow cytometry analysis of p47^*phox*^ expression in the CD11b-positive population upon granulocytic differentiation of PLB-985 WT, non-treated (NT) PLB-985 *NCF1* ΔGT, or PLB-985 *NCF1* ΔGT treated with RNP + AAV *NCF1* (2e–6 vg/cell) or with RNP + IDLV *NCF1* (5e–3 LP/cell). Images are representative flow cytometry plots. (G) DHR test after granulocytic differentiation. Rhodamine 123 (Rho)-positive cells measured by flow cytometry upon stimulation with PMA of corresponding samples in (F). Fluorescent Rho indicates NADPH oxidase activity. For the data in (F) and (G) (*n* = 3), percent p47^*phox*^-positive cells, percent Rho-positive cells, their respective relative MFI, and gating strategies are shown in Figure S4. (H) Representative light microscopy images of NBT test performed on granulocytic-differentiated cells from corresponding samples in (F) upon PMA stimulation. Scale bars, 50 μm. Blue formazan precipitates indicate ROS production. Quantification of formazan-positive cells is shown in Figure S4G.

We used CRISPR-Cas9-mediated gene editing to integrate a full-length *NCF1* cDNA downstream of the *NCF2* reading frame (Figure 1A). First, we designed and identified a single-guide RNA (sgRNA) that targets the *NCF2* stop codon (Figures S1A, S1B, and S1D–S1F; Table S1). The donor template consisted of a foot-and-mouth disease virus (FMDV) 2A sequence,^26^ linked to a codon-optimized cDNA of the human *NCF1*, and flanked by two ∼0.5 kb homology arms on both ends, and was delivered into the cells by an adeno-associated viral vector (AAV) serotype 6, or by an integration-deficient lentiviral vector (IDLV) (Figure 1B). We hypothesized that by coupling the *NCF1* cDNA to the *NCF2* locus, p47^*phox*^ production should depend on the expression of the endogenous p67^*phox*^ and the separation by 2A-mediated ribosomal skipping (Figure 1A).^27^

We considered that corrective templates of different lengths may yield different knock-in efficiency, and therefore evaluated several templates: the construct 2A-*NCF1* (*NCF1*) that expresses the therapeutic p47^*phox*^, the reporter 2A-*GFP* (*GFP*), or a combination of 2A-*NCF1*-2A-*GFP* (*NCF1-GFP*) as a cDNA-directed reporter (Figure 1C). To assess the knock-in efficiency, we electroporated PLB-985 *NCF1* ΔGT cells^28^ with a ribonucleoprotein (RNP) complex consisting of Cas9 protein and an sgRNA targeting the *NCF2* stop codon (sgRNA *NCF2*), followed by transduction of the cells with viral vectors that carry the donor templates (Figures 1B and S1A). After this treatment, the edited cells were differentiated into granulocyte-like cells and analyzed for the presence of p47^*phox*^ protein (Figure S1C).

As expected, increasing multiplicity of infection (MOI) resulted in a dose-dependent increase of the percentage of transgene-expressing cells (Figures 1D and 1E). The use of short templates consistently improved the knock-in efficiency compared with the long templates: comparing IDLV *GFP* and IDLV *NCF1-GFP*, edited PLB-985 *NCF1* ΔGT cells resulted in 1.7-fold more green fluorescent protein (GFP)-expressing cells when treated with the highest MOI (5 × 10^3^ lentiviral particles [LPs]/cell, 35.6% ± 4.2% versus 21.3% ± 5.4%, respectively) (Figures 1D and S2). Similarly, the percentage of p47^*phox*^-expressing cells was consistently higher when a shorter IDLV *NCF1* construct was used at different MOIs (Figures 1E and S3). Due to the limited packaging capacity of AAV vectors and the size of the *NCF1-GFP* template (>3 kb), we only tested short AAV templates (AAV *GFP* and AAV *NCF1*) (Figures 1D, 1E, S2, and S3). The MOI that achieved the highest knock-in efficiencies (5 × 10^3^ LPs/cell for IDLV templates and 2 × 10^6^ vector genomes [vg]/cell for AAV templates) with no effect on viability of the edited PLB-985 *NCF1* ΔGT cells was chosen for subsequent experiments.

### Transgenic p47^*phox*^ expression restores the NADPH oxidase function in the edited PLB-985 *NCF1* ΔGT cells

In p47-CGD, defective NADPH oxidase results in the production of ROS by phagocytes.^2^^,^^16^ To investigate whether CRISPR-mediated knock-in of the *NCF1* cDNA at the *NCF2* locus restores ROS production, we performed functional tests on the edited PLB-985 *NCF1* ΔGT cells.

The knock-in of the *NCF1* cDNA at the *NCF2* locus restored p47^*phox*^ expression in 33.5% ± 5.3% and 32.2% ± 0.7% of granulocytic-differentiated cells when treated with RNP + AAV *NCF1* or IDLV *NCF1*, respectively (*n* = 3, Figure 1F). Since the transgenic p47^*phox*^ expression was controlled by the *NCF2* promoter, the level of p47^*phox*^ expression per cell was not expected to differ between AAV- or IDLV-mediated knock-in. Indeed, we found no significant difference in the relative p47^*phox*^-positive median fluorescence intensity (MFI) between the two vector types (Figure S4E) and the level of the transgenic protein expression was sufficient to restore ROS production in the edited PLB-985 *NCF1* ΔGT cells after granulocytic differentiation (Figures 1G, 1H, and S4).

### Transgenic p47^*phox*^ expression under the control of *NCF2* follows granulocytic differentiation of edited PLB-985 *NCF1* ΔGT cells

**Figure 2:**
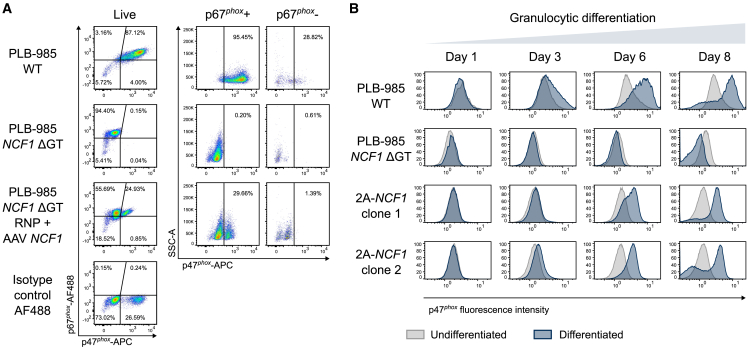
Transgene expression of p47^*phox*^ follows myeloid differentiation and expression of the endogenous *NCF2* gene (A) Flow cytometry analysis of p47^*phox*^ and p67^*phox*^ expression in total live cells upon granulocytic differentiation of PLB-985 WT, non-treated PLB-985 *NCF1* ΔGT, and PLB-985 *NCF1* ΔGT cells treated with RNP + AAV *NCF1*. The p47^*phox*^ expression was subsequently determined by gating on the p67^*phox*^-positive and -negative populations. The absence of p47^*phox*^ expression within the p67^*phox*^-negative population confirmed myeloid-determined expression of p47^*phox*^ under the control of *NCF2* encoding p67^*phox*^; *n* = 3. Gating strategies and replicates are shown in Figure S5. (B) Histogram showing p47^*phox*^ fluorescence intensity in PLB-985 WT, non-treated PLB-985 *NCF1* ΔGT, and two individual clones derived from PLB-985 *NCF1* ΔGT knocked in with 2A-*NCF1* vector over 8 days of granulocytic differentiation. Histograms of differentiated samples, overlaid with undifferentiated samples from respective bulk cultures, with relative cell count using modal y axis scaling.

To investigate if transgenic p47^*phox*^ expression follows granulocytic differentiation as intended, we analyzed PLB-985 *NCF1* ΔGT cells edited with RNP + AAV *NCF1* for p67^*phox*^ and p47^*phox*^ expression. We confirmed that, upon knock-in of the *NCF1* cDNA at the *NCF2*, p47^*phox*^ expression was limited to the p67^*phox*^-positive population (Figures 2A and S5A), indicating the desired regulation of the transgenic protein expression by the *NCF2* promoter.

To analyze the expression pattern of p47^*phox*^, we generated single PLB-985 *NCF1* ΔGT clones that were knocked in with one or two copies of 2A-*NCF1* (clones 1 and 2, respectively) and monitored them for p47^*phox*^ expression over 8 days of granulocytic differentiation. The 2A-*NCF1* knocked in clones exhibited a gradual increase of p47^*phox*^ MFI upon differentiation (Figures 2B and S5B), showing that p47^*phox*^ expression followed the physiological expression pattern of p67^*phox*^, which was driven by the endogenous *NCF2* promoter.

### Minimal transgene expression is detected before *in vitro* myeloid differentiation of edited p47^*phox*^-deficient mouse HSPCs

To analyze the applicability of the editing approach to the actual target cells for gene therapy, we assessed the knock-in efficiency in p47^*phox*^-deficient lineage-negative mouse HSPCs (mHSPCs) using the *NCF1* template. As the editing of HSPCs with IDLV vectors did not result in sufficient levels of transgene-expressing cells (<2.4% knock-in efficiency in treated bulk cultures, Figure S6), we subsequently used AAV vectors for template delivery.

**Figure 3:**
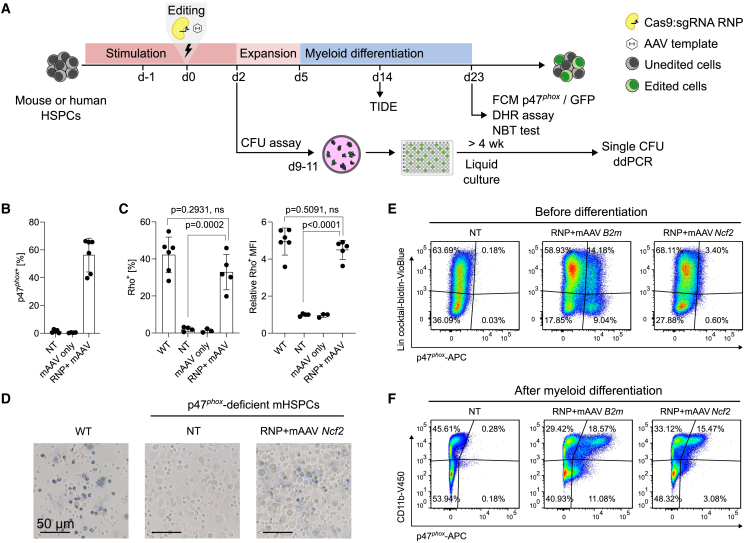
Myeloid-directed p47^*phox*^ expression shown in editing of p47^*phox*^-deficient mouse HSPCs (A) Experimental workflow of the knock-in strategy and analyses performed in mouse or human HSPCs. Mouse lineage-negative bone marrow HSPCs were derived from a p47^*phox*^-deficient mice. (B) Flow cytometry analysis of p47^*phox*^ expression upon myeloid differentiation of non-treated (NT) p47^*phox*^-deficient mHSPCs, p47^*phox*^-deficient mHSPCs treated with mAAV only (AAV *NCF1* template with homology arms targeting the mouse *Ncf2* gene), or treated with RNP + mAAV. (C) DHR test upon myeloid differentiation of mHSPCs. Percentage of Rho-positive cells (left) and their respective relative MFI (right) measured by flow cytometry upon stimulation with PMA of WT mHSPCs (as positive control) and corresponding samples in (B); *n =* 3–6, data are shown as mean ± SD. Statistical analysis was performed with ordinary one-way ANOVA followed by Tukey’s multiple comparisons test. (D) Representative light microscopy images from NBT test performed on myeloid-differentiated cells of NT, or RNP + mAAV *NCF1*-treated p47^*phox*^-deficient mHSPCs, and of WT mHSPCs upon PMA stimulation. Scale bars, 100 μm. Quantification of formazan-positive cells is shown in Figure S7E. (E) Flow cytometry analysis of p47^*phox*^ expression, gated on the lineage-negative population before differentiation of NT cells, cells treated with RNP + mAAV *NCF1* knocked in into a constitutive gene, *B2m* (RNP + mAAV *B2m*) or into the myeloid-specific *Ncf2* locus (RNP + mAAV *Ncf2*). (F) Flow cytometry analysis of p47^*phox*^ versus CD11b expression after myeloid differentiation of NT, RNP + mAAV *B2m*, or RNP + mAAV *Ncf2*-treated p47^*phox*^-deficient mHSPCs. Gating strategies and relative p47^*phox*^-positive or Rho-positive MFI in (B)–(F) are shown in Figure S7.

The knock-in protocol for HSPCs comprised electroporation of the Cas9 RNP targeting the mouse *Ncf2* in mHSPCs, followed by transduction of the AAV donor carrying the human *NCF1* cDNA (Figure 3A). Editing of p47^*phox*^-deficient mHSPCs restored p47^*phox*^ expression in 56.3% ± 12.2% of myeloid-differentiated cells (Figure 3B), and subsequently the restoration of ROS production, as measured by the dihydrorhodamine-1,2,3 (DHR) test (32.9% ± 9.5% Rho^+^ cells), and by the nitroblue tetrazolium (NBT) test (28.2% ± 2.7% formazan-positive cells) (Figures 3C, 3D, and S7A–S7D).

We confirmed that p47^*phox*^ expression upon knock-in into the *Ncf2* gene was restricted to the myeloid-differentiated cells (Figures 3F and S7D), with minimal transgene expression detected before differentiation (0.6% ± 0.2% lineage-negative, p47^*phox*^-expressing cells) (Figures 3E and S7C). In contrast to the *Ncf2* promoter, knock-in at a housekeeping gene, β-2-microglobulin (*B2m*),^29^ resulted in constitutive expression of p47^*phox*^, regardless of the differentiation status of the edited cells (Figures 3E and 3F).

### Human HSPCs can be efficiently targeted for *NCF1* cDNA knock-in *in vitro*

**Figure 4:**
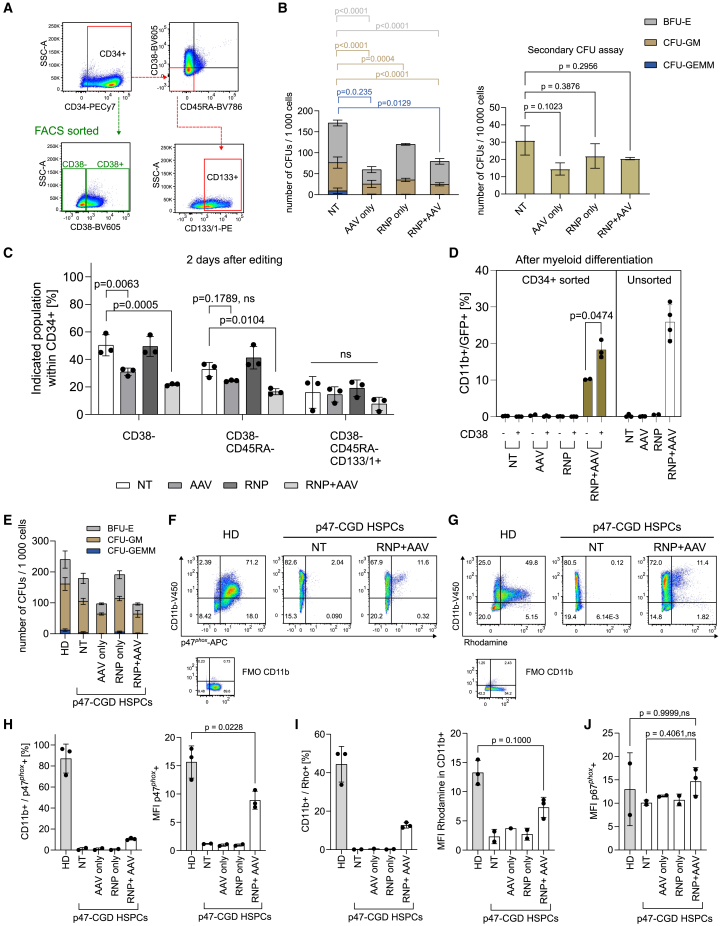
Application of knock-in approach in healthy and p47-CGD human HSPCs (A) Gating strategy for FACS sorting of CD38^−^/^+^ subpopulations and analysis of stem cell markers from hCD34^+^ HSPCs 2 days after editing. (B) CFU assay performed on non-treated (NT) healthy hCD34^+^ cells, treated with AAV *GFP* only, RNP only, or with RNP + AAV *GFP* (left graph). Secondary CFU assay was performed by replating 10 times the amount of cells from the same samples in the primary assay (right graph); *n* = 3 donors, with 2 technical replicates per donor; data are shown as mean ± SD; BFU-E, burst-forming unit-erythroid; CFU-G, colony-forming unit granulocyte; CFU-M, colony-forming unit macrophage; CFU-GEMM, colony-forming unit granulocyte, erythrocyte, monocyte, megakaryocyte; one-way ANOVA followed by Dunnett’s multiple comparisons test for CFU-GM and BFU-E, and Kruskal-Wallis test with Dunn’s multiple comparisons test for CFU-GEMM, to compare treated samples to NT only. Corresponding populations and significance values are indicated by corresponding fill and line colors, respectively. (C) Flow cytometry analysis of phenotypic HSC stemness markers on healthy control hCD34^+^ cells 2 days after editing; one-way ANOVA followed by Dunnett’s multiple comparisons test. (D) Flow cytometry analysis of GFP expression in the CD11b-positive population upon myeloid differentiation of CD34^+^ CD38^−^/^+^ sorted or unsorted healthy control hCD34^+^ cells as shown in (B); paired t test. In (B)–(D), *n* = 3 donors; data are shown as mean ± SD. (E) CFU assay performed on NT p47-CGD hCD34^+^ cells, treated with AAV *NCF1*, RNP, and RNP + AAV *NCF1*, with healthy hCD34^+^ (healthy donor [HD]) as control; *n* = 1 donor with 2 replicates per donor, data are shown as mean ± SD. (F and G) Representative flow cytometry plots showing (F) p47^*phox*^ and CD11b expression and (G) DHR test showing rhodamine 123-positive cells and CD11b expression. (H and I) Flow cytometry analysis of (H) p47^*phox*^ expression in the CD11b-positive population and their respective MFI in (F) and (I) rhodamine-positive cells in the CD11b-positive population and their respective MFI in (G). (J) Flow cytometry analysis of MFI p67^*phox*^ of corresponding samples in (H). In (F)–(J), *n* = 1 donor with 2 to 3 technical replicates, data are shown as mean ± SD. Statistical analyses in (H) and (I) were performed with unpaired t test and in (J) with Brown-Forsythe and Welch ANOVA tests, followed by Dunnett’s T3 multiple comparisons test. Gating strategies are shown in Figures S8 and S9.

Next, we treated human CD34^+^ (hCD34^+^) HSPCs isolated from healthy donors with an RNP + AAV *GFP* template, using the same protocol as for editing of mHSPCs. To investigate whether the knock-in treatment affects hematopoietic stem cell (HSC) stemness, we characterized the edited hCD34^+^ cells using key stem cell surface markers (CD34, CD38, CD45RA, CD133/1) that define cells with high hematopoietic reconstitution capacity and high colony forming activity (Figures 4A and S8A).^30^^,^^31^ We edited CD34-enriched cells and characterized them 2 days after editing *in vitro*. We found that AAV treatment reduced the percentage of CD34^+^ CD38^–^ CD45RA^−^ cells by 1.61 × (1.36)^±1^ times that of non-treated (NT) cells but did not significantly reduce the fraction of CD34^+^ CD38^–^ CD45RA^−^ CD133^+^ cells, which have been identified as highly enriched in long-term HSCs (Figures 4C and S8C). In addition, we performed a colony-forming unit (CFU) assay to evaluate the clonogenic potential of edited hCD34^+^ cells (Figures 4B, left, and S8B). In general, AAV treatment decreased the clonogenic potential of edited hCD34^+^ cells by 2.49 × (1.22)^±1^ times that of the NT control in the total number of CFUs, with significant decrease in number of burst-forming unit-erythroid (BFU-E) and colony-forming unit-granulocyte-macrophage (CFU-GM) (2.18 × (1.39)^±1^ times BFU-E and 2.72 × (1.00)^±1^ times CFU-GM lower than that of NT), mainly due to the cytotoxic effect of AAV treatment, which was also evident in our *in vitro* cultures of hCD34^+^ edited cells (Figure S8F). However, the clonogenic potential was retained upon serial CFU replating (Figure 4B, right), suggesting that this cytotoxic effect seems to mainly affect multipotent progenitors, rather than definite HSCs.

To determine the frequency of gene editing in CD34^+^ CD38^–^ and CD34^+^ CD38^+^ populations, we sorted CD34^+^ CD38^–^ and CD34^+^ CD38^+^ subsets 2 days after editing, alongside unsorted CD34-enriched cells (Figure S8A). These cells were differentiated into promyeloid cells in liquid culture and checked for knock-in efficiency by measuring GFP expression. While in the unsorted population we achieved knock-in efficiency of 25.9% ± 4.7%, a significant percentage of *in-vitro*-differentiated myeloid cells derived from the CD34^+^ CD38^–^ sorted cells were also edited (10.2% ± 0.1% GFP^+^ cells, Figures 4D, S8G, and S8H), suggesting that the HSC-enriched CD38^–^ fraction of CD34^+^ cells were targeted.

### Knock-in of *NCF1* cDNA at the *NCF2* locus functionally corrects p47-CGD patient HSPCs and does not affect endogenous p67^*phox*^ expression

We next evaluated the ability of our editing protocol (Figure 3A) to restore p47^*phox*^ expression and NADPH oxidase function in p47-CGD patient HSPCs. Bone marrow (BM) HSPCs derived from a p47-CGD patient were edited with an *NCF2*-targeted RNP + AAV *NCF1* donor template, and subsequently analyzed for clonogenic potential, p47^*phox*^ expression, and restoration of ROS production upon myeloid differentiation. Similar to editing in healthy HSPCs, AAV treatment reduced clonogenic potential in edited p47-CGD HSPCs (Figures 4E and S9E). We achieved 10.6% ± 1.0% of p47^*phox*^-expressing cells upon knock-in, with p47^*phox*^ expression levels in the corrected cells being slightly lower than that of healthy HSPCs (MFI p47^*phox*^-positive of 15.7 ± 2.8 in healthy versus 8.9 ± 1.6 in RNP + AAV *NCF1*; Figures 4F, 4H, and S9A). The achieved level of knock-in sufficiently restored NADPH oxidase function in 12.6% ± 1.4% cells (Figures 4G, 4I, and S9B). To evaluate the potential effect of knock-in at the *NCF2* locus, we analyzed for p67^*phox*^ expression in RNP + AAV *NCF1* p47-CGD bulk-treated HSPCs and found no reduction of p67^*phox*^ expression level (MFI p67^*phox*^) upon knock-in, suggesting that the knock-in did not affect endogenous expression of the *NCF2* gene (Figures 4J and S9A).

### Fusion proteins consisting of p67^*phox*^-2A-p47^*phox*^ negatively impact the NADPH oxidase function

**Figure 5:**
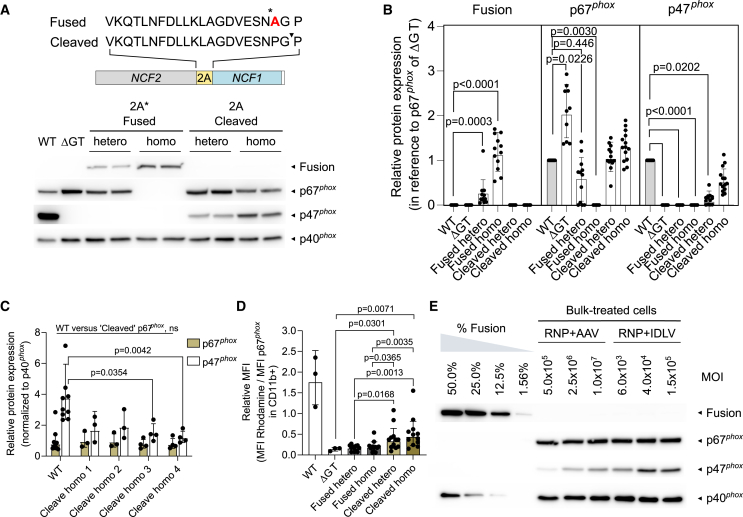
Analysis of potential fusion proteins caused by 2A oligopeptide activity upon *NCF1* knock-in (A) Western blot detecting fusion protein p67^*phox*^-2A∗-p47^*phox*^, p67^*phox*^, p47^*phox*^, and differentiation status loading control p40^*phox*^ upon granulocytic differentiation of PLB-985 WT cells, non-treated PLB-985 *NCF1* ΔGT cells, representative clones derived from PLB-985 *NCF1* ΔGT cells heterozygous or homozygous knock-in of a mutated 2A∗ oligopeptide carrying *NCF1* cDNA (denoted as 2A∗ Fused, hetero or homo), and representative clones knocked in with WT 2A oligopeptide carrying *NCF1* cDNA (denoted as 2A Cleaved, hetero or homo). Schematic representation of the 2A sequence in the respective constructs used for generating “Fused” and “Cleaved” clones. The P17A mutation (∗) introduced is highlighted in red. Arrow indicates 2A-mediated cleavage site between glycine-proline residue. (B) Relative protein expression of fusion p67^*phox*^-2A∗-p47^*phox*^, p67^*phox*^, and p47^*phox*^ quantified by western blots of samples in (A). Relative expression is represented in reference to PLB-985 WT after normalization with their respective differentiation status as indicated by p40^*phox*^ expression; *n =* 3 to 4 differentiation experiment from 4 clones per group; Kruskal-Wallis test with Dunn’s multiple comparisons test. (C) Protein expression of p67^*phox*^ and p47^*phox*^ quantified by western blot on PLB-985 WT and four PLB-985 *NCF1* ΔGT clones knocked in with 2 copies of *NCF1* cDNA; *n* = 4 clones with 3 to 4 differentiation experiments; Kruskal-Wallis test with Dunn’s multiple comparisons test. (D) Relative MFI rhodamine 123 normalized to MFI p67^*phox*^ expression in CD11b-positive cells from DHR test, performed on PLB-985 WT cells, non-treated PLB-985 *NCF1* ΔGT cells, heterozygous or homozygous Fused clones and Cleaved clones in (B); Kruskal-Wallis one-way ANOVA with two-stage step-up of Benjamin, Krieger and Yekutieli correction for multiple comparisons. Error bars for (B-D), data are shown as geometric mean ± geometric SD. (E) Western blot to analyze for presence of fusion proteins in bulk-treated PLB-985 *NCF1* ΔGT cells with RNP +AAV *NCF1* and RNP + IDLV *NCF1*. Cells were transduced with increasing MOI (vg/cell for AAVs and LP/cell for IDLVs). Protein expression was compared with a titration curve spiked with varying concentrations of fusion proteins. Unexposed and uncropped western blot images are shown in Figure S10. Representative flow cytometry plots and gating strategy are shown in Figure S11; data are shown as geometric mean × (geometric SD)^±1^.

Post-transcriptional processing of the transgenic 2A-*NCF1* sequence knocked in into the *NCF2* gene should result in the production of separate p67^*phox*^ and p47^*phox*^ proteins through 2A-mediated ribosomal skipping.^27^ In the case of an inefficient 2A activity, a fused translational product of p67^*phox*^-2A-p47^*phox*^ might be produced, which could negatively affect the protein’s function. In view of future clinical application, we analyzed the generation of unwanted p67^*phox*^-2A-p47^*phox*^ fusion proteins and analyzed their potential impact on the NADPH oxidase function: we generated four heterozygous and four homozygous clones from PLB-985 *NCF1* ΔGT cells (denoted as “Fused hetero” or “Fused homo”), which were knocked in with an *NCF1* vector that carried a non-functional 2A oligopeptide (denoted as 2A∗, carrying a mutation leading to a P17A amino acid change).^32^ Clones that carried the mutated 2A∗-*NCF1* vector produced fusion proteins of p67^*phox*^-2A∗-p47^*phox*^, as confirmed by western blot, as opposed to the “Cleaved” clones that produced separate p67^*phox*^ and p47^*phox*^ proteins (Figure 5A). As expected, Fused hetero clones expressed both the fused and the cleaved p67^*phox*^ proteins, while Fused homo clones expressed only the fusion proteins (Figures 5A, 5B, and S10A–S10D). Comparing p67^*phox*^ and p47^*phox*^ expression in differentiated homozygous knocked in clones (“Cleaved homo”) to PLB-985 wild-type (WT) cells, transgenic p47^*phox*^ expressed under the control of *NCF2* promoter were comparable with the endogenous p67^*phox*^ levels (Figure 5C), suggesting that the translation of p67^*phox*^-2A-p47^*phox*^ proteins by 2A-mediated skipping was close to the theoretical expression ratio of 1:1 (ratio of p67^*phox*^:p47^*phox*^ was 0.21 × (2.06)^±1^ in WT versus 0.55 × (1.63)^±1^ in Cleaved homo; *n* = 4 clones, three to four independent differentiation experiments; Figure 5C).

Results of the DHR test showed that fused p67^*phox*^-2A-p47^*phox*^ affected the differentiated cells’ ability to produce ROS compared with the cleaved counterparts (*n* = 16 clones, 0.15 × (1.67)^±1^ in Fused hetero and 0.16 × (1.94)^±1^ normalized MFI Rho in Fused homo versus 0.32 × (2.00)^±1^ in Cleaved hetero and 0.43 × (1.90) ^±1^ normalized MFI Rho in Cleaved homo; Figures 5D, S11A, and S11B).

Nonetheless, we found no p67^*phox*^-2A-p47^*phox*^ fusion proteins in bulk-treated PLB-985 *NCF1* ΔGT cells edited with either RNP + AAV or RNP + IDLV *NCF1*, confirming efficient 2A cleavage ability of our construct (Figures 5E and S10E).

### Knock-in treatment results in variable frequency of on- and off-target vector integrations and potential on-target deletions

**Figure 6:**
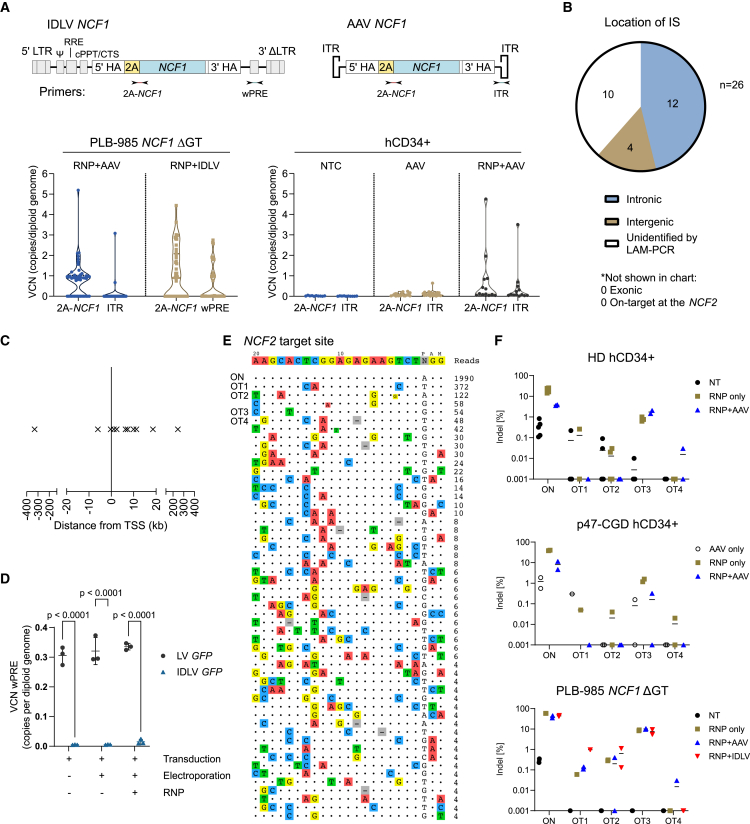
Assessment of unintended integrations and CRISPR off-targets upon knock-in treatment (A) VCN analysis of 2A-*NCF1* and AAV ITR or IDLV wPRE, as measured by ddPCR. The location of ddPCR primers targeting the vector is depicted by inverted arrows. VCN analysis was performed on individual clones derived from PLB-985 *NCF1* ΔGT cells, treated with RNP + AAV *NCF1* or RNP + IDLV *NCF1* (left graph, *n* = 46 clones per condition), and individual clones derived from non-treated (NT) human CD34^+^ cells, human CD34^+^ cells treated with AAV *NCF1* only, or treated with RNP + AAV *NCF1* (right graph, *n* = 10–21 clones per condition). The VCN of each target was normalized to 2 copies of the *FOXP2* housekeeping gene as reference diploid genome. Each dot in the violin plot represents a data point for an individual clone. Additional information on VCN of 2A-*NCF1* versus ITR/wPRE, flow cytometry analysis of p47^*phox*^ expression and results of the DHR test corresponding to each individual clone are presented in Figure S11. Data are shown as mean ± SD. (B) Pie chart showing the number of integration sites (ISs) by genomic location, as identified by LAM-PCR. The total number of ISs analyzed was based on the edited PLB-985 985 *NCF1* ΔGT clones that contain ITR or wPRE copies in (A) (*n* = 26 ISs predicted by ddPCR). (C) The distance from transcriptional start sites (TSSs) for individual ISs (*n* = 11 ISs that are within a gene). Each IS was depicted as a cross from the TSS at 0 kb. Detailed information of ISs is shown in Tables S5–S8. (D) VCN analysis of wPRE copies in PLB-985 *NCF1* ΔGT cells transduced with 5,000 lentiviral particles/cell of integration-competent LV *GFP* or IDLV *GFP*, compared with electroporated + transduced or electroporated with RNP + transduced samples. Data are shown as mean ± SD. (E) On- and off-target sites detected by CHANGE-seq for sgRNA *NCF2*. (F) The percentage of indels in edited hCD34^+^ cells from healthy donor (HD), or p47-CGD patient hCD34^+^ cells, and PLB-985 *NCF1* ΔGT cells confirmed by targeted amplicon deep sequencing at the on-target (ON) and four off-target (OT) sites detected by CHANGE-seq (Tables S9–S11); n = 2-6 biological replicates; data are shown as mean ± SD.

The use of CRISPR-Cas9 and viral vectors poses the risks of off-target (OT) vector integrations, which can be mediated either by undesired non-homologous end joining (NHEJ), microhomology-mediated end joining (MMEJ), or by the inherent low-frequency integration of AAVs and IDLVs.^33^^,^^34^^,^^35^^,^^36^ To identify vector integration events in the edited cells, we established a droplet digital PCR (ddPCR) approach to measure the copy number of 2A-*NCF1* vector, inverted terminal repeat (ITR) of AAV6, or the woodchuck hepatitis virus post-transcriptional regulatory element (wPRE) of IDLV (Figure 6A). With this method, we cannot determine if the integration took place at the on-target (*NCF2*) or OT sites, hence we refer to “unintended integrations” as any integration events that contained ITR/wPRE copies.

After editing, individual colonies were collected and grown in liquid culture for more than 4 weeks, and subsequently analyzed for vector copy number (VCN) (Figures 3A, 6A and S12). In PLB-985 *NCF1* ΔGT cells treated with RNP + AAV *NCF1* at MOI of 1 × 10^7^ vg/cell, we identified one clone with five copies of 2A-*NCF1* and three copies of ITR (denoted as 5N/3I), and one clone with one copy of 2A*-NCF1* and one copy of ITR (denoted as 1N/1I), indicating the occurrence of OT integrations (2/48 clones contained ITR copies, Figures 6A and S12C). Compared with the treatment with RNP + IDLV *NCF1* at MOI of 6 × 10^3^ LPs/cell, the OT events were markedly increased (14/24 clones contained wPRE copies, Figures 6A and S12D). The increase in number of clones containing wPRE copies could be associated with the increase in viral vector dose (MOI) (Figures S12D–S12F). In contrast to hCD34^+^ cells treated with RNP + AAV *NCF1*, we only identified two clones with 5N/4I and 1N/1I copies (2/13 clones contained ITR copies, Figures 6A and S12G). Interestingly, one clone with 0N/1I was detected with the AAV-transduced only samples (without CRISPR treatment; 1/21 clones), indicating that random AAV integration was present even without the induction of DSBs by Cas9 (Figures 6A and S12G).

In addition, we analyzed for the presence of deletions ∼1.3 kb upstream and downstream surrounding the targeted 3′ end of the *NCF2* locus by copy number quantification using ddPCR in knocked in clones. Copy number losses were detected in four clones, with three clones containing deletions upstream and one clone downstream of the insertion site (*n* = 18, AAV clones 17, 23, 25, and 26, Figures S12C and S13). Indeed, these clones did not express p47^*phox*^ and were unable to produce ROS (AAV clones 23 and 25, Figure S12C).

### Unintended integrations found in IDLV knocked in clones are due to the residual integrase activity of IDLVs

Although ddPCR VCN analysis can determine the frequency of unintended integrations, it does not provide information on their exact genomic locations. We therefore performed an integration site analysis by linear-amplification-mediated PCR (LAM-PCR)^37^ on the clones that contained ITR or wPRE copies that were identified by ddPCR in Figure 6A (2 clones contained ITR sequences and 14 clones contained wPRE sequences). Out of 16 clones that contained ITR or wPRE sequences, we expected to capture 26 integration sites (ISs) by LAM-PCR, corresponding to the total number of vector copies per clone.

We characterized the identified ISs in AAV and IDLV knocked in clones based on their genomic location, distance from a transcriptional start site (TSS), and presence of potential sgRNA binding sites in a 200 bp window upstream and downstream of an IS (Figures 6B and 6C; Tables S5–S7). Most of the ISs were in intronic regions (12/26 ISs), while a few were in intergenic regions (Figure 6B). We neither found IS in exons, nor at the *NCF2* on-target site (on-target integrations not mediated by HDR) (Table S5). Most of the ISs were enriched within 10 kb downstream of TSS (mean distance from TSS = 9.9 kb, Figure 6C). This observed integration pattern resembled the integration signatures typical for the lentiviral vectors.^38^^,^^39^ However, we confirmed the absence of integration-competent LV contamination in our IDLV preparation (Figures 6D and S15). As for AAV knocked in clones, no significant deductions can be made, because only two ITR-containing clones were identified and analyzed by LAM-PCR (clones 24 and 32, Figure S12C).

We further analyzed for CRISPR OT sites on the *NCF2* target gene by “circularization for high-throughput analysis of nuclease genome-wide effects by sequencing” (CHANGE-seq)^40^ (Figure 6E) and verified the OT sites by targeted amplicon sequencing on edited healthy hCD34^+^, p47-CGD hCD34^+^, and PLB-985 cells (*n =* 2–6, all samples below 1% indels on all OTs, except 7.7% ± 1.9% indels on OT3 in edited PLB-985 cells; Figure 6F; Tables S9 and S10). None of the ISs identified by LAM-PCR matched the CRISPR OT sites determined by CHANGE-seq, suggesting that the observed integration events are unlikely due to OT mediated by Cas9 nuclease activity. This was also confirmed by *in silico* prediction using CRISPRoff, Cas-OFFinder, or manual blastn alignment (search was limited to 200 bp upstream and downstream of the IS; OTs were predicted based on the sgRNA *NCF2* sequence mismatches; Table S5).^41^^,^^42^^,^^43^

## Discussion

In this study, we demonstrated the successful application of a CRISPR-mediated *NCF1* cDNA knock-in into the myeloid-regulated *NCF2* gene to correct p47-CGD. We have previously shown that direct targeting of the predominant *NCF1* ΔGT mutation is not suited for gene therapy, due to the induction of frequent chromosomal deletions at this genomic region, comprising the *NCF1* gene and its pseudogenes that share a high sequence similarity.^19^ Hence, we present an alternative approach that aims to avoid the induction of chromosomal deletions at the *NCF1* locus. Our approach has several advantages: the use of a promoter-less vector that lowers the risk of insertional activation in the case of OT integration.^44^^,^^45^^,^^46^^,^^47^ Importantly, without a vector-derived promoter, a near-physiological expression pattern of p47^*phox*^ should be achieved under the regulation of the endogenous *NCF2* promoter.

Since HSPCs are the target for *ex-vivo*-based long-term correction of p47-CGD, we aimed to limit the expression of p47^*phox*^ transgenic protein to the affected myeloid cells to avoid non-physiological activation of the NADPH oxidase and concomitant ROS production at the stem cell level. A balanced ROS level is crucial during hematopoiesis to support long-term repopulation ability of HSCs,^48^^,^^49^ whereas oxidative stress caused by dysregulated ROS production can affect stem cell migration, development, proliferation, and repopulation potential.^50^ By coupling the *NCF1* cDNA to the *NCF2* gene, the expression of p47^*phox*^ was closely linked to that of p67^*phox*^ following myeloid differentiation. However, this implies that the levels of p47^*phox*^ depend on the strength of the *NCF2* promoter, the overall knock-in efficiency, and the activities of homologous recombination (versus NHEJ, MMEJ, and inaccurate HDR).^33^^,^^47^^,^^51^ With the achieved ∼10% knock-in efficiency in p47-CGD patient HSPCs, we showed successful restoration of the NADPH oxidase activity *in vitro*. This level of correction should be sufficient for clinical correction, as in CGD only about 10%–20% of superoxide-producing cells were reported to be necessary for the reconstitution of host defense against infections.^52^^,^^53^^,^^54^

Apart from achieving a regulated expression of the therapeutic gene, correction of sufficient numbers of HSCs/LT-HSCs without impacting their stemness and engraftment potential is necessary for long-term treatment of CGD. Although sufficient gene correction levels of ∼26% were achieved in healthy hCD34^+^ cells, we observed reduced clonogenic potential and cell viability *in vitro*, especially after AAV treatment. Our data are in line with reports on the use of AAV6 for template delivery in HDR-based editing,^55^^,^^56^^,^^57^ indicating that the observed genotoxicity may be due to activation of p53-mediated DNA damage response upon concomitant exposure of HSPCs to CRISPR-induced DSBs and prolonged persistence of AAV genomes (ITRs) in the edited cells.^58^^,^^59^ We cannot judge on the potential toxic effects mediated by IDLV template delivery in HSCs as no correction was achieved with our construct. Another potential challenge with our approach could arise from the fact that the HDR mechanism specifically targets cycling cells (S/G2 phase), raising the possibility that a large fraction of dormant, long-term reconstituting HSCs will not be targeted. In our *in vitro* phenotypic analysis of edited hCD34^+^ cells, we found that the HSC-enriched subset (CD34^+^ CD38^–^ CD45RA^−^ CD133^+^) remained unchanged, and the clonogenic potential is retained upon secondary CFU replating. Nonetheless, the ability of edited HSCs to engraft in a CGD mouse model should be investigated further by *in vivo* transplantation studies, which could provide more evidence on the clinical potential of our editing approach.

Inefficient 2A-mediated ribosomal skipping results in the production of fusion proteins, which may negatively impact the protein’s function. Various forms of fused p67^*phox*^-p47^*phox*^ proteins have been reported to result in a constitutively active, highly stabilized NADPH oxidase in *in vitro* cell-free systems.^60^^,^^61^ Here, we showed that fusion proteins of p67^*phox*^-2A∗-p47^*phox*^ negatively affected ROS production in the edited cells. However, fusion proteins were undetectable with our current correction efficiency in bulk-treated cells.

As an additional safety assessment, we monitored the vector integration events by a ddPCR-based VCN determination method, LAM-PCR, and genome-wide unbiased CHANGE-seq. Random integrations of IDLVs and AAVs were reported to occur at relatively low levels (∼0.1%–2.3% for IDLVs and 0.1%–1% for AAVs),^35^^,^^62^^,^^63^^,^^64^ but were shown to be enhanced by nuclease-induced DSBs in the genome.^36^^,^^65^ We detected minimal levels of unintended integrations in hCD34^+^ and in PLB-985 *NCF1* ΔGT cells when knocked in with the AAV *NCF1* donor template compared with IDLVs. Interestingly, several knocked in clones that contained truncated 2A-*NCF1* copies or had deletions (∼1.3 kb) surrounding the *NCF2* target site did not express p47^*phox*^ and did not produce ROS (5/92 clones), indicating that not all knock-in sequences constituted an in-frame seamless integration, and that on-target deletions cannot be avoided upon Cas9-mediated DSBs.^66^^,^^67^ Since VCN quantification relies on the presence of the primer binding sites, not all forms of repair mechanisms (HDR, NHEJ, or MMEJ)^33^ could be detected and distinguished. We also found that the unintended integrations were not restricted only to CRISPR-treated samples (samples without transduction with the viral vector template), suggesting that random AAV integrations are present and could be of potential concern.^68^^,^^69^^,^^70^^,^^71^

Using LAM-PCR on IDLV knocked in clones derived from the edited PLB-985 cells, the identified ISs resembled integration signatures of lentiviral vectors.^38^^,^^39^ Whether the same ISs would also be retrieved in edited hCD34^+^ cells was not investigated in this setting. Importantly, a near-diploid model cell line like PLB-985 has a reported 4% polyploidy^72^ calls for copy-number variation and quantitative integration site analysis on the appropriate primary cells instead. We did not find any potential gRNA binding sites surrounding the ISs, thus could not establish a link between the observed integrations and the CRISPR-induced off targets.^36^^,^^58^ From our observation, we confirmed that the unintended integrations were mediated by residual lentiviral integrase activity in the IDLVs^73^ and not by CRISPR-induced OT DNA breaks or contamination from integration-competent LVs in the IDLV preparation. To address the observed CRISPR-induced OTs in the edited HSPCs, albeit at a low frequency (e.g., 0.32%–2.06% indels on OT3), the use of HiFi Cas9 for instance can improve the targeting specificity.^74^

Compared with IDLVs, our data showed that AAV template delivery resulted in fewer OT integrations and exhibited higher efficacy than using IDLV in HDR-based gene editing, as opposed to a recent report by Ferrari et al.^58^ Differences in our editing protocol, i.e., longer HSPC stimulation period, use of the electroporation enhancer or transduction enhancer cyclosporin H, and the order of transduction and electroporation, may account for the different findings in our study. However, we cannot exclude the reported genotoxicity risks of ITR trapping in the edited HSPCs because of differences in the detection methods used by the group.

Recently, a knock-in approach based on cDNA insertion has been explored for the treatment of p47-CGD by Klatt et al.^15^ In that study, a “minigene” insertion (cDNA comprising *NCF1* exons 2–11) was targeted to a unique 3 bp polymorphism in the *NCF1* intron 1, preventing cleavage of the *NCF1* pseudogenes. This approach cannot be applied to all p47-CGD patients, as gene conversion in p47-CGD usually results in the transfer of large stretches of pseudogene sequence onto the *NCF1*.^75^ As the crossover events between *NCF1* and its pseudogenes vary between patients, single-nucleotide polymorphisms are typically not shared between patients. Another potential approach to correct the predominant ΔGT deletion without the risks of Cas9-induced DSBs^19^ is prime editing.^76^ Prime editing presents a more streamlined correction procedure without viral vectors or targeting of other loci, but in common with base/prime editors still carries risks of chromosomal rearrangements,^77^ potentially due to conversion of single-strand breaks into DSBs,^78^ which was also observed in our attempt to correct the ΔGT deletion using Cas9 nickases (unpublished data). Nonetheless, our approach has the advantage of avoiding the complexity of editing at the highly homologous *NCF1* gene and the pseudogene loci. Importantly, knock-in of a full-length *NCF1* cDNA has the benefit of being applicable to treat all genetic forms of p47-CGD, not only the *NCF1* ΔGT mutation.

In conclusion, the CRISPR-based knock-in approach downstream of *NCF2* we propose has proven efficacious for the treatment of p47-CGD in relevant cellular models, with the transgene expression following myeloid differentiation. The editing approach also has the potential to be adapted for the treatment of other diseases where myeloid-directed expression is required. However, our findings on OT integrations and the associated genotoxicity when the viral template delivery is used emphasize the general need for a more comprehensive risk-benefit assessment for future clinical translation of nuclease- or AAV/IDLV vector-based therapies.

## Materials and methods

### Cell culture

The human myeloid leukemia cell lines, PLB-985 WT and PLB-985 *NCF1* ΔGT, were cultured in RPMI 1640 medium (PAN-Biotech, Germany) supplemented with 10% (v/v) fetal bovine serum (FBS) (PAN-Biotech, Germany), 10 mM HEPES, 100 U/mL penicillin, and 100 mg/mL streptomycin (Thermo Fisher Scientific, USA). PLB-985 cells are models of granulocyte-like cells, therefore *in vitro* differentiation of these cells was termed as “granulocytic differentiation.” For granulocytic differentiation, cells were plated at a density of 0.8 × 10^6^ cells/mL in RPMI 1640 medium, supplemented with 5% (v/v) FBS, 0.5% (v/v) N,N-dimethylformamide (DMF) (Sigma-Aldrich, USA), 100 U/mL penicillin, and 100 mg/mL streptomycin for 7 days. Generation of individual clones were performed by fluorescence-activated cell sorting (FACS) with BD FACSAria III Cell Sorter (BD Biosciences, USA).

Human CD34^+^ cells from mobilized peripheral blood of healthy donors were purchased from Lonza (Switzerland). The p47-CGD CD34^+^ cells were obtained from a CGD donor under written informed consent. Sample collection and processing were performed in accordance with ethical principles, applicable local laws and regulations (ethics vote KEK ZH 2015/0135, BASEC-Nr. PB_2016-02202). CD34^+^ cells were thawed and stimulated in X-VIVO 20 medium (Lonza, Switzerland), supplemented with 300 ng/mL recombinant human (rh) stem cell factor (SCF), 300 ng/mL rh FMS-like tyrosine kinase 3 ligand (Flt3-L), 100 ng/mL rh thrombopoietin (Sartorius CellGenix, Germany), and 1% (v/v) human serum albumin (HSA) (CSL Behring, Germany) for 1–2 days before electroporation and transduction procedures. For expansion and differentiation, cells were cultured in IMDM medium (Lonza, Switzerland), 10% (v/v) FBS, 50 U/mL penicillin, 50 mg/mL streptomycin, supplemented with 100 ng/mL rh SCF, 100 ng/mL rh IL-3 (Sartorius CellGenix, Germany), for 3 days, differentiated for 8 days with 100 ng/mL rh SCF and 10 ng/mL rh granulocyte colony-stimulating factor (G-CSF) (PeproTech, USA), and for another 9 days with only 10 ng/mL G-CSF. Differentiation of hCD34^+^ toward myeloid lineages was termed myeloid differentiation, in contrast to granulocytic differentiation of PLB-985 cells.

Lineage-negative BM mHSPCs from p47-deficient mice or WT mice were cultured for 1–2 days in the medium of the same composition as described for hCD34^+^ cells, but with recombinant murine (rm) cytokines (PeproTech, USA). For expansion and myeloid differentiation, the same medium composition was used, except with rm cytokines for a 3-day expansion, 4-day differentiation with rm SCF, rm IL-3, and rm G-CSF, followed by 4-day differentiation with only G-CSF.

All cell cultures were kept at 37°C in a humidified CO_2_ incubator with 5% CO_2_.

### Isolation of BM-derived mHSPCs

WT B6 mice (strain C57BL/6J, JAX: 000664) and p47^*phox*−/−^ (strain B6(Cg)-*Ncf1*^*m1J*^/J, JAX: 004742) mice were maintained at the Laboratory Animal Services Center (LASC), University of Zurich, Schlieren, Switzerland. The animals were housed under specific pathogen-free conditions with unlimited access to water and food. All procedures were conducted in accordance with the Swiss legislation on animal protection and welfare (Tierschutzverordnung, TSchV 455.163/Tierschutzgesetz, TSchG 455) and were approved by the Cantonal Veterinary Office (VETA) and the Cantonal Commission for Animal Experimentation (TVK) in Zurich (license no. ZH084/2019). All mice were physically examined and acclimatized for a minimum of 7 days, followed by euthanasia with CO_2_ inhalation. BM cells were obtained from femur, tibia, humerus, and pelvis. The excised bones were crushed with a mortar and a pestle in PBS (Thermo Fisher Scientific, USA) supplemented with 1% (v/v) HSA, 100 U/mL penicillin, and 100 mg/mL streptomycin. The BM cell suspension was then filtered through a 70- and 40-μm cell strainer (LubioScience, Switzerland), and subjected to lineage depletion to obtain Lin^–^ cells using Direct Lineage Cell Depletion Kit (Miltenyi Biotec, Germany) according to the manufacturer’s instructions.

### Vector design

Repair template 2A-*NCF1* and control template 2A-*GFP* were synthesized by GeneArt Subcloning and Plasmid services (Thermo Fisher Scientific, USA) with 2A peptide sequence from FMDV X00871; nts 3,483–3,475, NCF1 from NM_000265.5; nts 74–1,240 (codon-optimized), and enhanced GFP (eGFP) from pcDNA3-EGFP. The plasmid pcDNA3-EGFP was provided by Doug Golenbock (Addgene, plasmid no. 13031; http://n2t.net/addgene:13031). Homology arms with 501 bp each were PCR amplified from PLB-985 genomic DNA (gDNA), flanking the human *NCF2* NG_007267.1 or mouse *B2M* NC_000068.8 upstream and downstream of the transcription stop codon (TAG). IDLV constructs were constructed by inserting the corresponding transgene into the XhoI and XmaI sites on self-inactivating (SIN) SIN HIV-1 lentiviral plasmid backbone by Gibson assembly (New England Biolabs [NEB], USA). AAV6 vectors were generated by cloning the transgene (sequence shown in Table S9) into the KpnI and SalI sites on the p24 plasmid (Repository ID: p24, pssAAV-2-CAG-EGFP-WPRE-SV40p(A), Viral Vector Facility, University of Zurich) by Gibson assembly.

### Viral vector production

AAV and IDLV vectors were produced by the Viral Vector Facility (VVF) of the Neuroscience Center Zurich (ZNZ). To produce IDLVs, HEK293T cells were transfected with transfer plasmid containing donor templates as described above, *gag*-*pol* packaging plasmid pSLQ8001-pCMV-R8.91 (D64V) provided by Stanley Qi (Addgene, plasmid no. 202687; http://n2t.net/addgene:202687; RRID:Addgene_202687) and VSV-G envelop plasmid pMD2.G provided by Didier Trono (Addgene, plasmid no. 12259; http://n2t.net/addgene:12259; RRID:Addgene_12259). Viral vector supernatant was precipitated by PEG6000 and resuspended in sterile PBS (pH 7.4). The viral titer was quantified by QuickTiter Lentivirus Titer Kit (HIV p24) ELISA kit (Cell Biolabs, USA) and expressed as LPs/mL.

To produce single-stranded AAVs, HEK293T cells were transfected with AAV helper plasmid serotype 6 (cap ORF: AF028704.1), adenovirus helper plasmid pBS-E2A-VA-E4,^79^ and transfer plasmid containing donor templates as described above. Viral vector supernatant was purified by OptiPrep density gradient ultracentrifugation and diafiltration, followed by resuspension in PBS (pH 7.4), 1 mM MgCl_2_, 2.5 mM KCl. The viral titer was quantified by fluorometric method using a Qubit dsDNA HS kit (Thermo Fisher Scientific, USA) and expressed as vg/mL.

### sgRNA design

The *Streptococcus pyogenes* Cas9 (SpCas9) sgRNAs were designed using the CHOPCHOP v.3 web tool^80^ (https://chopchop.cbu.uib.no/) and purchased from Integrated DNA Technologies (Alt-R CRISPR-Cas9 sgRNA).

### Cas9 nuclease preparation

The *S. pyogenes* (*Sp*)Cas9 expression vector (Addgene, no. 78312; pMJ922 from Martin Jinek; http://n2t.net/addgene:78312)^81^ encodes a Cas9 fusion protein with an N-terminal hexahistidine-maltose binding protein tag (6xHis-MBP) followed by a tobacco etch virus (TEV) protease cleavage site and a C-terminal hemagglutinin (HA) tag, GFP tag, and three nuclear localization signals (NLSs) yielding a 6xHis-MBP-TEV-Cas9-HA-2xNLS-GFP-NLS construct. Purification of Cas9 was carried out as described,^82^^,^^83^ with minor adjustments. In brief, Cas9 construct was expressed in *E. coli* BL21 Rosetta2 (DE3) cells (Merck KgaA, Germany). Cells were lysed in 20 mM Tris (pH 8.0), 500 mM NaCl, 5 mM imidazole, 1 μg/mL pepstatin, 200 μg/mL AEBSF by ultrasonication. Clarified lysate was applied to a 10 mL Ni-NTA (Sigma-Aldrich, USA) affinity column. The column was washed with 20 mM Tris (pH 8.0), 500 mM NaCl, and 10 mM imidazole, and bound protein was eluted by increasing imidazole concentration to 250 mM. Eluted protein was dialyzed against 20 mM HEPES (pH 7.5), 250 mM KCl, 10% glycerol, 1 mM dithiothreitol (DTT), and 1 mM EDTA overnight at 4°C in the presence of TEV protease to remove the 6xHis-MBP affinity tag. Cleaved protein was further purified using a HiTrap HP Heparin column (GE HealthCare, USA), eluting with a linear gradient to 1.0 M KCl. Elution fractions were pooled, concentrated, and further purified by size-exclusion chromatography using a Superdex 200 (16/600) column (GE HealthCare, USA) in 20 mM HEPES-KOH (pH 7.5), 500 mM KCl, and 01 mM DTT, yielding pure, monodisperse proteins. Aliquots were flash-frozen in liquid nitrogen and stored at −80°C.

### CRISPR-Cas RNP electroporation and donor template delivery

For electroporation of hCD34^+^ cells, 18 pmol of SpCas9-GFP protein and 36 pmol of sgRNA were incubated for 15 min at room temperature to form an RNP complex. A total of 1 × 10^5^ cells was combined with RNP complex in 10 μL Resuspension Buffer T, and subjected to electroporation at 1,400 V, 10 ms, 3 pulses using the Neon Transfection System 10 μL kit (Thermo Fisher Scientific, USA). Within 30 min after electroporation, cells were transduced with IDLV or AAV donor at the indicated MOI in LPs/cell or vg/cell, respectively, and incubated in a CO_2_ incubator at 37°C 5% CO_2_ for 2 h, followed by 80% (v/v) medium change. For electroporation of PLB-985 cells, 2 × 10^5^ cells were combined with RNP complex in Resuspension Buffer R and subjected to electroporation at 1,150 V, 10 ms, 3 pulses. For electroporation of mHSPCs, 2 × 10^5^ cells were combined with RNP complex in Resuspension Buffer T and subjected to electroporation at 1,700 V, 20 ms, 1 pulse.

### TIDE analysis

For tracking of indels by decomposition (TIDE) analysis,^84^ the target sequence was amplified using Phusion High Fidelity DNA Polymerase (primers are shown in Table S3). PCR products were purified using QIAquick PCR Purification Kit (QIAGEN, Germany), analyzed by 1% agarose gel electrophoresis, and sent for Sanger sequencing (Microsynth, Switzerland). Sequence traces from control and treated samples were analyzed using the TIDE web tool (https://tide.deskgen.com/).

### VCN analysis by ddPCR

The VCN in single clones was determined by ddPCR using a QX200 Droplet Digital PCR System (Bio-Rad Laboratories, USA). Genomic DNA was digested with DraI (20 U/μL) (NEB, USA) for 1 h at 37°C followed by heat inactivation for 20 min at 65°C. 3–4 ng/μL of DraI-digested gDNA was used per ddPCR reaction, containing ddPCR supermix for robes (no dUTP) (Bio-Rad Laboratories, USA), 900 nM primers, and 250 nM probes. A total of 20 μL of sample reaction was transferred to a DG8 Cartridge for the QX200/QX100 Droplet Generator and processed according to the manufacturer’s instructions. Droplets containing DNA samples were first denatured for 10 min at 95°C, and then subjected to 40 cycles of amplification comprising consecutive steps of denaturation (95°C, 30 s), annealing (60°C, 1 min), and extension (72°C, 1 min), with a final 10 min incubation at 95°C. Following amplification, samples were analyzed using QX200 Droplet Reader and QuantaSoft Analysis Pro software v.1.0 (Bio-Rad Laboratories, USA). The values of the VCN per cell were quantified with reference to a housekeeping gene, *FOXP2.* Primers used are shown in Table S2.

### Flow cytometry

Flow cytometry analysis was performed using a BD LSRFortessa (BD Biosciences, USA) or a MACSQuant Analyser 10 flow cytometer (Miltenyi Biotec, Germany). Extracellular staining was performed using anti-CD11b, anti-CD14, and anti-CD15 for myeloid cells, or anti-CD34, anti-CD38, anti-CD45RA, and anti-CD133/1 antibodies for HSCs and a viability dye after incubation with an FcR blocking reagent (Miltenyi Biotec, Germany), followed by fixation with 1% (v/v) paraformaldehyde (Sigma-Aldrich, USA). Cells were permeabilized with 0.1% saponin (Sigma-Aldrich, USA) and stained intracellularly with anti-p47^*phox*^ and anti-p67^*phox*^ antibody. Endogenous GFP expressions were analyzed *in situ*. In mHSPCs, LSK cells were stained by lineage cell detection cocktail conjugated with biotin, anti-c-Kit, and anti-Sca-1, followed by secondary staining with anti-biotin (Miltenyi Biotec, Germany). Fluorescence minus one controls and isotype controls were used when appropriate (antibodies, dyes, and reagents are listed in Table S4).

For DHR test, CD11b-labeled cells were incubated with 10 μM DHR 123 (Sigma-Aldrich, USA) and 3,450 U/mL catalase from bovine liver (Sigma-Aldrich, USA) in PBS containing 0.05% (w/v) gelatin from cold water fish skin (Sigma-Aldrich, USA) and 0.9 g/L glucose (Thermo Fisher Scientific, USA) for 15 min at 37°C in 5% CO_2_, followed by stimulation with 1 μg/mL phorbol 12-myristate 13-acetate (PMA) (Sigma-Aldrich, USA) for 15 min at 37°C in 5% CO_2_ before measurement.

### Western blot

Total proteins were isolated from whole cells by protein lysis using M-PER Mammalian Protein Extraction Reagent (Thermo Fisher Scientific, USA). Upon centrifugation for 30 min at 16,000 × *g* and at 4°C, protein supernatant was collected, and concentration was measured by Bradford assay (Sigma-Aldrich, USA). Protein supernatant was resuspended in 4× Laemmli sample buffer (Bio-Rad Laboratories, USA) with 1:100 (w/v) β-mercaptoethanol (Sigma-Aldrich, USA). Prior to SDS-PAGE, protein samples in sample buffer were then denatured at 95°C for 5 min and cooled for 10 min at room temperature. A total of 80 μg proteins per sample were loaded on a 4%–12% polyacrylamide gel by SDS-PAGE using Mini-PROTEAN Tetra Cell electrophoresis system, followed by wet transfer overnight at 4°C onto a 0.2 μm PVDF membrane using Mini Trans-Blot Cell transfer system (Bio-Rad Laboratories, USA). The membranes were blocked with 5% skimmed milk and immune-stained using primary antibodies against p47^*phox*^, p67^*phox*^, and p40^*phox*^ in 5% skimmed milk overnight at 4°C, followed by secondary staining with anti-mouse conjugated to horseradish peroxidase. The signal was developed by incubation of the membrane in SuperSignal West Pico PLUS Chemiluminescent Substrate (Thermo Fisher Scientific, USA), visualized with Amersham ImageQuant 800 (Cytiva, USA). Band intensities were subjected to densitometric analysis using ImageJ software (National Institutes of Health, USA).^85^ To determine the expression of myeloid-dependent p67^*phox*^ and p47^*phox*^ proteins, their band intensities were first normalized to p40^*phox*^ as a differentiation marker (DM), by protein of interest (PI)/DM. To ensure quantification of band intensities are comparable across different blots, WT protein sample was loaded on every blot as an internal control reference. The WT samples were differentiated alongside treated samples of the same replicate and loaded on its respective blot. A second normalization was then performed by (PI/DM) of sample/(PI/DM) of WT to determine the relative protein expression.

### NBT

A total of 1 × 10^5^ cells of differentiated PLB-985 cells were plated on a 96-well plate and were incubated with 100 μg/mL PMA (Sigma-Aldrich) and 200 ng/mL NBT for 30 min at 37°C and 5% CO_2_, followed by cell fixation in 1% (w/v) formaldehyde. Fixed cells were analyzed visually for the presence of formazan precipitates using a Leica DM IL Fluo light microscope, equipped with a DFC420 digital camera and Leica application suite acquisition software (Leica Microsystems, Germany).

### Hematopoietic CFU assay

A total of 1,000 hCD34^+^ cells was plated in MethoCult H4434 Classic (STEMCELL Technologies, Germany) in a 35-mm cell culture dish and incubated at 37°C in a humidified CO_2_ incubator with 5% CO_2_ for 10–14 days. Secondary replating was performed with a total of 10,000 cells collected from the primary CFU plates, pelleted, washed twice in X-VIVO 20 medium, and replated with the same conditions described above. The identity of formed CFUs was determined visually using a Leica EZ4 Stereo Microscope (Leica Microsystems, Germany). Individual CFUs were counted, and collected for gDNA lysis in Buffer K. The data were represented as a total CFU number and a frequency of each CFU type.

### LAM-PCR

A total of 300 ng gDNA was used to perform linear amplification of vector-genome junctions using biotinylated primers LTR I or ITR I. The pre-amplified DNA-bead complex was separated by magnetic beads using the Dynabeads kilobaseBINDER kit (Thermo Fisher Scientific, USA) according to the manufacturer’s instructions, followed by dsDNA synthesis using random hexanucleotide as primers and Klenow polymerase (NEB, USA). After two washing steps with DNase-free water, the DNA-bead complex was digested with TasI enzyme and ligated with a linker cassette for 1 h at room temperature. After two washing steps with DNase-free water, the linker-ligated fragments were released from magnetic beads by denaturation using 0.1 N sodium hydroxide solution for 5 min at room temperature. The released ssDNA fragments were then used as a template for the first nested PCR, followed by a SacI digest of the internal control and the second nested PCR. The protocol was adapted from Schmidt et al.^37^ and primers used in each step are listed in Table S6. Amplicons from the second nested PCR were subcloned into the pCR Blunt II-TOPO vector using Zero Blunt TOPO PCR Cloning Kit (Thermo Fisher Scientific, USA), and sequences were identified by Sanger sequencing (additional methods used downstream of LAM-PCR are described in the supplemental information).

### CHANGE-seq

The sgRNA *NCF2* was first tested for functionality by digesting a PCR amplicon of the genomic target site *in vitro*. NT and Cas9 sgRNA *NCF2* RNP-treated human CD34^+^ cells from healthy donors (three technical replicates per sample) were used for this method. The CHANGE-seq experiment was performed by Functional Genomics Center Zurich (FGCZ). Next-generation sequencing data were processed using the CHANGE-seq analysis pipeline (https://github.com/tsailabSJ/changeseq) with the following parameters: read_threshold, 4; window_size, 3; mapq_threshold, 50; start_threshold, 1; gap_threshold, 3; mismatch_threshold, 10; search_radius, 30; and merged_analysis, True. Raw data of the CHANGE-seq results is provided in Table S11. Next-generation sequencing (NGS) sequencing data are deposited at NCBI Sequence Read Archive (NCBI BioProject: PRJNA1085720).

### Indel analysis by NGS

Four selected OT sites were deep sequenced (detailed number of reads, NGS data summary information, and primer sequences are provided in Tables S9 and S10). Amplicons for deep sequencing were generated using two rounds of PCR to attach Illumina handles. Q5 high-fidelity polymerase and 150 ng of gDNA in a total volume of 15 μL were used. The thermal cycling profile of the PCR was: 98°C 30 s; 25 × (98°C 30 s; annealing [see Table S9] 30 s; 72°C 30 s); 72°C 5 min. i5 and i7 Illumina adapters were added in a second PCR reaction using Q5 high-fidelity polymerase (NEB, USA) and 1 μL of a first-step PCR product in a total volume of 15 μL. The thermal cycling profile of the PCR was: 98°C 30 s; 15 × (98°C 30 s, 72°C 1 min); 72°C 5 min. Approximately equal amounts of PCR products from each sample were pooled, gel purified, quantified using a Qubit fluorometer with a dsDNA HS Assay Kit (Thermo Fisher Scientific, USA) and were 2 × 150 bp paired-end sequenced on MiSeq (Illumina, USA). NGS amplicon sequencing data are deposited at NCBI Sequence Read Archive (NCBI BioProject: PRJNA1085720).

### Statistical analysis

Statistical analysis was performed using GraphPad Prism v.8.4.3 for Windows, GraphPad Software, www.graphpad.com. Parametric tests were used for group-wise comparisons if data assumed normal distribution when tested by QQ plot, otherwise non-parametric tests were used. Interpretations for ratios comparing treated and control samples, i.e., in Figures 5B–5D, were represented in geometric mean × (geometric SD)^±1^. Each specific statistical test is indicated in the figure legends.

## Data and code availability

The data supporting the findings of this study are available in the supplemental information and additional details upon request from the corresponding author, Janine Reichenbach.

## Supplemental information

Document S1. Figures S1–S14 and Table S1–S11

Document S2. Article plus supplemental information
