# Supplementary material for: Targeted knock-in of NCF1 cDNA into the NCF2 locus leads to myeloid phenotypic correction of p47phox-deficient chronic granulomatous disease
Source: Mol Ther Nucleic Acids. 2024 May 24;35(3):102229. doi: 10.1016/j.omtn.2024.102229 (PMC11215332; doi:10.1016/j.omtn.2024.102229)
Supplement: Document S1. Figures S1–S14 and Table S1–S11 [file mmc1.pdf]

OMTN, Volume *35*

Supplemental information

Targeted knock-in of *NCF1* cDNA into the *NCF2* locus leads to myeloid phenotypic correction of p47^{phox}-deficient chronic granulomatous disease

Kah Mun Siow, Merve Güngör, Dominik Wrona, Federica Raimondi, Oleksandr Pastukhov, Panagiotis Tsapogas, Timon Menzi, Michael Schmitz, Péter István Kulcsár, Gerald Schwank, Ansgar Schulz, Martin Jinek, Ute Modlich, Ulrich Siler, and Janine Reichenbach

Supplemental Material

Supplemental Methods

Cas12a nuclease preparation

The *Acidaminococcus sp.* (As)Cas12a gene was amplified by PCR (Addgene plasmid #90095; 6His-MBP-TEV-huAsCpf1 was provided by Feng Zhang; http://n2t.net/addgene:90095)[1] to replace the Cas9 gene in the Cas9 expression vector by Gibson assembly, generating the 6xHis-MBP-TEV-Cas12a-HA-2xNLS-GFP-NLS expression construct. Purification of Cas12a was done as described.[2,3] Cas12a construct was expressed in *E. coli* BL21 Rosetta2 (DE3) cells (Merck KgaA, Germany). Eluted protein was dialyzed against 20 mM HEPES pH 7.5, 250 mM KCl, 1 mM dithiothreitol (DTT), 1 mM EDTA (Cas12a) overnight at 4 °C in the presence of TEV protease to remove the 6xHis-MBP affinity tag. The subsequent procedures (clarification, concentration, and purification steps) were performed as described in the 'Materials and methods' section.

In vitro cell-free cleavage assay

PCR product (857 bp) containing the *NCF2* on-target site was cloned into pCR Blunt II-TOPO vector, using Zero Blunt TOPO PCR Cloning Kit (Thermo Fisher Scientific, USA). The plasmid was linearized using SmaI (NEB, USA) as a target DNA for *in vitro* cleavage assay. Prior to RNP complexing, gRNA was denatured at 95 °C for 2 min and incubated at room temperature for 30 min. RNP was complexed with 1.5 µM of Cas9-GFP protein and 1.5 µM of gRNA for 15 min at room temperature. 55 ng of linearized target DNA was mixed with RNP complex and incubated for 1 hour at 37 °C. Reaction was stopped by addition of 0.02 M ethylenediamine tetraacetic acid (EDTA) and 1 µg/µL proteinase K (Roche, Switzerland), and incubated in a thermocycler (Biometra TOne, Analytik Jena, Germany) at 37 °C for 30 min. Digestion products were visualized on 1% agarose gel following electrophoresis. Band intensities were measured by densitometric analysis using ImageJ software (National Institutes of Health, USA).[1] Cleavage efficiency resulting from DSBs was calculated using the following formula: % Cleavage = 100 x (1 – (1-fraction cleaved)$^{1/2}$).

DNA isolation by Buffer K lysis

Genomic DNA from whole cells was lysed in Buffer K containing 0.1 mg/mL proteinase K (Roche, Switzerland), and 1X (v/v) GC buffer (Thermo Fisher Scientific, USA) in RNAse-/DNase-free water. Samples were incubated at 57 °C for 45 min, followed by heat inactivation at 95 °C for 15 min.

T7 endonuclease I (T7EI) assay

Genomic DNA was isolated 4 days after treatment, PCR-purified using QIAquick Gel Extraction Kit or QIAquick PCR Purification Kit (QIAGEN, Germany) and subjected to T7EI assay. A total of 100 ng purified PCR products from untreated control and treated samples were hybridized in a thermocycler (Biometra TOne, Analytik Jena, Germany) by heating at 95 °C for 5 min, followed by slow cooling from 95 °C to 25 °C to form heteroduplexes. Heteroduplex DNA was then treated with 1U of T7EI (NEB, USA) and incubated in the thermocycler at 37 °C for 15 min. The reaction was blocked by addition of 0.02 M EDTA and analyzed by 1% agarose gel following electrophoresis. Band intensities were subjected to densitometric analysis using ImageJ software (National Institutes of Health, USA).[4] Non-homologous end joining (NHEJ) frequencies were calculated using the following formula: % Cleavage = 100 x (1 – (1- fraction cleaved)$^{1/2}$).

Copy number variation (CNV) analysis of *NCF2* by ddPCR

CNV quantification was determined by ddPCR using QX200 Droplet Reader (Bio-Rad Laboratories, USA). Genomic DNA was digested with DraI enzyme (20 U/µL) (NEB, USA). 50-100 ng of digested gDNA was used per ddPCR reaction, primers, and probes (concentrations are stated in 'Materials and methods') targeting 3 locations upstream and 2 locations downstream of *NCF2* target site using primer *NCF2*_T1 to _T5 with reference to *FOXP2* housekeeping gene (Table S2). For droplet generation, 20 µL of sample reaction was transferred to a DG8 Cartridges for QX200/QX100 Droplet Generator (Bio-Rad Laboratories) and processed according to manufacturer's recommendation. Droplet generation and analysis were performed as described in the 'Materials and methods' section.

Characterization of identified integration sites by linear amplification mediated PCR (LAM-PCR)

The conditions for the first and second nested PCR were: initial denaturation for 10 min at 98 °C, then amplification with 35 cycles of denaturation (98 °C, 10 sec), annealing (62 °C, 10 sec) and extension (72 °C, 20 sec), with a final 5 min incubation at 72 °C. ISs were identified by Sanger sequencing of subcloned amplicons from the second nested PCR. The sequences were analysed for the presence of LTR III or ITR III primers, a restriction enzyme recognition site, and LC II primer. The unknown genome fragment was then subjected to standard nucleotide BLAST (blastn) using the National Center for Biotechnology Information (NCBI) webtool.[5] To calculate the distance of IS from TSS, the closest TSS was determined by identification of the first nucleotide of the 5' untranslated region (5' UTR) of the gene with the IS. To verify if the IS contained potential gRNA binding sites, a list of CRISPR off-targets was generated *in silico,* using CRISPRoff (v1.2beta) and Cas-OFFinder,[6,7] with a query criteria of up to 6 mismatches, and 2 DNA or RNA bulges around the IS (200bp upstream and downstream). The ISs were then manually analysed using the generated list of off targets, followed by a standard alignment using blastn.

Supplemental Tables

Table S1. Guide RNA target sequences. Underlining denotes the PAM sequence of the sgRNA or the crRNA target.

Target gene	Name	Cas nuclease	Guide RNA target sequence (5'-3'), PAM
Human *NCF2*	sgRNA #1	Cas9	AAGCACTCGGAGAGAAGTCTAGG
Human *NCF2*	sgRNA #2	Cas9	AGACTGCGCAACTACAGATTTGG
Human *NCF2*	sgRNA #3	Cas9	CTACAGATTTGGAAAGCACTCGG
Human *NCF2*	sgRNA #4	Cas9	AATCTTACAAACAAGTAATAGGG
Human *NCF2*	sgRNA #5	Cas9	GCAGTCTTCAACAAAAACTTTGG
Human *NCF2*	sgRNA #6	Cas9	AGTCTTCAACAAAAACTTTGGGG
Human *NCF2*	sgRNA #7	Cas9	CAGTCTTCAACAAAAACTTTGGG
Human *NCF2*	sgRNA #8	Cas9	AAATCTTACAAACAAGTAATAGG
Human *NCF2*	crRNA #1	Cas12a	TTTCCCCAAAGTTTTTGTTGAAGACTGC
Human *NCF2*	crRNA #2	Cas12a	TTTCCAAATCTGTAGTTGCGCAGTCTTC
Human *NCF2*	crRNA #3	Cas12a	TTTTTGTTGAAGACTGCGCAACTACAGA
Human *NCF2*	crRNA #4	Cas12a	TTTTGTTGAAGACTGCGCAACTACAGAT
Human *NCF2*	crRNA #5	Cas12a	TTTTCTTCAGCTTTGTAGTTTGTGAAAC
Human *NCF2*	crRNA #6	Cas12a	TTTGTAAGATTTAGCACCCTTCTGCTGT
Human *NCF2*	crRNA #7	Cas12a	TTTAGCACCCTTCTGCTGTATACTGTAC
Human *NCF2*	crRNA #8	Cas12a	TTTCACAAACTACAAAGCTGAAGAAAAT

Table S2. Primer and probe sequences for ddPCR. BHQ1, Black Hole Quencher®. Brackets denote fluorophore or quencher modifications.

Target gene	Primer	Sequence (5'-3')	Amplicon size (bp)
**2A-*NCF1***	Forward	TGACCTTCTCAAGTTGGCGG	122
	Reverse	TGTACACGTAGTGCTGGCTG	
	Probe	[6FAM]-CGGCGACACCTTCATCCGGCATATC-[BHQ1]	
ITR	Forward	TCCTCTAGAGTCGACCTGC	75
	Reverse	TGATTAACCCGCCATGCTAC	
	Probe	[6FAM]-ACGTAGCCATGCTCTAGAGCTTGC-[BHQ1]	
wPRE	Forward	TTCTGGGACTTTCGCT	118
	Reverse	CGACAACACCACGGAATTATC	

	Probe	[6FAM]-TCGCCACGGCAGAACTCATC-[BHQ1]	
FOXP2	Forward	TTGCTGGGTGTGTTTAATGC	139
	Reverse	AGGGTATAATGGATGGTGGC	
	Probe	[HEX]-CTGCCCGTGCCCTGGCCCAC-[BHQ1]	
NCF2_T1 (-1124 bp from Cut side)	Forward	GGCACCAGTGCCTTAATGTC	91
	Reverse	GTATGATCTCCAAAGCCCACCT	
	Probe	[6FAM]-ACTTCTGAACTGGCTGTGTGGTCA-[3IBFQ]	
NCF2_T2 (-794 bp from Cut side)	Forward	CTCACCTGTTTAATGGAGTTCAGTG	128
	Reverse	TACCTGCGCTTTGTTGGGAA	
	Probe	[6FAM]-ATTTAACCAATGGCATGTCCCTTT-[3IBFQ]	
NCF2_T3 (-111 bp from Cut side)	Forward	TGTGGAATAGCCAGACAGGG	128
	Reverse	TTTGGGGAAAATGCCCACCT	
	Probe	[6FAM]-TCAGTGAATGAAGAATGGCTGGAAGGG-[3IBFQ]	
NCF2_T4 (+231 bp from Cut side)	Forward	GCACAGTGGGTGAGGAGATG	99
	Reverse	CCTGGAAGACTCTCTCGTGC	
	Probe	[6FAM]-CCCTAACTCCTCCATTCACCTGTCC-[3IBFQ]	
NCF2_T5 (+682 bp from Cut side)	Forward	TGGACTACAACACTCAATTCCAC	151
	Reverse	ACTAGCTTCATGAACACCGCT	
	Probe	[6FAM]-ACCTTTGTACATTCTCAGAGAGGAACATGT-[3IBFQ]	
NCF2_T6 (+1266 bp from Cut side)	Forward	ATTTGGCTAACCCAATTGTCCA	126
	Reverse	ACGCTACAACAAGAAGTGGGT	
	Probe	[6FAM]-CCTGGCCTTAGTTCCTCTCCCT-[3IBFQ] **- Primer did not work**	

Table S3. Oligonucleotide sequences for TIDE and Sanger sequencing.

Primer	Target gene/purpose	Sequence (5’-3’)
**p67_F38773**	*NCF2*/TIDE forward primer	CCCCTATCACCACAGCACTT
**p67_R38975**	*NCF2*/TIDE reverse primer	CCTGGACTTGGGTGTCTTGT
**seqp67_R**	*NCF2*/Sanger sequencing	GTGCCCTTTCCAGACACTTC
**R8.91_6**	D64V/to check IDLV D64V packaging plasmid sequence	ACCTACCACCTGTAGTAGCA

Table S4. Antibodies and reagents for flow cytometry

Antibody/Reagent/Dye	Cat. No.	Manufacturer	Isotype	Clone
Rat anti-human/mouse CD11b V450	560456	BD Biosciences	Rat IgG2β,κ	M1/70
Rat anti-human CD11b PE-Cy7	101216	Biolegend	Rat IgG2β,κ	M1/70
Mouse anti-human CD15 VioBlue	130-113-488	Miltenyi Biotec	mouse IgM	VIMC6
REAfinity anti-human CD14 APC	130-110-520	Miltenyi Biotec	Human IgG1	REA599
Mouse anti-human $p47^{phox}$ APC	624076	BD Biosciences	Mouse IgG1	1/p47phox
Mouse anti-human $p67^{phox}$ AF488	sc-374510	Santa Cruz	Mouse IgG1,κ	D-6
Mouse anti-human CD34 PE-Cy7	A21691	Beckman Coulter	Mouse IgG1	581
Mouse anti-human CD38 BV605	562665	BD Biosciences	Mouse IgG1, κ	HB7
Mouse anti-human CD45RA BV786	563870	BD Biosciences	Mouse IgG2b, κ	HI100
Mouse anti-human CD133/1 PE	130-113-670	Miltenyi Biotec	Mouse IgG1, κ	AC133
Human anti-mouse c-Kit VioBright B515	130-111-618	Miltenyi Biotec	Human IgG1	REA791
Human anti-mouse Sca-1 PE	130-116-489	Miltenyi Biotec	Human IgG1	REA422
Lineage cell detection cocktail biotin	130-092-613	Miltenyi Biotec	N/A	N/A
LIVE/DEAD Fixable Yellow Dead cell stain kit	L34968	Thermo Fisher	N/A	N/A

LIVE/DEAD Fixable Near-IR Dead cell stain kit	L10119	Thermo Fisher	N/A	N/A
LIVE/DEAD Fixable Aqua Dead cell stain kit	L34957	Thermo Fisher	N/A	N/A
Rat IgG2β,κ isotype control V450	560457	BD Biosciences	Rat IgG2β,κ	A95-1
Rat IgG2β,κ isotype control PE-Cy7	400618	Biolegend	Rat IgG2β,κ	RTK4530
Mouse IgG1,κ isotype control APC	400120	Biolegend	Mouse IgG1,κ	MOPC-21
Mouse IgG1 isotype control AF488	sc-3890	Santa Cruz	Mouse IgG1	N/A
Human IgG1 isotype control VioBright B515	130-113-457	Miltenyi Biotec	Human IgG1	REA293
Human IgG1 isotype control PE	130-113-462	Miltenyi Biotec	Human IgG1	REA293
Human anti-biotin VioBlue	130-111-068	Miltenyi Biotec		REA746
FcR blocking reagent, human	130-059-901	Miltenyi Biotec	N/A	N/A
FcR blocking reagent, mouse	130-092-575	Miltenyi Biotec	N/A	N/A
Propidium iodide	P4170	Sigma-Aldrich	N/A	N/A

Table S5. Information of integration sites identified by LAM-PCR in chosen knocked-in clones. Clone #, clone name described in Figure S12C and S12D. VCN, vector copy number measured by ddPCR as described in 'Materials and methods'. IS chromosomal location, gene of IS, and IS location description are the location information of the IS determined by LAM-PCR based on the *Homo sapiens* genome assembly hg38 (GRCh38.p14). (-) indicates genomic orientation of a reverse strand. TSS, transcriptional start site predicted by Ensembl (https://www.ensembl.org)[8] that is closest to the IS. Identified ISs were checked on *in silico* CRISPR off-target sites of sgRNA *NCF2* using webtools, CRISPRoff (v1.2beta) and Cas-OFFinder.[7,9] Guide RNA binding sites with up to 6 mismatches and 2 DNA or RNA bulges around the IS (200bp upstream and downstream) are checked manually on standard nucleotide BLAST (blastn) using the National Center for Biotechnology Information (NCBI) webtool.[5] No potential CRISPR off-target sites were matched with the identified IS except on Clone 24 (Figure S11D), whereby a 11-nucleotide match of the sgRNA *NCF2* sequence + 1 DNA bulge including the protospacer adjacent motif (PAM) sequence was found 4 bp downstream of IS.

Clones derived from PLB-985 *NCF1* ΔGT cells treated with RNP+AAV *NCF1* at MOI 1e7 vg/cell (Figure S12C)

Clone # on Figure S11C	VCN 2A-*NCF1*	VCN ITR	IS chromosomal location	Gene of IS	IS location description	Predicted TSS at the gene	CRISPR-OFF / CasOFFinder / BLASTn alignment
**24**	1.19	0.67	Unidentified	-	-	-	-/-/-
**32**	3.19	1.53	11p13: 32874235	-	intergenic	NA	-/-/-

Clones derived from PLB-985 *NCF1* ΔGT cells treated with RNP+IDLV *NCF1* at MOI 6e3 LP/cell (Figure S12D)

Clone # on Figure S11D	VCN 2A-*NCF1*	VCN wPRE5	IS chromosomal location	Gene of IS	IS location description	Predicted TSS at the gene	CRISPR-OFF / CasOFFinder / BLASTn alignment
**10**	1.71	1.04	Unidentified	-	-	-	-/-/-
**11**	1.89	1.03	Unidentified	-	-	-	-/-/-
**14**	2.20	0.78	6p22.22: 6094020	HFE	intron 5 of HFE	26087439	-/-/-
			2p22.1: 40610981	-	intergenic	-	-/-/-
**15**	2.29	1.92	16: tandem repeats between 36340645-36421329	-	centromere	-	-/-/-
			1p32.2: 57536282	DAB1	intron 3 of DAB1	57291065	-/-/-
**16**	2.51	1.80	6p22.3: 18966464	-	intergenic	-	-/-/-
**17**	2.79	1.80	Unidentified	-	-	-	-/-/-

**18**	2.81	0.96	Unidentified	-	-	-	-/-/-
**19**	2.88	1.84	19p13.3: 1964394	CSNK1G2	intron 1 of CSNK1G2	1,941,172	-/-/-
			3q26.33: 181177379	SOX2-OT	intron 3 of SOX2-OT	181174890	-/-/-
**20**	2.89	1.15	11q12.1: 57960715	-	intergenic	-	-/-/-
			3q29: 196271658	PCYT1A	intron 1 of PCYT1A	196270541	-/-/-
**21**	2.98	2.63	Unidentified	-	-	-	-/-/-
**22**	3.01	2.58	8q24.21:129574663 (-)	CCDC26	Intron 1 of CCDC26	129574944	-/-/-
			6q13: 73299191	KHDC1	Intron 1 of KHDC1	73,310,215	-/-/-
			5p13.3-p13.2: 33562256 (-)	ADAMTS12	Intron 19 of ADAMTS12	33891941	-/-/-
**23**	3.62	1.91	1q32.2: 211096126 (-)	KCNH1	Intron 3 of KCNH1	211,103,515	-/-/-
			12p13.33: 1037807	ERC1	Intron 2 of ERC1	1,027,772	-/-/-
**24**	4.44	2.75	22q13.1: 37716096	TRIOBP	Intron 5 of TRIOBP	37,697,048	-/-/-
			12q24.33: 130627848 (-)	RIMBP2	Intron of RIMBP2	130633861	-/-/matched GGAGAGAANTCTAGG at 130627496-130627482 but 1 mismatch at seed sequence

Table S6. Primers used for LAM-PCR. Brackets indicate the specified modifications at the 5’. Underlined denotes TasI restriction enzyme recognition site.

Primer	Target gene/purpose	Sequence (5’-3’)
**LTR I_Biotin**	LTR/Linear PCR amplification	[Biotin]-GAACCCACTGCTTAAGCCTCA
**LTR II**	LTR/Nested PCR 1	GCTTGCCTTGAGTGCTTCA
**LTR III**	LTR/Nested PCR 2	AGTAGTGTGTGCCCGTCTGT
**ITR I_Biotin**	ITR/Linear PCR amplification	[Biotin]-ACCTCTGCCTGGTACCGAAT
**ITR II**	ITR/Nested PCR 1	GTAGCATGGCGGGTTAATCA
**ITR III**	ITR/Nested PCR 2	CAAAGGTCGCCCGACGC
**LC I**	Linker/Nested PCR 1	GACCCGGGAGATCTGAATTC
**LC II**	Linker/Nested PCR2	AGTGGCACAGCAGTTAGG
**Linker**	Linker cassette generation – top	GACCCGGGAGATCTGAATTCAGTGGCACAGCAGTTAGG
**Linker**	Linker cassette generation – bottom	[Phosphorylation]-AATTCCTAACTGCTGTGCCACTGAATTCAGATC

Table S7. Sequence of integration sites identified by LAM-PCR in chosen knocked-in clones. Clone #, clone name described in Figure S11C and S11D. (-) indicates genomic orientation of a reverse strand. Blue denotes the LTR III or ITR III primer sequence that binds to the LTR or ITR, respectively. Green denotes the linker LC II primer sequence. Underlined indicates the TasI restriction enzyme recognition site. Italicized indicates an unknown genomic sequence captured by LAM-PCR next to an LTR/ITR sequence.

Clone # on Figure S11C	Chromosomal location of IS	Sequence identified by subcloning after 2nd nested PCR
32	11p13	**GAACCCCTAGTGATGGAGTTG***GCCACACCCTTGGTTTGCTCTTCTAAACATGACTTTTCACTCTTTACATGGCCAGGCTGGACATTTTCCAAGTCTTTCTATTCTGCTTCCCTTTTAAATGATAAATGCCATCTTTAAGTCATGCGTGTCCACTCTA*AATT**CCTAACTGCTGTGCCACT**

Clone # on Figure S11D		Sequence
14	6p22.2	**AGTGGCACAGCAGTTAGG**AATT*ATGCATTTCTACCCCCTGAACATCTGTGGTGTAGGGAAAAGAGAATCAGAAAGAAGCCAGCTCATACAGAGTCCAAGGGTCTTTTGGGATATTGGGTTATGATCACTGGGGTGTCATTGAAGGATCCTAAGAAAGGAGGACCACGATCTCCCTTATATGGTGAATGTGTTGTTAAGAAGTTAGATGAGAGGTGAGGAGACCAG*TCAAGTCCCTGTTCGGGCGCCACTGCTAGAGATTTTCCACACTGACTAAAAGGGTCTGAGGGATCTCTAGTTACCAGAGTCACACA**ACAGACGGGCACACACTACT**
	2p22.1	**AGTAGTGTGTGCCCGTCTGT**TGTGTGACTCTGGTAACTAGAGATCCCTCAGACCCTTTTAGTCAGTGTGGAAAATCTCTAGCAGTGGCGCCCGAACAGGGACTTGAAAGCGAAAGGGAAACCAGAGG*CCTCTCCAGAAGCAGATTCTAGCACCATGCTGCTCGCACAGCCTTCAAAACTGTGAGTCAAATAAATCTCTTTTATTTATAAATTACTCAGTCTCAGATACTTTTACAGCAGTGCAAATGGATGAACACAGAAAATTAGTATCAAGGATCTCTCTCCTTCTAGCCTCCGCTAGTCAA*AATT**CCTAACTGCTGTGCCACT**
15	16 centromere	**AGTGGCACAGCAGTTAGG**AATT*TGCAAGTGGAGATTTCAGCCGCTTTGAGGTCAATGGTAGAAAAGGAAATATCTTCGTATAAAAACTAGACAGAATGATTCTCAGAAACTCCTTTGTGATGTGTGCGTTCAACTCACAGAGTTTAACCTTTCTTTTCACAGAGCAGTTAGGAAACACTCTGTTTGTGAAGCCTGCCAGTGGATATTC*CTTCGTTTCTCAGTAGCATCATTTAAATGATTAGCTCAGTGCTTTCAAGTCCCTGTTCGGGCGCCACTGCTAGAGATTTTCCACACTGACTAAAAGGGTCTGAGGGATCTCTAGTTACCAGAGTCACACA**ACAGACGGGCACACACTACT**
	1p32.2	**AGTGTGTGCCCGTCTGT**TGTGTGACTCTGGTAACTAGAGATCCCTCAGACCCTTTTAGTCAGTGTGGAAAATCTCTAGCAGTGGCGCCCGAACAGGGACTTGAAAGCGAAAGGG*GACAAACTTTGTGAGTCAGCTAGTGCCAAAAAAGCAGGACAGATTTCTGTAAAAGGATTTTGACAGATGGGATCATTCGAATCAGTGTTATTGTCTTAAAATGTGTCTGATGCACCAACAGTGATGCTTCTGAGGACCTTGACAAAATCTGAAGTTGA*AATT**CCTAACTGCTGTGCCACT**
16	6p22.3	**AGTGGCACAGCAGTTAGG**AATT*TGGGTAAAGCAGATTACCCTACATAATGTGGGTGGGCCTCATATAATCAGTTGAGAGCCTTAAGAGCAAAGATTGAGGTCCCCTGAGGAAGAT*CCTGTTCGGGCGCCACTGCTAGAGATTTTCCACACTGACTAAAAGGGTCTGAGGGATCTCTAGTTACCAGAGTCACACA**ACAGACGGGCACACACTACT**
19	19p13.3	**AGTAGTGTGTGCCCGTCTGT**TGTGTGACTCTGGTAACTAGAGATCCCTCAGACCCTTTTAGTCAGTGTGGAAAATCT*CCAGCAGTGACCTTGGCTAGATCGTCTGAATGCTGGCTGACACGTCTCAAGGAACACTTAATCCTTTTCACCTTGAACCTGGTTTTTTTTTGTTTTTTTTTTAACACA*

		*GGGTCTCACTCTGTCACCCAGGCAGGAGTGC*AGTAGCGCAATCTCGGCTCACTGCAACCTCGGCCTCCCAAGGTGCTGGGATTACAGGCATGAGCCACCGCTCCTGGCCTACAAAAATT**CCTAACTGCTGTGCCACT**
	3q26.33	**AGTGTGTGCCCGTCTGTTGT**GTGACTCTGGTAACTAGAGATCCCTC*GCTTACAAGATTGAGTAGATATGAAACTAGCCAGCAAAAGACAAAGCATTATTTCCCAGGTACT*AATT**CCTAACTGCTGTGCCACT**
20	11q12.1	**AGTGGCACAGCAGTTAGG**AATT*AAGTGCTGCACACCAGCAACACCGACATCGCTGGGTGCTTCTGACCGGGCGCAGTGGCTCACGCCTGTAATCCCAGCACTTTGGGAGGCTGAGGCGGGCGGATCACGAGGTCAGGAGGTCGAGACCATCCTGACTAACACGGTGAAACCCTGTCTCTACTAAAAATACAAAAACATTAGCTGGGCGTGGTGGTGGACGCCTGTAGTCCCAGCTACCTGGAAGGCTGAGGCAGGAGAATGGCGTGAACCCGGGAGGCGGAGCTTGTGGGTGAGCCAAGATGGCGCCAC*TGCTAGAGATTTTCCACACTGACTAAAAGGGTCTGAGGGATCTCTAGTTACCAGAGTCACACA**ACAGACGGGCACACACTACT**
	3q29	**AGTGGCACAGCAGTTAGG**AATT*AGCCAGGCGTGGTGGTGGGTGCCTGTAATCCCACCTACTCAGGAGGCTGAGGCAGGAGAATCGCTTGAACCTGGGAGGCGGAGGTTGTAGTGAGCCGAGATGGTGCCACTGCACTCTAGCCAGGGTGACGGAGTGAGACTCTTTCTCAAAAAAAAAAAAAAGGAAAGAAGGAGAAGGACATTTAAAAAATACATTATGCTGGTCCTAGCTATTCTGGAGGCTCAGATGGGAGGATTGCTTAAGCTCAGGAGTTTGAGGCTACAGTGTGCTATGATTATGGCTGTGAGAGCCGAGATCGTGCCACTGCACTCTAGCCAGGGTGACAGAGTGAGACTCTTTCTCAAAAAAAAAAAAAAGGAAAGAAGGAGAAGGACATTTAAAAAATACATTATGCTGGTCCTAGCTATTCTGGAGGCTCAGATGGGAGGATTGCTTAAGCTCAGGAGTTTGAGGCTACAGTGTGCTATGATTATGGCTGTGAGAGCCGAGATCGTGCCACTGCACTCTARCCAGGGTGACAGARTGAGACTCTTTCTCAAAAAAAAAAAAAAGGAAAGAAGGAGAAGGACATTTAAAAAATACATTATGCTGGTCCTARCTATTCTGGAGGSTCAGATGGGAGGATTGCTTAAGCTCAGGAGTTTGAGGCTACAGTGTGCTATGATTATGGCTGTGAGAGCCGAGATCGTGCC*CCTTTCGCTTTCAAGTCCCTGTTCGGGCGCCACTGCTAGAGATTTTCCACACTGACTAAAAGGGTCTGAGGGATCTCTAGTTACCAGAGTCACACA**ACAGACGGGCACACACTACT**
22	8q24.21 (-)	**AGTAGTGTGTGCCCGTCTGT**TGTGTGACTCTGGTAACTAGAGATCCCTCAGAC*AAATCATGCTGGGCTTTTTGGCCAGGTAAGGAGTTTGGTTTATAGTCTAAAAGAAATGGTGAACCATGTACAACATATTGTTTTAGAGTATATGTGCATTGTGGAATGAGTTAATCTAGCT*AATT**CCTAACTGCTGTGCCACT**
	6q13	**AGTGTGTGCCCGTCTGTTGT**GTGACTCTGGTAACTAGAGATCCCTCAGACCCTTTTAGKCAGTGTGGAAAATCTCTAGCAGTGGCGCCCGAACA*TGCTGGTCCAGCTGCGTGCTGTCGGTGGCATCTGGTGGCTCTAGTCTTGGAGGTTTCCGCGTGCTCCCATTTCTTGCCGTYTTTCTMAMTAACGCAGAGGGTCTCCCAATAGGTCTGACACCAGCCTCGTGGACCCTGAAAGATGAAGTCAGCCTCAAGTCGGC*AATT**CCTAACTGCTGTGCCACT**
	5p13.3-p13.2 (-)	**AGTGGCACAGCAGTTAGG**AATT*ATAAACAAACAAAAACACTTTGAGGGGAATGAGCTGTATGTGTGGCAAAGGCGACTGAGTTCTTCACCACCAGTGGA*CTGAGGGATCTCTAGTTACCAGAGTCACACA**ACAGACGGGCACACACTACT**
23	1q32.2 (-)	**AGTGGCACAGCAGTTAGG**AATT*AGCCGGGTGCGGTGGTGGGCACCTATAATCCCAGCTACTCAGGAGGCTGAGGCAGGAGAATCGCTTGAGCCTGGGAGGCGGAAGTTGCAGTGAGCTGAGATTG*CGCCACTGCTAGAGATTTTCCACACTGACTAAAAGGGTCTGAGGGATCTCTAGTTACCAGAGTCACACA**ACAGACGGGCACACACTACT**
	12p13.33	**AGTGGCACAGCAGTTAGG**AATT*TTGGCTGCAAGGAGGAATGGATTTTTGAAAAAAGCTATATCCGGCCGGGCGCAGTGGCTCACGTTTGTAAGCCCAGCACTTTGGGAGG*CCGAGTCCTGCGTCGAGAGAGCTCCTCTGGTTTCCCTTTCGCTTTCAAGTCCCTGTTCGGGCGCCACTGCTAGAGATTTTCCACACTGACTAAAAGGGTCTGAGGGATCTCTAGTTACCAGAGTCACACA**ACAGACGGGCACACACTACT**
24	22q13.1	**AGTGGCACAGCAGTTAGG**AATT*CCCTTCCCTTCCTTCTGCCCCTCTCATTTGGAGAGTATATGTCCTGCAGCCTTTTTTCTCCCGCAAACATACTTTCTCCTCCCCTTCTCTGGCAGGACTGGGACACTGTTGAGAGGCAGGAGGAGGAGGCCCCCAGCTGGGACGAGCTCGCAGTGATGATCCCGAGGAGGCCTCGGGAGGGGCCGAGAGCTGACAGCTCCCAAAGGGCTCCGTCTCTCCTCACCAGGTCCCCTGTGGGAGGAGATGCTGCAGGC*

		CAGAAAAAGGAGGGTGAGTCCTTCTGCCAGGTTGGTTCCCATGGTGATGGCCTGGGGCCCCCCAGATAGCCATCTCACTGGCCATTTGGGACT<br>*CTGGGCACGGCTTACTTTGGTGGCCTGAGTGTTAATAATAGTAACAGTTTGCATGTCATGATGGAGCAGCCACAATGAGTTA*TCTTTTATCTCTA<br>TCCTTTGATGCACACAATAGAGGGTTGCTACTGTATTATATAATGATCTAAGTTCTTCTGATCCTGTCTGAAGGGATGGTTGTAGCTGT<br>CCCAGTATTTGTCTACAGCCTTCTGATGTTTCTAACAGGCCAGGATTAACTGCGAATCGTTCTAGCTCCCTGCTTGCCCATACTATATGT<br>TTTAATTTATATTTTTTCTTTCCCCCTGGCCTTAACCGAATTTTTTCCCATCGCGATCTAATTCTCCCCCGCTTAATACTGACGCTCTCGC<br>ACCCATCTCTCTCCTTCTAGCCTCCGCTAGTCAAAATTTTTGGCGTACTCACCAGTCGCCGCCCCTCGCCTCTTGCCGTGCGCGCTTCAG<br>CAAGCCGAGTCCTGCGTCGAGAGAGCTCCTCTGGTTTCCCTTTCGCTTTCAAGTCCCTGTTTGGGCGCCACTGCTAGAGATTTTCCACA<br>CTGACTAAAAGGGTCTGAGGGATCTCTAGTTACCAGAGTCACACA**ACAGACGGGCACMCACTAC**
	12q24.33 (-)	**AGTGGCACAGCAGTTAGG***CCAGCCTAGAGTTCTCTCCTGAACCCTGAACCTGACCATCGAACTGTGGATTTTCCCGTGTATTTAGGTGTCAG*<br>*TCGGGTAACTCAAACTCAAAACTGGGCTTCGGCGCAAACCTGAATGTGAATCTCCCCACACTTCTACTGCTCTCTCAGTCTTTCTCATCCTAGGA*<br>*AATGGTCCTCCCCTTCCACCATGCAGCCCACGCCAAAAATATGAAGGCCATTCTTGTCACCCCCTTGCACTCCCTCTCAGCCCAGTCTATCACAA*<br>*TGGCTAGCAGGTCCTGCTGCCCCAATATATCCGGGACCTCTCCAATGACCCTCACGTCCACTACCATCCTGGTGCAGGCCACCCTTGCCAGTC*<br>*ATGTGGACTACATG*GTCGCCGCCCCTCGCCTCTTGCCGTGCGCGCTTCAGCAAGCCGAGTCCTGCGTCGAGAGAGCTCCTCTGGTTTCCC<br>TTTCGCTTTCAAGTCCCTGTTCGGGCGCCACTGCTAGAGATTTTCCACACTGACTAAAAGGGTCTGAGGGATCTCTAGTTACCAGAGTC<br>ACACA**ACAGACGGGCACACACTAC**

Table S8. Sequence of 2A-*NCF1* construct. Grey denotes *NCF2* homology arms. Orange denotes FMDV 2A. Blue denotes *NCF1* cDNA. Green denotes eGFP.

Donor template	Sequence (5’-3’)
NCF1-GFP	AGGCAGAGGTTGCAGTAAGCCCAGATTGTGCCACTGCACTCCAGCCTGGGTGACAGTGAGACCCTGTCAAGGTTGGGGGTGGGGAAAGGACAGAA<br>ATTCTATTTAAGGATTCGTTGTTCAATTTCTTTGACCCTTCTTCTCTATCTGGTAACTTTTTGAAAAACATAATTTATCCTTCTTCATTTTGCTCATTAT<br>CATGTTTAAGACAGATCAATAAGATGGTTAAACCCTGTGTTCACTCTCAAACCACTTTGCAATACTGTCTTTTCCCTGTTGATCACAATTAGGGGTGG<br>GGAAGGGTGACCGATAACAAATTCTGTGTGGAATAGCCAGACAGGGTAATCTTCCTACAGTGGTTTTAGAAATCCATGTGTACTTTTCCTTTTATCA<br>GTGAATGAAGAATGGCTGGAAGGGGAGTGCAAAGGGAAGGTGGGCATTTTCCCCAAAGTTTTTGTTGAAGACTGCGCAACTACAGATTTGGAAAG<br>CACTCGGAGAGAAGTCTCTAGACACAAACAGAAAATTGTGGCACCGGTGAAACAGACTTTGAATTTTGACCTTCTCAAGTTGGCGGGAGACGTCGA<br>GTCCAACCCTGGGCCCGGCGACACCTTCATCCGGCATATCGCCCTGCTGGGCTTCGAGAAGCGGTTCGTGCCCAGCCAGCACTACGTGTACATGTTT<br>CTGGTGAAGTGGCAGGACTTGAGCGAGAAGGTGGTGTACCGGCGGTTCACCGAGATCTACGAGTTCCACAAGACCCTGAAAGAGATGTTCCCCATC<br>GAGGCCGGAGCCATCAACCCCGAGAACCGGATCATCCCCCACCTGCCTGCCCCCAAGTGGTTCGACGGCCAGAGAGCCGCCGAGAACAGGCAGGG<br>CACCCTGACCGAGTACTGCAGCACCCTGATGAGCCTGCCCACCAAGATCAGCCGGTGCCCTCATCTGCTGGATTTCTTCAAAGTGCGGCCCGACGAC<br>CTGAAGCTGCCCACCGACAACCAGACCAAGAAGCCCGAGACCTATCTGATGCCCAAGGACGGCAAGAGCACCGCCACCGACATCACCGGCCCCAT<br>CATCCTGCAGACCTATCGGGCAATCGCCAACTACGAGAAAACCAGCGGCAGCGAGATGGCCCTGAGCACCGGCGACGTGGTGGAGGTGGTGGAAA<br>AGAGCGAGAGCGGGTGGTGGTTCTGCCAGATGAAGGCCAAGCGGGGCTGGATTCCCGCCAGCTTCCTGGAACCCCTGGACAGCCCCGACGAGACC<br>GAGGACCCCGAGCCCAACTACGCCGGCGAGCCCTATGTGGCCATCAAGGCCTACACCGCCGTGGAGGGCGACGAGGTGTCCCTGCTGGAAGGCGA<br>GGCCGTGGAGGTGATCCACAAGCTGCTGGACGGTTGGTGGGTGATCCGGAAGGACGACGTGACCGGCTACTTCCCCAGCATGTACTTGCAGAAGAG

	CGGCCAGGACGTCAGCCAGGCCCAGCGGCAGATCAAGAGAGGCGCCCCTCCCAGGCGGAGCAGCATCCGGAACGCCCACAGCATCCACCAGAGAA<br>GCCGGAAGAGACTGAGCCAGGACGCCTATCGGCGGAACAGCGTGCGGTTCTTGCAGCAGCGGCGGAGGCAGGCCAGACCCGGCCCTCAGAGCCCC<br>GGCAGCCCATTGGAAGAGGAACGGCAGACCCAGCGGAGCAAGCCCCAGCCCGCCGTGCCCCCCCGACCCAGCGCTGATCTGATCCTGAACCGGTG<br>CAGCGAGAGCACCAAGCGGAAGCTGGCCAGCGCCGTGCTGTGCGTCTTCAGACACAAGCAAAAGATCGTAGCCCCTGTTAAGCAAACCTTAAACTT<br>CGATCTCCTTAAATTAGCAGGGGATGTTGAGTCCAACCCTGGGCCCCACACACGCATGGTCTCAAAAGGAGAAGAACTGTTCACAGGCGTTGTACC<br>GATCCTGGTGGAGCTGGACGGCGACGTGAACGGCCACAAGTTCAGCGTGAGCGGCGAGGGCGAGGGCGACGCCACCTACGGCAAGCTGACCCTGA<br>AGTTCATCTGCACCACCGGCAAGCTGCCCGTGCCCTGGCCCACCCTGGTGACCACCCTGACGTATGGAGTACAATGCTTCAGCAGATACCCCGACCA<br>CATGAAGCAGCACGACTTCTTCAAGAGCGCCATGCCCGAGGGCTACGTGCAGGAGAGAACCATCTTCTTCAAGGACGACGGCAATTACAAGACCA<br>GAGCCGAGGTGAAGTTCGAGGGCGACACCCTGGTGAACAGAATCGAGCTGAAGGGCATCGACTTCAAGGAGGACGGCAACATCCTGGGCCACAAG<br>CTGGAGTACAACTACAACAGCCACAACGTGTACATCATGGCCGACAAGCAGAAGAACGGCATCAAAGTGAATTTTAAAATCAGACACAACATCGA<br>GGACGGCAGCGTGCAGCTGGCCGACCACTACCAGCAGAACACCCCCATCGGCGACGGCCCCGTGCTGCTGCCCGACAACCACTACCTGAGCACCCA<br>ATCAGCGTTAAGCAAGGACCCCAACGAGAAGAGAGACCACATGGTGCTGCTGGAGTTTGTGACCGCCGCCGGCATCACCCTTGGCATGGACGAATT<br>GTACAAACCGAAGAAGAAGAGAAAAGTTCTGTAGGTCTTCGCTAGCGATGTTTCACAAACTACAAAGCTGAAGAAAATGAAGCCCTATTACTTGTT<br>TGTAAGATTTAGCACCCTTCTGCTGTATACTGTACTGAGACATTACAGTTTGGAAGTGTTAACTATTTATTCCCTGTTAAAATTTAACCTACTAGACA<br>ATGATGTGAGTACCCAGGATGATTTCCTGGGGCACAGTGGGTGAGGAGATGGGGACAGGTGAATGGAGGAGTTAGGGGAGAGGAAAAGTGGATG<br>GAAGTGTCTGGAAAGGGCACGAGAGAGTCTTCCAGGTACTGATCCTGTTTCTTGCTCTGAGTGCTAGCTAGCCAGCTGTGTTCACACTGTAAACATT<br>CATCAAGCTGTACATTTGGTGCACTTTTCTGTGTCATACCACAATAAAAAAAAACCTAGCATCTTACAAAAACAAGACACCCAAGTCCAGGCCCAA<br>GGAGTAAGTACAAATATTCCTGTTTCTGAACCATTACTGTAATTGGCTCTTAAGGCTTGAAGTAAC
GFP	AGGCAGAGGTTGCAGTAAGCCCAGATTGTGCCACTGCACTCCAGCCTGGGTGACAGTGAGACCCTGTCAAGGTTGGGGGTGGGGAAAGGACAGAA<br>ATTCTATTTAAGGATTCGTTGTTCAATTTCTTTGACCCTTCTTCTCTATCTGGTAACTTTTTGAAAAACATAATTTATCCTTCTTCATTTTGCTCATTAT<br>CATGTTTAAGACAGATCAATAAGATGGTTAAACCCTGTGTTCACTCTCAAACCACTTTGCAATACTGTCTTTTCCCTGTTGATCACAATTAGGGGTGG<br>GGAAGGGTGACCGATAACAAATTCTGTGTGGAATAGCCAGACAGGGTAATCTTCCTACAGTGGTTTTAGAAATCCATGTGTACTTTTCCTTTTATCA<br>GTGAATGAAGAATGGCTGGAAGGGGAGTGCAAAGGGAAGGTGGGCATTTTCCCCAAAGTTTTTGTTGAAGACTGCGCAACTACAGATTTGGAAAG<br>CACTCGGAGAGAAGTCTCTAGAAGACACAAGCAAAAGATCGTAGCCCCTGTTAAGCAAACCTTAAACTTCGATCTCCTTAAATTAGCAGGGGATGT<br>TGAGTCCAACCCTGGGCCCCACACACGCATGGTCTCAAAAGGAGAAGAACTGTTCACAGGCGTTGTACCGATCCTGGTGGAGCTGGACGGCGACGT<br>GAACGGCCACAAGTTCAGCGTGAGCGGCGAGGGCGAGGGCGACGCCACCTACGGCAAGCTGACCCTGAAGTTCATCTGCACCACCGGCAAGCTGC<br>CCGTGCCCTGGCCCACCCTGGTGACCACCCTGACGTATGGAGTACAATGCTTCAGCAGATACCCCGACCACATGAAGCAGCACGACTTCTTCAAGA<br>GCGCCATGCCCGAGGGCTACGTGCAGGAGAGAACCATCTTCTTCAAGGACGACGGCAATTACAAGACCAGAGCCGAGGTGAAGTTCGAGGGCGAC<br>ACCCTGGTGAACAGAATCGAGCTGAAGGGCATCGACTTCAAGGAGGACGGCAACATCCTGGGCCACAAGCTGGAGTACAACTACAACAGCCACAA<br>CGTGTACATCATGGCCGACAAGCAGAAGAACGGCATCAAAGTGAATTTTAAAATCAGACACAACATCGAGGACGGCAGCGTGCAGCTGGCCGACC<br>ACTACCAGCAGAACACCCCCATCGGCGACGGCCCCGTGCTGCTGCCCGACAACCACTACCTGAGCACCCAATCAGCGTTAAGCAAGGACCCCAACG<br>AGAAGAGAGACCACATGGTGCTGCTGGAGTTTGTGACCGCCGCCGGCATCACCCTTGGCATGGACGAATTGTACAAACCGAAGAAGAAGAGAAAA<br>GTTCTGTAGGTCTTCGCTAGCGATGTTTCACAAACTACAAAGCTGAAGAAAATGAAGCCCTATTACTTGTTTGTAAGATTTAGCACCCTTCTGCTGTA<br>TACTGTACTGAGACATTACAGTTTGGAAGTGTTAACTATTTATTCCCTGTTAAAATTTAACCTACTAGACAATGATGTGAGTACCCAGGATGATTTCC<br>TGGGGCACAGTGGGTGAGGAGATGGGGACAGGTGAATGGAGGAGTTAGGGGAGAGGAAAAGTGGATGGAAGTGTCTGGAAAGGGCACGAGAGAG<br>TCTTCCAGGTACTGATCCTGTTTCTTGCTCTGAGTGCTAGCTAGCCAGCTGTGTTCACACTGTAAACATTCATCAAGCTGTACATTTGGTGCACTTTTC

	TGTGTCATACCACAATAAAAAAAAACCTAGCATCTTACAAAAACAAGACACCCAAGTCCAGGCCCAAGGAGTAAGTACAAATATTCCTGTTTCTGA<br>ACCATTACTGTAATTGGCTCTTAAGGCTTGAAGTAAC
NCF1	AGGCAGAGGTTGCAGTAAGCCCAGATTGTGCCACTGCACTCCAGCCTGGGTGACAGTGAGACCCTGTCAAGGTTGGGGGTGGGGAAAGGACAGAA<br>ATTCTATTTAAGGATTCGTTGTTCAATTTCTTTGACCCTTCTTCTCTATCTGGTAACTTTTTGAAAAACATAATTTATCCTTCTTCATTTTGCTCATTAT<br>CATGTTTAAGACAGATCAATAAGATGGTTAAACCCTGTGTTCACTCTCAAACCACTTTGCAATACTGTCTTTTCCCTGTTGATCACAATTAGGGGTGG<br>GGAAGGGTGACCGATAACAAATTCTGTGTGGAATAGCCAGACAGGGTAATCTTCCTACAGTGGTTTTAGAAATCCATGTGTACTTTTCCTTTTATCA<br>GTGAATGAAGAATGGCTGGAAGGGGAGTGCAAAGGGAAGGTGGGCATTTTCCCCAAAGTTTTTGTTGAAGACTGCGCAACTACAGATTTGGAAAG<br>CACTCGGAGAGAAGTCTCTAGACACAAACAGAAAATTGTGGCACCGGTGAAACAGACTTTGAATTTTGACCTTCTCAAGTTGGCGGGAGACGTCGA<br>GTCCAACCCTGGGCCCGGCGACACCTTCATCCGGCATATCGCCCTGCTGGGCTTCGAGAAGCGGTTCGTGCCCAGCCAGCACTACGTGTACATGTTT<br>CTGGTGAAGTGGCAGGACTTGAGCGAGAAGGTGGTGTACCGGCGGTTCACCGAGATCTACGAGTTCCACAAGACCCTGAAAGAGATGTTCCCCATC<br>GAGGCCGGAGCCATCAACCCCGAGAACCGGATCATCCCCCACCTGCCTGCCCCCAAGTGGTTCGACGGCCAGAGAGCCGCCGAGAACAGGCAGGG<br>CACCCTGACCGAGTACTGCAGCACCCTGATGAGCCTGCCCACCAAGATCAGCCGGTGCCCTCATCTGCTGGATTTCTTCAAAGTGCGGCCCGACGAC<br>CTGAAGCTGCCCACCGACAACCAGACCAAGAAGCCCGAGACCTATCTGATGCCCAAGGACGGCAAGAGCACCGCCACCGACATCACCGGCCCCAT<br>CATCCTGCAGACCTATCGGGCAATCGCCAACTACGAGAAAACCAGCGGCAGCGAGATGGCCCTGAGCACCGGCGACGTGGTGGAGGTGGTGGAAA<br>AGAGCGAGAGCGGGTGGTGGTTCTGCCAGATGAAGGCCAAGCGGGGCTGGATTCCCGCCAGCTTCCTGGAACCCCTGGACAGCCCCGACGAGACC<br>GAGGACCCCGAGCCCAACTACGCCGGCGAGCCCTATGTGGCCATCAAGGCCTACACCGCCGTGGAGGGCGACGAGGTGTCCCTGCTGGAAGGCGA<br>GGCCGTGGAGGTGATCCACAAGCTGCTGGACGGTTGGTGGGTGATCCGGAAGGACGACGTGACCGGCTACTTCCCCAGCATGTACTTGCAGAAGAG<br>CGGCCAGGACGTCAGCCAGGCCCAGCGGCAGATCAAGAGAGGCGCCCCTCCCAGGCGGAGCAGCATCCGGAACGCCCACAGCATCCACCAGAGAA<br>GCCGGAAGAGACTGAGCCAGGACGCCTATCGGCGGAACAGCGTGCGGTTCTTGCAGCAGCGGCGGAGGCAGGCCAGACCCGGCCCTCAGAGCCCC<br>GGCAGCCCATTGGAAGAGGAACGGCAGACCCAGCGGAGCAAGCCCCAGCCCGCCGTGCCCCCCCGACCCAGCGCTGATCTGATCCTGAACCGGTG<br>CAGCGAGAGCACCAAGCGGAAGCTGGCCAGCGCCGTGTAGGTCTTCGCTAGCGATGTTTCACAAACTACAAAGCTGAAGAAAATGAAGCCCTATTA<br>CTTGTTTGTAAGATTTAGCACCCTTCTGCTGTATACTGTACTGAGACATTACAGTTTGGAAGTGTTAACTATTTATTCCCTGTTAAAATTTAACCTACT<br>AGACAATGATGTGAGTACCCAGGATGATTTCCTGGGGCACAGTGGGTGAGGAGATGGGGACAGGTGAATGGAGGAGTTAGGGGAGAGGAAAAGT<br>GGATGGAAGTGTCTGGAAAGGGCACGAGAGAGTCTTCCAGGTACTGATCCTGTTTCTTGCTCTGAGTGCTAGCTAGCCAGCTGTGTTCACACTGTAA<br>ACATTCATCAAGCTGTACATTTGGTGCACTTTTCTGTGTCATACCACAATAAAAAAAAACCTAGCATCTTACAAAAACAAGACACCCAAGTCCAGGC<br>CCAAGGAGTAAGTACAAATATTCCTGTTTCTGAACCATTACTGTAATTGGCTCTTAAGGCTTGAAGTAAC

Table S9. Primers used for NGS verification of CHANGE-seq off-target sites.

Site	Genomic Coordinate	Site sequence	Forward primer	Reverse primer	Annealing temperature (°C)
ON	chr1:183556116-183556139	AAGCACTCGGAGAGAAGTCTAGG	CTTTCCCTACACGACGCTCTTCCGATCTACAGTATACAGCAGAAGGGTGC	GGAGTTCAGACGTGTGCTCTTCCGATCTTGTGGAATAGCCAGACAGGG	62

OT1	chr16:2169909-2169932	AAGCACCAGGAGAGAAGCCTTGG	CTTTCCCTACACGACGCTCTTCCGATCTTGAGCCACCGACAAGGGA	GGAGTTCAGACGTGTGCTCTTCCGATCTACTGGTTTGCCTCTGTGCTC	59<br>(less efficient PCR)
OT2	chr5:107756848-107756872	TAGAACTCGGAGAGGAGGTCTAGG	CTTTCCCTACACGACGCTCTTCCGATCTCCTAGTCTTAGGTAGTCTGTTATAGC	GGAGTTCAGACGTGTGCTCTTCCGATCTATGTTCCCTGGGTAAATGGC	62
OT3	chr21:25516315-25516338	CAGCTCTCGGAGAGAAGTCTGGG	CTTTCCCTACACGACGCTCTTCCGATCTGTCCCCTTTGCCAAC	GGAGTTCAGACGTGTGCTCTTCCGATCTTGATTTAAAAATTATCTTGAGACAG	57
OT4	chr6:15827983-15828005	AGGCACCCAGAGGAAGTCTTGG	CTTTCCCTACACGACGCTCTTCCGATCTCTCTCAGTGAGGCTGACCAC	GGAGTTCAGACGTGTGCTCTTCCGATCTCTGTGACATGAAGCCCCCAG	68
	chr22:46578343-46578367	CAGCACTCGAGAGAGGAGTCTGGG	CTTTCCCTACACGACGCTCTTCCGATCTACATGCCAAAAAGGAGGTGC	GGAGTTCAGACGTGTGCTCTTCCGATCTCATTTTGAACCCCAACCCAGG	66<br>(PCR not working)

Table S10. Raw data of NGS deep sequencing for verification of CHANGE-seq off-target sites.

			On-target			Off-target 1			Off-target 2			Off-target 3			Off-target 4		
	**Cell type**	**Treatment**	**Aligned reads**	**Reads with indel**	**Indel%**	**Aligned reads**	**Reads with indel**	**Indel%**	**Aligned reads**	**Reads with indel**	**Indel%**	**Aligned reads**	**Reads with indel**	**Indel%**	**Aligned reads**	**Reads with indel**	**Indel%**
1	HD hCD34+	Cas9-sgRNA NCF2	3973	658	16.56%	17910	47	0.26%	3182	0	0.00%	na	na	na	1738	0	0.00%
2.1	HD hCD34+	Cas9-sgRNA NCF2 + AAV 2A-NCF1	4215	150	3.56%	na	na	na	2551	0	0.00%	2943	43	1.46%	3125	1	0.03%
2.2	HD hCD34+	Cas9-sgRNA NCF2 + AAV 2A-NCF1	6511	253	3.89%	1227	0	0.00%	3254	0	0.00%	6419	132	2.06%	3707	0	0.00%
4	HD hCD34+	Unedited	4421	37	0.84%	1884	0	0.00%	2278	2	0.09%	3960	0	0.00%	1200	0	0.00%
5	HD hCD34+	Unedited	10008	32	0.32%	105	0	0.00%	3419	0	0.00%	8376	0	0.00%	3756	0	0.00%
6	HD hCD34+	Unedited	12600	16	0.13%	1796	0	0.00%	3430	0	0.00%	7051	0	0.00%	3825	0	0.00%
7	HD hCD34+	Cas9-sgRNA NCF2	7513	1078	14.35%	243	0	0.00%	3090	0	0.00%	5661	43	0.76%	2703	0	0.00%
8	HD hCD34+	Unedited	6628	7	0.11%	381	0	0.00%	3502	0	0.00%	5822	0	0.00%	3246	0	0.00%
9	HD hCD34+	Cas9-sgRNA NCF2	5871	1493	25.43%	414	0	0.00%	2957	1	0.03%	5118	51	1.00%	3483	0	0.00%
10	HD hCD34+	Unedited	6494	29	0.45%	1365	3	0.22%	3750	1	0.03%	6857	1	0.01%	2987	0	0.00%
11	HD hCD34+	Cas9-sgRNA NCF2	8392	2018	24.05%	1967	0	0.00%	4065	1	0.02%	1280	8	0.63%	5692	0	0.00%
12.1	p47-CGD hCD34+	AAV 2A-NCF1	1645	31	1.88%	669	2	0.30%	1364	0	0.00%	470	0	0.00%	2492	0	0.00%
12.2	p47-CGD hCD34+	AAV 2A-NCF1	10399	58	0.56%	na	na	na	3193	0	0.00%	635	1	0.16%	3350	0	0.00%
13.1	p47-CGD hCD34+	Cas9-sgRNA NCF2	7194	2738	38.06%	na	na	na	2158	0	0.00%	4415	52	1.18%	5104	1	0.02%
13.2	p47-CGD hCD34+	Cas9-sgRNA NCF2	7943	3209	40.40%	1868	1	0.05%	2438	1	0.04%	6081	97	1.60%	5110	0	0.00%
14.1	p47-CGD hCD34+	Cas9-sgRNA NCF2 + AAV 2A-NCF1	4511	520	11.53%	2106	0	0.00%	1953	0	0.00%	627	2	0.32%	3831	0	0.00%
14.2	p47-CGD hCD34+	Cas9-sgRNA NCF2 + AAV 2A-NCF1	8677	402	4.63%	na	na	na	988	0	0.00%	582	0	0.00%	148	0	0.00%
15	PLB-985 *NCF1* ΔGT	Unedited	7046	16	0.23%	na	na	na	2470	0	0.00%	5423	0	0.00%	2748	0	0.00%
16	PLB-985 *NCF1* ΔGT	Unedited	5450	19	0.35%	2002	0	0.00%	1340	0	0.00%	2017	0	0.00%	835	0	0.00%
17.1	PLB-985 *NCF1* ΔGT	Cas9-sgRNA NCF2	6447	3790	58.79%	425	0	0.00%	2553	8	0.31%	4924	403	8.18%	3109	0	0.00%
17.2	PLB-985 *NCF1* ΔGT	Cas9-sgRNA NCF2	5916	3447	58.27%	1758	1	0.06%	2521	7	0.28%	3785	347	9.17%	4518	0	0.00%
18	PLB-985 *NCF1* ΔGT	Cas9-sgRNA NCF2 + AAV 2A-NCF1	3818	1728	45.26%	724	1	0.14%	2235	9	0.40%	5959	631	10.59%	3833	1	0.03%
19	PLB-985 *NCF1* ΔGT	Cas9-sgRNA NCF2 + AAV 2A-NCF1	6351	2225	35.03%	2649	3	0.11%	1700	0	0.00%	3464	336	9.70%	2015	0	0.00%
20	PLB-985 *NCF1* ΔGT	Cas9-sgRNA NCF2 + IDLV 2A-NCF1	6240	2912	46.67%	930	9	0.97%	1599	18	1.13%	5656	537	9.49%	2383	0	0.00%
21	PLB-985 *NCF1* ΔGT	Cas9-sgRNA NCF2 + IDLV 2A-NCF1	2971	1224	41.20%	275	1	0.36%	766	1	0.13%	1740	95	5.46%	1447	0	0.00%
22	HEK293	Control untreated	17910	47	0.26%	1921	0	0.00%	2124	0	0.00%	3717	0	0.00%	3616	0	0.00%

Table S11. Raw data of CHANGE-seq results.

#Chromosome	Genomic Coordinate	Nuclease_Read_Count	Strand	Site_Sequence	Site_Substitution_Number	Site_Sequence_Gaps_Allowed	Realigned_Target_Sequence
chr1	chr1:183556116-183556139	1990	-	AAGCACTCGGAGAGAAGTCTAGG	0		none
chr16	chr16:2169909-2169932	372	+	AAGCACCAGGAGAGAAGCCTTGG	3		none
chr5	chr5:107756848-107756872	122	+			TAGAACTCGGAGAGGAGGTCTAGG	AAGCACTCGGAGAGAAG-TCTNGG
chr22	chr22:46578343-46578367	58	+			CAGCACTCGAGAGAGGAGTCTGGG	AAGCACTCG-GAGAGAAGTCTNGG
chr21	chr21:25516315-25516338	54	+	CAGCTCTCGGAGAGAAGTCTGGG	2		none
chr6	chr6:15827983-15828005	48	+			AGGCACCCAGAG-GAAGTCTTGG	AAGCACTCGGAGAGAAGTCTNGG
chr13	chr13:42807847-42807871	42	-			TAGCACTCAGTAGAGAAGTCTGGG	AAGCACTCGG-AGAGAAGTCTNGG
chr12	chr12:40598403-40598426	30	+	AAACACTGGGAGAGGAGTTTGGG	4		none
chr22	chr22:49208293-49208315	30	-			AGGCACCCAGAGA-AAGTCTTGG	AAGCACTCGGAGAGAAGTCTNGG
chr3	chr3:136501538-136501561	30	+	AGACACTAGGAGAGAAGTCTGGA	4		none
chr16	chr16:77829361-77829384	24	-	TGAAACTCGGACAGAAGTCTTGG	5	ATGAAACTCGGACAGAAGTCTTGG	AAGCA-CTCGGAGAGAAGTCTNGG
chr11	chr11:57478391-57478414	22	+	GAGCACTTGGAGAGAAGTTTGGT	4	GAGCACTTGGAGAGAAGT-TTGG	AAGCACTCGGAGAGAAGTCTNGG
chr1	chr1:204462322-204462345	16	-	CAGCCCACGGAGAGAAGTCCTAG	5	CAGCCCACGGAGAGAAGTCCTAGG	AAGCACTCGGAGAGAAGTC-TNGG
chr17	chr17:4531821-4531844	14	+	TCCCACCCGGAGAGAAGCCTGGG	5		none
chr5	chr5:152113531-152113554	14	+	CAGCACCCAGAGAGAAGCCTGGG	4		none
chr14	chr14:98954426-98954449	10	-	AGCCACCCGGAGAGAAGTTAGGC	6	AGCCACCCGGAGAGAAGT-TAGG	AAGCACTCGGAGAGAAGTCTNGG
chr18	chr18:8290133-8290156	10	+	AAGCACCAGAAGAGAAGTCTGAG	4		none
chr10	chr10:18783649-18783671	8	-			AAGCCCTAGAAGAGAAG-CTAGG	AAGCACTCGGAGAGAAGTCTNGG
chr10	chr10:70782490-70782512	8	+			ATGCACTCAG-GAGTAGTCTTGG	AAGCACTCGGAGAGAAGTCTNGG
chr14	chr14:91292959-91292982	8	-	AGGCACCCAGAGAGAAGGCTGGA	5		none
chr15	chr15:96449784-96449807	8	-	AACCACTCGAAGAGGAAGCCTGG	6	AACCACTCGAAGAGGAAGCCTGGG	AAGCACTCGGAGAG-AAGTCTNGG
chr2	chr2:71891430-71891453	8	+	CAGCACCCGCAGAGAAGCCCTGG	5		none
chr7	chr7:103010789-103010812	8	+	AAGCACACAGACAGAAGATTAGG	5		none
chr16	chr16:54141943-54141966	6	-	TAGCCCCAGGAGAGAAGTCTTCT	6		none
chr1	chr1:160349626-160349649	6	-	GTACACTAGGAGAGAAGCCTGAG	6		none
chr22	chr22:17993655-17993678	6	-	AAGCACTGGGAGGAGAGTGTGGG	5		none
chr22	chr22:41482320-41482342	6	+			TAGCACAGGGAGA-AAGTCTCGG	AAGCACTCGGAGAGAAGTCTNGG
chr2	chr2:5938617-5938640	6	-	AAAGCCTGGGAGAGAAGCCTGGG	5	AAGC-CTGGGAGAGAAGCCTGGG	AAGCACTCGGAGAGAAGTCTNGG
chr3	chr3:52678520-52678543	6	+	AAGAGCTCACAGAGAAGTCTGCC	6		none
chr7	chr7:159212022-159212044	6	-			TAGC-CTTGGAGAGAAGTCAGGG	AAGCACTCGGAGAGAAGTCTNGG
chr8	chr8:32514105-32514128	6	-	AAGAATTTGCAGACAAGTCTTGG	5		none
chr8	chr8:6648645-6648668	6	+	TAGCACTCAGGCAGAAGTCTTCT	6		none
chr9	chr9:136908165-136908188	6	+	GAGTGCCCGGAGAGAAGCCTGAG	6		none
chrX	chrX:95644230-95644253	6	+	AGGCACTGGGAAAAAAGTCCTGT	6		none
chr12	chr12:132714738-132714761	4	-	AAGAGCATGGAGAGAAGACAAGG	6		none
chr12	chr12:4535130-4535153	4	+	GAGCACTGAGAGAGAAGTTAGGA	6	GAGCACTGAGAGAGAAGT-TAGG	AAGCACTCGGAGAGAAGTCTNGG
chr12	chr12:57782849-57782872	4	+	AGCTACCCGGTGAGAAGTCCTGG	6		none
chr13	chr13:40563272-40563295	4	+	ACAAACTGGGAGAGAAGTCTTCT	6		none
chr17	chr17:42964460-42964483	4	+	GCGCACGCGCAGAGAAGCCTGGG	5		none
chr1	chr1:161074585-161074608	4	-	AAGCACTGGAGCAGAAGTCCTTG	6		none
chr20	chr20:61582528-61582551	4	-	AAGCACTGGGAGGGAAGTCTACA	4		none
chr2	chr2:126915783-126915805	4	-			ACGCACTAGGGG-GAAGTCTAGG	AAGCACTCGGAGAGAAGTCTNGG
chr2	chr2:138915425-138915447	4	-			AAGCCCTCGGACAGA-GTCTTGG	AAGCACTCGGAGAGAAGTCTNGG
chr2	chr2:234373478-234373501	4	+	AAGCCCCCGGAGAGAAGCCTGAG	4		none
chr4	chr4:105006167-105006190	4	-	ATGCACACGGAGTGAAGTCTTAG	4		none
chr4	chr4:636894-636917	4	+	AACCCCCCGGAGGGAAGTCTCAG	5		none
chr5	chr5:41354218-41354241	4	-	TAGCATCCAGAGAGAAGTCCGGG	5		none
chr6	chr6:110553723-110553746	4	-	CTGCACACGGAGAGAACTCTGGG	4		none

chr6	chr6:163632363-163632386	4	-	AAGTGCTCGTAAAGAAGGCTTGG	5		none
chr7	chr7:74306877-74306899	4	+			AGCCACTCGGAGA-AAGTCTGGG	AAGCACTCGGAGAGAAGTCTNGG
chr9	chr9:109483005-109483028	4	+	ATGCACAGGGAGAAAAGTCTTGA	5		none
chr9	chr9:129829378-129829401	4	-	AGCCCCTCGGAGAGCAGTCTTGC	5	AAGCCCCTCGGAGAGCAGTCTTGC	AAGCAC-TCGGAGAGAAGTCTNGG
chr9	chr9:88141639-88141662	4	+	AAGCCCTGGGAGAGAAGTCTGGT	3		none

Supplemental Figures

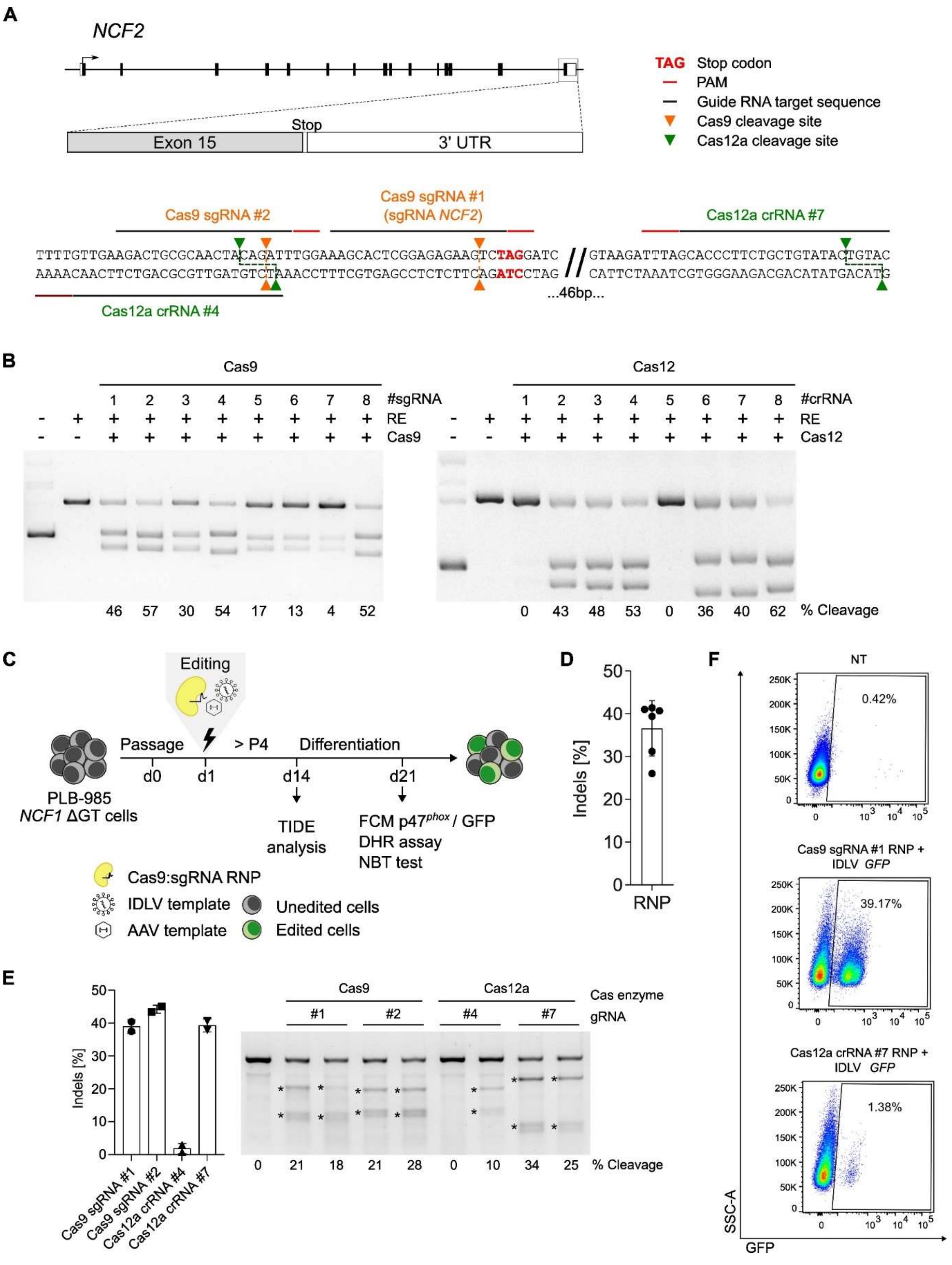

Figure S1. CRISPR-Cas gRNA design and screening *in vitro* and in cells.

(**A**) Position of Cas9 and Cas12a targeting the *NCF2* locus at exon 15 and the 3' UTR. Red marks the stop codon of *NCF2*; grey line, target sequence; red line, PAM sequence; arrows, cleavage site

(blunt/staggered cut) of the Cas9 or Cas12a target sequence (full list of gRNA sequences are shown in **Table S1**). (**B**) *In vitro* cell-free cleavage assay of Cas9 sgRNAs and Cas12a crRNAs. The % cleavage resulting from RNP activity is shown under the graph for each reaction. Different Cas-sgRNA targeting the *NCF2* stop codon region were tested. Cas9 sgRNA #1 (subsequently referred to as sgRNA *NCF2*) was determined to have a high cleavage activity (45.8%) in an *in vitro* cell-free assay (**Figure S1B**) and in mammalian cells (39.0 ± 2.05%) by TIDE analysis (**Figure S1E**), with the Cas9 cleavage site located closest to the intended knock-in position (translational stop codon of *NCF2*). RE, restriction enzyme used to linearize template DNA. (**C**) Experimental workflow of the knock-in treatment and downstream analyses. (**D**) Indel frequency of sgRNA *NCF2* for experiments shown in **Figure 1D and 1E**. (**E**) Left: indel frequency of respective gRNAs measured by TIDE analysis PLB-985 *NCF1* ΔGT cells treated with Cas9 RNP (n=2). Right: results of the T7EI assay with the same samples (n=2 shown in 2 lanes). Asterisks mark bands of heteroduplex products cleaved by T7EI and cleavage efficiency in % is shown under the graph for each reaction. (**F**) Flow cytometry analysis of GFP expression in PLB-985 *NCF1* ΔGT cells upon treatment with Cas9 sgRNA #1 RNP + IDLV *GFP* and Cas12a crRNA #7 RNP + IDLV *GFP*. Knock-in efficiency of SpCas9 RNP and AsCpf1/Cas12a RNP was compared. Although cleavage efficiency of Cas9 sgRNA #1 and Cas12a crRNA #7 was similar, the knock-in efficiency was low when the Cas12a system was used. NT, non-treated control.

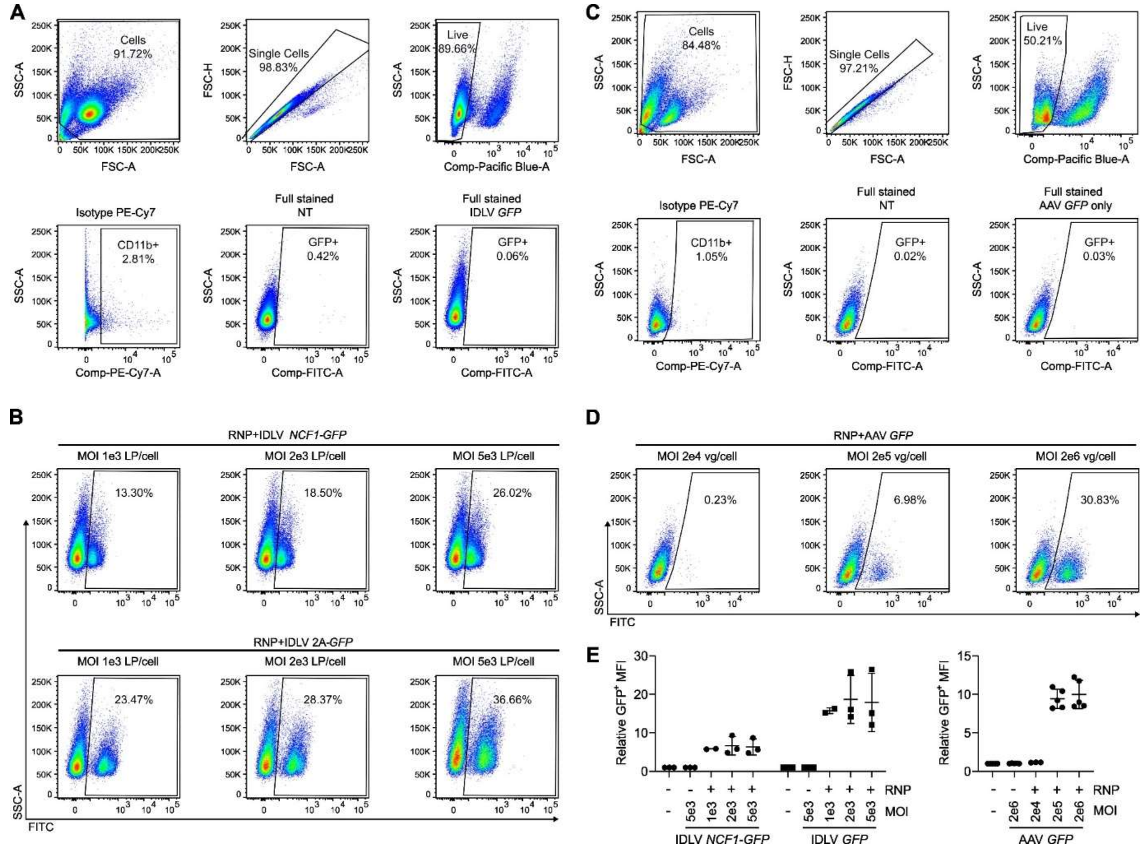

Figure S2. Gating strategy of flow cytometry analyses in PLB-985 cells shown in Figure 1D.

(**A, C**) Gating strategy for determination of GFP expression in live, CD11b-positive cells. Gating of CD11b-positive cells is based on the fluorescence minus one (FMO) control, with the addition of the isotype control PE-Cy7 antibody. Gating of the GFP-positive population is based on fully stained PLB-985 *NCF1* ΔGT cells. Gating strategy for RNP+IDLV *GFP* treated cells is presented in **A**, while for RNP + AAV *GFP* treated cells is presented in **C**. NT, non-treated control. (**B**) Representative flow cytometry plots of cells treated with RNP + IDLV *NCF1-GFP* and RNP + IDLV *GFP* at the respective MOI. (**D**) Representative flow cytometry plots showing cells treated with RNP+AAV *GFP* with the indicated MOI. (**E**) Relative GFP-positive MFI of corresponding samples in **Figure 1D;** n=2-5, data are shown as mean ± SD. MOI is denoted as lentiviral particle/cell (LP/cell) for IDLVs, or vector genomes/cell (vg/cell) for AAVs.

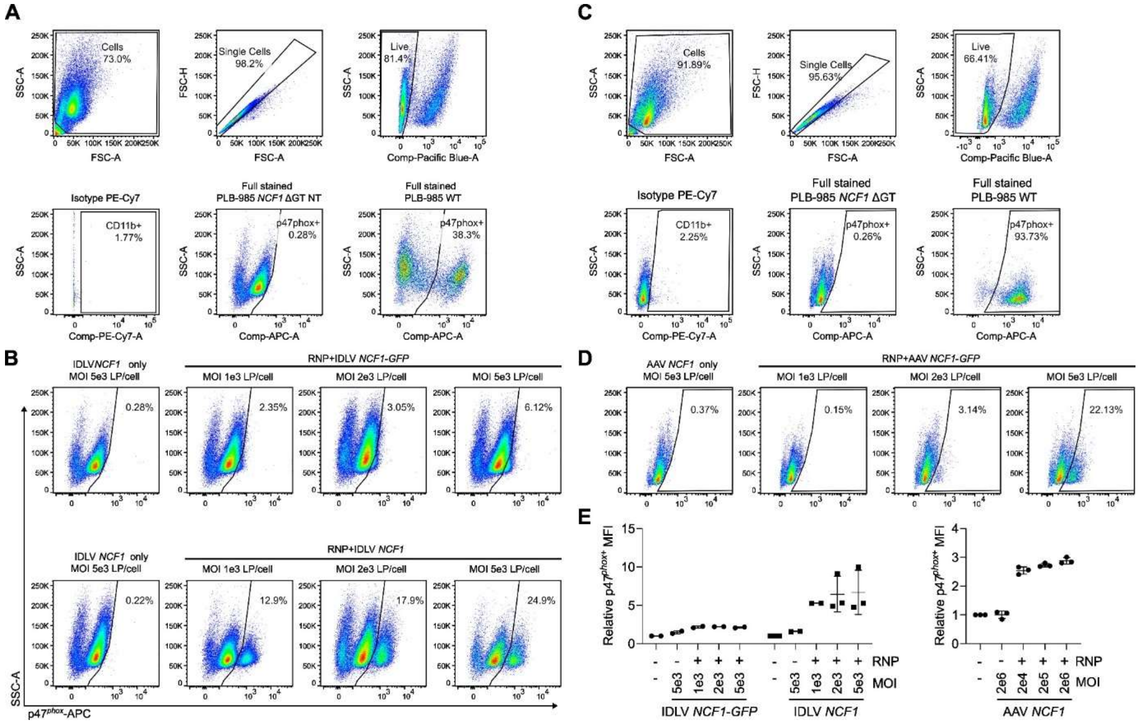

Figure S3. Gating strategy of flow cytometry analyses in PLB-985 cells shown in Figure 1E.

(**A, C**) Gating strategy for analysis of $p47^{phox}$ expression in live, CD11b-positive cells. Gating of CD11b-positive cells is set on FMO control, with the addition of the isotype control PE-Cy7 antibody. Gating of the $p47^{phox}$-positive population within the CD11b-positive population is based on fully stained PLB-985 *NCF1* ΔGT cells. Gating strategy for cells treated with RNP + IDLV *NCF1* is presented in **A**, and for cells treated with RNP + AAV *NCF1* in **C**. NT, non-treated control. (**B**) Representative flow cytometry showing cells treated with RNP + IDLV *NCF1-GFP* and RNP + IDLV *NCF1* at the indicated MOIs. (**D**) Representative flow cytometry plots showing cells treated with RNP + AAV *NCF1* at the indicated MOIs. (**E**) Relative $p47^{phox}$-positive MFI of corresponding samples in **Figure 1E;** n=2-3, data are shown as mean ± SD. MOI is denoted as LP/cell for IDLVs, or vg/cell for AAVs.

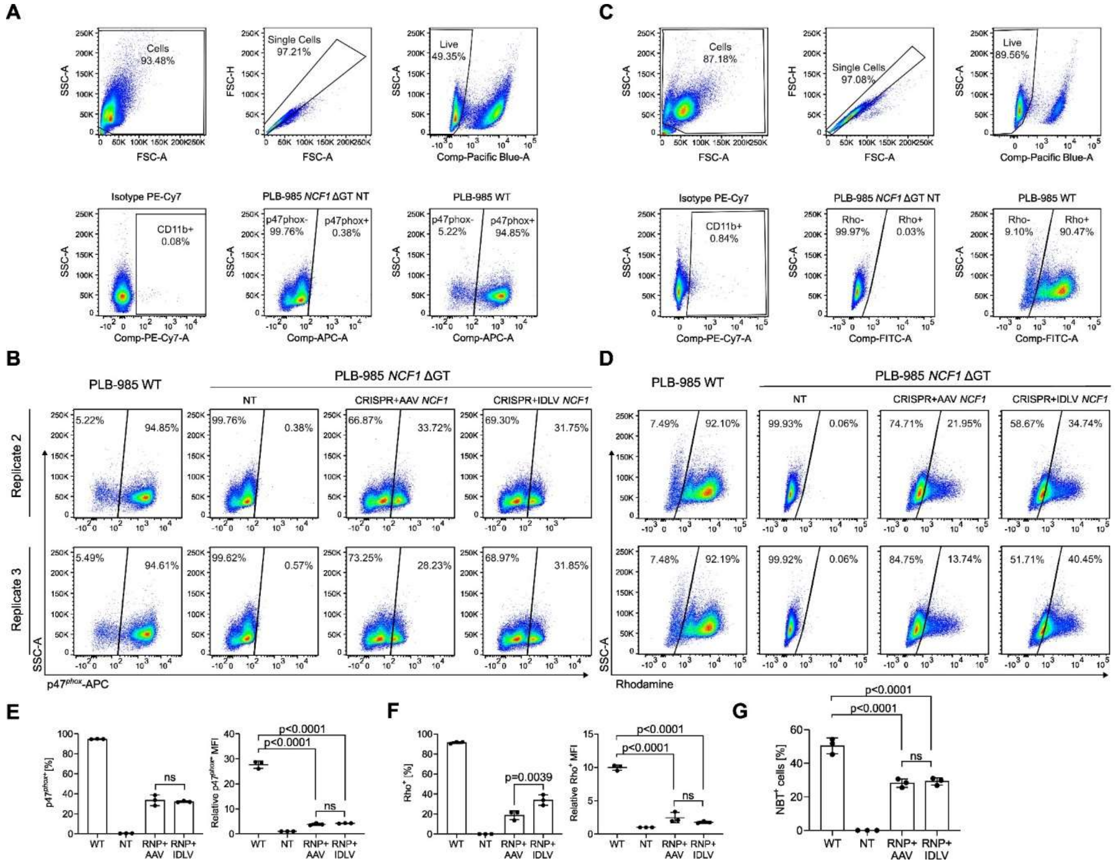

Figure S4. Gating strategy of flow cytometry analyses in PLB-985 cells shown in Figure 1F and 1G, and quantification of NBT test shown in Figure 1H

(**A**) Gating strategy in **Figure 1F** to determine p47^{phox} positive cells in live, CD11b-positive population. FMO control plus isotype control PE-Cy7 antibody were used for gating of CD11b-positive cells, followed by gating of the p47^{phox}-positive population in fully stained PLB-985 *NCF1* ΔGT cells. (**B**) Flow cytometry plots of p47^{phox}-positive populations in replicates 2 and 3 of corresponding samples in **Figure 1F**. Replicate 1 is shown as a representative plot in **Figure 1F**. (**C**) Gating strategy in **Figure 1G** to determine Rho-positive cells in live, CD11b-positive population. Gating of Rho-positive cells within the CD11b-positive population is set on fully stained, PMA-stimulated, non-treated PLB-985 *NCF1* ΔGT cells as a negative control. (**D**) Flow cytometry plots of Rho-positive populations in replicates 2 and 3 of corresponding samples in **Figure 1G**. (**E**) Bar graphs show summary data of % p47^{phox}-positive cells and relative p47^{phox}-positive MFI gated on live, CD11b-positive cells of samples in **Figure 1F**. **(F)** Bar graphs show summary data of % Rho-positive cells and relative Rho positive MFI gated on live, CD11b-positive cells of samples in **Figure 1G** (In **E** and **F**, relative MFI is calculated as the ratio of MFI-positive population over the negative population of the same sample; n=3; ns, non-significant; data are shown as mean ± SD). (**G**) Quantification of the NBT test results in

Figure 1H. Statistical analysis performed with one-way ANOVA followed by Sidak's multiple comparisons test.

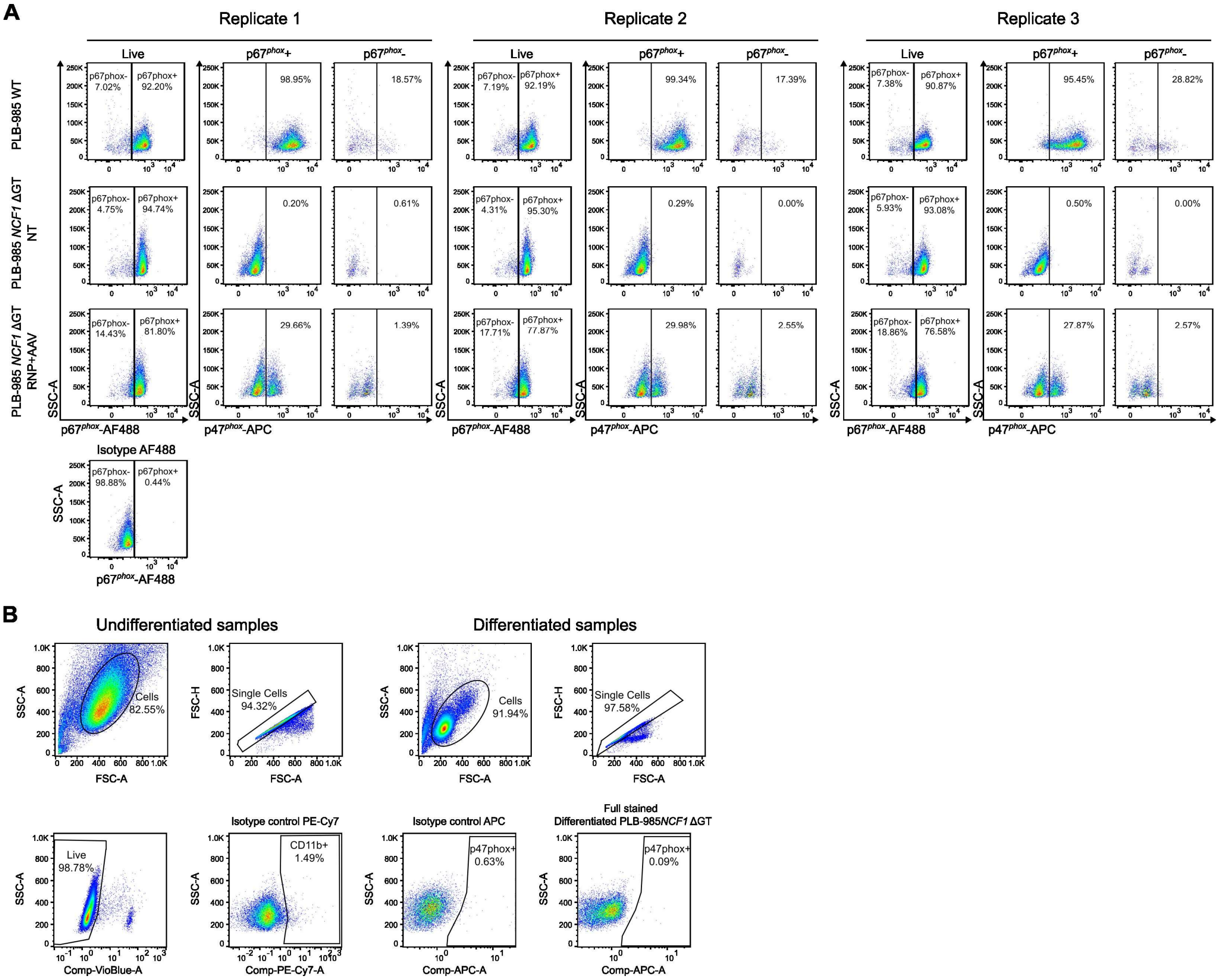

Figure S5. Gating strategy of flow cytometry analyses in PLB-985 cells shown in Figure 2A and 2B.

(**A**) Flow cytometry analyses supplementary to **Figure 2A** to determine p67^{phox} and p47^{phox} expression in three independent replicates of PLB-985 WT cells, non-treated PLB-985 *NCF1* ΔGT cells, and RNP + AAV *NCF1* treated PLB-985 *NCF1* ΔGT cells upon knock-in into *NCF2*. The plots show live cells, gated on both p67^{phox} positive and p67^{phox} negative populations that express p47^{phox}. All three replicates of cells treated with RNP + AAV *NCF1* showed p47^{phox} expression that was mainly restricted to the p67^{phox}-positive population, with a minimal leakage into the p67^{phox}-negative population (up to 2.6%). The gating of the p67^{phox} population was determined by FMO control, stained with isotype control AF488 antibody. (**B**) Gating strategy supplementary to **Figure 2B** to determine the p47^{phox}-positive population in undifferentiated cells and in myeloid-differentiated PLB-985 *NCF1* ΔGT cells, gated on

live and CD11b-positive cells. FMO controls, plus isotype control PE-Cy7 and APC antibodies were used to gate CD11b- and p47^{phox}-positive cells. NT, non-treated control.

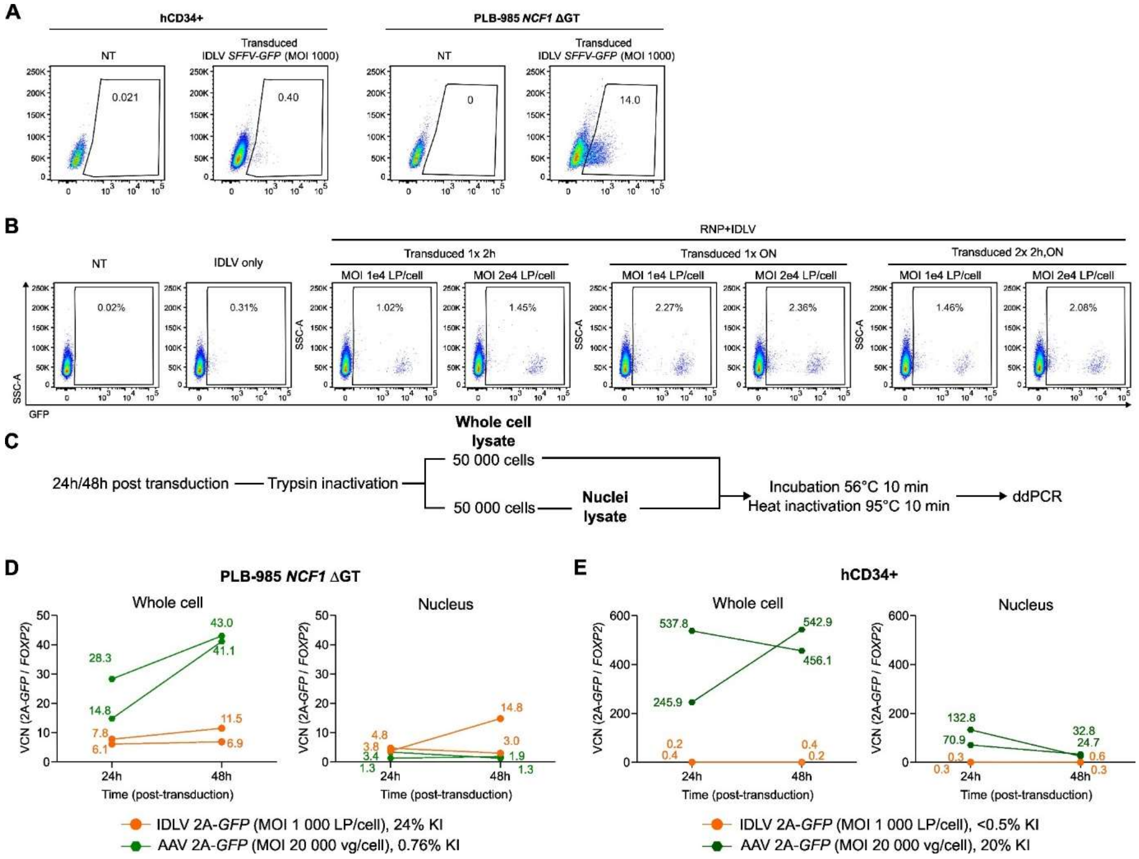

Figure S6. IDLV knock-in in human CD34+ cells is limited by transduction efficiency and cell-type tropism.

(**A**) Comparison of transduction efficiency in human CD34+ (hCD34+) cells and PLB-985 *NCF1* ΔGT cells. Cells were transduced with IDLV *SFFV-GFP* at an MOI of 1 000 LP/cell. Flow cytometry analysis of GFP expression was performed 24 hours after transduction. Transduction efficiency was low in hCD34+ cells, as compared to PLB-985 *NCF1* ΔGT cells (n=1). (**B**) Determination of knock-in efficiency in hCD34+ cells treated with RNP + IDLV *SFFV-GFP*. Cells were electroporated with RNPs targeting the *NCF2* locus, as described in 'Materials and methods', followed by transduction with IDLV *SFFV-GFP* at an MOI 1x10^4 or 2x10^4 LP/cell: one time for 2h, one time overnight (ON), or two times for 2h and overnight. After treatment, cells were differentiated towards myeloid lineages for 2 weeks and GFP expression was analysed by flow cytometry. At all tested transduction conditions, the IDLV knock-in efficiency in hCD34+ cells was low, not exceeding 2.4%. (**C**) Scheme of sample preparation for ddPCR-based determination of the vector copy number (VCN). Cells were transduced with IDLV 2A-*GFP,* or AAV 2A-*GFP* for 24h or 48h. Prior to nuclei isolation, cells were washed in

PBS twice, and treated with trypsin to remove remaining viral vector particles bound to the cell surface. Nuclei isolation was performed with Monarch® gDNA nuclei prep and lysis buffer pack (NEB, USA), according to the manufacturer's instructions. Two fractions of whole cells (including nuclei and cytoplasm) or nuclei only were isolated and subjected to DNA isolation by Buffer K lysis (as described in 'Materials and methods'). Isolated DNA samples contained host gDNA and AAV genomes. (**D, E**) VCN of 2A-*GFP* determined by ddPCR, normalized to two copies of *FOXP2* in the host genome of (**D**) PLB-985 *NCF1* ΔGT cells and (**E**) hCD34+ cells. PLB-985 *NCF1* ΔGT cells and hCD34+ cells were transduced with IDLV *GFP* or AAV *GFP* at the indicated MOI for 24h or 48h prior to DNA isolation from whole cells or nuclei. Each data point of similar colour represents replicates (n=2). Transduction of PLB-985 *NCF1* ΔGT cells with IDLVs was efficient, with the highest VCN of 11.5 copies detected in the whole cell samples, and 3.4 copies in the nuclei samples. In hCD34+ cells, there were less than 0.6 copies detected in both whole cell and nuclei samples, suggesting that either the IDLVs used in our experiments (D64V) have a low tropism to hCD34+ cells, or reverse transcription of lentiviral RNA to DNA was impaired and/or delayed. This observation was contrary to the previously published data showing that IDLVs outperformed AAVs in template delivery.[10] High VCN in AAV-transduced hCD34+ cells (up to 543 copies in the whole cell samples) indicated that AAV serotype 6 is highly efficient in targeting hCD34+ cells, while it is less active in PLB-985 cells (up to 43 copies in whole cell samples). When comparing nuclear transport of vector DNA, IDLVs were more efficiently transported into the nucleus than AAVs (*e.g.,* 7.8 copies in the whole cell samples, and 4.8 copies in the nuclei samples at 24h post transduction). A higher MOI was needed for AAV transduction as the nuclear transport was poor, suggesting the high particle-to-infectivity ratio.

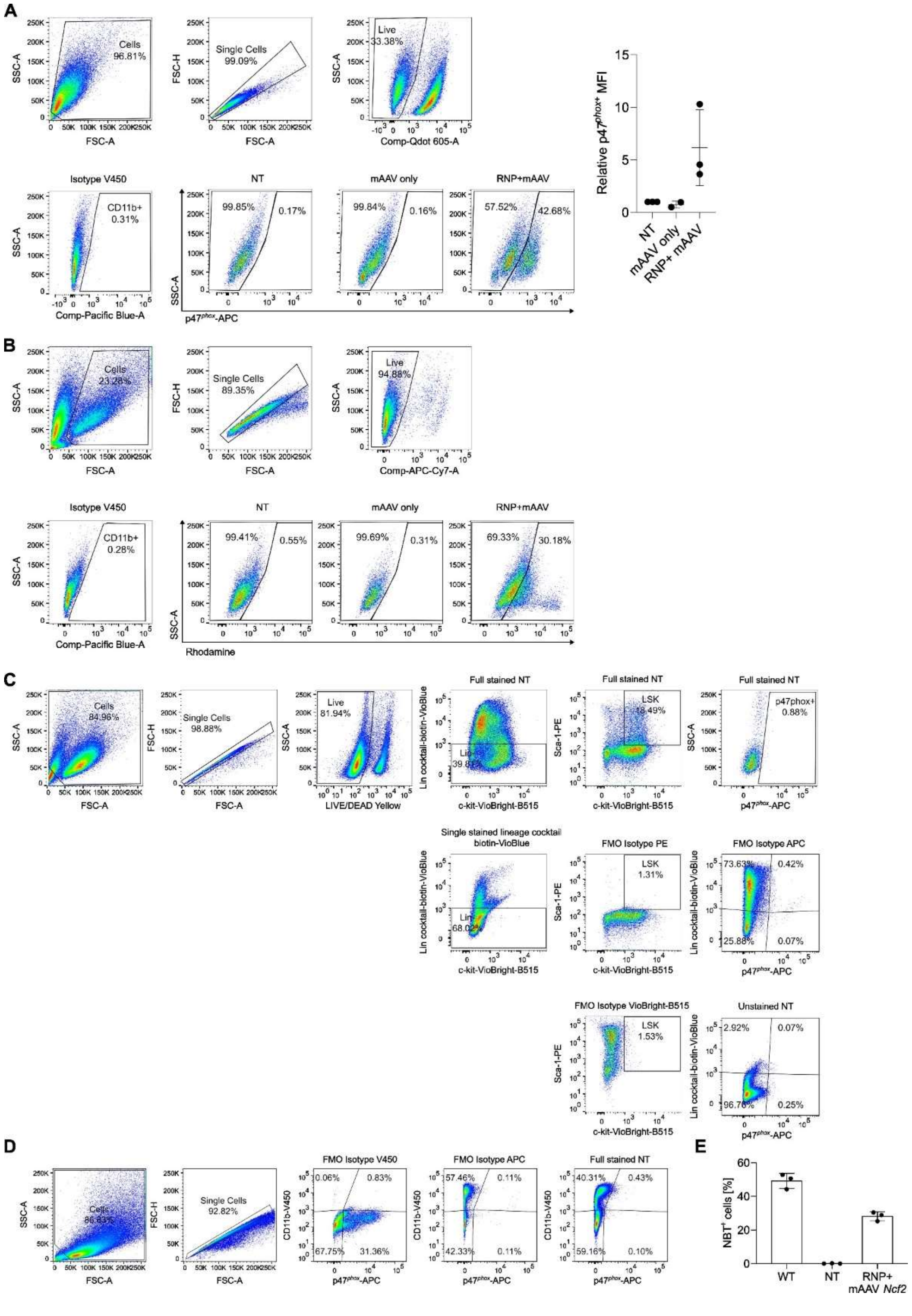

Figure S7. Gating strategy of flow cytometry analyses in lineage-negative mouse hematopoietic stem cells supplementary to Figure 3B, 3C, 3E and 3F.

(**A**) Expression of p47*phox* was gated on live, CD11b-positive cells. FMO control, stained with isotype control V450 antibody, was used for CD11b gating. Gating of p47*phox*-positive cells was set on fully stained p47*phox*-deficient mouse hematopoietic stem cells (mHSPCs) as negative control. The relative p47*phox*-positive MFI of corresponding samples is shown in **Figure 3B**; n=2-3, data presented as mean

± SD. (**B**) Gating strategy determining Rho-positive cells in the live, CD11b-positive population. CD11b gating was set on FMO control plus isotype control V450 antibody. Rho positive cells were gated on fully stained and PMA-stimulated, non-treated p47*phox*-deficient mHSPCs. (**C**) Gating strategy supplementary to **Figure 3E**, determining p47*phox* expression in LSK (Lin- Sca-1+ c-Kit+) cells in mHSPCs before differentiation. Gating was done on the live and Lin- population, followed by Sca-1 versus c-Kit, gated on FMO controls, stained with isotype control PE and VioBright-B515, respectively. Within the Lin- population, p47*phox* expression was determined on fully stained non-treated p47*phox*-deficient mHSPCs as negative control. Alternative quadrant gating with Lin- versus p47*phox* was performed to check for transgene expression in the undifferentiated population. (**D**) Gating strategy supplementary to **Figure 3F** to determine p67*phox* versus p47*phox* expression on live cells, after myeloid differentiation of p47*phox*-deficient mHSPCs. (**E**) Quantification of formazan-positive cells in NBT test shown in **Figure 3D**.

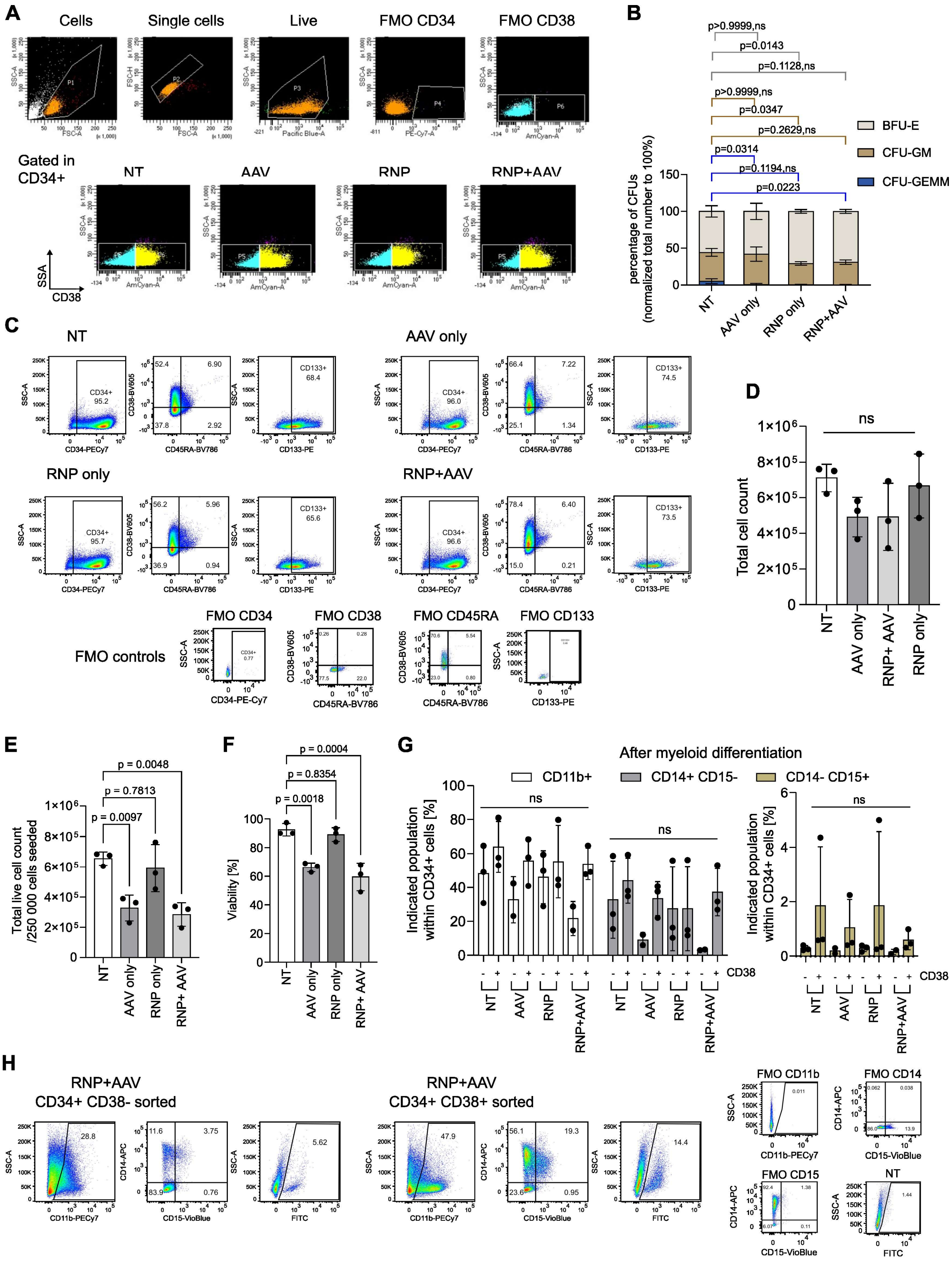

Figure S8. Editing of healthy human CD34+ hematopoietic stem cells shown in Figure 4A-D.

(**A**) Gating strategy supplementary to **Figure 4A and 4C** for FACS sorting of $CD34^+$ $CD38^+$ and $CD34^+$ $CD38^-$ subpopulation 2 days after editing of human HSPCs from healthy donors. CD34 and CD38 gates were set on FMO controls. (**B**) Percentage of CFUs normalized to the total number of colonies per sample shown in **Figure 4B**; n=3 donors, with 2 technical replicates per donor; data are shown as mean ± SD; BFU-E, burst-forming unit-erythroid; CFU-G, colony-forming unit granulocyte; CFU-M, colony-forming unit macrophage; CFU-GEMM, colony-forming unit granulocyte, erythrocyte, monocyte, megakaryocyte; one-way ANOVA with Dunnett's multiple comparisons test for CFU-GM and BFU-E, Kruskal-Wallis test with Dunn's multiple comparisons test for CFU-GEMM. (**C**) Representative flow cytometry plots showing CD34, CD38 versus CD45RA, CD133 expression in samples shown in **Figure 4C** and their respective FMO controls for gating. (**D**) Total cell count of healthy HSPCs 2 days after editing. (**E**) Total live cells and (**F**) percentage of cell viability as counted by trypan blue exclusion test 2 days after editing of 250 000 cells as starting material. (**G**) Percentage of CD11b+ (myeloid cells), CD14+ CD15- (monocyte/macrophages) and CD14- CD15+ (granulocytes) subpopulation in edited and sorted healthy HSPCS 14 days after myeloid differentiation. Myeloid subpopulations were not significantly different between $CD34^+$ $CD38^+$ and $CD34^+$ $CD38^-$ fractions nor between treatments. Results in **D-F** correspond to the state of cells shown in **Figure 4C**. In **D-G**, n=2 healthy donors, 3 technical replicates; data are shown as mean ± SD; one-way ANOVA followed by Dunnett's multiple comparisons test in **D-F** and Brown-Forsythe and Welch ANOVA test followed by Dunnett's T3 multiple comparisons test in **G**. (**H**) Representative flow cytometry plots showing CD11b, CD14 versus CD15, and GFP expression of edited and $CD34^+$ $CD38^+$/$CD34^+$ $CD38^-$ sorted fractions shown in **Figure 4D** 14 days after myeloid differentiation. FMO controls were used as gating reference.

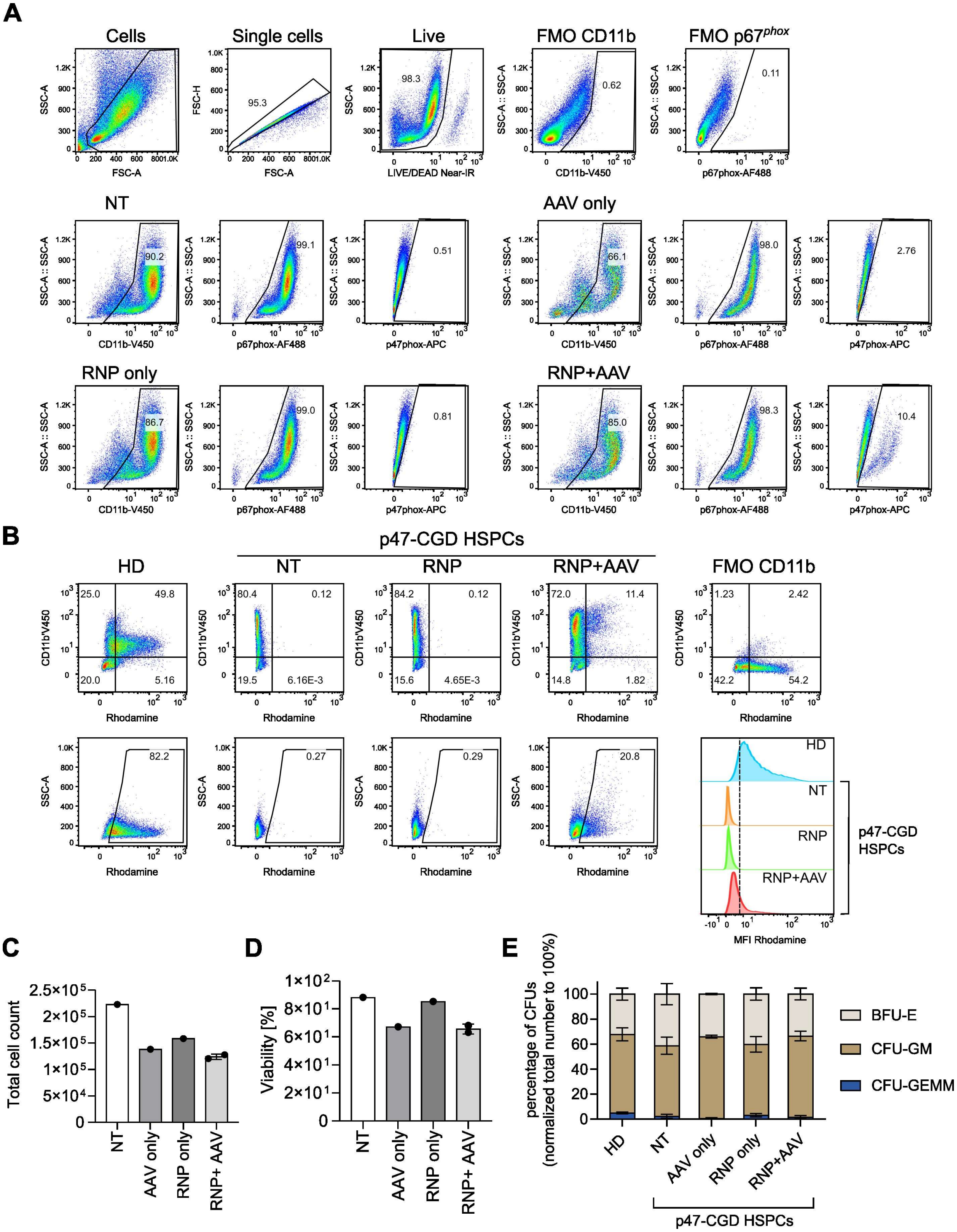

Figure S9. Editing of p47-CGD patient CD34+ hematopoietic stem cells shown in Figure 4E-J.

(**A**) Gating strategy supplementary to **Figure 4F 4H, and 4J** and their representative flow cytometry plots showing CD11b, p67*phox*, and p47*phox* expression of edited p47-CGD patient HSPCs after 14 days

of myeloid differentiation. (**B**) Representative flow cytometry plots of DHR tests performed on samples shown in **Figure 4G and 4I**, showing rhodamine positive cells gated within CD11b positive population. Rhodamine MFI is alternatively represented in a histogram. (**C**) Total cell count of p47-CGD HSPCs 2 days after editing. (**D**) Percentage of cell viability as counted by trypan blue exclusion test 2 days after editing of 250 000 p47-CGD HSPCs as starting material. (**E**) Percentage of CFUs normalized to the total number of colonies per sample shown in **Figure 4E**; n=1 donor, with 3 technical replicates; data are shown as mean ± SD.

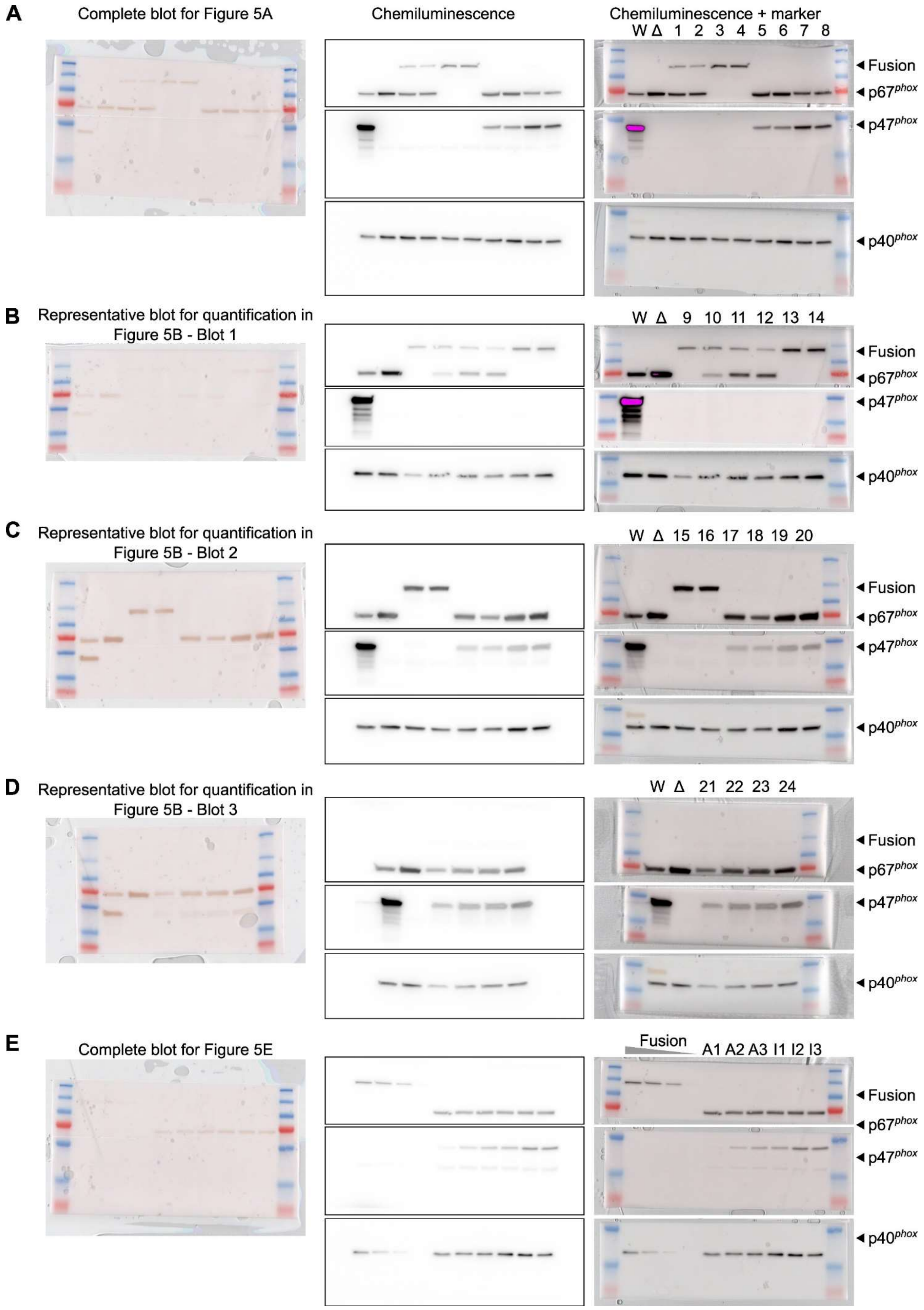
A
Complete blot for Figure 5A
Chemiluminescence
Chemiluminescence + marker
W Δ 1 2 3 4 5 6 7 8
◀ Fusion
◀ p67phox
◀ p47phox
◀ p40phox
B
Representative blot for quantification in Figure 5B - Blot 1
W Δ 9 10 11 12 13 14
◀ Fusion
◀ p67phox
◀ p47phox
◀ p40phox
C
Representative blot for quantification in Figure 5B - Blot 2
W Δ 15 16 17 18 19 20
◀ Fusion
◀ p67phox
◀ p47phox
◀ p40phox
D
Representative blot for quantification in Figure 5B - Blot 3
W Δ 21 22 23 24
◀ Fusion
◀ p67phox
◀ p47phox
◀ p40phox
E
Complete blot for Figure 5E
Fusion
A1 A2 A3 I1 I2 I3
◀ Fusion
◀ p67phox
◀ p47phox
◀ p40phox

Figure S10. Uncropped and unexposed western blot membranes shown in Figure 5A, 5B, and 5E.

(**A**) Complete uncropped blot (left) for **Figure 5A** imaged with protein ladder, PageRuler™ Plus Prestained Protein Ladder 10 to 250 kDa (Thermo Fisher Scientific, USA). The membrane was cut-split at the level of approximately 55 kDa for separate antibody incubations before imaging. Unexposed (middle) western blot membrane, immuno-stained for p67^{phox}-2A*-p47^{phox} fusion (using anti-human p67^{phox} antibody), anti-human p67^{phox}, anti-p47^{phox} antibody, and anti-p40^{phox} antibody conjugated with horse radish peroxidase (HRP) imaged by chemiluminescence detection, alongside the same membrane imaged for protein ladder (right). Protein samples loaded were: W, PLB-985 WT; Δ, PLB-985 *NCF1* ΔGT; 1 and 2, 'Fused hetero' clones; 3 and 4, 'Fused homo' clones; 5 and 6, 'Cleaved hetero' clones; 7 and 8, 'Cleaved homo' clones. (**B-D**) Representative blots of one set of differentiation experiment used for quantification in **Figure 5B**. Same set of clones were differentiated in 3 independent differentiation experiments and quantified for absolute intensities using ImageJ; n=16 clones. Protein samples loaded were: (**B**) 9 to 12, 'Fused hetero' clones; 13 and 14, 'Fused homo' clones; (**C**) 15 and 16, 'Fused homo' clones; 17 to 20, 'Cleaved hetero' clones; (**D**) 21 to 24, 'Cleaved homo' clones. (**E**) Uncropped, unexposed blot used in **Figure 5E**. Protein samples loaded were: A1-A3, RNP+AAV *NCF1* treated PLB-985 cells in bulk culture with 3 different (increasing) MOI; I1-I3, RNP+IDLV *NCF1* treated PLB-985 cells in bulk culture with increasing MOI. Arrowheads indicate the expected location of respective proteins based on the ladder position.

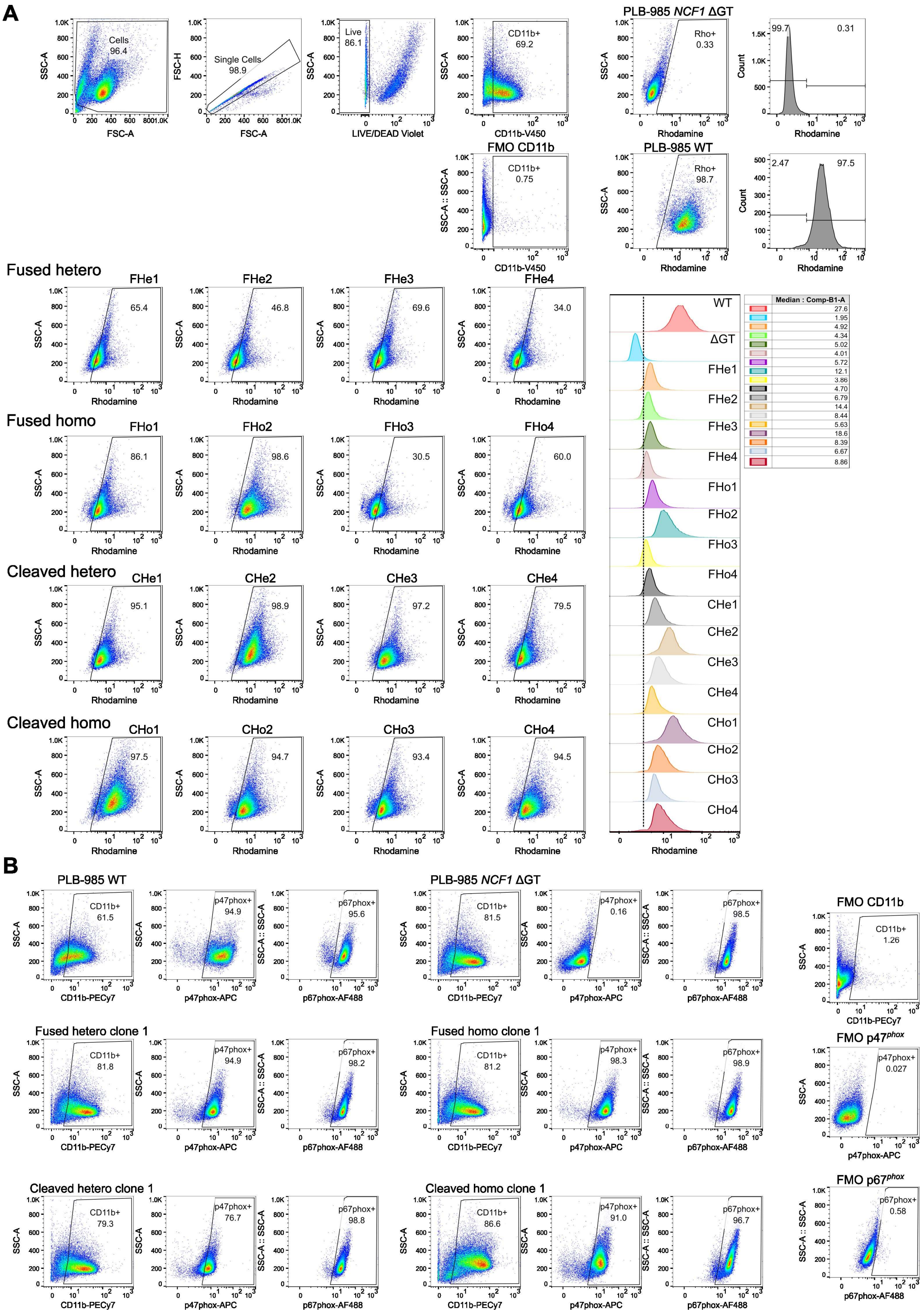
A
Cells 96.4
Single Cells 98.9
Live 86.1
CD11b+ 69.2
PLB-985 NCF1 ΔGT
Rho+ 0.33
99.7
0.31
FMO CD11b
CD11b+ 0.75
PLB-985 WT
Rho+ 98.7
2.47
97.5
SSC-A
FSC-A
FSC-H
LIVE/DEAD Violet
CD11b-V450
SSC-A :: SSC-A
Rhodamine
Count
Fused hetero
FHe1 65.4
FHe2 46.8
FHe3 69.6
FHe4 34.0
Fused homo
FHo1 86.1
FHo2 98.6
FHo3 30.5
FHo4 60.0
Cleaved hetero
CHe1 95.1
CHe2 98.9
CHe3 97.2
CHe4 79.5
Cleaved homo
CHo1 97.5
CHo2 94.7
CHo3 93.4
CHo4 94.5
WT
ΔGT
FHe1
FHe2
FHe3
FHe4
FHo1
FHo2
FHo3
FHo4
CHe1
CHe2
CHe3
CHe4
CHo1
CHo2
CHo3
CHo4
Median : Comp-B1-A
27.6
1.95
4.92
4.34
5.02
4.01
5.72
12.1
3.86
4.70
6.79
14.4
8.44
5.63
18.6
8.39
6.67
8.86
B
PLB-985 WT
CD11b+ 61.5
p47phox+ 94.9
p67phox+ 95.6
PLB-985 NCF1 ΔGT
CD11b+ 81.5
p47phox+ 0.16
p67phox+ 98.5
FMO CD11b
CD11b+ 1.26
Fused hetero clone 1
CD11b+ 81.8
p47phox+ 94.9
p67phox+ 98.2
Fused homo clone 1
CD11b+ 81.2
p47phox+ 98.3
p67phox+ 98.9
FMO p47phox
p47phox+ 0.027
Cleaved hetero clone 1
CD11b+ 79.3
p47phox+ 76.7
p67phox+ 98.8
Cleaved homo clone 1
CD11b+ 86.6
p47phox+ 91.0
p67phox+ 96.7
FMO p67phox
p67phox+ 0.58
CD11b-PECy7
p47phox-APC
p67phox-AF488

Figure S11. Flow cytometry analysis and DHR tests performed on 'Fused' and 'Cleaved' clones shown in Figure 5D.

(**A**) Gating strategy of a DHR test performed on 'Fused' and 'Cleaved' clones. Rhodamine positive cells were gated within CD11b positive population. FMO controls were used for CD11b gating and NT PLB-985 *NCF1* GT cells were referred as negative for rhodamine signal. Representative flow cytometry plots and histograms of rhodamine MFI of clones shown in **Figure 5D,** gated for rhodamine positive cells and rhodamine MFI values. 'Fused hetero' or 'Fused homo' clones were abbreviated as FHe and FHo, 'Cleaved hetero' or 'Cleaved homo' clones were abbreviated as CHe and CHo. (**B**) Representative flow cytometry plots showing CD11b expression, $p47^{phox}$ expression within CD11b positive cells and $p67^{phox}$ expression within CD11b positive cells in 'Fused' and 'Cleaved' clones. The MFI values of $p67^{phox}$ gated within CD11b positive cells were used for normalization of the clones' differentiation status in **Figure 5D**.

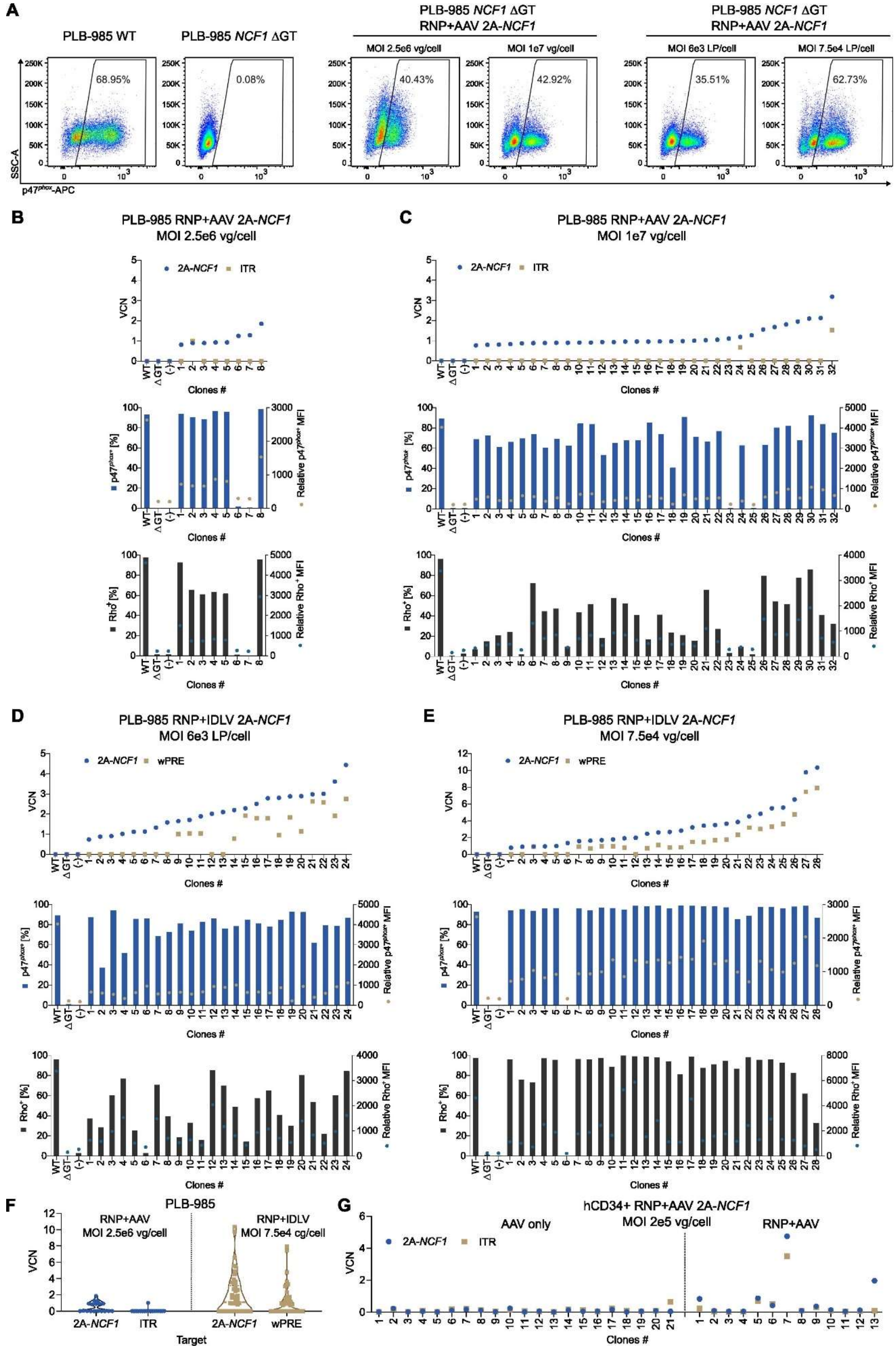

Figure S12. Detailed characterization of individual clones knocked-in with 2A-*NCF1* shown in Figure 6A.

(**A**) Flow cytometry analysis of p47^{phox} expression to determine knock-in efficiency prior to single clone generation by fluorescence activated cell sorting. PLB-985 *NCF1* ΔGT cells were treated with RNP+AAV *NCF1* or with IDLV *NCF1* at the indicated MOIs, differentiated intro granulocytic-like cells, and analysed by flow cytometry for p47^{phox} expression. (**B, C**) VCN, % p47^{phox} expression, and % Rho-positive cells, and the respective absolute MFI of PLB-985 *NCF1* ΔGT cells treated with RNP + AAV *NCF1* at an MOI of 2.5x10^6 vg/cell (**B**, n=8) or 1x10^7 vg/cell (**C**, n=32). ITR, inverted terminal repeat; wPRE, woodchuck hepatitis virus post-transcriptional regulatory element. (**D, E**) VCN, % p47^{phox} expression, and % Rho-positive cells, and the respective absolute MFI of PLB-985 *NCF1* ΔGT cells treated with RNP + IDLV *NCF1* at an MOI of 6x10^3 LP/cell (**D**, n=24) or 7.5x10^4 LP/cell (**E**, n=28). (**F**) VCN of 2A-*NCF1* versus ITR or wPRE in PLB-985 *NCF1* ΔGT cells treated with RNP + AAV *NCF1* or with IDLV *NCF1*. Each data point represents an individual clone. The higher the MOI used during gene editing, the higher was the frequency of unintended integration events detected (unintended integrations mean integrations other than the on-target knock-in at *NCF2*). IDLV-transduced samples showed higher frequency of unintended integration events (*e.g.*, at MOI 6x10^3 LP/cell, unintended integration events=14/24, knock-in efficiency of 35.6%) when compared with AAV-transduced samples (*e.g.*, at MOI 1x10^7 vg/cell, unintended integration events=2/32, knock-in efficiency of 43.0%) even with higher knock-in efficiencies. (**G**) VCN of 2A-*NCF1* versus ITR in hCD34+ cells treated with RNP + AAV *NCF1*.

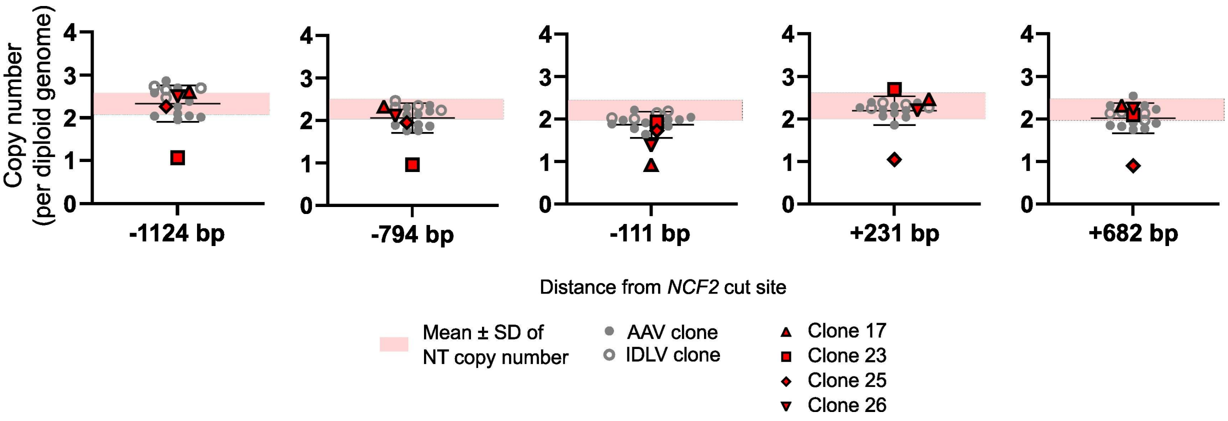

Figure S13. Evaluation of copy number gains or losses surrounding the *NCF2* on-target site.

Copy number detected 5 locations on the *NCF2* gene (in reference to 2 copies of *FOXP2* housekeeping gene) was evaluated in 13 AAV knocked-in clones (grey dots) and 5 IDLV knocked-in clones (unfilled grey circles). T1 to T5 represents primers and probes detecting copy number surrounding the *NCF2* cut site at positions -1124, -794bp, -111, +231 and +682 bp, respectively. A reference bar (pink) was set based on the mean ± SD of copy numbers determined from 15 non-treated PLB-985 WT or ΔGT

cells. Any copy number loss that deviates from the reference bar is considered as a deletion at the indicated location. Clones 17, 23, 25 and 26 were identified to have copy number losses surrounding the five sites. Referring to the flow cytometry data in **Figure S12**, the clones 23 and 25 indeed did not express p47*phox* and did not produce ROS.

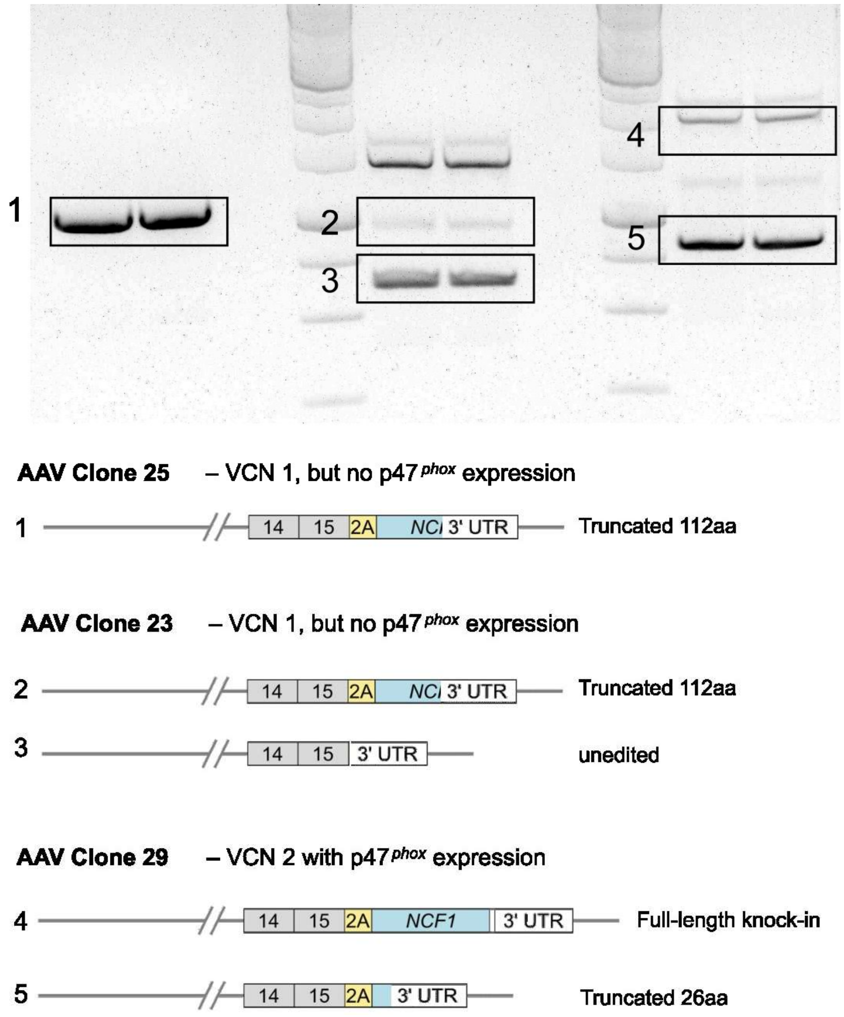

Figure S14. Several knocked-in clones carried truncated *NCF1* cDNA fragment.

(**A**) AAV knocked-in clones 23, 25 and 29 carried truncated *NCF1* cDNA at the *NCF2* target site as confirmed by Sanger sequencing. 2.3kb surrounding *NCF2* cut site was PCR amplified and gel extracted before sending for Sanger sequencing. Clones 23 and 25 were also identified to have on-target deletion by copy number ddPCR and to not express p47*phox*/produce ROS (**Figure S12 and S13**).
