# Supplementary material for: Targeted knock-in of NCF1 cDNA into the NCF2 locus leads to myeloid phenotypic correction of p47phox-deficient chronic granulomatous disease
Source: Mol Ther Nucleic Acids. 2024 May 24;35(3):102229. doi: 10.1016/j.omtn.2024.102229 (PMC11215332; doi:10.1016/j.omtn.2024.102229)
Supplement: Document S2. Article plus supplemental information [file mmc2.pdf]

Molecular Therapy
Nucleic Acids
Original Article

Targeted knock-in of *NCF1* cDNA into the *NCF2* locus leads to myeloid phenotypic correction of p47^{phox}-deficient chronic granulomatous disease

Kah Mun Siow,[1] Merve Güngör,[1] Dominik Wrona,[1] Federica Raimondi,[1] Oleksandr Pastukhov,[1] Panagiotis Tsapogas,[1] Timon Menzi,[1] Michael Schmitz,[2] Péter István Kulcsár,[3] Gerald Schwank,[3] Ansgar Schulz,[4] Martin Jinek,[2,8] Ute Modlich,[1,8] Ulrich Siler,[1,5,8] and Janine Reichenbach[1,6,7,8]

[1]Division of Gene and Cell Therapy, Institute for Regenerative Medicine, University of Zurich, Schlieren, 8952 Zurich, Switzerland; [2]Department of Biochemistry, University of Zurich, 8057 Zurich, Switzerland; [3]Institute of Pharmacology and Toxicology, University of Zurich, 8057 Zurich, Switzerland; [4]Department of Pediatrics, University Medical Center Ulm, 89075 Ulm, Germany; [5]School of Life Sciences, Institute for Pharma Technology, University of Applied Sciences and Arts Northwestern Switzerland, 4132 Muttenz, Switzerland; [6]Department of Somatic Gene Therapy, University Children's Hospital Zurich, 8032 Zurich, Switzerland; [7]Center for Applied Biotechnology and Molecular Medicine (CABMM), University of Zurich, 8057 Zurich, Switzerland

p47^{phox}-deficient chronic granulomatous disease (p47-CGD) is a primary immunodeficiency caused by mutations in the neutrophil cytosolic factor 1 (*NCF1*) gene, resulting in defective NADPH oxidase function in phagocytes. Due to its complex genomic context, the *NCF1* locus is not suited for safe gene editing with current genome editing technologies. Therefore, we developed a targeted *NCF1* coding sequence knock-in by CRISPR-Cas9 ribonucleoprotein and viral vector template delivery, to restore p47^{phox} expression under the control of the endogenous *NCF2* locus. *NCF2* encodes for p67^{phox}, an NADPH oxidase subunit that closely interacts with p47^{phox} and is predominantly expressed in myeloid cells. This approach restored p47^{phox} expression and NADPH oxidase function in p47-CGD patient hematopoietic stem and progenitor cells (HSPCs) and in p47^{phox}-deficient mouse HSPCs, with the transgene expression following a myeloid differentiation pattern. Adeno-associated viral vectors performed favorably over integration-deficient lentiviral vectors for template delivery, with fewer off-target integrations and higher correction efficacy in HSPCs. Such myeloid-directed gene editing is promising for clinical CGD gene therapy, as it leads to the co-expression of p47^{phox} and p67^{phox}, ensuring spatiotemporal and near-physiological transgene expression in myeloid cells.

INTRODUCTION

Chronic granulomatous disease (CGD) is a group of primary immunodeficiency disorders characterized by defective respiratory bursts in phagocytes, which are caused by mutations in genes encoding subunits of the nicotinamide adenine dinucleotide phosphate (NADPH) oxidase, including p47^{phox} and p67^{phox}.[1,2] The resulting impaired NADPH oxidase function leads to the loss of reactive oxygen species (ROS) production, accounting for life-threatening bacterial and fungal infections, as well as hyperinflammation in affected patients.[3,4] A potentially curative treatment for CGD is the transplantation of genetically modified autologous hematopoietic stem and progenitor cells (HSPCs). Several research groups have explored various *ex vivo* gene therapy approaches, including retroviral gene addition,[5–7] direct mutation targeting with designer nucleases,[8,9] safe harbor targeting,[10–13] exon replacement,[14] or minigene insertion,[15] to correct different genetic subtypes of CGD.

In this study, we focused on p47^{phox}-deficient CGD (p47-CGD), which in the majority of patients is caused by a two-nucleotide deletion (ΔGT) in the neutrophil cytosolic factor 1 (*NCF1*) gene.[16] The mutated gene is colocalized on the same chromosome with two homologous pseudogenes, *NCF1B* and *NCF1C*, which display a high degree of sequence similarity to *NCF1*, and also carry the ΔGT sequence.[17,18] Consequently, gene editing approaches that target the ΔGT sequence can simultaneously induce multiple double-strand breaks (DSBs) in the *NCF1* loci, ultimately leading to chromosomal aberrations, which could limit the potential clinical application of such strategies.[8,19]

To avoid such adverse outcomes, we developed an alternative gene editing approach using CRISPR-Cas9 and homology-directed repair (HDR)-mediated knock-in, inserting a promoter-less *NCF1* cDNA into the 3′ end of the endogenous *NCF2* gene. This gene has been selected as the target for *NCF1* knock-in, as the encoded p67^{phox} protein is predominantly expressed in myeloid cells,[20–22] and exists as a tight complex with p47^{phox} in the resting state and in the activated NADPH oxidase.[23,24] To ensure spatiotemporal regulation of the transgenic p47^{phox}, we coupled both subunits into a single reading

Received 3 October 2023; accepted 22 May 2024;
https://doi.org/10.1016/j.omtn.2024.102229.

[8]These authors contributed equally

Correspondence: Janine Reichenbach, Division of Gene and Cell Therapy, Institute for Regenerative Medicine, University of Zurich, 8952 Schlieren, Zurich, Switzerland.
E-mail: janine.reichenbach@irem.uzh.ch

This is an open access article under the CC BY license (http://creativecommons.org/licenses/by/4.0/).

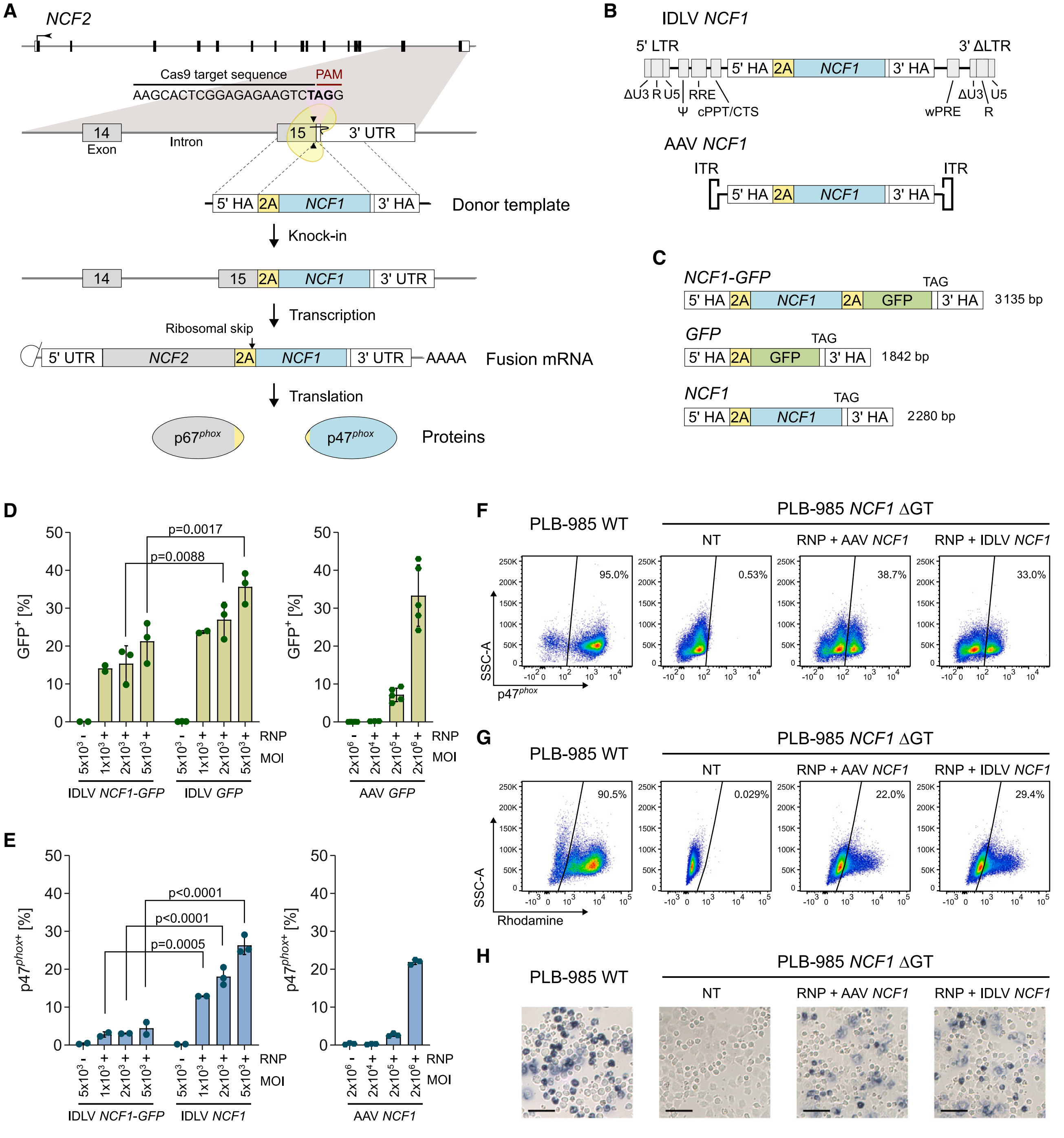

Figure 1. Targeted knock-in of *NCF1* cDNA into the *NCF2* locus restores p47*phox* expression and ROS production in PLB-985 *NCF1* ΔGT cells

(A) Schematic diagram of the knock-in strategy targeted at the translational stop codon of the *NCF2* gene. The stop codon (TAG in bold) was targeted by the gRNA for cleavage (arrowheads) by Cas9. Gray boxes indicate exons of *NCF2*. PAM, protospacer adjacent motif; TAG in bold, translational stop codon; HA, homology arm; 2A, FMDV 2A oligopeptide; *NCF1*, *NCF1* codon-optimized full-length cDNA; UTR, 3′ untranslated region; AAAA, poly(A) tail. (B) Types of vectors carrying the IDLV *NCF1* and AAV *NCF1* donor template. LTR, long terminal repeat; ΔU3, self-inactivating untranslated 3′; R, repeat region; U5, untranslated 5′; Ψ, psi; RRE, Rev response element; CPPT/CTS, central polypurine tract/central termination sequence; wPRE, woodchuck hepatitis virus post-transcriptional regulatory element; ITR, inverted terminal repeat of AAVs. (C) Design of donor templates denoted with their respective names and constructs' length (bp). GFP, green fluorescence protein; TAG, translational stop codon. (D) Flow cytometry analysis of GFP expression in the CD11b-positive population upon granulocytic differentiation of PLB-985 *NCF1* ΔGT cells, treated with CRISPR-Cas9 RNP and

(legend continued on next page)

frame using the 2A co-expression system,[25,26] limiting the p47^{phox} expression to the myeloid compartment of the hematopoietic system.

RESULTS

Knock-in of *NCF1* cDNA into the *NCF2* locus restores p47^{phox} expression in p47^{phox}-deficient cells

We used CRISPR-Cas9-mediated gene editing to integrate a full-length *NCF1* cDNA downstream of the *NCF2* reading frame (Figure 1A). First, we designed and identified a single-guide RNA (sgRNA) that targets the *NCF2* stop codon (Figures S1A, S1B, and S1D–S1F; Table S1). The donor template consisted of a foot-and-mouth disease virus (FMDV) 2A sequence,[26] linked to a codon-optimized cDNA of the human *NCF1*, and flanked by two ~0.5 kb homology arms on both ends, and was delivered into the cells by an adeno-associated viral vector (AAV) serotype 6, or by an integration-deficient lentiviral vector (IDLV) (Figure 1B). We hypothesized that by coupling the *NCF1* cDNA to the *NCF2* locus, p47^{phox} production should depend on the expression of the endogenous p67^{phox} and the separation by 2A-mediated ribosomal skipping (Figure 1A).[27]

We considered that corrective templates of different lengths may yield different knock-in efficiency, and therefore evaluated several templates: the construct 2A-*NCF1* (*NCF1*) that expresses the therapeutic p47^{phox}, the reporter 2A-*GFP* (*GFP*), or a combination of 2A-*NCF1*-2A-*GFP* (*NCF1-GFP*) as a cDNA-directed reporter (Figure 1C). To assess the knock-in efficiency, we electroporated PLB-985 *NCF1* ΔGT cells[28] with a ribonucleoprotein (RNP) complex consisting of Cas9 protein and an sgRNA targeting the *NCF2* stop codon (sgRNA *NCF2*), followed by transduction of the cells with viral vectors that carry the donor templates (Figures 1B and S1A). After this treatment, the edited cells were differentiated into granulocyte-like cells and analyzed for the presence of p47^{phox} protein (Figure S1C).

As expected, increasing multiplicity of infection (MOI) resulted in a dose-dependent increase of the percentage of transgene-expressing cells (Figures 1D and 1E). The use of short templates consistently improved the knock-in efficiency compared with the long templates: comparing IDLV *GFP* and IDLV *NCF1-GFP*, edited PLB-985 *NCF1* ΔGT cells resulted in 1.7-fold more green fluorescent protein (GFP)-expressing cells when treated with the highest MOI (5×10^3 lentiviral particles [LPs]/cell, 35.6% ± 4.2% versus 21.3% ± 5.4%, respectively) (Figures 1D and S2). Similarly, the percentage of p47^{phox}-expressing cells was consistently higher when a shorter IDLV *NCF1* construct was used at different MOIs (Figures 1E and S3). Due to the limited packaging capacity of AAV vectors and the size of the *NCF1-GFP* template (>3 kb), we only tested short AAV templates (AAV *GFP* and AAV *NCF1*) (Figures 1D, 1E, S2, and S3). The MOI that achieved the highest knock-in efficiencies (5×10^3 LPs/cell for IDLV templates and 2×10^6 vector genomes [vg]/cell for AAV templates) with no effect on viability of the edited PLB-985 *NCF1* ΔGT cells was chosen for subsequent experiments.

Transgenic p47^{phox} expression restores the NADPH oxidase function in the edited PLB-985 *NCF1* ΔGT cells

In p47-CGD, defective NADPH oxidase results in the production of ROS by phagocytes.[2,16] To investigate whether CRISPR-mediated knock-in of the *NCF1* cDNA at the *NCF2* locus restores ROS production, we performed functional tests on the edited PLB-985 *NCF1* ΔGT cells.

The knock-in of the *NCF1* cDNA at the *NCF2* locus restored p47^{phox} expression in 33.5% ± 5.3% and 32.2% ± 0.7% of granulocytic-differentiated cells when treated with RNP + AAV *NCF1* or IDLV *NCF1*, respectively ($n = 3$, Figure 1F). Since the transgenic p47^{phox} expression was controlled by the *NCF2* promoter, the level of p47^{phox} expression per cell was not expected to differ between AAV- or IDLV-mediated knock-in. Indeed, we found no significant difference in the relative p47^{phox}-positive median fluorescence intensity (MFI) between the two vector types (Figure S4E) and the level of the transgenic protein expression was sufficient to restore ROS production in the edited PLB-985 *NCF1* ΔGT cells after granulocytic differentiation (Figures 1G, 1H, and S4).

Transgenic p47^{phox} expression under the control of *NCF2* follows granulocytic differentiation of edited PLB-985 *NCF1* ΔGT cells

To investigate if transgenic p47^{phox} expression follows granulocytic differentiation as intended, we analyzed PLB-985 *NCF1* ΔGT cells edited with RNP + AAV *NCF1* for p67^{phox} and p47^{phox} expression. We confirmed that, upon knock-in of the *NCF1* cDNA at the *NCF2*, p47^{phox} expression was limited to the p67^{phox}-positive population (Figures 2A and S5A), indicating the desired regulation of the transgenic protein expression by the *NCF2* promoter.

To analyze the expression pattern of p47^{phox}, we generated single PLB-985 *NCF1* ΔGT clones that were knocked in with one or two

IDLV *NCF1-GFP*, IDLV *GFP*, or AAV *GFP* with increasing MOI. (E) Flow cytometry analysis of p47^{phox} expression in the CD11b-positive population upon granulocytic differentiation of PLB-985 *NCF1* ΔGT cells, treated with CRISPR-Cas9 RNP and IDLV *NCF1-GFP*, IDLV *NCF1*, or AAV *NCF1* with increasing MOI. MOI is denoted as LP/cell for IDLVs and vg/cell for AAVs. Gating strategies and relative MFI of (D) and (E) are shown in Figures S2 and S3, respectively; $n = 2–5$, data are shown as mean ± SD. Statistical analysis in (D) and (E) was performed with two-way ANOVA followed by Sidak's multiple comparisons test. (F) Flow cytometry analysis of p47^{phox} expression in the CD11b-positive population upon granulocytic differentiation of PLB-985 WT, non-treated (NT) PLB-985 *NCF1* ΔGT, or PLB-985 *NCF1* ΔGT treated with RNP + AAV *NCF1* (2e–6 vg/cell) or with RNP + IDLV *NCF1* (5e–3 LP/cell). Images are representative flow cytometry plots. (G) DHR test after granulocytic differentiation. Rhodamine 123 (Rho)-positive cells measured by flow cytometry upon stimulation with PMA of corresponding samples in (F). Fluorescent Rho indicates NADPH oxidase activity. For the data in (F) and (G) ($n = 3$), percent p47^{phox}-positive cells, percent Rho-positive cells, their respective relative MFI, and gating strategies are shown in Figure S4. (H) Representative light microscopy images of NBT test performed on granulocytic-differentiated cells from corresponding samples in (F) upon PMA stimulation. Scale bars, 50 μm. Blue formazan precipitates indicate ROS production. Quantification of formazan-positive cells is shown in Figure S4G.

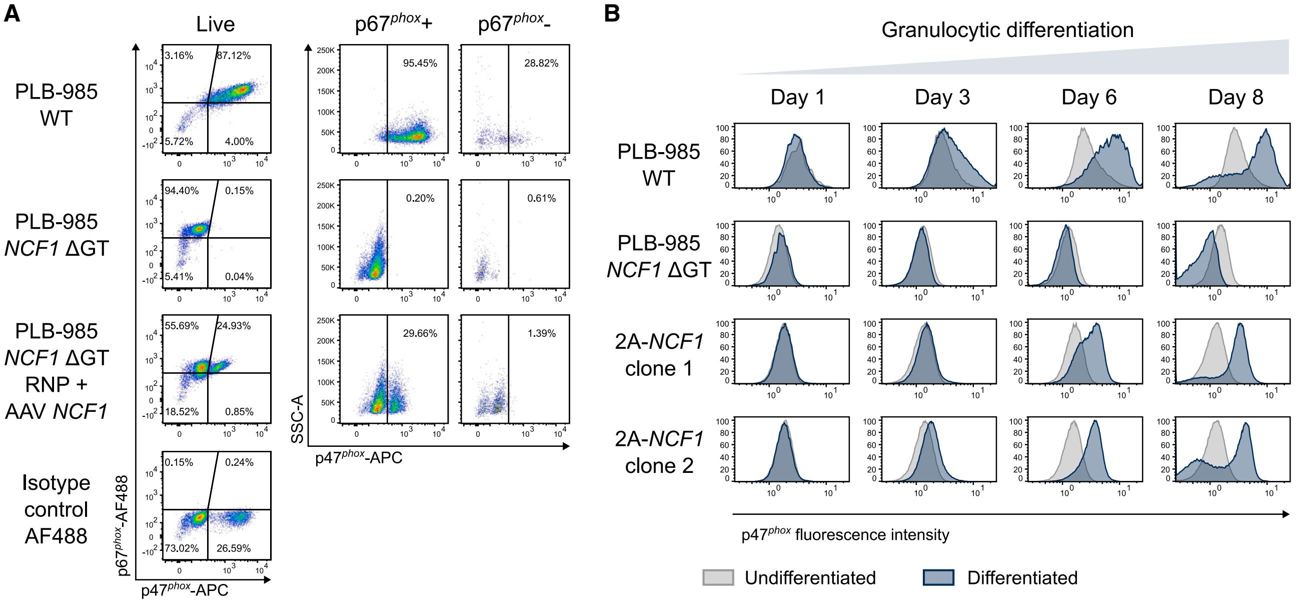

Figure 2. Transgene expression of p47*phox* follows myeloid differentiation and expression of the endogenous *NCF2* gene

(A) Flow cytometry analysis of p47*phox* and p67*phox* expression in total live cells upon granulocytic differentiation of PLB-985 WT, non-treated PLB-985 *NCF1* ΔGT, and PLB-985 *NCF1* ΔGT cells treated with RNP + AAV *NCF1*. The p47*phox* expression was subsequently determined by gating on the p67*phox*-positive and -negative populations. The absence of p47*phox* expression within the p67*phox*-negative population confirmed myeloid-determined expression of p47*phox* under the control of *NCF2* encoding p67*phox*; *n* = 3. Gating strategies and replicates are shown in Figure S5. (B) Histogram showing p47*phox* fluorescence intensity in PLB-985 WT, non-treated PLB-985 *NCF1* ΔGT, and two individual clones derived from PLB-985 *NCF1* ΔGT knocked in with 2A-*NCF1* vector over 8 days of granulocytic differentiation. Histograms of differentiated samples, overlaid with undifferentiated samples from respective bulk cultures, with relative cell count using modal y axis scaling.

copies of 2A-*NCF1* (clones 1 and 2, respectively) and monitored them for p47*phox* expression over 8 days of granulocytic differentiation. The 2A-*NCF1* knocked in clones exhibited a gradual increase of p47*phox* MFI upon differentiation (Figures 2B and S5B), showing that p47*phox* expression followed the physiological expression pattern of p67*phox*, which was driven by the endogenous *NCF2* promoter.

Minimal transgene expression is detected before *in vitro* myeloid differentiation of edited p47*phox*-deficient mouse HSPCs

To analyze the applicability of the editing approach to the actual target cells for gene therapy, we assessed the knock-in efficiency in p47*phox*-deficient lineage-negative mouse HSPCs (mHSPCs) using the *NCF1* template. As the editing of HSPCs with IDLV vectors did not result in sufficient levels of transgene-expressing cells (<2.4% knock-in efficiency in treated bulk cultures, Figure S6), we subsequently used AAV vectors for template delivery.

The knock-in protocol for HSPCs comprised electroporation of the Cas9 RNP targeting the mouse *Ncf2* in mHSPCs, followed by transduction of the AAV donor carrying the human *NCF1* cDNA (Figure 3A). Editing of p47*phox*-deficient mHSPCs restored p47*phox* expression in 56.3% ± 12.2% of myeloid-differentiated cells (Figure 3B), and subsequently the restoration of ROS production, as measured by the dihydrorhodamine-1,2,3 (DHR) test (32.9% ± 9.5% Rho^+ cells), and by the nitroblue tetrazolium (NBT) test (28.2% ± 2.7% formazan-positive cells) (Figures 3C, 3D, and S7A–S7D).

We confirmed that p47*phox* expression upon knock-in into the *Ncf2* gene was restricted to the myeloid-differentiated cells (Figures 3F and S7D), with minimal transgene expression detected before differentiation (0.6% ± 0.2% lineage-negative, p47*phox*-expressing cells) (Figures 3E and S7C). In contrast to the *Ncf2* promoter, knock-in at a housekeeping gene, β-2-microglobulin (*B2m*),[29] resulted in constitutive expression of p47*phox*, regardless of the differentiation status of the edited cells (Figures 3E and 3F).

Human HSPCs can be efficiently targeted for *NCF1* cDNA knock-in *in vitro*

Next, we treated human $CD34^+$ ($hCD34^+$) HSPCs isolated from healthy donors with an RNP + AAV *GFP* template, using the same protocol as for editing of mHSPCs. To investigate whether the knock-in treatment affects hematopoietic stem cell (HSC) stemness, we characterized the edited $hCD34^+$ cells using key stem cell surface markers (CD34, CD38, CD45RA, CD133/1) that define cells with high hematopoietic reconstitution capacity and high colony forming activity (Figures 4A and S8A).[30,31] We edited CD34-enriched cells and characterized them 2 days after editing *in vitro*. We found that AAV treatment reduced the percentage of $CD34^+$ $CD38^-$ $CD45RA^-$ cells by $1.61 \times (1.36)^{\pm 1}$ times that of non-treated

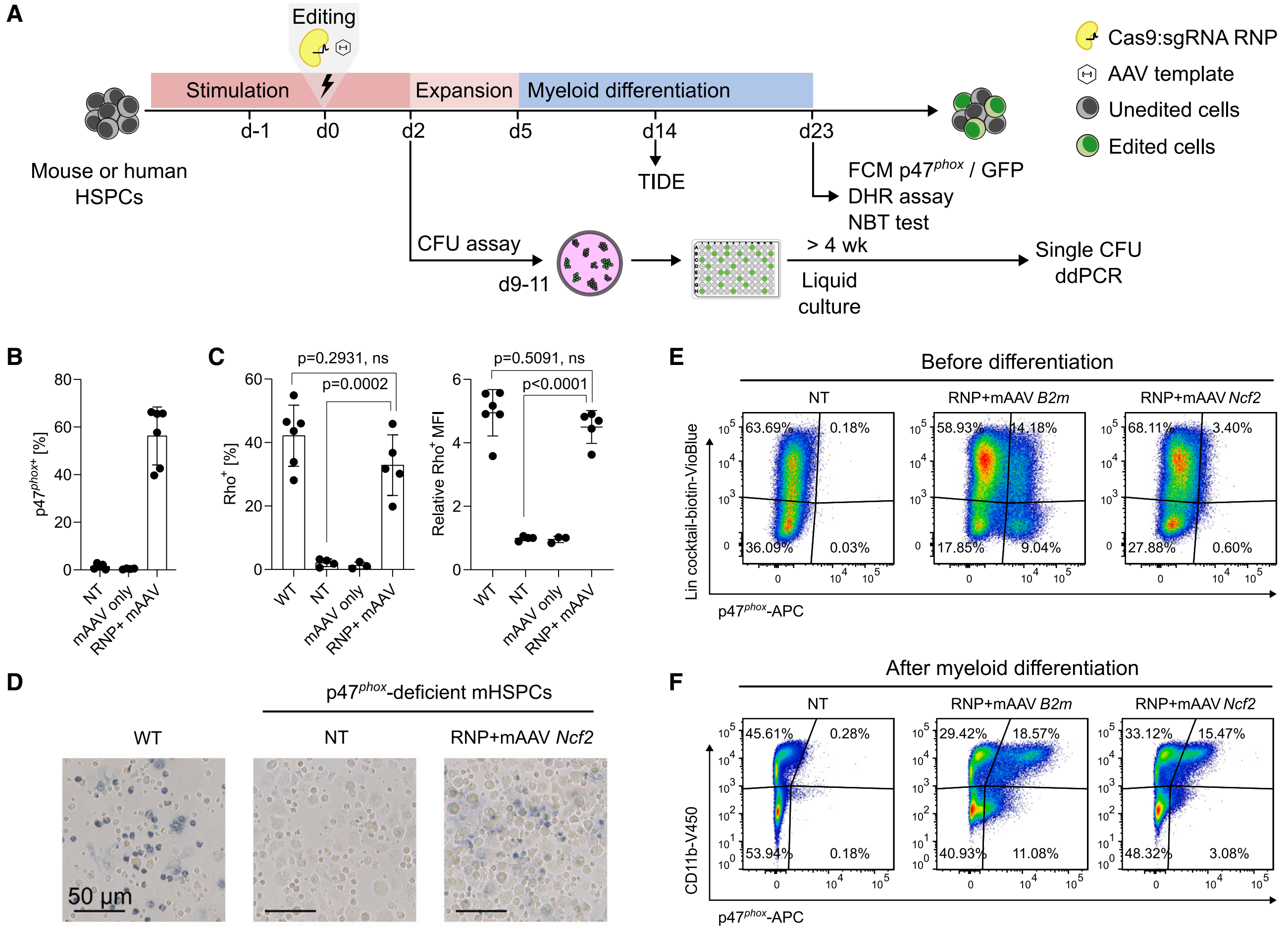

Figure 3. Myeloid-directed p47*phox* expression shown in editing of p47*phox*-deficient mouse HSPCs

(A) Experimental workflow of the knock-in strategy and analyses performed in mouse or human HSPCs. Mouse lineage-negative bone marrow HSPCs were derived from a p47*phox*-deficient mice. (B) Flow cytometry analysis of p47*phox* expression upon myeloid differentiation of non-treated (NT) p47*phox*-deficient mHSPCs, p47*phox*-deficient mHSPCs treated with mAAV only (AAV *NCF1* template with homology arms targeting the mouse *Ncf2* gene), or treated with RNP + mAAV. (C) DHR test upon myeloid differentiation of mHSPCs. Percentage of Rho-positive cells (left) and their respective relative MFI (right) measured by flow cytometry upon stimulation with PMA of WT mHSPCs (as positive control) and corresponding samples in (B); *n* = 3–6, data are shown as mean ± SD. Statistical analysis was performed with ordinary one-way ANOVA followed by Tukey's multiple comparisons test. (D) Representative light microscopy images from NBT test performed on myeloid-differentiated cells of NT, or RNP + mAAV *NCF1*-treated p47*phox*-deficient mHSPCs, and of WT mHSPCs upon PMA stimulation. Scale bars, 100 µm. Quantification of formazan-positive cells is shown in Figure S7E. (E) Flow cytometry analysis of p47*phox* expression, gated on the lineage-negative population before differentiation of NT cells, cells treated with RNP + mAAV *NCF1* knocked in into a constitutive gene, *B2m* (RNP + mAAV *B2m*) or into the myeloid-specific *Ncf2* locus (RNP + mAAV *Ncf2*). (F) Flow cytometry analysis of p47*phox* versus CD11b expression after myeloid differentiation of NT, RNP + mAAV *B2m*, or RNP + mAAV *Ncf2*-treated p47*phox*-deficient mHSPCs. Gating strategies and relative p47*phox*-positive or Rho-positive MFI in (B)–(F) are shown in Figure S7.

(NT) cells but did not significantly reduce the fraction of $CD34^+$ $CD38^-$ $CD45RA^-$ $CD133^+$ cells, which have been identified as highly enriched in long-term HSCs (Figures 4C and S8C). In addition, we performed a colony-forming unit (CFU) assay to evaluate the clonogenic potential of edited $hCD34^+$ cells (Figures 4B, left, and S8B). In general, AAV treatment decreased the clonogenic potential of edited $hCD34^+$ cells by $2.49 \times (1.22)^{\pm 1}$ times that of the NT control in the total number of CFUs, with significant decrease in number of burst-forming unit-erythroid (BFU-E) and colony-forming unit-granulocyte-macrophage (CFU-GM) ($2.18 \times (1.39)^{\pm 1}$ times BFU-E and $2.72 \times (1.00)^{\pm 1}$ times CFU-GM lower than that of NT), mainly due to the cytotoxic effect of AAV treatment, which was also evident in our *in vitro* cultures of $hCD34^+$ edited cells (Figure S8F). However, the clonogenic potential was retained upon serial CFU replating (Figure 4B, right), suggesting that this cytotoxic effect seems to mainly affect multipotent progenitors, rather than definite HSCs.

To determine the frequency of gene editing in $CD34^+$ $CD38^-$ and $CD34^+$ $CD38^+$ populations, we sorted $CD34^+$ $CD38^-$ and $CD34^+$

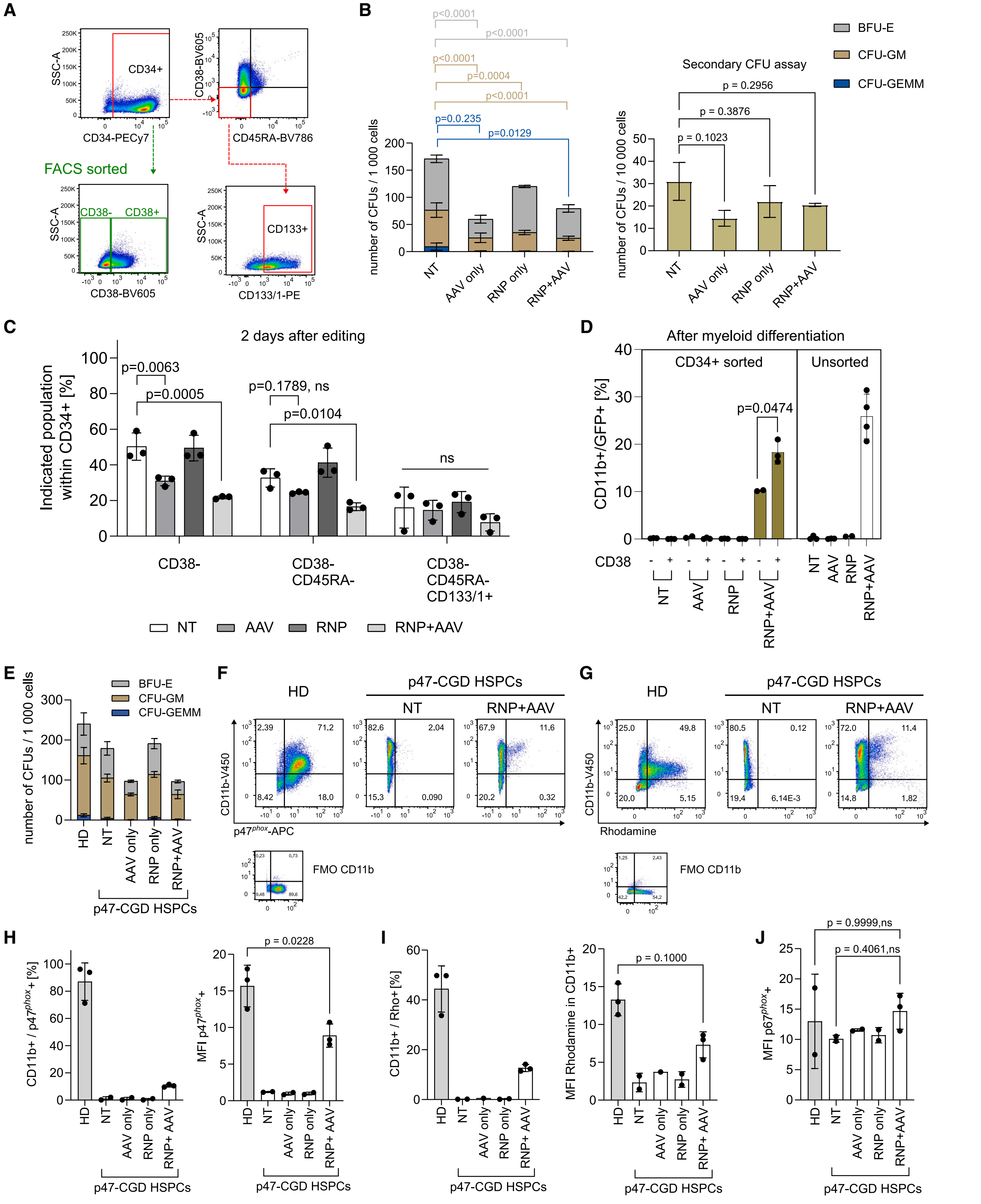

(legend on next page)

CD38^{+} subsets 2 days after editing, alongside unsorted CD34-enriched cells (Figure S8A). These cells were differentiated into promyeloid cells in liquid culture and checked for knock-in efficiency by measuring GFP expression. While in the unsorted population we achieved knock-in efficiency of 25.9% ± 4.7%, a significant percentage of *in-vitro*-differentiated myeloid cells derived from the CD34^{+} CD38^{-} sorted cells were also edited (10.2% ± 0.1% GFP^{+} cells, Figures 4D, S8G, and S8H), suggesting that the HSC-enriched CD38^{-} fraction of CD34^{+} cells were targeted.

Knock-in of *NCF1* cDNA at the *NCF2* locus functionally corrects p47-CGD patient HSPCs and does not affect endogenous p67^{phox} expression

We next evaluated the ability of our editing protocol (Figure 3A) to restore p47^{phox} expression and NADPH oxidase function in p47-CGD patient HSPCs. Bone marrow (BM) HSPCs derived from a p47-CGD patient were edited with an *NCF2*-targeted RNP + AAV *NCF1* donor template, and subsequently analyzed for clonogenic potential, p47^{phox} expression, and restoration of ROS production upon myeloid differentiation. Similar to editing in healthy HSPCs, AAV treatment reduced clonogenic potential in edited p47-CGD HSPCs (Figures 4E and S9E). We achieved 10.6% ± 1.0% of p47^{phox}-expressing cells upon knock-in, with p47^{phox} expression levels in the corrected cells being slightly lower than that of healthy HSPCs (MFI p47^{phox}-positive of 15.7 ± 2.8 in healthy versus 8.9 ± 1.6 in RNP + AAV *NCF1*; Figures 4F, 4H, and S9A). The achieved level of knock-in sufficiently restored NADPH oxidase function in 12.6% ± 1.4% cells (Figures 4G, 4I, and S9B). To evaluate the potential effect of knock-in at the *NCF2* locus, we analyzed for p67^{phox} expression in RNP + AAV *NCF1* p47-CGD bulk-treated HSPCs and found no reduction of p67^{phox} expression level (MFI p67^{phox}) upon knock-in, suggesting that the knock-in did not affect endogenous expression of the *NCF2* gene (Figures 4J and S9A).

Fusion proteins consisting of p67^{phox}-2A-p47^{phox} negatively impact the NADPH oxidase function

Post-transcriptional processing of the transgenic 2A-*NCF1* sequence knocked in into the *NCF2* gene should result in the production of separate p67^{phox} and p47^{phox} proteins through 2A-mediated ribosomal skipping.[27] In the case of an inefficient 2A activity, a fused translational product of p67^{phox}-2A-p47^{phox} might be produced, which could negatively affect the protein's function. In view of future clinical application, we analyzed the generation of unwanted p67^{phox}-2A-p47^{phox} fusion proteins and analyzed their potential impact on the NADPH oxidase function: we generated four heterozygous and four homozygous clones from PLB-985 *NCF1* ΔGT cells (denoted as "Fused hetero" or "Fused homo"), which were knocked in with an *NCF1* vector that carried a non-functional 2A oligopeptide (denoted as 2A*, carrying a mutation leading to a P17A amino acid change).[32] Clones that carried the mutated 2A*-*NCF1* vector produced fusion proteins of p67^{phox}-2A*-p47^{phox}, as confirmed by western blot, as opposed to the "Cleaved" clones that produced separate p67^{phox} and p47^{phox} proteins (Figure 5A). As expected, Fused hetero clones expressed both the fused and the cleaved p67^{phox} proteins, while Fused homo clones expressed only the fusion proteins (Figures 5A, 5B, and S10A–S10D). Comparing p67^{phox} and p47^{phox} expression in differentiated homozygous knocked in clones ("Cleaved homo") to PLB-985 wild-type (WT) cells, transgenic p47^{phox} expressed under the control of *NCF2* promoter were comparable with the endogenous p67^{phox} levels (Figure 5C), suggesting that the translation of p67^{phox}-2A-p47^{phox} proteins by 2A-mediated skipping was close to the theoretical expression ratio of 1:1 (ratio of p67^{phox}:p47^{phox} was 0.21 × $(2.06)^{\pm 1}$ in WT versus 0.55 × $(1.63)^{\pm 1}$ in Cleaved homo; n = 4 clones, three to four independent differentiation experiments; Figure 5C).

Results of the DHR test showed that fused p67^{phox}-2A-p47^{phox} affected the differentiated cells' ability to produce ROS compared with the cleaved counterparts (n = 16 clones, 0.15 × $(1.67)^{\pm 1}$ in Fused hetero and 0.16 × $(1.94)^{\pm 1}$ normalized MFI Rho in Fused homo versus 0.32 × $(2.00)^{\pm 1}$ in Cleaved hetero and 0.43 × (1.90) $^{\pm 1}$ normalized MFI Rho in Cleaved homo; Figures 5D, S11A, and S11B).

Nonetheless, we found no p67^{phox}-2A-p47^{phox} fusion proteins in bulk-treated PLB-985 *NCF1* ΔGT cells edited with either RNP + AAV or

Figure 4. Application of knock-in approach in healthy and p47-CGD human HSPCs

(A) Gating strategy for FACS sorting of CD38^{-}/$^{+}$ subpopulations and analysis of stem cell markers from hCD34^{+} HSPCs 2 days after editing. (B) CFU assay performed on non-treated (NT) healthy hCD34^{+} cells, treated with AAV *GFP* only, RNP only, or with RNP + AAV *GFP* (left graph). Secondary CFU assay was performed by replating 10 times the amount of cells from the same samples in the primary assay (right graph); n = 3 donors, with 2 technical replicates per donor; data are shown as mean ± SD; BFU-E, burst-forming unit-erythroid; CFU-G, colony-forming unit granulocyte; CFU-M, colony-forming unit macrophage; CFU-GEMM, colony-forming unit granulocyte, erythrocyte, monocyte, megakaryocyte; one-way ANOVA followed by Dunnett's multiple comparisons test for CFU-GM and BFU-E, and Kruskal-Wallis test with Dunn's multiple comparisons test for CFU-GEMM, to compare treated samples to NT only. Corresponding populations and significance values are indicated by corresponding fill and line colors, respectively. (C) Flow cytometry analysis of phenotypic HSC stemness markers on healthy control hCD34^{+} cells 2 days after editing; one-way ANOVA followed by Dunnett's multiple comparisons test. (D) Flow cytometry analysis of GFP expression in the CD11b-positive population upon myeloid differentiation of CD34^{+} CD38^{-}/$^{+}$ sorted or unsorted healthy control hCD34^{+} cells as shown in (B); paired t test. In (B)–(D), n = 3 donors; data are shown as mean ± SD. (E) CFU assay performed on NT p47-CGD hCD34^{+} cells, treated with AAV *NCF1*, RNP, and RNP + AAV *NCF1*, with healthy hCD34^{+} (healthy donor [HD]) as control; n = 1 donor with 2 replicates per donor, data are shown as mean ± SD. (F and G) Representative flow cytometry plots showing (F) p47^{phox} and CD11b expression and (G) DHR test showing rhodamine 123-positive cells and CD11b expression. (H and I) Flow cytometry analysis of (H) p47^{phox} expression in the CD11b-positive population and their respective MFI in (F) and (I) rhodamine-positive cells in the CD11b-positive population and their respective MFI in (G). (J) Flow cytometry analysis of MFI p67^{phox} of corresponding samples in (H). In (F)–(J), n = 1 donor with 2 to 3 technical replicates, data are shown as mean ± SD. Statistical analyses in (H) and (I) were performed with unpaired t test and in (J) with Brown-Forsythe and Welch ANOVA tests, followed by Dunnett's T3 multiple comparisons test. Gating strategies are shown in Figures S8 and S9.

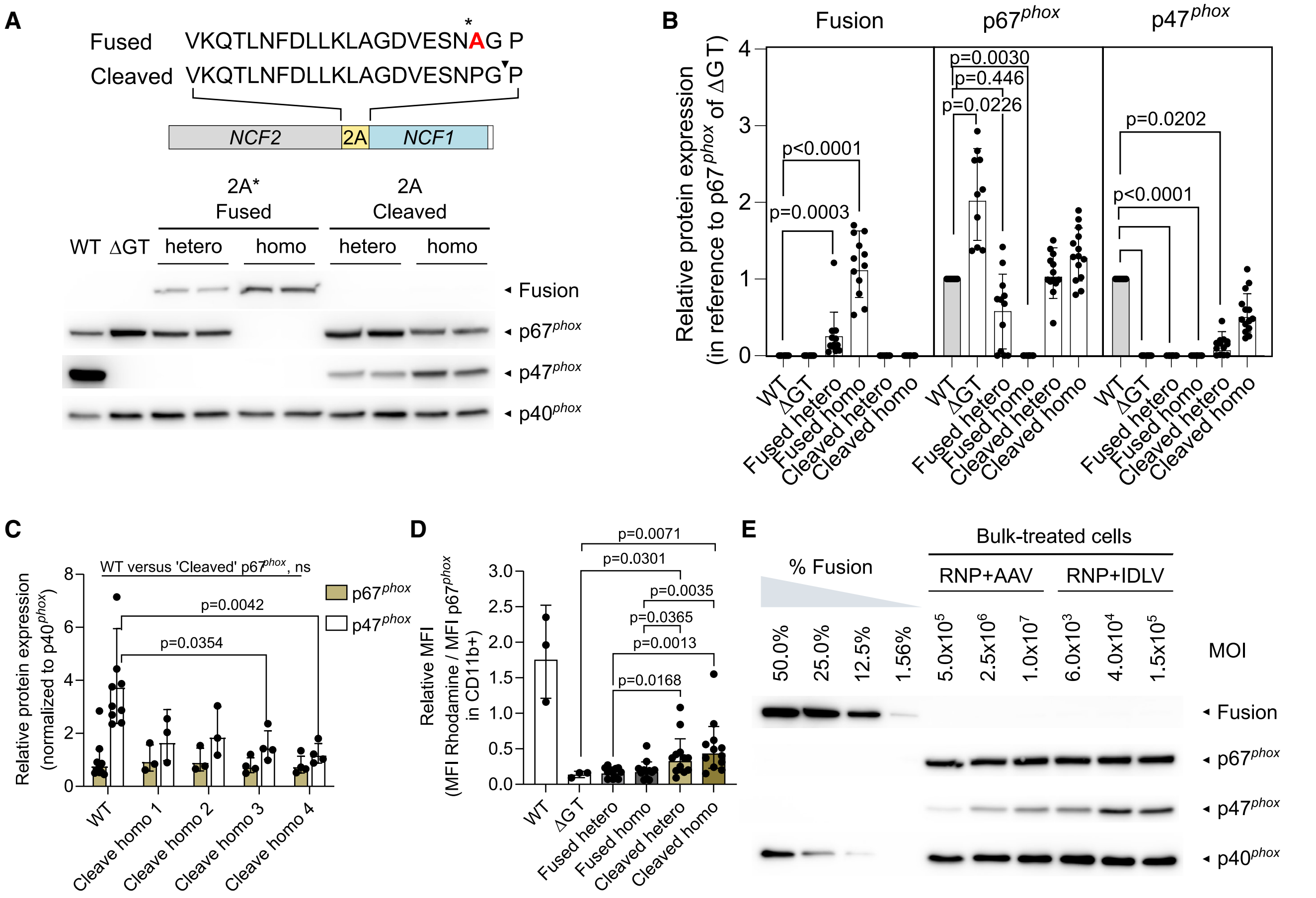

Figure 5. Analysis of potential fusion proteins caused by 2A oligopeptide activity upon *NCF1* knock-in

(A) Western blot detecting fusion protein p67*phox*-2A*-p47*phox*, p67*phox*, p47*phox*, and differentiation status loading control p40*phox* upon granulocytic differentiation of PLB-985 WT cells, non-treated PLB-985 *NCF1* ΔGT cells, representative clones derived from PLB-985 *NCF1* ΔGT cells heterozygous or homozygous knock-in of a mutated 2A* oligopeptide carrying *NCF1* cDNA (denoted as 2A* Fused, hetero or homo), and representative clones knocked in with WT 2A oligopeptide carrying *NCF1* cDNA (denoted as 2A Cleaved, hetero or homo). Schematic representation of the 2A sequence in the respective constructs used for generating "Fused" and "Cleaved" clones. The P17A mutation (*) introduced is highlighted in red. Arrow indicates 2A-mediated cleavage site between glycine-proline residue. (B) Relative protein expression of fusion p67*phox*-2A*-p47*phox*, p67*phox*, and p47*phox* quantified by western blots of samples in (A). Relative expression is represented in reference to PLB-985 WT after normalization with their respective differentiation status as indicated by p40*phox* expression; n = 3 to 4 differentiation experiment from 4 clones per group; Kruskal-Wallis test with Dunn's multiple comparisons test. (C) Protein expression of p67*phox* and p47*phox* quantified by western blot on PLB-985 WT and four PLB-985 *NCF1* ΔGT clones knocked in with 2 copies of *NCF1* cDNA; n = 4 clones with 3 to 4 differentiation experiments; Kruskal-Wallis test with Dunn's multiple comparisons test. (D) Relative MFI rhodamine 123 normalized to MFI p67*phox* expression in CD11b-positive cells from DHR test, performed on PLB-985 WT cells, non-treated PLB-985 *NCF1* ΔGT cells, heterozygous or homozygous Fused clones and Cleaved clones in (B); Kruskal-Wallis one-way ANOVA with two-stage step-up of Benjamin, Krieger and Yekutieli correction for multiple comparisons. Error bars for (B-D), data are shown as geometric mean ± geometric SD. (E) Western blot to analyze for presence of fusion proteins in bulk-treated PLB-985 *NCF1* ΔGT cells with RNP +AAV *NCF1* and RNP + IDLV *NCF1*. Cells were transduced with increasing MOI (vg/cell for AAVs and LP/cell for IDLVs). Protein expression was compared with a titration curve spiked with varying concentrations of fusion proteins. Unexposed and uncropped western blot images are shown in Figure S10. Representative flow cytometry plots and gating strategy are shown in Figure S11; data are shown as geometric mean × (geometric SD)$^{\pm 1}$.

RNP + IDLV *NCF1*, confirming efficient 2A cleavage ability of our construct (Figures 5E and S10E).

Knock-in treatment results in variable frequency of on- and off-target vector integrations and potential on-target deletions

The use of CRISPR-Cas9 and viral vectors poses the risks of off-target (OT) vector integrations, which can be mediated either by undesired non-homologous end joining (NHEJ), microhomology-mediated end joining (MMEJ), or by the inherent low-frequency integration of AAVs and IDLVs.[33–36] To identify vector integration events in the edited cells, we established a droplet digital PCR (ddPCR) approach to measure the copy number of 2A-*NCF1* vector, inverted terminal repeat (ITR) of AAV6, or the woodchuck hepatitis virus post-transcriptional regulatory element (wPRE) of IDLV (Figure 6A). With this method, we cannot determine if the integration took place at the on-target (*NCF2*) or OT sites, hence we refer to "unintended integrations" as any integration events that contained ITR/wPRE copies.

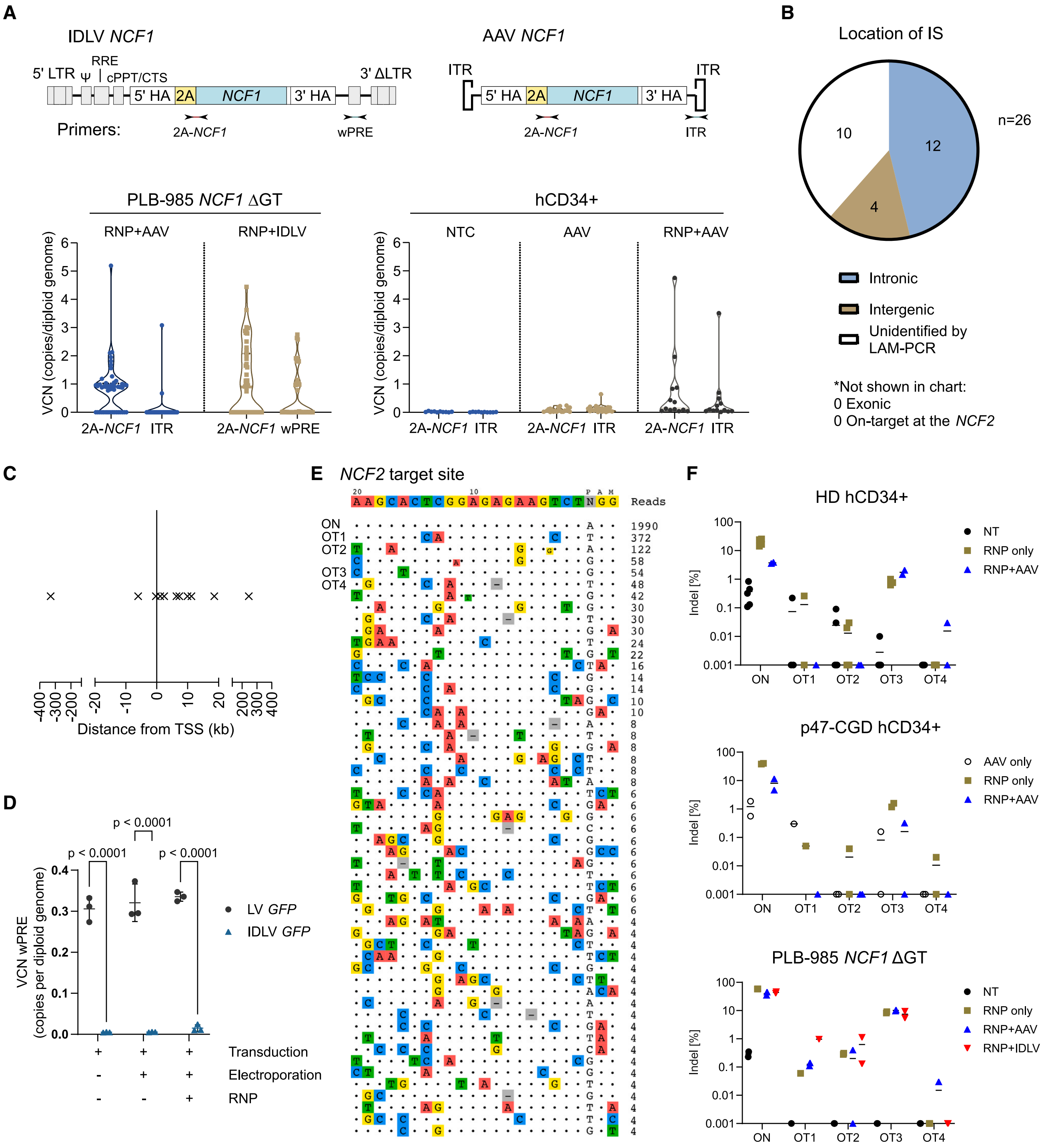

Figure 6. Assessment of unintended integrations and CRISPR off-targets upon knock-in treatment

(A) VCN analysis of 2A-*NCF1* and AAV ITR or IDLV wPRE, as measured by ddPCR. The location of ddPCR primers targeting the vector is depicted by inverted arrows. VCN analysis was performed on individual clones derived from PLB-985 *NCF1* ΔGT cells, treated with RNP + AAV *NCF1* or RNP + IDLV *NCF1* (left graph, n = 46 clones per condition), and individual clones derived from non-treated (NT) human CD34$^+$ cells, human CD34$^+$ cells treated with AAV *NCF1* only, or treated with RNP + AAV *NCF1* (right graph, n = 10–21 clones per condition). The VCN of each target was normalized to 2 copies of the *FOXP2* housekeeping gene as reference diploid genome. Each dot in the violin plot represents a data point for an individual clone. Additional information on VCN of 2A-*NCF1* versus ITR/wPRE, flow cytometry analysis of p47^{phox} expression and results of the DHR test corresponding to each individual clone are presented in Figure S11. Data are shown as mean ± SD. (B) Pie chart showing the number of integration

(legend continued on next page)

After editing, individual colonies were collected and grown in liquid culture for more than 4 weeks, and subsequently analyzed for vector copy number (VCN) (Figures 3A, 6A and S12). In PLB-985 *NCF1* ΔGT cells treated with RNP + AAV *NCF1* at MOI of 1×10^7 vg/cell, we identified one clone with five copies of 2A-*NCF1* and three copies of ITR (denoted as 5N/3I), and one clone with one copy of 2A-*NCF1* and one copy of ITR (denoted as 1N/1I), indicating the occurrence of OT integrations (2/48 clones contained ITR copies, Figures 6A and S12C). Compared with the treatment with RNP + IDLV *NCF1* at MOI of 6×10^3 LPs/cell, the OT events were markedly increased (14/24 clones contained wPRE copies, Figures 6A and S12D). The increase in number of clones containing wPRE copies could be associated with the increase in viral vector dose (MOI) (Figures S12D–S12F). In contrast to hCD34$^+$ cells treated with RNP + AAV *NCF1*, we only identified two clones with 5N/4I and 1N/1I copies (2/13 clones contained ITR copies, Figures 6A and S12G). Interestingly, one clone with 0N/1I was detected with the AAV-transduced only samples (without CRISPR treatment; 1/21 clones), indicating that random AAV integration was present even without the induction of DSBs by Cas9 (Figures 6A and S12G).

In addition, we analyzed for the presence of deletions ∼1.3 kb upstream and downstream surrounding the targeted 3′ end of the *NCF2* locus by copy number quantification using ddPCR in knocked in clones. Copy number losses were detected in four clones, with three clones containing deletions upstream and one clone downstream of the insertion site (n = 18, AAV clones 17, 23, 25, and 26, Figures S12C and S13). Indeed, these clones did not express p47^{phox} and were unable to produce ROS (AAV clones 23 and 25, Figure S12C).

Unintended integrations found in IDLV knocked in clones are due to the residual integrase activity of IDLVs

Although ddPCR VCN analysis can determine the frequency of unintended integrations, it does not provide information on their exact genomic locations. We therefore performed an integration site analysis by linear-amplification-mediated PCR (LAM-PCR)[37] on the clones that contained ITR or wPRE copies that were identified by ddPCR in Figure 6A (2 clones contained ITR sequences and 14 clones contained wPRE sequences). Out of 16 clones that contained ITR or wPRE sequences, we expected to capture 26 integration sites (ISs) by LAM-PCR, corresponding to the total number of vector copies per clone.

We characterized the identified ISs in AAV and IDLV knocked in clones based on their genomic location, distance from a transcriptional start site (TSS), and presence of potential sgRNA binding sites in a 200 bp window upstream and downstream of an IS (Figures 6B and 6C; Tables S5–S7). Most of the ISs were in intronic regions (12/26 ISs), while a few were in intergenic regions (Figure 6B). We neither found IS in exons, nor at the *NCF2* on-target site (on-target integrations not mediated by HDR) (Table S5). Most of the ISs were enriched within 10 kb downstream of TSS (mean distance from TSS = 9.9 kb, Figure 6C). This observed integration pattern resembled the integration signatures typical for the lentiviral vectors.[38,39] However, we confirmed the absence of integration-competent LV contamination in our IDLV preparation (Figures 6D and S15). As for AAV knocked in clones, no significant deductions can be made, because only two ITR-containing clones were identified and analyzed by LAM-PCR (clones 24 and 32, Figure S12C).

We further analyzed for CRISPR OT sites on the *NCF2* target gene by "circularization for high-throughput analysis of nuclease genome-wide effects by sequencing" (CHANGE-seq)[40] (Figure 6E) and verified the OT sites by targeted amplicon sequencing on edited healthy hCD34$^+$, p47-CGD hCD34$^+$, and PLB-985 cells (n = 2–6, all samples below 1% indels on all OTs, except 7.7% ± 1.9% indels on OT3 in edited PLB-985 cells; Figure 6F; Tables S9 and S10). None of the ISs identified by LAM-PCR matched the CRISPR OT sites determined by CHANGE-seq, suggesting that the observed integration events are unlikely due to OT mediated by Cas9 nuclease activity. This was also confirmed by *in silico* prediction using CRISPRoff, Cas-OFFinder, or manual blastn alignment (search was limited to 200 bp upstream and downstream of the IS; OTs were predicted based on the sgRNA *NCF2* sequence mismatches; Table S5).[41–43]

DISCUSSION

In this study, we demonstrated the successful application of a CRISPR-mediated *NCF1* cDNA knock-in into the myeloid-regulated *NCF2* gene to correct p47-CGD. We have previously shown that direct targeting of the predominant *NCF1* ΔGT mutation is not suited for gene therapy, due to the induction of frequent chromosomal deletions at this genomic region, comprising the *NCF1* gene and its pseudogenes that share a high sequence similarity.[19] Hence, we present an alternative approach that aims to avoid the induction of chromosomal deletions at the *NCF1* locus. Our approach has several advantages: the use of a promoter-less vector that lowers the risk of insertional activation in the case of OT integration.[44–47] Importantly, without a vector-derived promoter, a near-physiological expression pattern of p47^{phox} should be achieved under the regulation of the endogenous *NCF2* promoter.

Since HSPCs are the target for *ex-vivo*-based long-term correction of p47-CGD, we aimed to limit the expression of p47^{phox} transgenic

sites (ISs) by genomic location, as identified by LAM-PCR. The total number of ISs analyzed was based on the edited PLB-985 985 *NCF1* ΔGT clones that contain ITR or wPRE copies in (A) (n = 26 ISs predicted by ddPCR). (C) The distance from transcriptional start sites (TSSs) for individual ISs (n = 11 ISs that are within a gene). Each IS was depicted as a cross from the TSS at 0 kb. Detailed information of ISs is shown in Tables S5–S8. (D) VCN analysis of wPRE copies in PLB-985 *NCF1* ΔGT cells transduced with 5,000 lentiviral particles/cell of integration-competent LV *GFP* or IDLV *GFP*, compared with electroporated + transduced or electroporated with RNP + transduced samples. Data are shown as mean ± SD. (E) On- and off-target sites detected by CHANGE-seq for sgRNA *NCF2*. (F) The percentage of indels in edited hCD34$^+$ cells from healthy donor (HD), or p47-CGD patient hCD34$^+$ cells, and PLB-985 *NCF1* ΔGT cells confirmed by targeted amplicon deep sequencing at the on-target (ON) and four off-target (OT) sites detected by CHANGE-seq (Tables S9–S11); n = 2-6 biological replicates; data are shown as mean ± SD.

protein to the affected myeloid cells to avoid non-physiological activation of the NADPH oxidase and concomitant ROS production at the stem cell level. A balanced ROS level is crucial during hematopoiesis to support long-term repopulation ability of HSCs,[48,49] whereas oxidative stress caused by dysregulated ROS production can affect stem cell migration, development, proliferation, and repopulation potential.[50] By coupling the *NCF1* cDNA to the *NCF2* gene, the expression of p47^{phox} was closely linked to that of p67^{phox} following myeloid differentiation. However, this implies that the levels of p47^{phox} depend on the strength of the *NCF2* promoter, the overall knock-in efficiency, and the activities of homologous recombination (versus NHEJ, MMEJ, and inaccurate HDR).[33,47,51] With the achieved ~10% knock-in efficiency in p47-CGD patient HSPCs, we showed successful restoration of the NADPH oxidase activity *in vitro*. This level of correction should be sufficient for clinical correction, as in CGD only about 10%–20% of superoxide-producing cells were reported to be necessary for the reconstitution of host defense against infections.[52–54]

Apart from achieving a regulated expression of the therapeutic gene, correction of sufficient numbers of HSCs/LT-HSCs without impacting their stemness and engraftment potential is necessary for long-term treatment of CGD. Although sufficient gene correction levels of ~26% were achieved in healthy hCD34$^+$ cells, we observed reduced clonogenic potential and cell viability *in vitro*, especially after AAV treatment. Our data are in line with reports on the use of AAV6 for template delivery in HDR-based editing,[55–57] indicating that the observed genotoxicity may be due to activation of p53-mediated DNA damage response upon concomitant exposure of HSPCs to CRISPR-induced DSBs and prolonged persistence of AAV genomes (ITRs) in the edited cells.[58,59] We cannot judge on the potential toxic effects mediated by IDLV template delivery in HSCs as no correction was achieved with our construct. Another potential challenge with our approach could arise from the fact that the HDR mechanism specifically targets cycling cells (S/G2 phase), raising the possibility that a large fraction of dormant, long-term reconstituting HSCs will not be targeted. In our *in vitro* phenotypic analysis of edited hCD34$^+$ cells, we found that the HSC-enriched subset (CD34$^+$ CD38$^-$ CD45RA$^-$ CD133$^+$) remained unchanged, and the clonogenic potential is retained upon secondary CFU replating. Nonetheless, the ability of edited HSCs to engraft in a CGD mouse model should be investigated further by *in vivo* transplantation studies, which could provide more evidence on the clinical potential of our editing approach.

Inefficient 2A-mediated ribosomal skipping results in the production of fusion proteins, which may negatively impact the protein's function. Various forms of fused p67^{phox}-p47^{phox} proteins have been reported to result in a constitutively active, highly stabilized NADPH oxidase in *in vitro* cell-free systems.[60,61] Here, we showed that fusion proteins of p67^{phox}-2A*-p47^{phox} negatively affected ROS production in the edited cells. However, fusion proteins were undetectable with our current correction efficiency in bulk-treated cells.

As an additional safety assessment, we monitored the vector integration events by a ddPCR-based VCN determination method, LAM-PCR, and genome-wide unbiased CHANGE-seq. Random integrations of IDLVs and AAVs were reported to occur at relatively low levels (~0.1%–2.3% for IDLVs and 0.1%–1% for AAVs),[35,62–64] but were shown to be enhanced by nuclease-induced DSBs in the genome.[36,65] We detected minimal levels of unintended integrations in hCD34$^+$ and in PLB-985 *NCF1* ΔGT cells when knocked in with the AAV *NCF1* donor template compared with IDLVs. Interestingly, several knocked in clones that contained truncated 2A-*NCF1* copies or had deletions (~1.3 kb) surrounding the *NCF2* target site did not express p47^{phox} and did not produce ROS (5/92 clones), indicating that not all knock-in sequences constituted an in-frame seamless integration, and that on-target deletions cannot be avoided upon Cas9-mediated DSBs.[66,67] Since VCN quantification relies on the presence of the primer binding sites, not all forms of repair mechanisms (HDR, NHEJ, or MMEJ)[33] could be detected and distinguished. We also found that the unintended integrations were not restricted only to CRISPR-treated samples (samples without transduction with the viral vector template), suggesting that random AAV integrations are present and could be of potential concern.[68–71]

Using LAM-PCR on IDLV knocked in clones derived from the edited PLB-985 cells, the identified ISs resembled integration signatures of lentiviral vectors.[38,39] Whether the same ISs would also be retrieved in edited hCD34$^+$ cells was not investigated in this setting. Importantly, a near-diploid model cell line like PLB-985 has a reported 4% polyploidy[72] calls for copy-number variation and quantitative integration site analysis on the appropriate primary cells instead. We did not find any potential gRNA binding sites surrounding the ISs, thus could not establish a link between the observed integrations and the CRISPR-induced off targets.[36,58] From our observation, we confirmed that the unintended integrations were mediated by residual lentiviral integrase activity in the IDLVs[73] and not by CRISPR-induced OT DNA breaks or contamination from integration-competent LVs in the IDLV preparation. To address the observed CRISPR-induced OTs in the edited HSPCs, albeit at a low frequency (e.g., 0.32%–2.06% indels on OT3), the use of HiFi Cas9 for instance can improve the targeting specificity.[74]

Compared with IDLVs, our data showed that AAV template delivery resulted in fewer OT integrations and exhibited higher efficacy than using IDLV in HDR-based gene editing, as opposed to a recent report by Ferrari et al.[58] Differences in our editing protocol, i.e., longer HSPC stimulation period, use of the electroporation enhancer or transduction enhancer cyclosporin H, and the order of transduction and electroporation, may account for the different findings in our study. However, we cannot exclude the reported genotoxicity risks of ITR trapping in the edited HSPCs because of differences in the detection methods used by the group.

Recently, a knock-in approach based on cDNA insertion has been explored for the treatment of p47-CGD by Klatt et al.[15] In that study, a "minigene" insertion (cDNA comprising *NCF1* exons 2–11) was

targeted to a unique 3 bp polymorphism in the *NCF1* intron 1, preventing cleavage of the *NCF1* pseudogenes. This approach cannot be applied to all p47-CGD patients, as gene conversion in p47-CGD usually results in the transfer of large stretches of pseudogene sequence onto the *NCF1*.[75] As the crossover events between *NCF1* and its pseudogenes vary between patients, single-nucleotide polymorphisms are typically not shared between patients. Another potential approach to correct the predominant ΔGT deletion without the risks of Cas9-induced DSBs[19] is prime editing.[76] Prime editing presents a more streamlined correction procedure without viral vectors or targeting of other loci, but in common with base/prime editors still carries risks of chromosomal rearrangements,[77] potentially due to conversion of single-strand breaks into DSBs,[78] which was also observed in our attempt to correct the ΔGT deletion using Cas9 nickases (unpublished data). Nonetheless, our approach has the advantage of avoiding the complexity of editing at the highly homologous *NCF1* gene and the pseudogene loci. Importantly, knock-in of a full-length *NCF1* cDNA has the benefit of being applicable to treat all genetic forms of p47-CGD, not only the *NCF1* ΔGT mutation.

In conclusion, the CRISPR-based knock-in approach downstream of *NCF2* we propose has proven efficacious for the treatment of p47-CGD in relevant cellular models, with the transgene expression following myeloid differentiation. The editing approach also has the potential to be adapted for the treatment of other diseases where myeloid-directed expression is required. However, our findings on OT integrations and the associated genotoxicity when the viral template delivery is used emphasize the general need for a more comprehensive risk-benefit assessment for future clinical translation of nuclease- or AAV/IDLV vector-based therapies.

MATERIALS AND METHODS

Cell culture

The human myeloid leukemia cell lines, PLB-985 WT and PLB-985 *NCF1* ΔGT, were cultured in RPMI 1640 medium (PAN-Biotech, Germany) supplemented with 10% (v/v) fetal bovine serum (FBS) (PAN-Biotech, Germany), 10 mM HEPES, 100 U/mL penicillin, and 100 mg/mL streptomycin (Thermo Fisher Scientific, USA). PLB-985 cells are models of granulocyte-like cells, therefore *in vitro* differentiation of these cells was termed as “granulocytic differentiation.” For granulocytic differentiation, cells were plated at a density of 0.8×10^6 cells/mL in RPMI 1640 medium, supplemented with 5% (v/v) FBS, 0.5% (v/v) N,N-dimethylformamide (DMF) (Sigma-Aldrich, USA), 100 U/mL penicillin, and 100 mg/mL streptomycin for 7 days. Generation of individual clones were performed by fluorescence-activated cell sorting (FACS) with BD FACSAria III Cell Sorter (BD Biosciences, USA).

Human CD34$^+$ cells from mobilized peripheral blood of healthy donors were purchased from Lonza (Switzerland). The p47-CGD CD34$^+$ cells were obtained from a CGD donor under written informed consent. Sample collection and processing were performed in accordance with ethical principles, applicable local laws and regulations (ethics vote KEK ZH 2015/0135, BASEC-Nr. PB_2016-02202). CD34$^+$ cells were thawed and stimulated in X-VIVO 20 medium (Lonza, Switzerland), supplemented with 300 ng/mL recombinant human (rh) stem cell factor (SCF), 300 ng/mL rh FMS-like tyrosine kinase 3 ligand (Flt3-L), 100 ng/mL rh thrombopoietin (Sartorius CellGenix, Germany), and 1% (v/v) human serum albumin (HSA) (CSL Behring, Germany) for 1–2 days before electroporation and transduction procedures. For expansion and differentiation, cells were cultured in IMDM medium (Lonza, Switzerland), 10% (v/v) FBS, 50 U/mL penicillin, 50 mg/mL streptomycin, supplemented with 100 ng/mL rh SCF, 100 ng/mL rh IL-3 (Sartorius CellGenix, Germany), for 3 days, differentiated for 8 days with 100 ng/mL rh SCF and 10 ng/mL rh granulocyte colony-stimulating factor (G-CSF) (PeproTech, USA), and for another 9 days with only 10 ng/mL G-CSF. Differentiation of hCD34$^+$ toward myeloid lineages was termed myeloid differentiation, in contrast to granulocytic differentiation of PLB-985 cells.

Lineage-negative BM mHSPCs from p47-deficient mice or WT mice were cultured for 1–2 days in the medium of the same composition as described for hCD34$^+$ cells, but with recombinant murine (rm) cytokines (PeproTech, USA). For expansion and myeloid differentiation, the same medium composition was used, except with rm cytokines for a 3-day expansion, 4-day differentiation with rm SCF, rm IL-3, and rm G-CSF, followed by 4-day differentiation with only G-CSF.

All cell cultures were kept at 37°C in a humidified CO_2 incubator with 5% CO_2.

Isolation of BM-derived mHSPCs

WT B6 mice (strain C57BL/6J, JAX: 000664) and p47$^{phox-/-}$ (strain B6(Cg)-*Ncf1*^{m1J}/J, JAX: 004742) mice were maintained at the Laboratory Animal Services Center (LASC), University of Zurich, Schlieren, Switzerland. The animals were housed under specific pathogen-free conditions with unlimited access to water and food. All procedures were conducted in accordance with the Swiss legislation on animal protection and welfare (Tierschutzverordnung, TSchV 455.163/Tierschutzgesetz, TSchG 455) and were approved by the Cantonal Veterinary Office (VETA) and the Cantonal Commission for Animal Experimentation (TVK) in Zurich (license no. ZH084/2019). All mice were physically examined and acclimatized for a minimum of 7 days, followed by euthanasia with CO_2 inhalation. BM cells were obtained from femur, tibia, humerus, and pelvis. The excised bones were crushed with a mortar and a pestle in PBS (Thermo Fisher Scientific, USA) supplemented with 1% (v/v) HSA, 100 U/mL penicillin, and 100 mg/mL streptomycin. The BM cell suspension was then filtered through a 70- and 40-μm cell strainer (LubioScience, Switzerland), and subjected to lineage depletion to obtain Lin$^-$ cells using Direct Lineage Cell Depletion Kit (Miltenyi Biotec, Germany) according to the manufacturer’s instructions.

Vector design

Repair template 2A-*NCF1* and control template 2A-*GFP* were synthesized by GeneArt Subcloning and Plasmid services (Thermo Fisher Scientific, USA) with 2A peptide sequence from FMDV

X00871; nts 3,483–3,475, NCF1 from NM_000265.5; nts 74–1,240 (codon-optimized), and enhanced GFP (eGFP) from pcDNA3-EGFP. The plasmid pcDNA3-EGFP was provided by Doug Golenbock (Addgene, plasmid no. 13031; http://n2t.net/addgene:13031). Homology arms with 501 bp each were PCR amplified from PLB-985 genomic DNA (gDNA), flanking the human *NCF2* NG_007267.1 or mouse *B2M* NC_000068.8 upstream and downstream of the transcription stop codon (TAG). IDLV constructs were constructed by inserting the corresponding transgene into the XhoI and XmaI sites on self-inactivating (SIN) SIN HIV-1 lentiviral plasmid backbone by Gibson assembly (New England Biolabs [NEB], USA). AAV6 vectors were generated by cloning the transgene (sequence shown in Table S9) into the KpnI and SalI sites on the p24 plasmid (Repository ID: p24, pssAAV-2-CAG-EGFP-WPRE-SV40p(A), Viral Vector Facility, University of Zurich) by Gibson assembly.

Viral vector production

AAV and IDLV vectors were produced by the Viral Vector Facility (VVF) of the Neuroscience Center Zurich (ZNZ). To produce IDLVs, HEK293T cells were transfected with transfer plasmid containing donor templates as described above, *gag-pol* packaging plasmid pSLQ8001-pCMV-R8.91 (D64V) provided by Stanley Qi (Addgene, plasmid no. 202687; http://n2t.net/addgene:202687; RRID:Addgene_202687) and VSV-G envelop plasmid pMD2.G provided by Didier Trono (Addgene, plasmid no. 12259; http://n2t.net/addgene:12259; RRID:Addgene_12259). Viral vector supernatant was precipitated by PEG6000 and resuspended in sterile PBS (pH 7.4). The viral titer was quantified by QuickTiter Lentivirus Titer Kit (HIV p24) ELISA kit (Cell Biolabs, USA) and expressed as LPs/mL.

To produce single-stranded AAVs, HEK293T cells were transfected with AAV helper plasmid serotype 6 (cap ORF: AF028704.1), adenovirus helper plasmid pBS-E2A-VA-E4,[79] and transfer plasmid containing donor templates as described above. Viral vector supernatant was purified by OptiPrep density gradient ultracentrifugation and diafiltration, followed by resuspension in PBS (pH 7.4), 1 mM $MgCl_2$, 2.5 mM KCl. The viral titer was quantified by fluorometric method using a Qubit dsDNA HS kit (Thermo Fisher Scientific, USA) and expressed as vg/mL.

sgRNA design

The *Streptococcus pyogenes* Cas9 (SpCas9) sgRNAs were designed using the CHOPCHOP v.3 web tool[80] (https://chopchop.cbu.uib.no/) and purchased from Integrated DNA Technologies (Alt-R CRISPR-Cas9 sgRNA).

Cas9 nuclease preparation

The *S. pyogenes* (*Sp*)Cas9 expression vector (Addgene, no. 78312; pMJ922 from Martin Jinek; http://n2t.net/addgene:78312)[81] encodes a Cas9 fusion protein with an N-terminal hexahistidine-maltose binding protein tag (6xHis-MBP) followed by a tobacco etch virus (TEV) protease cleavage site and a C-terminal hemagglutinin (HA) tag, GFP tag, and three nuclear localization signals (NLSs) yielding a 6xHis-MBP-TEV-Cas9-HA-2xNLS-GFP-NLS construct. Purification of Cas9 was carried out as described,[82,83] with minor adjustments. In brief, Cas9 construct was expressed in *E. coli* BL21 Rosetta2 (DE3) cells (Merck KgaA, Germany). Cells were lysed in 20 mM Tris (pH 8.0), 500 mM NaCl, 5 mM imidazole, 1 μg/mL pepstatin, 200 μg/mL AEBSF by ultrasonication. Clarified lysate was applied to a 10 mL Ni-NTA (Sigma-Aldrich, USA) affinity column. The column was washed with 20 mM Tris (pH 8.0), 500 mM NaCl, and 10 mM imidazole, and bound protein was eluted by increasing imidazole concentration to 250 mM. Eluted protein was dialyzed against 20 mM HEPES (pH 7.5), 250 mM KCl, 10% glycerol, 1 mM dithiothreitol (DTT), and 1 mM EDTA overnight at 4°C in the presence of TEV protease to remove the 6xHis-MBP affinity tag. Cleaved protein was further purified using a HiTrap HP Heparin column (GE HealthCare, USA), eluting with a linear gradient to 1.0 M KCl. Elution fractions were pooled, concentrated, and further purified by size-exclusion chromatography using a Superdex 200 (16/600) column (GE HealthCare, USA) in 20 mM HEPES-KOH (pH 7.5), 500 mM KCl, and 01 mM DTT, yielding pure, monodisperse proteins. Aliquots were flash-frozen in liquid nitrogen and stored at −80°C.

CRISPR-Cas RNP electroporation and donor template delivery

For electroporation of $hCD34^+$ cells, 18 pmol of SpCas9-GFP protein and 36 pmol of sgRNA were incubated for 15 min at room temperature to form an RNP complex. A total of 1×10^5 cells was combined with RNP complex in 10 μL Resuspension Buffer T, and subjected to electroporation at 1,400 V, 10 ms, 3 pulses using the Neon Transfection System 10 μL kit (Thermo Fisher Scientific, USA). Within 30 min after electroporation, cells were transduced with IDLV or AAV donor at the indicated MOI in LPs/cell or vg/cell, respectively, and incubated in a CO_2 incubator at 37°C 5% CO_2 for 2 h, followed by 80% (v/v) medium change. For electroporation of PLB-985 cells, 2×10^5 cells were combined with RNP complex in Resuspension Buffer R and subjected to electroporation at 1,150 V, 10 ms, 3 pulses. For electroporation of mHSPCs, 2×10^5 cells were combined with RNP complex in Resuspension Buffer T and subjected to electroporation at 1,700 V, 20 ms, 1 pulse.

TIDE analysis

For tracking of indels by decomposition (TIDE) analysis,[84] the target sequence was amplified using Phusion High Fidelity DNA Polymerase (primers are shown in Table S3). PCR products were purified using QIAquick PCR Purification Kit (QIAGEN, Germany), analyzed by 1% agarose gel electrophoresis, and sent for Sanger sequencing (Microsynth, Switzerland). Sequence traces from control and treated samples were analyzed using the TIDE web tool (https://tide.deskgen.com/).

VCN analysis by ddPCR

The VCN in single clones was determined by ddPCR using a QX200 Droplet Digital PCR System (Bio-Rad Laboratories, USA). Genomic DNA was digested with DraI (20 U/μL) (NEB, USA) for 1 h at

37°C followed by heat inactivation for 20 min at 65°C. 3–4 ng/μL of DraI-digested gDNA was used per ddPCR reaction, containing ddPCR supermix for robes (no dUTP) (Bio-Rad Laboratories, USA), 900 nM primers, and 250 nM probes. A total of 20 μL of sample reaction was transferred to a DG8 Cartridge for the QX200/QX100 Droplet Generator and processed according to the manufacturer's instructions. Droplets containing DNA samples were first denatured for 10 min at 95°C, and then subjected to 40 cycles of amplification comprising consecutive steps of denaturation (95°C, 30 s), annealing (60°C, 1 min), and extension (72°C, 1 min), with a final 10 min incubation at 95°C. Following amplification, samples were analyzed using QX200 Droplet Reader and QuantaSoft Analysis Pro software v.1.0 (Bio-Rad Laboratories, USA). The values of the VCN per cell were quantified with reference to a housekeeping gene, *FOXP2.* Primers used are shown in Table S2.

Flow cytometry

Flow cytometry analysis was performed using a BD LSRFortessa (BD Biosciences, USA) or a MACSQuant Analyser 10 flow cytometer (Miltenyi Biotec, Germany). Extracellular staining was performed using anti-CD11b, anti-CD14, and anti-CD15 for myeloid cells, or anti-CD34, anti-CD38, anti-CD45RA, and anti-CD133/1 antibodies for HSCs and a viability dye after incubation with an FcR blocking reagent (Miltenyi Biotec, Germany), followed by fixation with 1% (v/v) paraformaldehyde (Sigma-Aldrich, USA). Cells were permeabilized with 0.1% saponin (Sigma-Aldrich, USA) and stained intracellularly with anti-p47^{phox} and anti-p67^{phox} antibody. Endogenous GFP expressions were analyzed *in situ*. In mHSPCs, LSK cells were stained by lineage cell detection cocktail conjugated with biotin, anti-c-Kit, and anti-Sca-1, followed by secondary staining with anti-biotin (Miltenyi Biotec, Germany). Fluorescence minus one controls and isotype controls were used when appropriate (antibodies, dyes, and reagents are listed in Table S4).

For DHR test, CD11b-labeled cells were incubated with 10 μM DHR 123 (Sigma-Aldrich, USA) and 3,450 U/mL catalase from bovine liver (Sigma-Aldrich, USA) in PBS containing 0.05% (w/v) gelatin from cold water fish skin (Sigma-Aldrich, USA) and 0.9 g/L glucose (Thermo Fisher Scientific, USA) for 15 min at 37°C in 5% CO_2, followed by stimulation with 1 μg/mL phorbol 12-myristate 13-acetate (PMA) (Sigma-Aldrich, USA) for 15 min at 37°C in 5% CO_2 before measurement.

Western blot

Total proteins were isolated from whole cells by protein lysis using M-PER Mammalian Protein Extraction Reagent (Thermo Fisher Scientific, USA). Upon centrifugation for 30 min at 16,000 × *g* and at 4°C, protein supernatant was collected, and concentration was measured by Bradford assay (Sigma-Aldrich, USA). Protein supernatant was resuspended in 4× Laemmli sample buffer (Bio-Rad Laboratories, USA) with 1:100 (w/v) β-mercaptoethanol (Sigma-Aldrich, USA). Prior to SDS-PAGE, protein samples in sample buffer were then denatured at 95°C for 5 min and cooled for 10 min at room temperature. A total of 80 μg proteins per sample were loaded on a 4%–12% polyacrylamide gel by SDS-PAGE using Mini-PROTEAN Tetra Cell electrophoresis system, followed by wet transfer overnight at 4°C onto a 0.2 μm PVDF membrane using Mini Trans-Blot Cell transfer system (Bio-Rad Laboratories, USA). The membranes were blocked with 5% skimmed milk and immune-stained using primary antibodies against p47^{phox}, p67^{phox}, and p40^{phox} in 5% skimmed milk overnight at 4°C, followed by secondary staining with anti-mouse conjugated to horseradish peroxidase. The signal was developed by incubation of the membrane in SuperSignal West Pico PLUS Chemiluminescent Substrate (Thermo Fisher Scientific, USA), visualized with Amersham ImageQuant 800 (Cytiva, USA). Band intensities were subjected to densitometric analysis using ImageJ software (National Institutes of Health, USA).[85] To determine the expression of myeloid-dependent p67^{phox} and p47^{phox} proteins, their band intensities were first normalized to p40^{phox} as a differentiation marker (DM), by protein of interest (PI)/DM. To ensure quantification of band intensities are comparable across different blots, WT protein sample was loaded on every blot as an internal control reference. The WT samples were differentiated alongside treated samples of the same replicate and loaded on its respective blot. A second normalization was then performed by (PI/DM) of sample/(PI/DM) of WT to determine the relative protein expression.

NBT

A total of 1 × 10^5 cells of differentiated PLB-985 cells were plated on a 96-well plate and were incubated with 100 μg/mL PMA (Sigma-Aldrich) and 200 ng/mL NBT for 30 min at 37°C and 5% CO_2, followed by cell fixation in 1% (w/v) formaldehyde. Fixed cells were analyzed visually for the presence of formazan precipitates using a Leica DM IL Fluo light microscope, equipped with a DFC420 digital camera and Leica application suite acquisition software (Leica Microsystems, Germany).

Hematopoietic CFU assay

A total of 1,000 hCD34$^+$ cells was plated in MethoCult H4434 Classic (STEMCELL Technologies, Germany) in a 35-mm cell culture dish and incubated at 37°C in a humidified CO_2 incubator with 5% CO_2 for 10–14 days. Secondary replating was performed with a total of 10,000 cells collected from the primary CFU plates, pelleted, washed twice in X-VIVO 20 medium, and replated with the same conditions described above. The identity of formed CFUs was determined visually using a Leica EZ4 Stereo Microscope (Leica Microsystems, Germany). Individual CFUs were counted, and collected for gDNA lysis in Buffer K. The data were represented as a total CFU number and a frequency of each CFU type.

LAM-PCR

A total of 300 ng gDNA was used to perform linear amplification of vector-genome junctions using biotinylated primers LTR I or ITR I. The pre-amplified DNA-bead complex was separated by magnetic beads using the Dynabeads kilobaseBINDER kit (Thermo Fisher Scientific, USA) according to the manufacturer's instructions, followed by dsDNA synthesis using random hexanucleotide as primers and Klenow polymerase (NEB, USA). After two washing steps with

DNase-free water, the DNA-bead complex was digested with TasI enzyme and ligated with a linker cassette for 1 h at room temperature. After two washing steps with DNase-free water, the linker-ligated fragments were released from magnetic beads by denaturation using 0.1 N sodium hydroxide solution for 5 min at room temperature. The released ssDNA fragments were then used as a template for the first nested PCR, followed by a SacI digest of the internal control and the second nested PCR. The protocol was adapted from Schmidt et al.[37] and primers used in each step are listed in Table S6. Amplicons from the second nested PCR were subcloned into the pCR Blunt II-TOPO vector using Zero Blunt TOPO PCR Cloning Kit (Thermo Fisher Scientific, USA), and sequences were identified by Sanger sequencing (additional methods used downstream of LAM-PCR are described in the supplemental information).

CHANGE-seq

The sgRNA *NCF2* was first tested for functionality by digesting a PCR amplicon of the genomic target site *in vitro*. NT and Cas9 sgRNA *NCF2* RNP-treated human $CD34^+$ cells from healthy donors (three technical replicates per sample) were used for this method. The CHANGE-seq experiment was performed by Functional Genomics Center Zurich (FGCZ). Next-generation sequencing data were processed using the CHANGE-seq analysis pipeline (https://github.com/tsailabSJ/changeseq) with the following parameters: read_threshold, 4; window_size, 3; mapq_threshold, 50; start_threshold, 1; gap_threshold, 3; mismatch_threshold, 10; search_radius, 30; and merged_analysis, True. Raw data of the CHANGE-seq results is provided in Table S11. Next-generation sequencing (NGS) sequencing data are deposited at NCBI Sequence Read Archive (NCBI BioProject: PRJNA1085720).

Indel analysis by NGS

Four selected OT sites were deep sequenced (detailed number of reads, NGS data summary information, and primer sequences are provided in Tables S9 and S10). Amplicons for deep sequencing were generated using two rounds of PCR to attach Illumina handles. Q5 high-fidelity polymerase and 150 ng of gDNA in a total volume of 15 μL were used. The thermal cycling profile of the PCR was: 98°C 30 s; 25 × (98°C 30 s; annealing [see Table S9] 30 s; 72°C 30 s); 72°C 5 min. i5 and i7 Illumina adapters were added in a second PCR reaction using Q5 high-fidelity polymerase (NEB, USA) and 1 μL of a first-step PCR product in a total volume of 15 μL. The thermal cycling profile of the PCR was: 98°C 30 s; 15 × (98°C 30 s, 72°C 1 min); 72°C 5 min. Approximately equal amounts of PCR products from each sample were pooled, gel purified, quantified using a Qubit fluorometer with a dsDNA HS Assay Kit (Thermo Fisher Scientific, USA) and were 2 × 150 bp paired-end sequenced on MiSeq (Illumina, USA). NGS amplicon sequencing data are deposited at NCBI Sequence Read Archive (NCBI BioProject: PRJNA1085720).

Statistical analysis

Statistical analysis was performed using GraphPad Prism v.8.4.3 for Windows, GraphPad Software, www.graphpad.com. Parametric tests were used for group-wise comparisons if data assumed normal distribution when tested by QQ plot, otherwise non-parametric tests were used. Interpretations for ratios comparing treated and control samples, i.e., in Figures 5B–5D, were represented in geometric mean × (geometric SD)$^{\pm 1}$. Each specific statistical test is indicated in the figure legends.

DATA AND CODE AVAILABILITY

The data supporting the findings of this study are available in the supplemental information and additional details upon request from the corresponding author, Janine Reichenbach.

SUPPLEMENTAL INFORMATION

Supplemental information can be found online at https://doi.org/10.1016/j.omtn.2024.102229.

ACKNOWLEDGMENTS

This study was supported by the Clinical Research Priority Program (CRPP) ImmuGene, University of Zurich (UZH), which has funded K.M.S., O.P., F.R., and D.W. (awarded to J.R. and M.J.); the University Research Priority Program Innovative Therapies in Rare Diseases (URPP ITINERARE) (awarded to J.R. and G.S.), UZH; the University Research Priority Program Human Reproduction Reloaded (URPP H2R) (awarded to P.I.K.); and the Swiss National Science Foundation (SNSF) grant 31003A_182567 (awarded to M.J.). We thank all members of J.R.'s laboratory for discussion and technical help, as well as the UZH Cytometry Facility for FACS operator-based cell sort, and the UZH Viral Vector Facility (VVF) of the Neuroscience Center Zurich (ZNZ) for viral vector production service.

AUTHOR CONTRIBUTIONS

K.M.S., M.G., D.W., F.R., O.P., P.T., P.I.K., M.J., U.M., U.S., and J.R. designed the experiments, analyzed the results, and wrote the manuscript. K.M.S., M.G., T.M., and P.I.K. performed the experiments. O.P. and P.T. provided technical help with mouse experiments. K.M.S., M.G., T.M., and P.I.K. prepared the figures. M.S. produced reagents used in the study. M.J and G.S. provided technical expertise related to design of CRISPR approaches and off-target analyses.

DECLARATION OF INTERESTS

The authors declare no competing interests.

REFERENCES

1. Segal, B.H., Leto, T.L., Gallin, J.I., Malech, H.L., and Holland, S.M. (2000). Genetic, Biochemical, and Clinical Features of Chronic Granulomatous Disease. Medicine *79*, 170–200. https://doi.org/10.1097/00005792-200005000-00004. http://journals.lww.com/00005792-200005000-00004.
2. Roos, D. (2019). Chronic Granulomatous Disease. Methods Mol. Biol. *1982*, 531–542. https://doi.org/10.1007/978-1-4939-9424-3_32. http://link.springer.com/10.1007/978-1-4939-9424-3_32.
3. Bianchi, M., Hakkim, A., Brinkmann, V., Siler, U., Seger, R.A., Zychlinsky, A., and Reichenbach, J. (2009). Restoration of NET formation by gene therapy in CGD controls aspergillosis. Blood *114*, 2619–2622. https://doi.org/10.1182/blood-2009-05-221606. https://ashpublications.org/blood/article/114/13/2619/26364/Restoration-of-NET-formation-by-gene-therapy-in.
4. Meissner, F., Seger, R.A., Moshous, D., Fischer, A., Reichenbach, J., and Zychlinsky, A. (2010). Inflammasome activation in NADPH oxidase defective mononuclear

phagocytes from patients with chronic granulomatous disease. Blood *116*, 1570–1573. https://doi.org/10.1182/blood-2010-01-264218. https://ashpublications.org/blood/article/116/9/1570/103869/Inflammasome-activation-in-NADPH-oxidase-defective.

5. Ott, M.G., Schmidt, M., Schwarzwaelder, K., Stein, S., Siler, U., Koehl, U., Glimm, H., Kühlcke, K., Schilz, A., Kunkel, H., et al. (2006). Correction of X-linked chronic granulomatous disease by gene therapy, augmented by insertional activation of MDS1-EVI1, PRDM16 or SETBP1. Nat. Med. *12*, 401–409. https://doi.org/10.1038/nm1393.

6. Siler, U., Paruzynski, A., Holtgreve-Grez, H., Kuzmenko, E., Koehl, U., Renner, E.D., Alhan, C., de Loosdrecht, A.A.v., Schwäble, J., Pfluger, T., et al. (2015). Successful Combination of Sequential Gene Therapy and Rescue Allo-HSCT in Two Children with X-CGD - Importance of Timing. Curr. Gene Ther. *15*, 416–427. https://doi.org/10.2174/1566523215666150515145255.

7. Schejtman, A., Aragão-Filho, W.C., Clare, S., Zinicola, M., Weisser, M., Burns, S.O., Booth, C., Gaspar, H.B., Thomas, D.C., Condino-Neto, A., et al. (2020). Lentiviral gene therapy rescues p47phox chronic granulomatous disease and the ability to fight Salmonella infection in mice. Gene Ther. *27*, 459–469. https://doi.org/10.1038/s41434-020-0164-6. http://www.nature.com/articles/s41434-020-0164-6.

8. Merling, R.K., Kuhns, D.B., Sweeney, C.L., Wu, X., Burkett, S., Chu, J., Lee, J., Koontz, S., Di Pasquale, G., Afione, S.A., et al. (2017). Gene-edited pseudogene resurrection corrects p47phox-deficient chronic granulomatous disease. Blood Adv. *1*, 270–278. https://doi.org/10.1182/bloodadvances.2016001214. https://ashpublications.org/bloodadvances/article/1/4/270/15524/Geneedited-pseudogene-resurrection-corrects.

9. Sürün, D., Schwäble, J., Tomasovic, A., Ehling, R., Stein, S., Kurrle, N., von Melchner, H., and Schnütgen, F. (2018). High Efficiency Gene Correction in Hematopoietic Cells by Donor-Template-Free CRISPR/Cas9 Genome Editing. Mol. Ther. Nucleic Acids *10*, 1–8. https://doi.org/10.1016/j.omtn.2017.11.001. https://linkinghub.elsevier.com/retrieve/pii/S2162253117302858.

10. Zou, J., Sweeney, C.L., Chou, B.-K., Choi, U., Pan, J., Wang, H., Dowey, S.N., Cheng, L., and Malech, H.L. (2011). Oxidase-deficient neutrophils from X-linked chronic granulomatous disease iPS cells: functional correction by zinc finger nuclease–mediated safe harbor targeting. Blood *117*, 5561–5572. https://doi.org/10.1182/blood-2010-12-328161. https://ashpublications.org/blood/article/117/21/5561/21393/Oxidasedeficient-neutrophils-from-Xlinked-chronic.

11. Merling, R.K., Sweeney, C.L., Chu, J., Bodansky, A., Choi, U., Priel, D.L., Kuhns, D.B., Wang, H., Vasilevsky, S., De Ravin, S.S., et al. (2015). An AAVS1-Targeted Minigene Platform for Correction of iPSCs From All Five Types of Chronic Granulomatous Disease. Mol. Ther. *23*, 147–157. https://doi.org/10.1038/mt.2014.195. https://linkinghub.elsevier.com/retrieve/pii/S152500161630020X.

12. De Ravin, S.S., Reik, A., Liu, P.-Q., Li, L., Wu, X., Su, L., Raley, C., Theobald, N., Choi, U., Song, A.H., et al. (2016). Targeted gene addition in human CD34+ hematopoietic cells for correction of X-linked chronic granulomatous disease. Nat. Biotechnol. *34*, 424–429. https://doi.org/10.1038/nbt.3513. http://www.nature.com/articles/nbt.3513.

13. De Ravin, S.S., Li, L., Wu, X., Choi, U., Allen, C., Koontz, S., Lee, J., Theobald-Whiting, N., Chu, J., Garofalo, M., et al. (2017). CRISPR-Cas9 gene repair of hematopoietic stem cells from patients with X-linked chronic granulomatous disease. Sci. Transl. Med. *9*, eaah3480. https://doi.org/10.1126/scitranslmed.aah3480.

14. Sweeney, C.L., Pavel-Dinu, M., Choi, U., Brault, J., Liu, T., Koontz, S., Li, L., Theobald, N., Lee, J., Bello, E.A., et al. (2021). Correction of X-CGD patient HSPCs by targeted CYBB cDNA insertion using CRISPR/Cas9 with 53BP1 inhibition for enhanced homology-directed repair. Gene Ther. *28*, 373–390. https://doi.org/10.1038/s41434-021-00251-z. http://www.nature.com/articles/s41434-021-00251-z.

15. Klatt, D., Cheng, E., Philipp, F., Selich, A., Dahlke, J., Schmidt, R.E., Schott, J.W., Büning, H., Hoffmann, D., Thrasher, A.J., and Schambach, A. (2019). Targeted Repair of p47-CGD in iPSCs by CRISPR/Cas9: Functional Correction without Cleavage in the Highly Homologous Pseudogenes. Stem Cell Rep. *13*, 590–598. https://doi.org/10.1016/j.stemcr.2019.08.008.

16. Roos, D., van Leeuwen, K., Hsu, A.P., Priel, D.L., Begtrup, A., Brandon, R., Rawat, A., Vignesh, P., Madkaikar, M., Stasia, M.J., et al. (2021). Hematologically important mutations: The autosomal forms of chronic granulomatous disease (third update). Blood Cells Mol. Dis. *92*, 102596. https://doi.org/10.1016/j.bcmd.2021.102596. https://linkinghub.elsevier.com/retrieve/pii/S1079979621000620.

17. Roesler, J., Curnutte, J.T., Rae, J., Barrett, D., Patino, P., Chanock, S.J., and Goerlach, A. (2000). Recombination events between the p47-phox gene and its highly homologous pseudogenes are the main cause of autosomal recessive chronic granulomatous disease. Blood *95*, 2150–2156. http://www.ncbi.nlm.nih.gov/pubmed/10706888.

18. Vázquez, N., Lehrnbecher, T., Chen, R., Christensen, B.L., Gallin, J.I., Malech, H., Holland, S., Zhu, S., and Chanock, S.J. (2001). Mutational analysis of patients with p47-phox-deficient chronic granulomatous disease: The significance of recombination events between the p47-phox gene (NCF1) and its highly homologous pseudogenes. Exp. Hematol. *29*, 234–243. https://doi.org/10.1016/s0301-472x(00)00646-9. http://www.ncbi.nlm.nih.gov/pubmed/11166463.

19. Wrona, D., Pastukhov, O., Pritchard, R.S., Raimondi, F., Tchinda, J., Jinek, M., Siler, U., and Reichenbach, J. (2020). CRISPR-Directed Therapeutic Correction at the NCF1 Locus Is Challenged by Frequent Incidence of Chromosomal Deletions. Mol. Ther. Methods Clin. Dev. *17*, 936–943. https://doi.org/10.1016/j.omtm.2020.04.015. https://linkinghub.elsevier.com/retrieve/pii/S2329050120300759.

20. Wada, T., Muraoka, M., Toma, T., Imai, T., Shigemura, T., Agematsu, K., Haraguchi, K., Moriuchi, H., Oh-ishi, T., Kitoh, T., et al. (2013). Rapid Detection of Intracellular p47phox and p67phox by Flow Cytometry; Useful Screening Tests for Chronic Granulomatous Disease. J. Clin. Immunol. *33*, 857–864. https://doi.org/10.1007/s10875-012-9859-9. http://link.springer.com/10.1007/s10875-012-9859-9.

21. Fazal, N., Sabeh, F., Gamelli, R.L., and Sayeed, M.M. (1997). Elevated expression of p47phox and p67phox proteins in neutrophils from burned rats. Shock *8*, 256–260. https://doi.org/10.1097/00024382-199710000-00004. http://journals.lww.com/00024382-199710000-00004.

22. Choi, J., Baldwin, T.M., Wong, M., Bolden, J.E., Fairfax, K.A., Lucas, E.C., Cole, R., Biben, C., Morgan, C., Ramsay, K.A., et al. (2019). Haemopedia RNA-seq: a database of gene expression during haematopoiesis in mice and humans. Nucleic Acids Res. *47*, D780–D785. https://doi.org/10.1093/nar/gky1020. http://www.ncbi.nlm.nih.gov/pubmed/30395284.

23. Park, J.W., Benna, J.E., Scott, K.E., Christensen, B.L., Chanock, S.J., and Babior, B.M. (1994). Isolation of a Complex of Respiratory Burst Oxidase Components from Resting Neutrophil Cytosol. Biochemistry *33*, 2907–2911. https://doi.org/10.1021/bi00176a021.

24. Nguyen, G.T., Green, E.R., and Mecsas, J. (2017). Neutrophils to the ROScue: Mechanisms of NADPH Oxidase Activation and Bacterial Resistance. Front. Cell. Infect. Microbiol. *7*, 373. https://doi.org/10.3389/fcimb.2017.00373. http://journal.frontiersin.org/article/10.3389/fcimb.2017.00373/full.

25. Ryan, M.D., and Drew, J. (1994). Foot-and-mouth disease virus 2A oligopeptide mediated cleavage of an artificial polyprotein. EMBO J. *13*, 928–933. https://doi.org/10.1002/j.1460-2075.1994.tb06337.x.

26. Minskaia, E., and Ryan, M.D. (2013). Protein Coexpression Using FMDV 2A: Effect of “Linker” Residues. BioMed Res. Int. *2013*, 291730–291812. https://doi.org/10.1155/2013/291730. http://www.hindawi.com/journals/bmri/2013/291730/.

27. Donnelly, M.L.L., Luke, G., Mehrotra, A., Li, X., Hughes, L.E., Gani, D., Ryan, M.D., Mehrotra, A., Gani, D., Donnelly, M.L.L.L., et al. (2001). Analysis of the aphthovirus 2A/2B polyprotein ‘cleavage’ mechanism indicates not a proteolytic reaction, but a novel translational effect: a putative ribosomal ‘skip. J. Gen. Virol. *82*, 1013–1025. https://doi.org/10.1099/0022-1317-82-5-1013. http://jgv.microbiologyresearch.org/content/journal/jgv/10.1099/0022-1317-82-5-1013.

28. Wrona, D., Siler, U., and Reichenbach, J. (2017). CRISPR/Cas9-generated p47 phox -deficient cell line for Chronic Granulomatous Disease gene therapy vector development. Sci. Rep. *7*, 44187–44211. https://doi.org/10.1038/srep44187.

29. Matsuzaki, Y., Umemoto, T., Tanaka, Y., Okano, T., and Yamato, M. (2015). β2-Microglobulin is an appropriate reference gene for RT-PCR-based gene expression analysis of hematopoietic stem cells. Regen. Ther. *1*, 91–97. https://doi.org/10.1016/j.reth.2015.04.003. https://linkinghub.elsevier.com/retrieve/pii/S2352320415000140.

30. Cimato, T.R., Conway, A., Nichols, J., and Wallace, P.K. (2019). CD133 expression in circulating hematopoietic progenitor cells. Cytometry B Clin. Cytom. *96*, 39–45. https://doi.org/10.1002/cyto.b.21732.

31. Anjos-Afonso, F., Buettner, F., Mian, S.A., Rhys, H., Perez-Lloret, J., Garcia-Albornoz, M., Rastogi, N., Ariza-McNaughton, L., and Bonnet, D. (2022). Single cell analyses identify a highly regenerative and homogenous human CD34+

hematopoietic stem cell population. Nat. Commun. *13*, 2048. https://doi.org/10.1038/s41467-022-29675-w. http://www.ncbi.nlm.nih.gov/pubmed/35440586.

32. Kjær, J., and Belsham, G.J. (2018). Modifications to the foot-and-mouth disease virus 2A peptide: influence on polyprotein processing and virus replication. J. Virol. *92*, e02218-17. https://doi.org/10.1128/JVI.02218-17.

33. Fu, Y.-W., Dai, X.-Y., Wang, W.-T., Yang, Z.-X., Zhao, J.-J., Zhang, J.-P., Wen, W., Zhang, F., Oberg, K.C., Zhang, L., et al. (2021). Dynamics and competition of CRISPR-Cas9 ribonucleoproteins and AAV donor-mediated NHEJ, MMEJ and HDR editing. Nucleic Acids Res. *49*, 969–985. https://doi.org/10.1093/nar/gkaa1251. http://www.ncbi.nlm.nih.gov/pubmed/33398341.

34. Guo, C., Ma, X., Gao, F., and Guo, Y. (2023). Off-target effects in CRISPR/Cas9 gene editing. Front. Bioeng. Biotechnol. *11*, 1143157. https://doi.org/10.3389/fbioe.2023.1143157. http://www.ncbi.nlm.nih.gov/pubmed/36970624.

35. Nightingale, S.J., Hollis, R.P., Pepper, K.A., Petersen, D., Yu, X.-J., Yang, C., Bahner, I., and Kohn, D.B. (2006). Transient Gene Expression by Nonintegrating Lentiviral Vectors. Mol. Ther. *13*, 1121–1132. https://doi.org/10.1016/j.ymthe.2006.01.008. https://linkinghub.elsevier.com/retrieve/pii/S1525001606000426.

36. Hanlon, K.S., Kleinstiver, B.P., Garcia, S.P., Zaborowski, M.P., Volak, A., Spirig, S.E., Muller, A., Sousa, A.A., Tsai, S.Q., Bengtsson, N.E., et al. (2019). High levels of AAV vector integration into CRISPR-induced DNA breaks. Nat. Commun. *10*, 4439. https://doi.org/10.1038/s41467-019-12449-2. http://www.nature.com/articles/s41467-019-12449-2.

37. Schmidt, M., Schwarzwaelder, K., Bartholomae, C., Zaoui, K., Ball, C., Pilz, I., Braun, S., Glimm, H., and von Kalle, C. (2007). High-resolution insertion-site analysis by linear amplification–mediated PCR (LAM-PCR). Nat. Methods *4*, 1051–1057. https://doi.org/10.1038/nmeth1103. http://www.nature.com/articles/nmeth1103.

38. Mitchell, R.S., Beitzel, B.F., Schroder, A.R.W., Shinn, P., Chen, H., Berry, C.C., Ecker, J.R., and Bushman, F.D. (2004). Retroviral DNA integration: ASLV, HIV, and MLV show distinct target site preferences. PLoS Biol. *2*, e234. https://doi.org/10.1371/journal.pbio.0020234. https://dx.plos.org/10.1371/journal.pbio.0020234.

39. Shao, L., Shi, R., Zhao, Y., Liu, H., Lu, A., Ma, J., Cai, Y., Fuksenko, T., Pelayo, A., Shah, N.N., et al. (2022). Genome-wide profiling of retroviral DNA integration and its effect on clinical pre-infusion CAR T-cell products. J. Transl. Med. *20*, 514. https://doi.org/10.1186/s12967-022-03729-5. https://translational-medicine.biomedcentral.com/articles/10.1186/s12967-022-03729-5.

40. Lazzarotto, C.R., Malinin, N.L., Li, Y., Zhang, R., Yang, Y., Lee, G., Cowley, E., He, Y., Lan, X., Jividen, K., et al. (2020). CHANGE-seq reveals genetic and epigenetic effects on CRISPR–Cas9 genome-wide activity. Nat. Biotechnol. *38*, 1317–1327. https://doi.org/10.1038/s41587-020-0555-7. https://www.nature.com/articles/s41587-020-0555-7.

41. Anthon, C., Corsi, G.I., and Gorodkin, J. (2022). CRISPRon/off: CRISPR/Cas9 on- and off-target gRNA design. Bioinformatics *38*, 5437–5439. https://doi.org/10.1093/bioinformatics/btac697. http://www.ncbi.nlm.nih.gov/pubmed/36271848.

42. Bae, S., Park, J., and Kim, J.-S. (2014). Cas-OFFinder: a fast and versatile algorithm that searches for potential off-target sites of Cas9 RNA-guided endonucleases. Bioinformatics *30*, 1473–1475. https://doi.org/10.1093/bioinformatics/btu048.

43. Altschul, S.F., Gish, W., Miller, W., Myers, E.W., and Lipman, D.J. (1990). Basic local alignment search tool. J. Mol. Biol. *215*, 403–410. https://doi.org/10.1016/S0022-2836(05)80360-2. http://www.ncbi.nlm.nih.gov/pubmed/2231712.

44. Barzel, A., Paulk, N.K., Shi, Y., Huang, Y., Chu, K., Zhang, F., Valdmanis, P.N., Spector, L.P., Porteus, M.H., Gaensler, K.M., and Kay, M.A. (2015). Promoterless gene targeting without nucleases ameliorates haemophilia B in mice. Nature *517*, 360–364. https://doi.org/10.1038/nature13864. http://www.nature.com/articles/nature13864.

45. Porro, F., Bortolussi, G., Barzel, A., De Caneva, A., Iaconcig, A., Vodret, S., Zentilin, L., Kay, M.A., and Muro, A.F. (2017). Promoterless gene targeting without nucleases rescues lethality of a Crigler-Najjar syndrome mouse model. EMBO Mol. Med. *9*, 1346–1355. https://doi.org/10.15252/emmm.201707601.

46. De Caneva, A., Porro, F., Bortolussi, G., Sola, R., Lisjak, M., Barzel, A., Giacca, M., Kay, M.A., Vlahoviček, K., Zentilin, L., and Muro, A.F. (2019). Coupling AAV-mediated promoterless gene targeting to SaCas9 nuclease to efficiently correct liver metabolic diseases. JCI Insight *5*, e128863. https://doi.org/10.1172/jci.insight.128863. https://insight.jci.org/articles/view/128863.

47. Lisjak, M., De Caneva, A., Marais, T., Barbon, E., Biferi, M.G., Porro, F., Barzel, A., Zentilin, L., Kay, M.A., Mingozzi, F., and Muro, A.F. (2022). Promoterless Gene Targeting Approach Combined to CRISPR/Cas9 Efficiently Corrects Hemophilia B Phenotype in Neonatal Mice. Front. Genome Ed. *4*, 785698. https://doi.org/10.3389/fgeed.2022.785698.

48. Ito, K., Hirao, A., Arai, F., Matsuoka, S., Takubo, K., Hamaguchi, I., Nomiyama, K., Hosokawa, K., Sakurada, K., Nakagata, N., et al. (2004). Regulation of oxidative stress by ATM is required for self-renewal of haematopoietic stem cells. Nature *431*, 997–1002. https://doi.org/10.1038/nature02989. http://www.nature.com/articles/nature02989.

49. Jang, Y.-Y., and Sharkis, S.J. (2007). A low level of reactive oxygen species selects for primitive hematopoietic stem cells that may reside in the low-oxygenic niche. Blood *110*, 3056–3063. https://doi.org/10.1182/blood-2007-05-087759. https://ashpublications.org/blood/article/110/8/3056/23952/A-low-level-of-reactive-oxygen-species-selects-for.

50. Naka, K., Muraguchi, T., Hoshii, T., and Hirao, A. (2008). Regulation of Reactive Oxygen Species and Genomic Stability in Hematopoietic Stem Cells. Antioxidants Redox Signal. *10*, 1883–1894. https://doi.org/10.1089/ars.2008.2114.

51. Karnan, S., Ota, A., Konishi, Y., Wahiduzzaman, M., Hosokawa, Y., and Konishi, H. (2016). Improved methods of AAV-mediated gene targeting for human cell lines using ribosome-skipping 2A peptide. Nucleic Acids Res. *44*, e54. https://doi.org/10.1093/nar/gkv1338.

52. Kuhns, D.B., Alvord, W.G., Heller, T., Feld, J.J., Pike, K.M., Marciano, B.E., Uzel, G., DeRavin, S.S., Priel, D.A.L., Soule, B.P., et al. (2010). Residual NADPH Oxidase and Survival in Chronic Granulomatous Disease. N. Engl. J. Med. *363*, 2600–2610. https://doi.org/10.1056/NEJMoa1007097.

53. Marciano, B.E., Spalding, C., Fitzgerald, A., Mann, D., Brown, T., Osgood, S., Yockey, L., Darnell, D.N., Barnhart, L., Daub, J., et al. (2015). Common Severe Infections in Chronic Granulomatous Disease. Clin. Infect. Dis. *60*, 1176–1183. https://doi.org/10.1093/cid/ciu1154.

54. Grunebaum, E., Arnold, D.E., Logan, B., Parikh, S., Marsh, R.A., Griffith, L.M., Mallhi, K., Chellapandian, D., Lim, S.S., Deal, C.L., et al. (2024). Allogeneic hematopoietic cell transplantation is effective for p47phox chronic granulomatous disease: A Primary Immune Deficiency Treatment Consortium study. J. Allergy Clin. Immunol. *153*, 1423–1431.e2. https://doi.org/10.1016/j.jaci.2024.01.013. https://linkinghub.elsevier.com/retrieve/pii/S0091674924000812.

55. Romero, Z., Lomova, A., Said, S., Miggelbrink, A., Kuo, C.Y., Campo-Fernandez, B., Hoban, M.D., Masiuk, K.E., Clark, D.N., Long, J., et al. (2019). Editing the Sickle Cell Disease Mutation in Human Hematopoietic Stem Cells: Comparison of Endonucleases and Homologous Donor Templates. Mol. Ther. *27*, 1389–1406. https://doi.org/10.1016/j.ymthe.2019.05.014. https://linkinghub.elsevier.com/retrieve/pii/S152500161930228X.

56. Schiroli, G., Conti, A., Ferrari, S., della Volpe, L., Jacob, A., Albano, L., Beretta, S., Calabria, A., Vavassori, V., Gasparini, P., et al. (2019). Precise Gene Editing Preserves Hematopoietic Stem Cell Function following Transient p53-Mediated DNA Damage Response. Cell Stem Cell *24*, 551–565.e8. https://doi.org/10.1016/j.stem.2019.02.019.

57. Ferrari, S., Jacob, A., Beretta, S., Unali, G., Albano, L., Vavassori, V., Cittaro, D., Lazarevic, D., Brombin, C., Cugnata, F., et al. (2020). Efficient gene editing of human long-term hematopoietic stem cells validated by clonal tracking. Nat. Biotechnol. *38*, 1298–1308. https://doi.org/10.1038/s41587-020-0551-y. https://www.nature.com/articles/s41587-020-0551-y.

58. Ferrari, S., Jacob, A., Cesana, D., Laugel, M., Beretta, S., Varesi, A., Unali, G., Conti, A., Canarutto, D., Albano, L., et al. (2022). Choice of template delivery mitigates the genotoxic risk and adverse impact of editing in human hematopoietic stem cells. Cell Stem Cell *29*, 1428–1444.e9. https://doi.org/10.1016/j.stem.2022.09.001. http://www.ncbi.nlm.nih.gov/pubmed/36206730.

59. Dorset, S.R., and Bak, R.O. (2023). The p53 challenge of hematopoietic stem cell gene editing. Mol. Ther. Methods Clin. Dev. *30*, 83–89. https://doi.org/10.1016/j.omtm.2023.06.003. http://www.ncbi.nlm.nih.gov/pubmed/37435043.

60. Ebisu, K., Nagasawa, T., Watanabe, K., Kakinuma, K., Miyano, K., and Tamura, M. (2001). Fused p47phox and p67phox truncations efficiently reconstitute NADPH oxidase with higher activity and stability than the individual components. J. Biol. Chem.

276, 24498–24505. https://doi.org/10.1074/jbc.M101122200. http://www.ncbi.nlm.nih.gov/pubmed/11333262.

61. Miyano, K., Fukuda, H., Ebisu, K., and Tamura, M. (2003). Remarkable stabilization of neutrophil NADPH oxidase using RacQ61L and a p67phox-p47phox fusion protein. Biochemistry *42*, 184–190. https://doi.org/10.1021/bi0269052. http://www.ncbi.nlm.nih.gov/pubmed/12515553.
62. Wanisch, K., and Yáñez-Muñoz, R.J. (2009). Integration-deficient Lentiviral Vectors: A Slow Coming of Age. Mol. Ther. *17*, 1316–1332. https://doi.org/10.1038/mt.2009.122. https://linkinghub.elsevier.com/retrieve/pii/S1525001616318524.
63. Deyle, D.R., and Russell, D.W. (2009). Adeno-associated virus vector integration. Curr. Opin. Mol. Therapeut. *11*, 442–447. http://www.ncbi.nlm.nih.gov/pubmed/19649989.
64. Colella, P., Ronzitti, G., and Mingozzi, F. (2018). Emerging Issues in AAV-Mediated In Vivo Gene Therapy. Mol. Ther. Methods Clin. Dev. *8*, 87–104. https://doi.org/10.1016/j.omtm.2017.11.007. https://linkinghub.elsevier.com/retrieve/pii/S2329050117301237.
65. Miller, D.G., Petek, L.M., and Russell, D.W. (2004). Adeno-associated virus vectors integrate at chromosome breakage sites. Nat. Genet. *36*, 767–773. https://doi.org/10.1038/ng1380. http://www.nature.com/articles/ng1380.
66. Kosicki, M., Tomberg, K., and Bradley, A. (2018). Repair of double-strand breaks induced by CRISPR–Cas9 leads to large deletions and complex rearrangements. Nat. Biotechnol. *36*, 765–771. https://doi.org/10.1038/nbt.4192. http://www.nature.com/articles/nbt.4192.
67. Adikusuma, F., Piltz, S., Corbett, M.A., Turvey, M., McColl, S.R., Helbig, K.J., Beard, M.R., Hughes, J., Pomerantz, R.T., and Thomas, P.Q. (2018). Large deletions induced by Cas9 cleavage. Nature *560*, E8–E9. https://doi.org/10.1038/s41586-018-0380-z. https://www.nature.com/articles/s41586-018-0380-z.
68. Inagaki, K., Lewis, S.M., Wu, X., Ma, C., Munroe, D.J., Fuess, S., Storm, T.A., Kay, M.A., and Nakai, H. (2007). DNA palindromes with a modest arm length of greater, similar 20 base pairs are a significant target for recombinant adeno-associated virus vector integration in the liver, muscles, and heart in mice. J. Virol. *81*, 11290–11303. https://doi.org/10.1128/JVI.00963-07.
69. Kaeppel, C., Beattie, S.G., Fronza, R., van Logtenstein, R., Salmon, F., Schmidt, S., Wolf, S., Nowrouzi, A., Glimm, H., von Kalle, C., et al. (2013). A largely random AAV integration profile after LPLD gene therapy. Nat. Med. *19*, 889–891. https://doi.org/10.1038/nm.3230. http://www.nature.com/articles/nm.3230.
70. Nowrouzi, A., Penaud-Budloo, M., Kaeppel, C., Appelt, U., Le Guiner, C., Moullier, P., von Kalle, C., Snyder, R.O., and Schmidt, M. (2012). Integration Frequency and Intermolecular Recombination of rAAV Vectors in Non-human Primate Skeletal Muscle and Liver. Mol. Ther. *20*, 1177–1186. https://doi.org/10.1038/mt.2012.47. https://linkinghub.elsevier.com/retrieve/pii/S1525001616325412.
71. Li, H., Malani, N., Hamilton, S.R., Schlachterman, A., Bussadori, G., Edmonson, S.E., Shah, R., Arruda, V.R., Mingozzi, F., Wright, J.F., et al. (2011). Assessing the potential for AAV vector genotoxicity in a murine model. Blood *117*, 3311–3319. https://doi.org/10.1182/blood-2010-08-302729. https://ashpublications.org/blood/article/117/12/3311/20420/Assessing-the-potential-for-AAV-vector.
72. PLB-985 (Subclone of HL-60) ACC 139. Leibniz Institute DSMZ-German Collection of Microorganisms and Cell Cultures GmbH. https://webshop.dsmz.de/en/human-animal-cell-lines/PLB-985-subclone-of-HL-60.html.
73. Chick, H.E., Nowrouzi, A., Fronza, R., McDonald, R.A., Kane, N.M., Alba, R., Delles, C., Sessa, W.C., Schmidt, M., Thrasher, A.J., and Baker, A.H. (2012). Integrase-deficient lentiviral vectors mediate efficient gene transfer to human vascular smooth muscle cells with minimal genotoxic risk. Hum. Gene Ther. *23*, 1247–1257. https://doi.org/10.1089/hum.2012.042. http://www.ncbi.nlm.nih.gov/pubmed/22931362.
74. Vakulskas, C.A., Dever, D.P., Rettig, G.R., Turk, R., Jacobi, A.M., Collingwood, M.A., Bode, N.M., McNeill, M.S., Yan, S., Camarena, J., et al. (2018). A high-fidelity Cas9 mutant delivered as a ribonucleoprotein complex enables efficient gene editing in human hematopoietic stem and progenitor cells. Nat. Med. *24*, 1216–1224. https://doi.org/10.1038/s41591-018-0137-0. https://www.nature.com/articles/s41591-018-0137-0.
75. Hayrapetyan, A., Dencher, P.C.D., van Leeuwen, K., de Boer, M., and Roos, D. (2013). Different unequal cross-over events between NCF1 and its pseudogenes in autosomal p47(phox)-deficient chronic granulomatous disease. Biochim. Biophys. Acta *1832*, 1662–1672. https://doi.org/10.1016/j.bbadis.2013.05.001. http://www.ncbi.nlm.nih.gov/pubmed/3688784.
76. Gori, J., Heath, J.M., and Tedeschi, J.G. (2023). Prime editing of human CD34+ long-term hematopoietic stem cells precisely corrects the causative mutation of p47phox chronic granulomatous disease and restores NADPH oxidase activity in myeloid progeny. Mol. Ther. *31*, 55.
77. Fiumara, M., Ferrari, S., Omer-Javed, A., Beretta, S., Albano, L., Canarutto, D., Varesi, A., Gaddoni, C., Brombin, C., Cugnata, F., et al. (2023). Genotoxic effects of base and prime editing in human hematopoietic stem cells. Nat. Biotechnol. https://doi.org/10.1038/s41587-023-01915-4. https://www.nature.com/articles/s41587-023-01915-4.
78. Kuzminov, A. (2001). Single-strand interruptions in replicating chromosomes cause double-strand breaks. Proc. Natl. Acad. Sci. USA *98*, 8241–8246. https://doi.org/10.1073/pnas.131009198.
79. Paterna, J.-C., Moccetti, T., Mura, A., Feldon, J., and Büeler, H. (2000). Influence of promoter and WHV post-transcriptional regulatory element on AAV-mediated transgene expression in the rat brain. Gene Ther. *7*, 1304–1311. https://doi.org/10.1038/sj.gt.3301221.
80. Labun, K., Montague, T.G., Krause, M., Torres Cleuren, Y.N., Tjeldnes, H., and Valen, E. (2019). CHOPCHOP v3: expanding the CRISPR web toolbox beyond genome editing. Nucleic Acids Res. *47*, W171–W174. https://doi.org/10.1093/nar/gkz365. https://academic.oup.com/nar/article/47/W1/W171/5491735.
81. Burger, A., Lindsay, H., Felker, A., Hess, C., Anders, C., Chiavacci, E., Zaugg, J., Weber, L.M., Catena, R., Jinek, M., et al. (2016). Maximizing mutagenesis with solubilized CRISPR-Cas9 ribonucleoprotein complexes. Development (Cambridge, U. K.) *143*, 2025–2037. https://doi.org/10.1242/dev.134809.
82. Jinek, M., Chylinski, K., Fonfara, I., Hauer, M., Doudna, J.A., and Charpentier, E. (2012). A Programmable Dual-RNA–Guided DNA Endonuclease in Adaptive Bacterial Immunity. Science *337*, 816–821. https://doi.org/10.1126/science.1225829.
83. Jinek, M., Jiang, F., Taylor, D.W., Sternberg, S.H., Kaya, E., Ma, E., Anders, C., Hauer, M., Zhou, K., Lin, S., et al. (2014). Structures of Cas9 Endonucleases Reveal RNA-Mediated Conformational Activation. Science *343*, 1247997. https://doi.org/10.1126/science.1247997.
84. Brinkman, E.K., Chen, T., Amendola, M., van Steensel, B., and van Steensel, B. (2014). Easy quantitative assessment of genome editing by sequence trace decomposition. Nucleic Acids Res. *42*, e168. https://doi.org/10.1093/nar/gku936. http://academic.oup.com/nar/article/42/22/e168/2411890/Easy-quantitative-assessment-of-genome-editing-by.
85. Schneider, C.A., Rasband, W.S., and Eliceiri, K.W. (2012). NIH Image to ImageJ: 25 years of image analysis. Nat. Methods *9*, 671–675. https://doi.org/10.1038/nmeth.2089.

OMTN, Volume *35*

Supplemental information

Targeted knock-in of *NCF1* cDNA into the *NCF2* locus leads to myeloid phenotypic correction of p47*phox*-deficient chronic granulomatous disease

Kah Mun Siow, Merve Güngör, Dominik Wrona, Federica Raimondi, Oleksandr Pastukhov, Panagiotis Tsapogas, Timon Menzi, Michael Schmitz, Péter István Kulcsár, Gerald Schwank, Ansgar Schulz, Martin Jinek, Ute Modlich, Ulrich Siler, and Janine Reichenbach

Supplemental Material

Supplemental Methods

Cas12a nuclease preparation

The *Acidaminococcus sp.* (As)Cas12a gene was amplified by PCR (Addgene plasmid #90095; 6His-MBP-TEV-huAsCpf1 was provided by Feng Zhang; http://n2t.net/addgene:90095)[1] to replace the Cas9 gene in the Cas9 expression vector by Gibson assembly, generating the 6xHis-MBP-TEV-Cas12a-HA-2xNLS-GFP-NLS expression construct. Purification of Cas12a was done as described.[2,3] Cas12a construct was expressed in *E. coli* BL21 Rosetta2 (DE3) cells (Merck KgaA, Germany). Eluted protein was dialyzed against 20 mM HEPES pH 7.5, 250 mM KCl, 1 mM dithiothreitol (DTT), 1 mM EDTA (Cas12a) overnight at 4 °C in the presence of TEV protease to remove the 6xHis-MBP affinity tag. The subsequent procedures (clarification, concentration, and purification steps) were performed as described in the 'Materials and methods' section.

***In vitro* cell-free cleavage assay**

PCR product (857 bp) containing the *NCF2* on-target site was cloned into pCR Blunt II-TOPO vector, using Zero Blunt TOPO PCR Cloning Kit (Thermo Fisher Scientific, USA). The plasmid was linearized using SmaI (NEB, USA) as a target DNA for *in vitro* cleavage assay. Prior to RNP complexing, gRNA was denatured at 95 °C for 2 min and incubated at room temperature for 30 min. RNP was complexed with 1.5 µM of Cas9-GFP protein and 1.5 µM of gRNA for 15 min at room temperature. 55 ng of linearized target DNA was mixed with RNP complex and incubated for 1 hour at 37 °C. Reaction was stopped by addition of 0.02 M ethylenediamine tetraacetic acid (EDTA) and 1 µg/µL proteinase K (Roche, Switzerland), and incubated in a thermocycler (Biometra TOne, Analytik Jena, Germany) at 37 °C for 30 min. Digestion products were visualized on 1% agarose gel following electrophoresis. Band intensities were measured by densitometric analysis using ImageJ software (National Institutes of Health, USA).[1] Cleavage efficiency resulting from DSBs was calculated using the following formula: % Cleavage = 100 x (1 – (1-fraction cleaved)$^{1/2}$).

DNA isolation by Buffer K lysis

Genomic DNA from whole cells was lysed in Buffer K containing 0.1 mg/mL proteinase K (Roche, Switzerland), and 1X (v/v) GC buffer (Thermo Fisher Scientific, USA) in RNAse-/DNase-free water. Samples were incubated at 57 °C for 45 min, followed by heat inactivation at 95 °C for 15 min.

T7 endonuclease I (T7EI) assay

Genomic DNA was isolated 4 days after treatment, PCR-purified using QIAquick Gel Extraction Kit or QIAquick PCR Purification Kit (QIAGEN, Germany) and subjected to T7EI assay. A total of 100 ng purified PCR products from untreated control and treated samples were hybridized in a thermocycler (Biometra TOne, Analytik Jena, Germany) by heating at 95 °C for 5 min, followed by slow cooling from 95 °C to 25 °C to form heteroduplexes. Heteroduplex DNA was then treated with 1U of T7EI (NEB, USA) and incubated in the thermocycler at 37 °C for 15 min. The reaction was blocked by addition of 0.02 M EDTA and analyzed by 1% agarose gel following electrophoresis. Band intensities were subjected to densitometric analysis using ImageJ software (National Institutes of Health, USA).[4] Non-homologous end joining (NHEJ) frequencies were calculated using the following formula: % Cleavage = 100 x (1 – (1- fraction cleaved)$^{1/2}$).

Copy number variation (CNV) analysis of *NCF2* by ddPCR

CNV quantification was determined by ddPCR using QX200 Droplet Reader (Bio-Rad Laboratories, USA). Genomic DNA was digested with DraI enzyme (20 U/µL) (NEB, USA). 50-100 ng of digested gDNA was used per ddPCR reaction, primers, and probes (concentrations are stated in 'Materials and methods') targeting 3 locations upstream and 2 locations downstream of *NCF2* target site using primer *NCF2*_T1 to _T5 with reference to *FOXP2* housekeeping gene (Table S2). For droplet generation, 20 µL of sample reaction was transferred to a DG8 Cartridges for QX200/QX100 Droplet Generator (Bio-Rad Laboratories) and processed according to manufacturer's recommendation. Droplet generation and analysis were performed as described in the 'Materials and methods' section.

Characterization of identified integration sites by linear amplification mediated PCR (LAM-PCR)

The conditions for the first and second nested PCR were: initial denaturation for 10 min at 98 °C, then amplification with 35 cycles of denaturation (98 °C, 10 sec), annealing (62 °C, 10 sec) and extension (72 °C, 20 sec), with a final 5 min incubation at 72 °C. ISs were identified by Sanger sequencing of subcloned amplicons from the second nested PCR. The sequences were analysed for the presence of LTR III or ITR III primers, a restriction enzyme recognition site, and LC II primer. The unknown genome fragment was then subjected to standard nucleotide BLAST (blastn) using the National Center for Biotechnology Information (NCBI) webtool.[5] To calculate the distance of IS from TSS, the closest TSS was determined by identification of the first nucleotide of the 5' untranslated region (5' UTR) of the gene with the IS. To verify if the IS contained potential gRNA binding sites, a list of CRISPR off-targets was generated *in silico,* using CRISPRoff (v1.2beta) and Cas-OFFinder,[6,7] with a query criteria of up to 6 mismatches, and 2 DNA or RNA bulges around the IS (200bp upstream and downstream). The ISs were then manually analysed using the generated list of off targets, followed by a standard alignment using blastn.

Supplemental Tables

Table S1. Guide RNA target sequences. Underlining denotes the PAM sequence of the sgRNA or the crRNA target.

Target gene	Name	Cas nuclease	Guide RNA target sequence (5'-3'), PAM
Human *NCF2*	sgRNA #1	Cas9	AAGCACTCGGAGAGAAGTCTAGG
Human *NCF2*	sgRNA #2	Cas9	AGACTGCGCAACTACAGATTTGG
Human *NCF2*	sgRNA #3	Cas9	CTACAGATTTGGAAAGCACTCGG
Human *NCF2*	sgRNA #4	Cas9	AATCTTACAAACAAGTAATAGGG
Human *NCF2*	sgRNA #5	Cas9	GCAGTCTTCAACAAAAACTTTGG
Human *NCF2*	sgRNA #6	Cas9	AGTCTTCAACAAAAACTTTGGGG
Human *NCF2*	sgRNA #7	Cas9	CAGTCTTCAACAAAAACTTTGGG
Human *NCF2*	sgRNA #8	Cas9	AAATCTTACAAACAAGTAATAGG
Human *NCF2*	crRNA #1	Cas12a	TTTCCCCAAAGTTTTTGTTGAAGACTGC
Human *NCF2*	crRNA #2	Cas12a	TTTCCAAATCTGTAGTTGCGCAGTCTTC
Human *NCF2*	crRNA #3	Cas12a	TTTTTGTTGAAGACTGCGCAACTACAGA
Human *NCF2*	crRNA #4	Cas12a	TTTTGTTGAAGACTGCGCAACTACAGAT
Human *NCF2*	crRNA #5	Cas12a	TTTTCTTCAGCTTTGTAGTTTGTGAAAC
Human *NCF2*	crRNA #6	Cas12a	TTTGTAAGATTTAGCACCCTTCTGCTGT
Human *NCF2*	crRNA #7	Cas12a	TTTAGCACCCTTCTGCTGTATACTGTAC
Human *NCF2*	crRNA #8	Cas12a	TTTCACAAACTACAAAGCTGAAGAAAAT

Table S2. Primer and probe sequences for ddPCR. BHQ1, Black Hole Quencher®. Brackets denote fluorophore or quencher modifications.

Target gene	Primer	Sequence (5'-3')	Amplicon size (bp)
**2A-*NCF1***	Forward	TGACCTTCTCAAGTTGGCGG	122
	Reverse	TGTACACGTAGTGCTGGCTG	
	Probe	[6FAM]-CGGCGACACCTTCATCCGGCATATC-[BHQ1]	
ITR	Forward	TCCTCTAGAGTCGACCTGC	75
	Reverse	TGATTAACCCGCCATGCTAC	
	Probe	[6FAM]-ACGTAGCCATGCTCTAGAGCTTGC-[BHQ1]	
wPRE	Forward	TTCTGGGACTTTCGCT	118
	Reverse	CGACAACACCACGGAATTATC	

	Probe	[6FAM]-TCGCCACGGCAGAACTCATC-[BHQ1]	
FOXP2	Forward	TTGCTGGGTGTGTTTAATGC	139
	Reverse	AGGGTATAATGGATGGTGGC	
	Probe	[HEX]-CTGCCCGTGCCCTGGCCCAC-[BHQ1]	
NCF2_T1	Forward	GGCACCAGTGCCTTAATGTC	91
(-1124 bp from	Reverse	GTATGATCTCCAAAGCCCACCT	
Cut side)	Probe	[6FAM]-ACTTCTGAACTGGCTGTGTGGTCA-[3IBFQ]	
NCF2_T2	Forward	CTCACCTGTTTAATGGAGTTCAGTG	128
(-794 bp from	Reverse	TACCTGCGCTTTGTTGGGAA	
Cut side)	Probe	[6FAM]-ATTTAACCAATGGCATGTCCCTTT-[3IBFQ]	
NCF2_T3	Forward	TGTGGAATAGCCAGACAGGG	128
(-111 bp from	Reverse	TTTGGGGAAAATGCCCACCT	
Cut side)	Probe	[6FAM]-TCAGTGAATGAAGAATGGCTGGAAGGG-[3IBFQ]	
NCF2_T4	Forward	GCACAGTGGGTGAGGAGATG	99
(+231 bp from	Reverse	CCTGGAAGACTCTCTCGTGC	
Cut side)	Probe	[6FAM]-CCCTAACTCCTCCATTCACCTGTCC-[3IBFQ]	
NCF2_T5	Forward	TGGACTACAACACTCAATTCCAC	151
(+682 bp from	Reverse	ACTAGCTTCATGAACACCGCT	
Cut side)	Probe	[6FAM]-ACCTTTGTACATTCTCAGAGAGGAACATGT-[3IBFQ]	
NCF2_T6	Forward	ATTTGGCTAACCCAATTGTCCA	126
(+1266 bp from	Reverse	ACGCTACAACAAGAAGTGGGT	
Cut side)	Probe	[6FAM]-CCTGGCCTTAGTTCCTCTCCCT-[3IBFQ]	
		**- Primer did not work**	

Table S3. Oligonucleotide sequences for TIDE and Sanger sequencing.

Primer	Target gene/purpose	Sequence (5'-3')
**p67_F38773**	*NCF2*/TIDE forward primer	CCCCTATCACCACAGCACTT
**p67_R38975**	*NCF2*/TIDE reverse primer	CCTGGACTTGGGTGTCTTGT
**seqp67_R**	*NCF2*/Sanger sequencing	GTGCCCTTTCCAGACACTTC
**R8.91_6**	D64V/to check IDLV D64V packaging plasmid sequence	ACCTACCACCTGTAGTAGCA

Table S4. Antibodies and reagents for flow cytometry

Antibody/Reagent/Dye	Cat. No.	Manufacturer	Isotype	Clone
Rat anti-human/mouse CD11b V450	560456	BD Biosciences	Rat IgG2β,κ	M1/70
Rat anti-human CD11b PE-Cy7	101216	Biolegend	Rat IgG2β,κ	M1/70
Mouse anti-human CD15 VioBlue	130-113-488	Miltenyi Biotec	mouse IgM	VIMC6
REAfinity anti-human CD14 APC	130-110-520	Miltenyi Biotec	Human IgG1	REA599
Mouse anti-human p47^{phox} APC	624076	BD Biosciences	Mouse IgG1	1/p47phox
Mouse anti-human p67^{phox} AF488	sc-374510	Santa Cruz	Mouse IgG1,κ	D-6
Mouse anti-human CD34 PE-Cy7	A21691	Beckman Coulter	Mouse IgG1	581
Mouse anti-human CD38 BV605	562665	BD Biosciences	Mouse IgG1, κ	HB7
Mouse anti-human CD45RA BV786	563870	BD Biosciences	Mouse IgG2b, κ	HI100
Mouse anti-human CD133/1 PE	130-113-670	Miltenyi Biotec	Mouse IgG1, κ	AC133
Human anti-mouse c-Kit VioBright B515	130-111-618	Miltenyi Biotec	Human IgG1	REA791
Human anti-mouse Sca-1 PE	130-116-489	Miltenyi Biotec	Human IgG1	REA422
Lineage cell detection cocktail biotin	130-092-613	Miltenyi Biotec	N/A	N/A
LIVE/DEAD Fixable Yellow Dead cell stain kit	L34968	Thermo Fisher	N/A	N/A

LIVE/DEAD Fixable Near-IR Dead cell stain kit	L10119	Thermo Fisher	N/A	N/A
LIVE/DEAD Fixable Aqua Dead cell stain kit	L34957	Thermo Fisher	N/A	N/A
Rat IgG2β,κ isotype control V450	560457	BD Biosciences	Rat IgG2β,κ	A95-1
Rat IgG2β,κ isotype control PE-Cy7	400618	Biolegend	Rat IgG2β,κ	RTK4530
Mouse IgG1,κ isotype control APC	400120	Biolegend	Mouse IgG1,κ	MOPC-21
Mouse IgG1 isotype control AF488	sc-3890	Santa Cruz	Mouse IgG1	N/A
Human IgG1 isotype control VioBright B515	130-113-457	Miltenyi Biotec	Human IgG1	REA293
Human IgG1 isotype control PE	130-113-462	Miltenyi Biotec	Human IgG1	REA293
Human anti-biotin VioBlue	130-111-068	Miltenyi Biotec		REA746
FcR blocking reagent, human	130-059-901	Miltenyi Biotec	N/A	N/A
FcR blocking reagent, mouse	130-092-575	Miltenyi Biotec	N/A	N/A
Propidium iodide	P4170	Sigma-Aldrich	N/A	N/A

Table S5. Information of integration sites identified by LAM-PCR in chosen knocked-in clones. Clone #, clone name described in Figure S12C and S12D. VCN, vector copy number measured by ddPCR as described in 'Materials and methods'. IS chromosomal location, gene of IS, and IS location description are the location information of the IS determined by LAM-PCR based on the *Homo sapiens* genome assembly hg38 (GRCh38.p14). (-) indicates genomic orientation of a reverse strand. TSS, transcriptional start site predicted by Ensembl (https://www.ensembl.org)[8] that is closest to the IS. Identified ISs were checked on *in silico* CRISPR off-target sites of sgRNA *NCF2* using webtools, CRISPRoff (v1.2beta) and Cas-OFFinder.[7,9] Guide RNA binding sites with up to 6 mismatches and 2 DNA or RNA bulges around the IS (200bp upstream and downstream) are checked manually on standard nucleotide BLAST (blastn) using the National Center for Biotechnology Information (NCBI) webtool.[5] No potential CRISPR off-target sites were matched with the identified IS except on Clone 24 (Figure S11D), whereby a 11-nucleotide match of the sgRNA *NCF2* sequence + 1 DNA bulge including the protospacer adjacent motif (PAM) sequence was found 4 bp downstream of IS.

Clones derived from PLB-985 *NCF1* ΔGT cells treated with RNP+AAV *NCF1* at MOI 1e7 vg/cell (Figure S12C)

Clone # on Figure S11C	VCN 2A-*NCF1*	VCN ITR	IS chromosomal location	Gene of IS	IS location description	Predicted TSS at the gene	CRISPR-OFF / CasOFFinder / BLASTn alignment
**24**	1.19	0.67	Unidentified	-	-	-	-/-/-
**32**	3.19	1.53	11p13: 32874235	-	intergenic	NA	-/-/-

Clones derived from PLB-985 *NCF1* ΔGT cells treated with RNP+IDLV *NCF1* at MOI 6e3 LP/cell (Figure S12D)

Clone # on Figure S11D	VCN 2A-*NCF1*	VCN wPRE5	IS chromosomal location	Gene of IS	IS location description	Predicted TSS at the gene	CRISPR-OFF / CasOFFinder / BLASTn alignment
**10**	1.71	1.04	Unidentified	-	-	-	-/-/-
**11**	1.89	1.03	Unidentified	-	-	-	-/-/-
**14**	2.20	0.78	6p22.22: 6094020	HFE	intron 5 of HFE	26087439	-/-/-
			2p22.1: 40610981	-	intergenic	-	-/-/-
**15**	2.29	1.92	16: tandem repeats between 36340645-36421329	-	centromere	-	-/-/-
			1p32.2: 57536282	DAB1	intron 3 of DAB1	57291065	-/-/-
**16**	2.51	1.80	6p22.3: 18966464	-	intergenic	-	-/-/-
**17**	2.79	1.80	Unidentified	-	-	-	-/-/-

**18**	2.81	0.96	Unidentified	-	-	-	-/-/-
**19**	2.88	1.84	19p13.3: 1964394	CSNK1G2	intron 1 of CSNK1G2	1,941,172	-/-/-
			3q26.33: 181177379	SOX2-OT	intron 3 of SOX2-OT	181174890	-/-/-
**20**	2.89	1.15	11q12.1: 57960715	-	intergenic	-	-/-/-
			3q29: 196271658	PCYT1A	intron 1 of PCYT1A	196270541	-/-/-
**21**	2.98	2.63	Unidentified	-	-	-	-/-/-
**22**	3.01	2.58	8q24.21:129574663 (-)	CCDC26	Intron 1 of CCDC26	129574944	-/-/-
			6q13: 73299191	KHDC1	Intron 1 of KHDC1	73,310,215	-/-/-
			5p13.3-p13.2: 33562256 (-)	ADAMTS12	Intron 19 of ADAMTS12	33891941	-/-/-
**23**	3.62	1.91	1q32.2: 211096126 (-)	KCNH1	Intron 3 of KCNH1	211,103,515	-/-/-
			12p13.33: 1037807	ERC1	Intron 2 of ERC1	1,027,772	-/-/-
**24**	4.44	2.75	22q13.1: 37716096	TRIOBP	Intron 5 of TRIOBP	37,697,048	-/-/-
			12q24.33: 130627848 (-)	RIMBP2	Intron of RIMBP2	130633861	-/-/matched GGAGAGAANTCTAGG at 130627496-130627482 but 1 mismatch at seed sequence

Table S6. Primers used for LAM-PCR. Brackets indicate the specified modifications at the 5'. Underlined denotes TasI restriction enzyme recognition site.

Primer	Target gene/purpose	Sequence (5'-3')
**LTR I_Biotin**	LTR/Linear PCR amplification	[Biotin]-GAACCCACTGCTTAAGCCTCA
**LTR II**	LTR/Nested PCR 1	GCTTGCCTTGAGTGCTTCA
**LTR III**	LTR/Nested PCR 2	AGTAGTGTGTGCCCGTCTGT
**ITR I_Biotin**	ITR/Linear PCR amplification	[Biotin]-ACCTCTGCCTGGTACCGAAT
**ITR II**	ITR/Nested PCR 1	GTAGCATGGCGGGTTAATCA
**ITR III**	ITR/Nested PCR 2	CAAAGGTCGCCCGACGC
**LC I**	Linker/Nested PCR 1	GACCCGGGAGATCTGAATTC
**LC II**	Linker/Nested PCR2	AGTGGCACAGCAGTTAGG
**Linker**	Linker cassette generation – top	GACCCGGGAGATCTGAATTCAGTGGCACAGCAGTTAGG
**Linker**	Linker cassette generation – bottom	[Phosphorylation]-AATTCCTAACTGCTGTGCCACTGAATTCAGATC

Table S7. Sequence of integration sites identified by LAM-PCR in chosen knocked-in clones. Clone #, clone name described in Figure S11C and S11D. (-) indicates genomic orientation of a reverse strand. Blue denotes the LTR III or ITR III primer sequence that binds to the LTR or ITR, respectively. Green denotes the linker LC II primer sequence. Underlined indicates the TasI restriction enzyme recognition site. Italicized indicates an unknown genomic sequence captured by LAM-PCR next to an LTR/ITR sequence.

Clone # on Figure S11C	Chromosomal location of IS	Sequence identified by subcloning after 2nd nested PCR
32	11p13	**GAACCCCTAGTGATGGAGTTG***GCCACACCCTTGGTTTGCTCTTCTAAACATGACTTTTCACTCTTTACATGGCCAGGCTGGACATTTTCCAAGTCTTTCTATTCTGCTTCCCTTTTAAATGATAAATGCCATCTTTAAGTCATGCGTGTCCACTCTA*AATT**CCTAACTGCTGTGCCACT**

Clone # on Figure S11D		Sequence
14	6p22.2	**AGTGGCACAGCAGTTAGG**AATT*ATGCATTTCTACCCCCTGAACATCTGTGGTGTAGGGAAAAGAGAATCAGAAAGAAGCCAGCTCATACAGAGTCCAAGGGTCTTTTGGGATATTGGGTTATGATCACTGGGGTGTCATTGAAGGATCCTAAGAAAGGAGGACCACGATCTCCCTTATATGGTGAATGTGTTGTTAAGAAGTTAGATGAGAGGTGAGGAGACCAG*TCAAGTCCCTGTTCGGGCGCCACTGCTAGAGATTTTCCACACTGACTAAAAGGGTCTGAGGGATCTCTAGTTACCAGAGTCACACA**ACAGACGGGCACACACTACT**
	2p22.1	**AGTAGTGTGTGCCCGTCTGT**TGTGTGACTCTGGTAACTAGAGATCCCTCAGACCCTTTTAGTCAGTGTGGAAAATCTCTAGCAGTGGCGCCCGAACAGGGACTTGAAAGCGAAAGGGAAACCAGAGG*CCTCTCCAGAAGCAGATTCTAGCACCATGCTGCTCGCACAGCCTTCAAAACTGTGAGTCAAATAAATCTCTTTTATTTATAAATTACTCAGTCTCAGATACTTTTACAGCAGTGCAAATGGATGAACACAGAAAATTAGTATCAAGGATCTCTCTCCTTCTAGCCTCCGCTAGTCAA*AATT**CCTAACTGCTGTGCCACT**
15	16 centromere	**AGTGGCACAGCAGTTAGG**AATT*TGCAAGTGGAGATTTCAGCCGCTTTGAGGTCAATGGTAGAAAAGGAAATATCTTCGTATAAAAACTAGACAGAATGATTCTCAGAAACTCCTTTGTGATGTGTGCGTTCAACTCACAGAGTTTAACCTTTCTTTTCACAGAGCAGTTAGGAAACACTCTGTTTGTGAAGCCTGCCAGTGGATATTC*CTTCGTTTCTCAGTAGCATCATTTAAATGATTAGCTCAGTGCTTTCAAGTCCCTGTTCGGGCGCCACTGCTAGAGATTTTCCACACTGACTAAAAGGGTCTGAGGGATCTCTAGTTACCAGAGTCACACA**ACAGACGGGCACACACTACT**
	1p32.2	**AGTGTGTGCCCGTCTGT**TGTGTGACTCTGGTAACTAGAGATCCCTCAGACCCTTTTAGTCAGTGTGGAAAATCTCTAGCAGTGGCGCCCGAACAGGGACTTGAAAGCGAAAGGG*GACAAACTTTGTGAGTCAGCTAGTGCCAAAAAAGCAGGACAGATTTCTGTAAAAGGATTTTGACAGATGGGATCATTCGAATCAGTGTTATTGTCTTAAAATGTGTCTGATGCACCAACAGTGATGCTTCTGAGGACCTTGACAAAATCTGAAGTTGA*AATT**CCTAACTGCTGTGCCACT**
16	6p22.3	**AGTGGCACAGCAGTTAGG**AATT*TGGGTAAAGCAGATTACCCTACATAATGTGGGTGGGCCTCATATAATCAGTTGAGAGCCTTAAGAGCAAAGATTGAGGTCCCCTGAGGAAGAT*CCTGTTCGGGCGCCACTGCTAGAGATTTTCCACACTGACTAAAAGGGTCTGAGGGATCTCTAGTTACCAGAGTCACACA**ACAGACGGGCACACACTACT**
19	19p13.3	**AGTAGTGTGTGCCCGTCTGT**TGTGTGACTCTGGTAACTAGAGATCCCTCAGACCCTTTTAGTCAGTGTGGAAAATCT*CCAGCAGTGACCTTGGCTAGATCGTCTGAATGCTGGCTGACACGTCTCAAGGAACACTTAATCCTTTTCACCTTGAACCTGGTTTTTTTTGTTTTTTTTTAACACA*

		*GGGTCTCACTCTGTCACCCAGGCAGGAGTGC*AGTAGCGCAATCTCGGCTCACTGCAACCTCGGCCTCCCAAGGTGCTGGGATTACAGGCATGAGCCACCGCTCCTGGCCTACAAAAATT**CCTAACTGCTGTGCCACT**
	3q26.33	**AGTGTGTGCCCGTCTGTTGT**GTGACTCTGGTAACTAGAGATCCCTC*GCTTACAAGATTGAGTAGATATGAAACTAGCCAGCAAAAGACAAAGCATTATTTCCCAGGTACT*AATT**CCTAACTGCTGTGCCACT**
20	11q12.1	**AGTGGCACAGCAGTTAGG**AATT*AAGTGCTGCACACCAGCAACACCGACATCGCTGGGTGCTTCTGACCGGGCGCAGTGGCTCACGCCTGTAATCCCAGCACTTTGGGAGGCTGAGGCGGGCGGATCACGAGGTCAGGAGGTCGAGACCATCCTGACTAACACGGTGAAACCCTGTCTCTACTAAAAATACAAAAACATTAGCTGGGCGTGGTGGTGGACGCCTGTAGTCCCAGCTACCTGGAAGGCTGAGGCAGGAGAATGGCGTGAACCCGGGAGGCGGAGCTTGTGGGTGAGCCAAGATGGCGCCAC*TGCTAGAGATTTTCCACACTGACTAAAAGGGTCTGAGGGATCTCTAGTTACCAGAGTCACACA**ACAGACGGGCACACACTACT**
	3q29	**AGTGGCACAGCAGTTAGG**AATT*AGCCAGGCGTGGTGGTGGGTGCCTGTAATCCCACCTACTCAGGAGGCTGAGGCAGGAGAATCGCTTGAACCTGGGAGGCGGAGGTTGTAGTGAGCCGAGATGGTGCCACTGCACTCTAGCCAGGGTGACGGAGTGAGACTCTTTCTCAAAAAAAAAAAAAAGGAAAGAAGGAGAAGGACATTTAAAAAATACATTATGCTGGTCCTAGCTATTCTGGAGGCTCAGATGGGAGGATTGCTTAAGCTCAGGAGTTTGAGGCTACAGTGTGCTATGATTATGGCTGTGAGAGCCGAGATCGTGCCACTGCACTCTAGCCAGGGTGACAGAGTGAGACTCTTTCTCAAAAAAAAAAAAAAGGAAAGAAGGAGAAGGACATTTAAAAAATACATTATGCTGGTCCTAGCTATTCTGGAGGCTCAGATGGGAGGATTGCTTAAGCTCAGGAGTTTGAGGCTACAGTGTGCTATGATTATGGCTGTGAGAGCCGAGATCGTGCCACTGCACTCTARCCAGGGTGACAGARTGAGACTCTTTCTCAAAAAAAAAAAAAAGGAAAGAAGGAGAAGGACATTTAAAAAATACATTATGCTGGTCCTARCTATTCTGGAGGSTCAGATGGGAGGATTGCTTAAGCTCAGGAGTTTGAGGCTACAGTGTGCTATGATTATGGCTGTGAGAGCCGAGATCGTGCC*CCTTTCGCTTTCAAGTCCCTGTTCGGGCGCCACTGCTAGAGATTTTCCACACTGACTAAAAGGGTCTGAGGGATCTCTAGTTACCAGAGTCACACA**ACAGACGGGCACACACTACT**
22	8q24.21 (-)	**AGTAGTGTGTGCCCGTCTGT**TGTGTGACTCTGGTAACTAGAGATCCCTCAGAC*AAATCATGCTGGGCTTTTGGCCAGGTAAGGAGTTTGGTTTATAGTCTAAAAGAAATGGTGAACCATGTACAACATATTGTTTTAGAGTATATGTGCATTGTGGAATGAGTTAATCTAGCT*AATT**CCTAACTGCTGTGCCACT**
	6q13	**AGTGTGTGCCCGTCTGTTGT**GTGACTCTGGTAACTAGAGATCCCTCAGACCCTTTTAGKCAGTGTGGAAAATCTCTAGCAGTGGCGCCCGAACA*TGCTGGTCCAGCTGCGTGCTGTCGGTGGCATCTGGTGGCTCTAGTCTTGGAGGTTTCCGCGTGCTCCCATTTCTTGCCGTYTTTCTMAMTAACGCAGAGGGTCTCCCAATAGGTCTGACACCAGCCTCGTGGACCCTGAAAGATGAAGTCAGCCTCAAGTCGGC*AATT**CCTAACTGCTGTGCCACT**
	5p13.3-p13.2 (-)	**AGTGGCACAGCAGTTAGG**AATT*ATAAACAAACAAAAACACTTTGAGGGGAATGAGCTGTATGTGTGGCAAAGGCGACTGAGTTCTTCACCACCAGTGGA*CTGAGGGATCTCTAGTTACCAGAGTCACACA**ACAGACGGGCACACACTACT**
23	1q32.2 (-)	**AGTGGCACAGCAGTTAGG**AATT*AGCCGGGTGCGGTGGTGGGCACCTATAATCCCAGCTACTCAGGAGGCTGAGGCAGGAGAATCGCTTGAGCCTGGGAGGCGGAAGTTGCAGTGAGCTGAGATTG*CGCCACTGCTAGAGATTTTCCACACTGACTAAAAGGGTCTGAGGGATCTCTAGTTACCAGAGTCACACA**ACAGACGGGCACACACTACT**
	12p13.33	**AGTGGCACAGCAGTTAGG**AATT*TTGGCTGCAAGGAGGAATGGATTTTTGAAAAAAGCTATATCCGGCCGGGCGCAGTGGCTCACGTTTGTAAGCCCAGCACTTTGGGAGG*CCGAGTCCTGCGTCGAGAGAGCTCCTCTGGTTTCCCTTTCGCTTTCAAGTCCCTGTTCGGGCGCCACTGCTAGAGATTTTCCACACTGACTAAAAGGGTCTGAGGGATCTCTAGTTACCAGAGTCACACA**ACAGACGGGCACACACTACT**
24	22q13.1	**AGTGGCACAGCAGTTAGG**AATT*CCCTTCCCTTCCTTCTGCCCCTCTCATTTGGAGAGTATATGTCCTGCAGCCTTTTTTCTCCCGCAAACATACTTTCTCCTCCCCTTCTCTGGCAGGACTGGGACACTGTTGAGAGGCAGGAGGAGGAGGCCCCCAGCTGGGACGAGCTCGCAGTGATGATCCCGAGGAGGCCTCGGGAGGGGCCGAGAGCTGACAGCTCCCAAAGGGCTCCGTCTCTCCTCACCAGGTCCCCTGTGGGAGGAGATGCTGCAGGC*

		CAGAAAAAGGAGGGTGAGTCCTTCTGCCAGGTTGGTTCCCATGGTGATGGCCTGGGGCCCCCCAGATAGCCATCTCACTGGCCATTTGGGACT<br>*CTGGGCACGGCTTACTTTGGTGGCCTGAGTGTTAATAATAGTAACAGTTTGCATGTCATGATGGAGCAGCCACAATGAGTTA*TCTTTTATCTCTA<br>TCCTTTGATGCACACAATAGAGGGTTGCTACTGTATTATATAATGATCTAAGTTCTTCTGATCCTGTCTGAAGGGATGGTTGTAGCTGT<br>CCCAGTATTTGTCTACAGCCTTCTGATGTTTCTAACAGGCCAGGATTAACTGCGAATCGTTCTAGCTCCCTGCTTGCCCATACTATATGT<br>TTTAATTTATATTTTTTCTTTCCCCCTGGCCTTAACCGAATTTTTTCCCATCGCGATCTAATTCTCCCCCGCTTAATACTGACGCTCTCGC<br>ACCCATCTCTCTCCTTCTAGCCTCCGCTAGTCAAAATTTTTGGCGTACTCACCAGTCGCCGCCCCTCGCCTCTTGCCGTGCGCGCTTCAG<br>CAAGCCGAGTCCTGCGTCGAGAGAGCTCCTCTGGTTTCCCTTTCGCTTTCAAGTCCCTGTTTGGGCGCCACTGCTAGAGATTTTCCACA<br>CTGACTAAAAGGGTCTGAGGGATCTCTAGTTACCAGAGTCACACA**ACAGACGGGCACMCACTAC**
	12q24.33 (-)	**AGTGGCACAGCAGTTAGG***CCAGCCTAGAGTTCTCTCCTGAACCCTGAACCTGACCATCGAACTGTGGATTTTCCCGTGTATTTAGGTGTCAG*<br>*TCGGGTAACTCAAACTCAAAACTGGGCTTCGGCGCAAACCTGAATGTGAATCTCCCCACACTTCTACTGCTCTCTCAGTCTTTCTCATCCTAGGA*<br>*AATGGTCCTCCCCTTCCACCATGCAGCCCACGCCAAAAATATGAAGGCCATTCTTGTCACCCCCTTGCACTCCCTCTCAGCCCAGTCTATCACAA*<br>*TGGCTAGCAGGTCCTGCTGCCCCAATATATCCGGGACCTCTCCAATGACCCTCACGTCCACTACCATCCTGGTGCAGGCCACCCTTGCCAGTC*<br>*ATGTGGACTACATG*GTCGCCGCCCCTCGCCTCTTGCCGTGCGCGCTTCAGCAAGCCGAGTCCTGCGTCGAGAGAGCTCCTCTGGTTTCCC<br>TTTCGCTTTCAAGTCCCTGTTCGGGCGCCACTGCTAGAGATTTTCCACACTGACTAAAAGGGTCTGAGGGATCTCTAGTTACCAGAGTC<br>ACACA**ACAGACGGGCACACACTAC**

Table S8. Sequence of 2A-*NCF1* construct. Grey denotes *NCF2* homology arms. Orange denotes FMDV 2A. Blue denotes *NCF1* cDNA. Green denotes eGFP.

Donor template	Sequence (5'-3')
NCF1-GFP	AGGCAGAGGTTGCAGTAAGCCCAGATTGTGCCACTGCACTCCAGCCTGGGTGACAGTGAGACCCTGTCAAGGTTGGGGGTGGGGAAAGGACAGAA<br>ATTCTATTTAAGGATTCGTTGTTCAATTTCTTTGACCCTTCTTCTCTATCTGGTAACTTTTTGAAAAACATAATTTATCCTTCTTCATTTTGCTCATTAT<br>CATGTTTAAGACAGATCAATAAGATGGTTAAACCCTGTGTTCACTCTCAAACCACTTTGCAATACTGTCTTTTCCCTGTTGATCACAATTAGGGGTGG<br>GGAAGGGTGACCGATAACAAATTCTGTGTGGAATAGCCAGACAGGGTAATCTTCCTACAGTGGTTTTAGAAATCCATGTGTACTTTTCCTTTTATCA<br>GTGAATGAAGAATGGCTGGAAGGGGAGTGCAAAGGGAAGGTGGGCATTTTCCCCAAAGTTTTTGTTGAAGACTGCGCAACTACAGATTTGGAAAG<br>CACTCGGAGAGAAGTCTCTAGACACAAACAGAAAATTGTGGCACCGGTGAAACAGACTTTGAATTTTGACCTTCTCAAGTTGGCGGGAGACGTCGA<br>GTCCAACCCTGGGCCCGGCGACACCTTCATCCGGCATATCGCCCTGCTGGGCTTCGAGAAGCGGTTCGTGCCCAGCCAGCACTACGTGTACATGTTT<br>CTGGTGAAGTGGCAGGACTTGAGCGAGAAGGTGGTGTACCGGCGGTTCACCGAGATCTACGAGTTCCACAAGACCCTGAAAGAGATGTTCCCCATC<br>GAGGCCGGAGCCATCAACCCCGAGAACCGGATCATCCCCCACCTGCCTGCCCCCAAGTGGTTCGACGGCCAGAGAGCCGCCGAGAACAGGCAGGG<br>CACCCTGACCGAGTACTGCAGCACCCTGATGAGCCTGCCCACCAAGATCAGCCGGTGCCCTCATCTGCTGGATTTCTTCAAAGTGCGGCCCGACGAC<br>CTGAAGCTGCCCACCGACAACCAGACCAAGAAGCCCGAGACCTATCTGATGCCCAAGGACGGCAAGAGCACCGCCACCGACATCACCGGCCCCAT<br>CATCCTGCAGACCTATCGGGCAATCGCCAACTACGAGAAAACCAGCGGCAGCGAGATGGCCCTGAGCACCGGCGACGTGGTGGAGGTGGTGGAAA<br>AGAGCGAGAGCGGGTGGTGGTTCTGCCAGATGAAGGCCAAGCGGGGCTGGATTCCCGCCAGCTTCCTGGAACCCCTGGACAGCCCCGACGAGACC<br>GAGGACCCCGAGCCCAACTACGCCGGCGAGCCCTATGTGGCCATCAAGGCCTACACCGCCGTGGAGGGCGACGAGGTGTCCCTGCTGGAAGGCGA<br>GGCCGTGGAGGTGATCCACAAGCTGCTGGACGGTTGGTGGGTGATCCGGAAGGACGACGTGACCGGCTACTTCCCCAGCATGTACTTGCAGAAGAG

	CGGCCAGGACGTCAGCCAGGCCCAGCGGCAGATCAAGAGAGGCGCCCCTCCCAGGCGGAGCAGCATCCGGAACGCCCACAGCATCCACCAGAGAA<br>GCCGGAAGAGACTGAGCCAGGACGCCTATCGGCGGAACAGCGTGCGGTTCTTGCAGCAGCGGCGGAGGCAGGCCAGACCCGGCCCTCAGAGCCCC<br>GGCAGCCCATTGGAAGAGGAACGGCAGACCCAGCGGAGCAAGCCCCAGCCCGCCGTGCCCCCCCGACCCAGCGCTGATCTGATCCTGAACCGGTG<br>CAGCGAGAGCACCAAGCGGAAGCTGGCCAGCGCCGTGCTGTGCGTCTTCAGACACAAGCAAAAGATCGTAGCCCCTGTTAAGCAAACCTTAAACTT<br>CGATCTCCTTAAATTAGCAGGGGATGTTGAGTCCAACCCTGGGCCCCACACACGCATGGTCTCAAAAGGAGAAGAACTGTTCACAGGCGTTGTACC<br>GATCCTGGTGGAGCTGGACGGCGACGTGAACGGCCACAAGTTCAGCGTGAGCGGCGAGGGCGAGGGCGACGCCACCTACGGCAAGCTGACCCTGA<br>AGTTCATCTGCACCACCGGCAAGCTGCCCGTGCCCTGGCCCACCCTGGTGACCACCCTGACGTATGGAGTACAATGCTTCAGCAGATACCCCGACCA<br>CATGAAGCAGCACGACTTCTTCAAGAGCGCCATGCCCGAGGGCTACGTGCAGGAGAGAACCATCTTCTTCAAGGACGACGGCAATTACAAGACCA<br>GAGCCGAGGTGAAGTTCGAGGGCGACACCCTGGTGAACAGAATCGAGCTGAAGGGCATCGACTTCAAGGAGGACGGCAACATCCTGGGCCACAAG<br>CTGGAGTACAACTACAACAGCCACAACGTGTACATCATGGCCGACAAGCAGAAGAACGGCATCAAAGTGAATTTTAAAATCAGACACAACATCGA<br>GGACGGCAGCGTGCAGCTGGCCGACCACTACCAGCAGAACACCCCCATCGGCGACGGCCCCGTGCTGCTGCCCGACAACCACTACCTGAGCACCCA<br>ATCAGCGTTAAGCAAGGACCCCAACGAGAAGAGAGACCACATGGTGCTGCTGGAGTTTGTGACCGCCGCCGGCATCACCCTTGGCATGGACGAATT<br>GTACAAACCGAAGAAGAAGAGAAAAGTTCTGTAGGTCTTCGCTAGCGATGTTTCACAAACTACAAAGCTGAAGAAAATGAAGCCCTATTACTTGTT<br>TGTAAGATTTAGCACCCTTCTGCTGTATACTGTACTGAGACATTACAGTTTGGAAGTGTTAACTATTTATTCCCTGTTAAAATTTAACCTACTAGACA<br>ATGATGTGAGTACCCAGGATGATTTCCTGGGGCACAGTGGGTGAGGAGATGGGGACAGGTGAATGGAGGAGTTAGGGGAGAGGAAAAGTGGATG<br>GAAGTGTCTGGAAAGGGCACGAGAGAGTCTTCCAGGTACTGATCCTGTTTCTTGCTCTGAGTGCTAGCTAGCCAGCTGTGTTCACACTGTAAACATT<br>CATCAAGCTGTACATTTGGTGCACTTTTCTGTGTCATACCACAATAAAAAAAAACCTAGCATCTTACAAAAACAAGACACCCAAGTCCAGGCCCAA<br>GGAGTAAGTACAAATATTCCTGTTTCTGAACCATTACTGTAATTGGCTCTTAAGGCTTGAAGTAAC
GFP	AGGCAGAGGTTGCAGTAAGCCCAGATTGTGCCACTGCACTCCAGCCTGGGTGACAGTGAGACCCTGTCAAGGTTGGGGGTGGGGAAAGGACAGAA<br>ATTCTATTTAAGGATTCGTTGTTCAATTTCTTTGACCCTTCTTCTCTATCTGGTAACTTTTTGAAAAACATAATTTATCCTTCTTCATTTTGCTCATTAT<br>CATGTTTAAGACAGATCAATAAGATGGTTAAACCCTGTGTTCACTCTCAAACCACTTTGCAATACTGTCTTTTCCCTGTTGATCACAATTAGGGGTGG<br>GGAAGGGTGACCGATAACAAATTCTGTGTGGAATAGCCAGACAGGGTAATCTTCCTACAGTGGTTTTAGAAATCCATGTGTACTTTTCCTTTTATCA<br>GTGAATGAAGAATGGCTGGAAGGGGAGTGCAAAGGGAAGGTGGGCATTTTCCCCAAAGTTTTTGTTGAAGACTGCGCAACTACAGATTTGGAAAG<br>CACTCGGAGAGAAGTCTCTAGAAGACACAAGCAAAAGATCGTAGCCCCTGTTAAGCAAACCTTAAACTTCGATCTCCTTAAATTAGCAGGGGATGT<br>TGAGTCCAACCCTGGGCCCCACACACGCATGGTCTCAAAAGGAGAAGAACTGTTCACAGGCGTTGTACCGATCCTGGTGGAGCTGGACGGCGACGT<br>GAACGGCCACAAGTTCAGCGTGAGCGGCGAGGGCGAGGGCGACGCCACCTACGGCAAGCTGACCCTGAAGTTCATCTGCACCACCGGCAAGCTGC<br>CCGTGCCCTGGCCCACCCTGGTGACCACCCTGACGTATGGAGTACAATGCTTCAGCAGATACCCCGACCACATGAAGCAGCACGACTTCTTCAAGA<br>GCGCCATGCCCGAGGGCTACGTGCAGGAGAGAACCATCTTCTTCAAGGACGACGGCAATTACAAGACCAGAGCCGAGGTGAAGTTCGAGGGCGAC<br>ACCCTGGTGAACAGAATCGAGCTGAAGGGCATCGACTTCAAGGAGGACGGCAACATCCTGGGCCACAAGCTGGAGTACAACTACAACAGCCACAA<br>CGTGTACATCATGGCCGACAAGCAGAAGAACGGCATCAAAGTGAATTTTAAAATCAGACACAACATCGAGGACGGCAGCGTGCAGCTGGCCGACC<br>ACTACCAGCAGAACACCCCCATCGGCGACGGCCCCGTGCTGCTGCCCGACAACCACTACCTGAGCACCCAATCAGCGTTAAGCAAGGACCCCAACG<br>AGAAGAGAGACCACATGGTGCTGCTGGAGTTTGTGACCGCCGCCGGCATCACCCTTGGCATGGACGAATTGTACAAACCGAAGAAGAAGAGAAAA<br>GTTCTGTAGGTCTTCGCTAGCGATGTTTCACAAACTACAAAGCTGAAGAAAATGAAGCCCTATTACTTGTTTGTAAGATTTAGCACCCTTCTGCTGTA<br>TACTGTACTGAGACATTACAGTTTGGAAGTGTTAACTATTTATTCCCTGTTAAAATTTAACCTACTAGACAATGATGTGAGTACCCAGGATGATTTCC<br>TGGGGCACAGTGGGTGAGGAGATGGGGACAGGTGAATGGAGGAGTTAGGGGAGAGGAAAAGTGGATGGAAGTGTCTGGAAAGGGCACGAGAGAG<br>TCTTCCAGGTACTGATCCTGTTTCTTGCTCTGAGTGCTAGCTAGCCAGCTGTGTTCACACTGTAAACATTCATCAAGCTGTACATTTGGTGCACTTTTC

	TGTGTCATACCACAATAAAAAAAAACCTAGCATCTTACAAAAACAAGACACCCAAGTCCAGGCCCAAGGAGTAAGTACAAATATTCCTGTTTCTGA<br>ACCATTACTGTAATTGGCTCTTAAGGCTTGAAGTAAC
NCF1	AGGCAGAGGTTGCAGTAAGCCCAGATTGTGCCACTGCACTCCAGCCTGGGTGACAGTGAGACCCTGTCAAGGTTGGGGGTGGGGAAAGGACAGAA<br>ATTCTATTTAAGGATTCGTTGTTCAATTTCTTTGACCCTTCTTCTCTATCTGGTAACTTTTTGAAAAACATAATTTATCCTTCTTCATTTTGCTCATTAT<br>CATGTTTAAGACAGATCAATAAGATGGTTAAACCCTGTGTTCACTCTCAAACCACTTTGCAATACTGTCTTTTCCCTGTTGATCACAATTAGGGGTGG<br>GGAAGGGTGACCGATAACAAATTCTGTGTGGAATAGCCAGACAGGGTAATCTTCCTACAGTGGTTTTAGAAATCCATGTGTACTTTTCCTTTTATCA<br>GTGAATGAAGAATGGCTGGAAGGGGAGTGCAAAGGGAAGGTGGGCATTTTCCCCAAAGTTTTTGTTGAAGACTGCGCAACTACAGATTTGGAAAG<br>CACTCGGAGAGAAGTCTCTAGACACAAACAGAAAATTGTGGCACCGGTGAAACAGACTTTGAATTTTGACCTTCTCAAGTTGGCGGGAGACGTCGA<br>GTCCAACCCTGGGCCCGGCGACACCTTCATCCGGCATATCGCCCTGCTGGGCTTCGAGAAGCGGTTCGTGCCCAGCCAGCACTACGTGTACATGTTT<br>CTGGTGAAGTGGCAGGACTTGAGCGAGAAGGTGGTGTACCGGCGGTTCACCGAGATCTACGAGTTCCACAAGACCCTGAAAGAGATGTTCCCCATC<br>GAGGCCGGAGCCATCAACCCCGAGAACCGGATCATCCCCCACCTGCCTGCCCCCAAGTGGTTCGACGGCCAGAGAGCCGCCGAGAACAGGCAGGG<br>CACCCTGACCGAGTACTGCAGCACCCTGATGAGCCTGCCCACCAAGATCAGCCGGTGCCCTCATCTGCTGGATTTCTTCAAAGTGCGGCCCGACGAC<br>CTGAAGCTGCCCACCGACAACCAGACCAAGAAGCCCGAGACCTATCTGATGCCCAAGGACGGCAAGAGCACCGCCACCGACATCACCGGCCCCAT<br>CATCCTGCAGACCTATCGGGCAATCGCCAACTACGAGAAAACCAGCGGCAGCGAGATGGCCCTGAGCACCGGCGACGTGGTGGAGGTGGTGGAAA<br>AGAGCGAGAGCGGGTGGTGGTTCTGCCAGATGAAGGCCAAGCGGGGCTGGATTCCCGCCAGCTTCCTGGAACCCCTGGACAGCCCCGACGAGACC<br>GAGGACCCCGAGCCCAACTACGCCGGCGAGCCCTATGTGGCCATCAAGGCCTACACCGCCGTGGAGGGCGACGAGGTGTCCCTGCTGGAAGGCGA<br>GGCCGTGGAGGTGATCCACAAGCTGCTGGACGGTTGGTGGGTGATCCGGAAGGACGACGTGACCGGCTACTTCCCCAGCATGTACTTGCAGAAGAG<br>CGGCCAGGACGTCAGCCAGGCCCAGCGGCAGATCAAGAGAGGCGCCCCTCCCAGGCGGAGCAGCATCCGGAACGCCCACAGCATCCACCAGAGAA<br>GCCGGAAGAGACTGAGCCAGGACGCCTATCGGCGGAACAGCGTGCGGTTCTTGCAGCAGCGGCGGAGGCAGGCCAGACCCGGCCCTCAGAGCCCC<br>GGCAGCCCATTGGAAGAGGAACGGCAGACCCAGCGGAGCAAGCCCCAGCCCGCCGTGCCCCCCCGACCCAGCGCTGATCTGATCCTGAACCGGTG<br>CAGCGAGAGCACCAAGCGGAAGCTGGCCAGCGCCGTGTAGGTCTTCGCTAGCGATGTTTCACAAACTACAAAGCTGAAGAAAATGAAGCCCTATTA<br>CTTGTTTGTAAGATTTAGCACCCTTCTGCTGTATACTGTACTGAGACATTACAGTTTGGAAGTGTTAACTATTTATTCCCTGTTAAAATTTAACCTACT<br>AGACAATGATGTGAGTACCCAGGATGATTTCCTGGGGCACAGTGGGTGAGGAGATGGGGACAGGTGAATGGAGGAGTTAGGGGAGAGGAAAAGT<br>GGATGGAAGTGTCTGGAAAGGGCACGAGAGAGTCTTCCAGGTACTGATCCTGTTTCTTGCTCTGAGTGCTAGCTAGCCAGCTGTGTTCACACTGTAA<br>ACATTCATCAAGCTGTACATTTGGTGCACTTTTCTGTGTCATACCACAATAAAAAAAAACCTAGCATCTTACAAAAACAAGACACCCAAGTCCAGGC<br>CCAAGGAGTAAGTACAAATATTCCTGTTTCTGAACCATTACTGTAATTGGCTCTTAAGGCTTGAAGTAAC

Table S9. Primers used for NGS verification of CHANGE-seq off-target sites.

Site	Genomic Coordinate	Site sequence	Forward primer	Reverse primer	Annealing temperature (°C)
ON	chr1:183556116-183556139	AAGCACTCGGAGAGAAGTCTAGG	CTTTCCCTACACGACGCTCTTCCGATCTACAGTATACAGCAGAAGGGTGC	GGAGTTCAGACGTGTGCTCTTCCGATCTTGTGGAATAGCCAGACAGGG	62

OT1	chr16:2169909-2169932	AAGCACCAGGAGAGAAGCCTTGG	CTTTCCCTACACGACGCTCTTCCGATCTTGAGCCACCGACAAGGGA	GGAGTTCAGACGTGTGCTCTTCCGATCTACTGGTTTGCCTCTGTGCTC	59 (less efficient PCR)
OT2	chr5:107756848-107756872	TAGAACTCGGAGAGGAGGTCTAGG	CTTTCCCTACACGACGCTCTTCCGATCTCCTAGTCTTAGGTAGTCTGTTATAGC	GGAGTTCAGACGTGTGCTCTTCCGATCTATGTTCCCTGGGTAAATGGC	62
OT3	chr21:25516315-25516338	CAGCTCTCGGAGAGAAGTCTGGG	CTTTCCCTACACGACGCTCTTCCGATCTGTCCCCTTTGCCAAC	GGAGTTCAGACGTGTGCTCTTCCGATCTTGATTTAAAAATTATCTTGAGACAG	57
OT4	chr6:15827983-15828005	AGGCACCCAGAGGAAGTCTTGG	CTTTCCCTACACGACGCTCTTCCGATCTCTCTCAGTGAGGCTGACCAC	GGAGTTCAGACGTGTGCTCTTCCGATCTCTGTGACATGAAGCCCCCAG	68
	chr22:46578343-46578367	CAGCACTCGAGAGAGGAGTCTGGG	CTTTCCCTACACGACGCTCTTCCGATCTACATGCCAAAAAGGAGGTGC	GGAGTTCAGACGTGTGCTCTTCCGATCTCATTTTGAACCCCAACCCAGG	66 (PCR not working)

Table S10. Raw data of NGS deep sequencing for verification of CHANGE-seq off-target sites.

			On-target			Off-target 1			Off-target 2			Off-target 3			Off-target 4		
	**Cell type**	**Treatment**	**Aligned reads**	**Reads with indel**	**Indel%**	**Aligned reads**	**Reads with indel**	**Indel%**	**Aligned reads**	**Reads with indel**	**Indel%**	**Aligned reads**	**Reads with indel**	**Indel%**	**Aligned reads**	**Reads with indel**	**Indel%**
1	HD hCD34+	Cas9-sgRNA NCF2	3973	658	16.56%	17910	47	0.26%	3182	0	0.00%	na	na	na	1738	0	0.00%
2.1	HD hCD34+	Cas9-sgRNA NCF2 + AAV 2A-NCF1	4215	150	3.56%	na	na	na	2551	0	0.00%	2943	43	1.46%	3125	1	0.03%
2.2	HD hCD34+	Cas9-sgRNA NCF2 + AAV 2A-NCF1	6511	253	3.89%	1227	0	0.00%	3254	0	0.00%	6419	132	2.06%	3707	0	0.00%
4	HD hCD34+	Unedited	4421	37	0.84%	1884	0	0.00%	2278	2	0.09%	3960	0	0.00%	1200	0	0.00%
5	HD hCD34+	Unedited	10008	32	0.32%	105	0	0.00%	3419	0	0.00%	8376	0	0.00%	3756	0	0.00%
6	HD hCD34+	Unedited	12600	16	0.13%	1796	0	0.00%	3430	0	0.00%	7051	0	0.00%	3825	0	0.00%
7	HD hCD34+	Cas9-sgRNA NCF2	7513	1078	14.35%	243	0	0.00%	3090	0	0.00%	5661	43	0.76%	2703	0	0.00%
8	HD hCD34+	Unedited	6628	7	0.11%	381	0	0.00%	3502	0	0.00%	5822	0	0.00%	3246	0	0.00%
9	HD hCD34+	Cas9-sgRNA NCF2	5871	1493	25.43%	414	0	0.00%	2957	1	0.03%	5118	51	1.00%	3483	0	0.00%
10	HD hCD34+	Unedited	6494	29	0.45%	1365	3	0.22%	3750	1	0.03%	6857	1	0.01%	2987	0	0.00%
11	HD hCD34+	Cas9-sgRNA NCF2	8392	2018	24.05%	1967	0	0.00%	4065	1	0.02%	1280	8	0.63%	5692	0	0.00%
12.1	p47-CGD hCD34+	AAV 2A-NCF1	1645	31	1.88%	669	2	0.30%	1364	0	0.00%	470	0	0.00%	2492	0	0.00%
12.2	p47-CGD hCD34+	AAV 2A-NCF1	10399	58	0.56%	na	na	na	3193	0	0.00%	635	1	0.16%	3350	0	0.00%
13.1	p47-CGD hCD34+	Cas9-sgRNA NCF2	7194	2738	38.06%	na	na	na	2158	0	0.00%	4415	52	1.18%	5104	1	0.02%
13.2	p47-CGD hCD34+	Cas9-sgRNA NCF2	7943	3209	40.40%	1868	1	0.05%	2438	1	0.04%	6081	97	1.60%	5110	0	0.00%
14.1	p47-CGD hCD34+	Cas9-sgRNA NCF2 + AAV 2A-NCF1	4511	520	11.53%	2106	0	0.00%	1953	0	0.00%	627	2	0.32%	3831	0	0.00%
14.2	p47-CGD hCD34+	Cas9-sgRNA NCF2 + AAV 2A-NCF1	8677	402	4.63%	na	na	na	988	0	0.00%	582	0	0.00%	148	0	0.00%
15	PLB-985 *NCF1* ΔGT	Unedited	7046	16	0.23%	na	na	na	2470	0	0.00%	5423	0	0.00%	2748	0	0.00%
16	PLB-985 *NCF1* ΔGT	Unedited	5450	19	0.35%	2002	0	0.00%	1340	0	0.00%	2017	0	0.00%	835	0	0.00%
17.1	PLB-985 *NCF1* ΔGT	Cas9-sgRNA NCF2	6447	3790	58.79%	425	0	0.00%	2553	8	0.31%	4924	403	8.18%	3109	0	0.00%
17.2	PLB-985 *NCF1* ΔGT	Cas9-sgRNA NCF2	5916	3447	58.27%	1758	1	0.06%	2521	7	0.28%	3785	347	9.17%	4518	0	0.00%
18	PLB-985 *NCF1* ΔGT	Cas9-sgRNA NCF2 + AAV 2A-NCF1	3818	1728	45.26%	724	1	0.14%	2235	9	0.40%	5959	631	10.59%	3833	1	0.03%
19	PLB-985 *NCF1* ΔGT	Cas9-sgRNA NCF2 + AAV 2A-NCF1	6351	2225	35.03%	2649	3	0.11%	1700	0	0.00%	3464	336	9.70%	2015	0	0.00%
20	PLB-985 *NCF1* ΔGT	Cas9-sgRNA NCF2 + IDLV 2A-NCF1	6240	2912	46.67%	930	9	0.97%	1599	18	1.13%	5656	537	9.49%	2383	0	0.00%
21	PLB-985 *NCF1* ΔGT	Cas9-sgRNA NCF2 + IDLV 2A-NCF1	2971	1224	41.20%	275	1	0.36%	766	1	0.13%	1740	95	5.46%	1447	0	0.00%
22	HEK293	Control untreated	17910	47	0.26%	1921	0	0.00%	2124	0	0.00%	3717	0	0.00%	3616	0	0.00%

Table S11. Raw data of CHANGE-seq results.

#Chromosome	Genomic Coordinate	Nuclease_Read_Count	Strand	Site_Sequence	Site_Substitution_Number	Site_Sequence_Gaps_Allowed	Realigned_Target_Sequence
chr1	chr1:183556116-183556139	1990	-	AAGCACTCGGAGAGAAGTCTAGG	0		none
chr16	chr16:2169909-2169932	372	+	AAGCACCAGGAGAGAAGCCTTGG	3		none
chr5	chr5:107756848-107756872	122	+			TAGAACTCGGAGAGGAGGTCTAGG	AAGCACTCGGAGAGAAG-TCTNGG
chr22	chr22:46578343-46578367	58	+			CAGCACTCGAGAGAGGAGTCTGGG	AAGCACTCG-GAGAGAAGTCTNGG
chr21	chr21:25516315-25516338	54	+	CAGCTCTCGGAGAGAAGTCTGGG	2		none
chr6	chr6:15827983-15828005	48	+			AGGCACCCAGAG-GAAGTCTTGG	AAGCACTCGGAGAGAAGTCTNGG
chr13	chr13:42807847-42807871	42	-			TAGCACTCAGTAGAGAAGTCTGGG	AAGCACTCGG-AGAGAAGTCTNGG
chr12	chr12:40598403-40598426	30	+	AAACACTGGGAGAGGAGTTTGGG	4		none
chr22	chr22:49208293-49208315	30	-			AGGCACCCAGAGA-AAGTCTTGG	AAGCACTCGGAGAGAAGTCTNGG
chr3	chr3:136501538-136501561	30	+	AGACACTAGGAGAGAAGTCTGGA	4		none
chr16	chr16:77829361-77829384	24	-	TGAAACTCGGACAGAAGTCTTGG	5	ATGAAACTCGGACAGAAGTCTTGG	AAGCA-CTCGGAGAGAAGTCTNGG
chr11	chr11:57478391-57478414	22	+	GAGCACTTGGAGAGAAGTTTGGT	4	GAGCACTTGGAGAGAAGT-TTGG	AAGCACTCGGAGAGAAGTCTNGG
chr1	chr1:204462322-204462345	16	-	CAGCCCACGGAGAGAAGTCCTAG	5	CAGCCCACGGAGAGAAGTCCTAGG	AAGCACTCGGAGAGAAGTC-TNGG
chr17	chr17:4531821-4531844	14	+	TCCCACCCGGAGAGAAGCCTGGG	5		none
chr5	chr5:152113531-152113554	14	+	CAGCACCCAGAGAGAAGCCTGGG	4		none
chr14	chr14:98954426-98954449	10	-	AGCCACCCGGAGAGAAGTTAGGC	6	AGCCACCCGGAGAGAAGT-TAGG	AAGCACTCGGAGAGAAGTCTNGG
chr18	chr18:8290133-8290156	10	+	AAGCACCAGAAGAGAAGTCTGAG	4		none
chr10	chr10:18783649-18783671	8	-			AAGCCCTAGAAGAGAAG-CTAGG	AAGCACTCGGAGAGAAGTCTNGG
chr10	chr10:70782490-70782512	8	+			ATGCACTCAG-GAGTAGTCTTGG	AAGCACTCGGAGAGAAGTCTNGG
chr14	chr14:91292959-91292982	8	-	AGGCACCCAGAGAGAAGGCTGGA	5		none
chr15	chr15:96449784-96449807	8	-	AACCACTCGAAGAGGAAGCCTGG	6	AACCACTCGAAGAGGAAGCCTGGG	AAGCACTCGGAGAG-AAGTCTNGG
chr2	chr2:71891430-71891453	8	+	CAGCACCCGCAGAGAAGCCCTGG	5		none
chr7	chr7:103010789-103010812	8	+	AAGCACACAGACAGAAGATTAGG	5		none
chr16	chr16:54141943-54141966	6	-	TAGCCCCAGGAGAGAAGTCTTCT	6		none
chr1	chr1:160349626-160349649	6	-	GTACACTAGGAGAGAAGCCTGAG	6		none
chr22	chr22:17993655-17993678	6	-	AAGCACTGGGAGGAGAGTGTGGG	5		none
chr22	chr22:41482320-41482342	6	+			TAGCACAGGGAGA-AAGTCTCGG	AAGCACTCGGAGAGAAGTCTNGG
chr2	chr2:5938617-5938640	6	-	AAAGCCTGGGAGAGAAGCCTGGG	5	AAGC-CTGGGAGAGAAGCCTGGG	AAGCACTCGGAGAGAAGTCTNGG
chr3	chr3:52678520-52678543	6	+	AAGAGCTCACAGAGAAGTCTGCC	6		none
chr7	chr7:159212022-159212044	6	-			TAGC-CTTGGAGAGAAGTCAGGG	AAGCACTCGGAGAGAAGTCTNGG
chr8	chr8:32514105-32514128	6	-	AAGAATTTGCAGACAAGTCTTGG	5		none
chr8	chr8:6648645-6648668	6	+	TAGCACTCAGGCAGAAGTCTTCT	6		none
chr9	chr9:136908165-136908188	6	+	GAGTGCCCGGAGAGAAGCCTGAG	6		none
chrX	chrX:95644230-95644253	6	+	AGGCACTGGGAAAAAAGTCCTGT	6		none
chr12	chr12:132714738-132714761	4	-	AAGAGCATGGAGAGAAGACAAGG	6		none
chr12	chr12:4535130-4535153	4	+	GAGCACTGAGAGAGAAGTTAGGA	6	GAGCACTGAGAGAGAAGT-TAGG	AAGCACTCGGAGAGAAGTCTNGG
chr12	chr12:57782849-57782872	4	+	AGCTACCCGGTGAGAAGTCCTGG	6		none
chr13	chr13:40563272-40563295	4	+	ACAAACTGGGAGAGAAGTCTTCT	6		none
chr17	chr17:42964460-42964483	4	+	GCGCACGCGCAGAGAAGCCTGGG	5		none
chr1	chr1:161074585-161074608	4	-	AAGCACTGGAGCAGAAGTCCTTG	6		none
chr20	chr20:61582528-61582551	4	-	AAGCACTGGGAGGGAAGTCTACA	4		none
chr2	chr2:126915783-126915805	4	-			ACGCACTAGGGG-GAAGTCTAGG	AAGCACTCGGAGAGAAGTCTNGG
chr2	chr2:138915425-138915447	4	-			AAGCCCTCGGACAGA-GTCTTGG	AAGCACTCGGAGAGAAGTCTNGG
chr2	chr2:234373478-234373501	4	+	AAGCCCCCGGAGAGAAGCCTGAG	4		none
chr4	chr4:105006167-105006190	4	-	ATGCACACGGAGTGAAGTCTTAG	4		none
chr4	chr4:636894-636917	4	+	AACCCCCCGGAGGGAAGTCTCAG	5		none
chr5	chr5:41354218-41354241	4	-	TAGCATCCAGAGAGAAGTCCGGG	5		none
chr6	chr6:110553723-110553746	4	-	CTGCACACGGAGAGAACTCTGGG	4		none

chr6	chr6:163632363-163632386	4	-	AAGTGCTCGTAAAGAAGGCTTGG	5		none
chr7	chr7:74306877-74306899	4	+			AGCCACTCGGAGA-AAGTCTGGG	AAGCACTCGGAGAGAAGTCTNGG
chr9	chr9:109483005-109483028	4	+	ATGCACAGGGAGAAAAGTCTTGA	5		none
chr9	chr9:129829378-129829401	4	-	AGCCCCTCGGAGAGCAGTCTTGC	5	AAGCCCCTCGGAGAGCAGTCTTGC	AAGCAC-TCGGAGAGAAGTCTNGG
chr9	chr9:88141639-88141662	4	+	AAGCCCTGGGAGAGAAGTCTGGT	3		none

Supplemental Figures

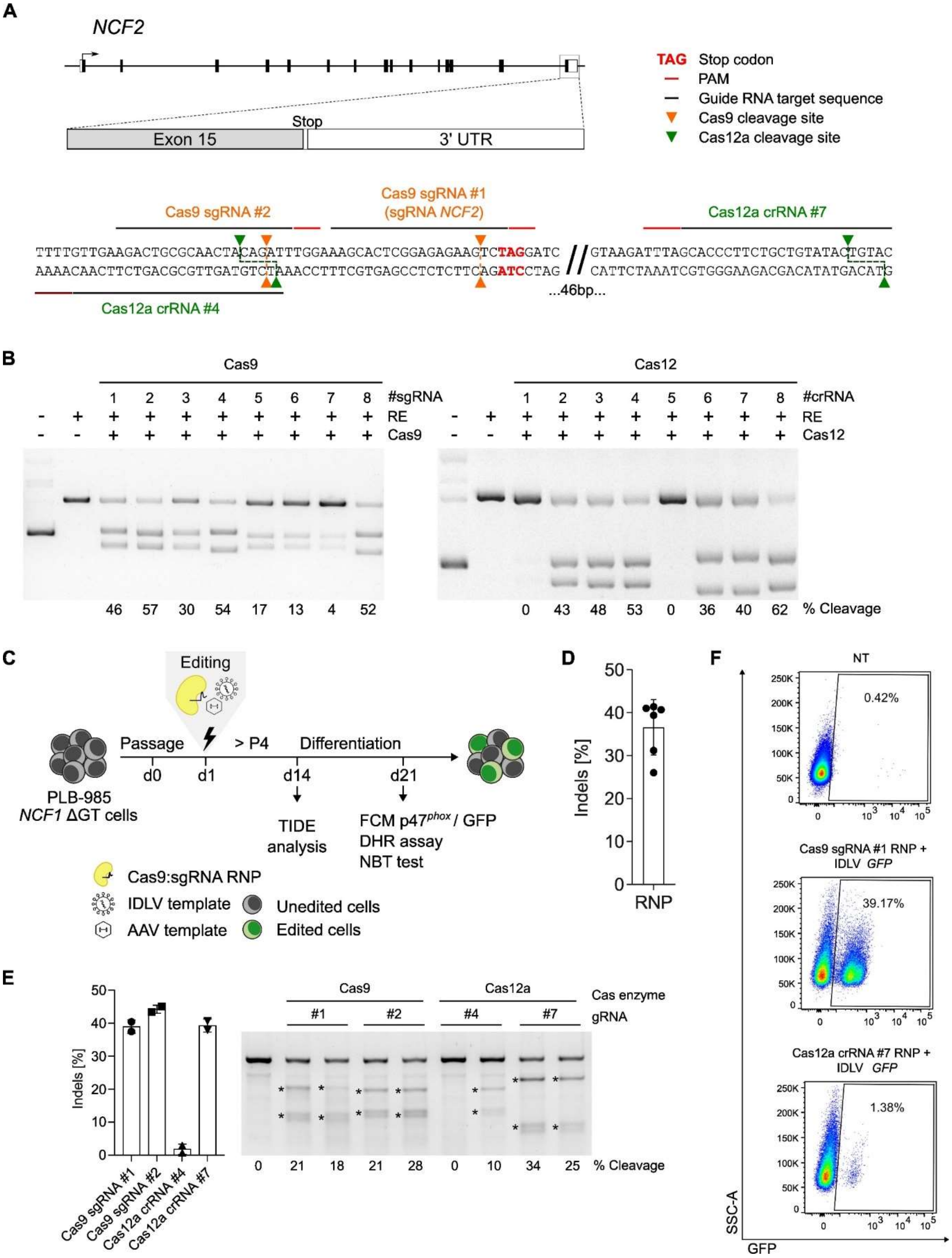

Figure S1. CRISPR-Cas gRNA design and screening *in vitro* and in cells.

(**A**) Position of Cas9 and Cas12a targeting the *NCF2* locus at exon 15 and the 3' UTR. Red marks the stop codon of *NCF2*; grey line, target sequence; red line, PAM sequence; arrows, cleavage site

(blunt/staggered cut) of the Cas9 or Cas12a target sequence (full list of gRNA sequences are shown in **Table S1**). (**B**) *In vitro* cell-free cleavage assay of Cas9 sgRNAs and Cas12a crRNAs. The % cleavage resulting from RNP activity is shown under the graph for each reaction. Different Cas-sgRNA targeting the *NCF2* stop codon region were tested. Cas9 sgRNA #1 (subsequently referred to as sgRNA *NCF2*) was determined to have a high cleavage activity (45.8%) in an *in vitro* cell-free assay (**Figure S1B**) and in mammalian cells (39.0 ± 2.05%) by TIDE analysis (**Figure S1E**), with the Cas9 cleavage site located closest to the intended knock-in position (translational stop codon of *NCF2*). RE, restriction enzyme used to linearize template DNA. (**C**) Experimental workflow of the knock-in treatment and downstream analyses. (**D**) Indel frequency of sgRNA *NCF2* for experiments shown in **Figure 1D and 1E**. (**E**) Left: indel frequency of respective gRNAs measured by TIDE analysis PLB-985 *NCF1* ΔGT cells treated with Cas9 RNP (n=2). Right: results of the T7EI assay with the same samples (n=2 shown in 2 lanes). Asterisks mark bands of heteroduplex products cleaved by T7EI and cleavage efficiency in % is shown under the graph for each reaction. (**F**) Flow cytometry analysis of GFP expression in PLB-985 *NCF1* ΔGT cells upon treatment with Cas9 sgRNA #1 RNP + IDLV *GFP* and Cas12a crRNA #7 RNP + IDLV *GFP*. Knock-in efficiency of SpCas9 RNP and AsCpf1/Cas12a RNP was compared. Although cleavage efficiency of Cas9 sgRNA #1 and Cas12a crRNA #7 was similar, the knock-in efficiency was low when the Cas12a system was used. NT, non-treated control.

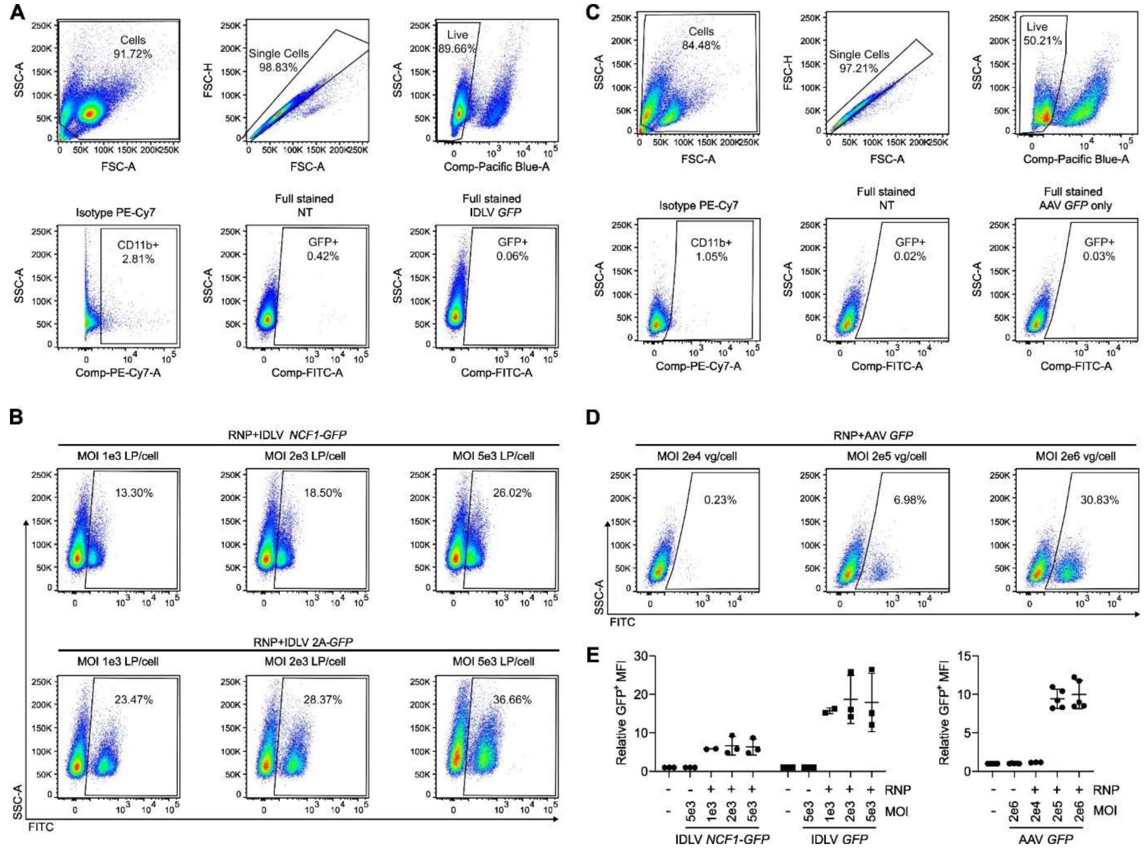

Figure S2. Gating strategy of flow cytometry analyses in PLB-985 cells shown in Figure 1D.

(**A, C**) Gating strategy for determination of GFP expression in live, CD11b-positive cells. Gating of CD11b-positive cells is based on the fluorescence minus one (FMO) control, with the addition of the isotype control PE-Cy7 antibody. Gating of the GFP-positive population is based on fully stained PLB-985 *NCF1* ΔGT cells. Gating strategy for RNP+IDLV *GFP* treated cells is presented in **A**, while for RNP + AAV *GFP* treated cells is presented in **C**. NT, non-treated control. (**B**) Representative flow cytometry plots of cells treated with RNP + IDLV *NCF1-GFP* and RNP + IDLV *GFP* at the respective MOI. (**D**) Representative flow cytometry plots showing cells treated with RNP+AAV *GFP* with the indicated MOI. (**E**) Relative GFP-positive MFI of corresponding samples in **Figure 1D;** n=2-5, data are shown as mean ± SD. MOI is denoted as lentiviral particle/cell (LP/cell) for IDLVs, or vector genomes/cell (vg/cell) for AAVs.

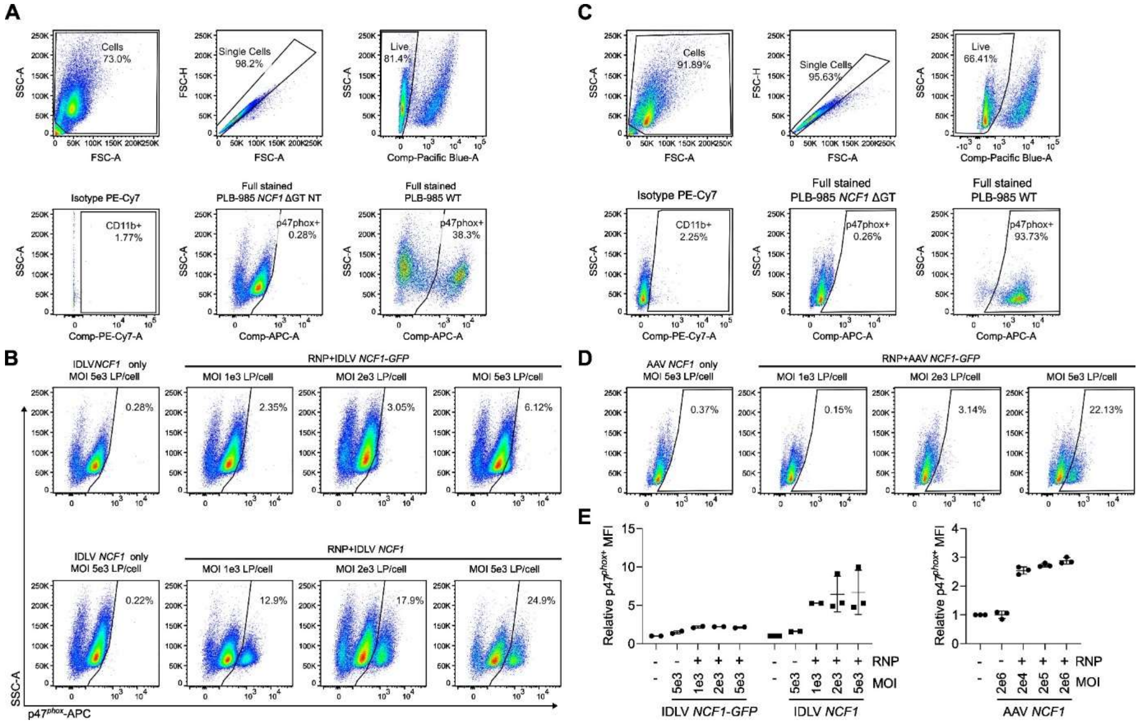

Figure S3. Gating strategy of flow cytometry analyses in PLB-985 cells shown in Figure 1E.

(**A, C**) Gating strategy for analysis of p47*phox* expression in live, CD11b-positive cells. Gating of CD11b-positive cells is set on FMO control, with the addition of the isotype control PE-Cy7 antibody. Gating of the p47*phox*-positive population within the CD11b-positive population is based on fully stained PLB-985 *NCF1* ΔGT cells. Gating strategy for cells treated with RNP + IDLV *NCF1* is presented in **A**, and for cells treated with RNP + AAV *NCF1* in **C**. NT, non-treated control. (**B**) Representative flow cytometry showing cells treated with RNP + IDLV *NCF1-GFP* and RNP + IDLV *NCF1* at the indicated MOIs. (**D**) Representative flow cytometry plots showing cells treated with RNP + AAV *NCF1* at the indicated MOIs. (**E**) Relative p47*phox*-positive MFI of corresponding samples in **Figure 1E;** n=2-3, data are shown as mean ± SD. MOI is denoted as LP/cell for IDLVs, or vg/cell for AAVs.

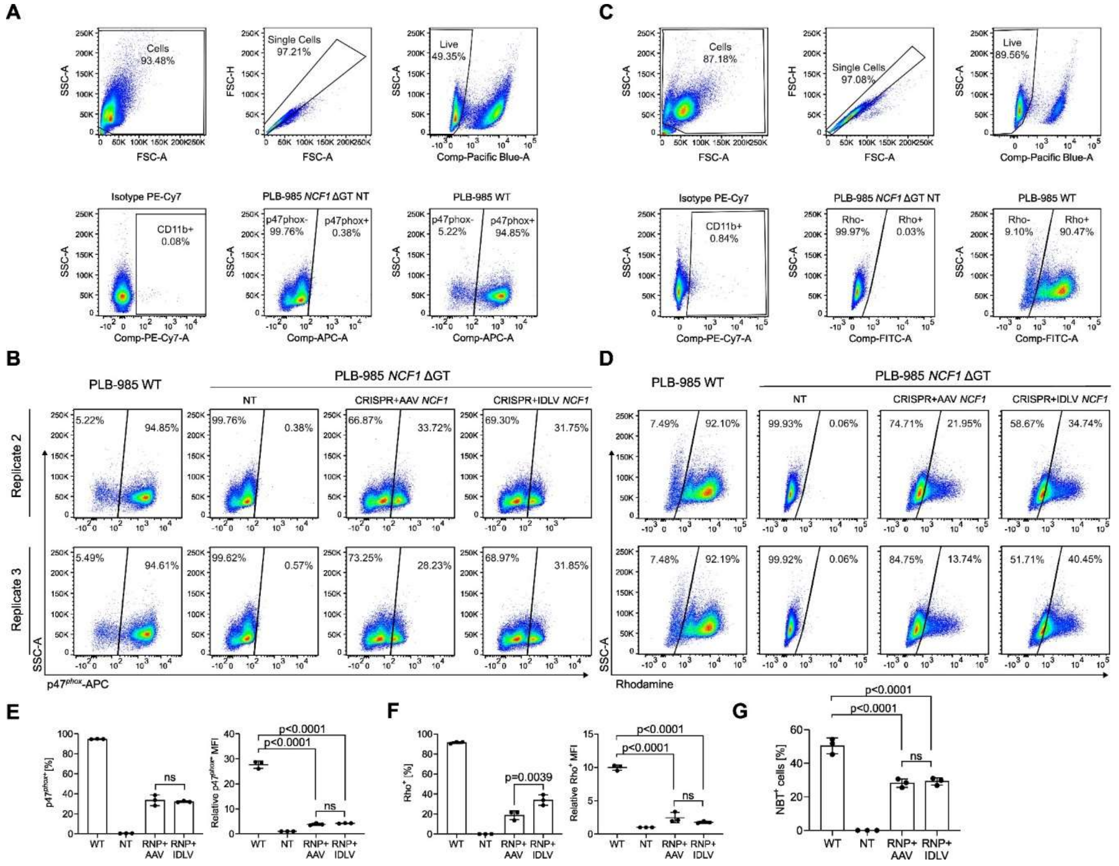

Figure S4. Gating strategy of flow cytometry analyses in PLB-985 cells shown in Figure 1F and 1G, and quantification of NBT test shown in Figure 1H

(**A**) Gating strategy in **Figure 1F** to determine p47^{phox} positive cells in live, CD11b-positive population. FMO control plus isotype control PE-Cy7 antibody were used for gating of CD11b-positive cells, followed by gating of the p47^{phox}-positive population in fully stained PLB-985 *NCF1* ΔGT cells. (**B**) Flow cytometry plots of p47^{phox}-positive populations in replicates 2 and 3 of corresponding samples in **Figure 1F**. Replicate 1 is shown as a representative plot in **Figure 1F**. (**C**) Gating strategy in **Figure 1G** to determine Rho-positive cells in live, CD11b-positive population. Gating of Rho-positive cells within the CD11b-positive population is set on fully stained, PMA-stimulated, non-treated PLB-985 *NCF1* ΔGT cells as a negative control. (**D**) Flow cytometry plots of Rho-positive populations in replicates 2 and 3 of corresponding samples in **Figure 1G**. (**E**) Bar graphs show summary data of % p47^{phox}-positive cells and relative p47^{phox}-positive MFI gated on live, CD11b-positive cells of samples in **Figure 1F**. **(F)** Bar graphs show summary data of % Rho-positive cells and relative Rho positive MFI gated on live, CD11b-positive cells of samples in **Figure 1G** (In **E** and **F**, relative MFI is calculated as the ratio of MFI-positive population over the negative population of the same sample; n=3; ns, non-significant; data are shown as mean ± SD). (**G**) Quantification of the NBT test results in

Figure 1H. Statistical analysis performed with one-way ANOVA followed by Sidak's multiple comparisons test.

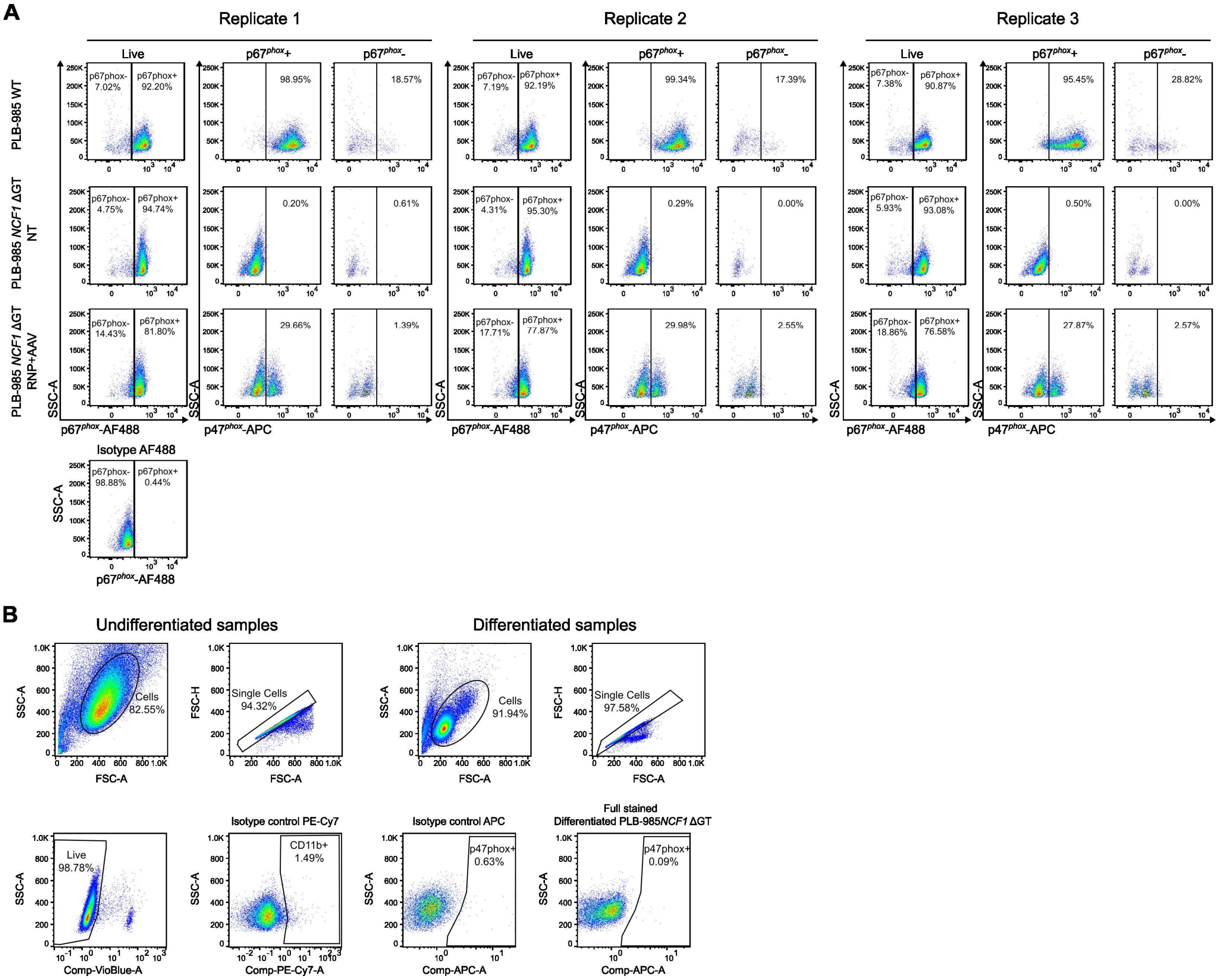

Figure S5. Gating strategy of flow cytometry analyses in PLB-985 cells shown in Figure 2A and 2B.

(**A**) Flow cytometry analyses supplementary to **Figure 2A** to determine p67^{phox} and p47^{phox} expression in three independent replicates of PLB-985 WT cells, non-treated PLB-985 *NCF1* ΔGT cells, and RNP + AAV *NCF1* treated PLB-985 *NCF1* ΔGT cells upon knock-in into *NCF2*. The plots show live cells, gated on both p67^{phox} positive and p67^{phox} negative populations that express p47^{phox}. All three replicates of cells treated with RNP + AAV *NCF1* showed p47^{phox} expression that was mainly restricted to the p67^{phox}-positive population, with a minimal leakage into the p67^{phox}-negative population (up to 2.6%). The gating of the p67^{phox} population was determined by FMO control, stained with isotype control AF488 antibody. (**B**) Gating strategy supplementary to **Figure 2B** to determine the p47^{phox}-positive population in undifferentiated cells and in myeloid-differentiated PLB-985 *NCF1* ΔGT cells, gated on

live and CD11b-positive cells. FMO controls, plus isotype control PE-Cy7 and APC antibodies were used to gate CD11b- and p47^{phox}-positive cells. NT, non-treated control.

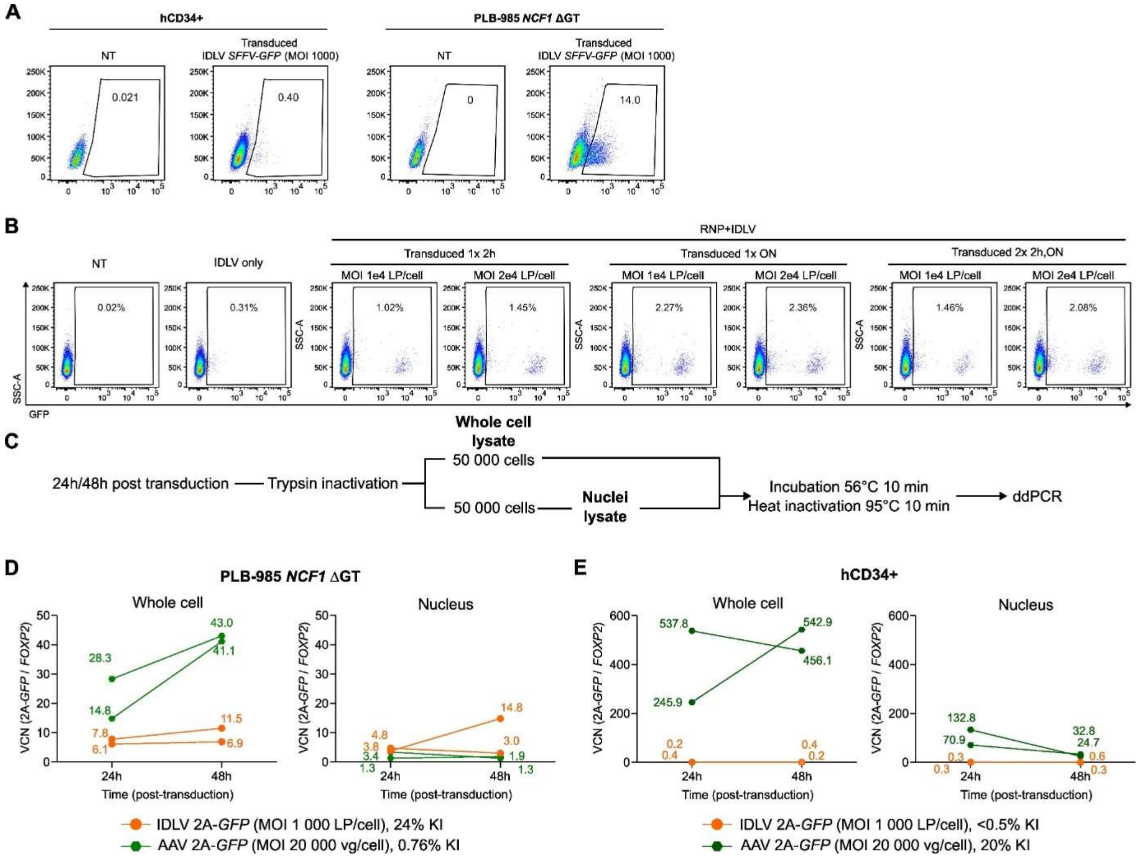

Figure S6. IDLV knock-in in human CD34+ cells is limited by transduction efficiency and cell-type tropism.

(**A**) Comparison of transduction efficiency in human CD34+ (hCD34+) cells and PLB-985 *NCF1* ΔGT cells. Cells were transduced with IDLV *SFFV-GFP* at an MOI of 1 000 LP/cell. Flow cytometry analysis of GFP expression was performed 24 hours after transduction. Transduction efficiency was low in hCD34+ cells, as compared to PLB-985 *NCF1* ΔGT cells (n=1). (**B**) Determination of knock-in efficiency in hCD34+ cells treated with RNP + IDLV *SFFV-GFP*. Cells were electroporated with RNPs targeting the *NCF2* locus, as described in 'Materials and methods', followed by transduction with IDLV *SFFV-GFP* at an MOI 1x10^4 or 2x10^4 LP/cell: one time for 2h, one time overnight (ON), or two times for 2h and overnight. After treatment, cells were differentiated towards myeloid lineages for 2 weeks and GFP expression was analysed by flow cytometry. At all tested transduction conditions, the IDLV knock-in efficiency in hCD34+ cells was low, not exceeding 2.4%. (**C**) Scheme of sample preparation for ddPCR-based determination of the vector copy number (VCN). Cells were transduced with IDLV 2A-*GFP,* or AAV 2A-*GFP* for 24h or 48h. Prior to nuclei isolation, cells were washed in

PBS twice, and treated with trypsin to remove remaining viral vector particles bound to the cell surface. Nuclei isolation was performed with Monarch® gDNA nuclei prep and lysis buffer pack (NEB, USA), according to the manufacturer's instructions. Two fractions of whole cells (including nuclei and cytoplasm) or nuclei only were isolated and subjected to DNA isolation by Buffer K lysis (as described in 'Materials and methods'). Isolated DNA samples contained host gDNA and AAV genomes. (**D, E**) VCN of 2A-*GFP* determined by ddPCR, normalized to two copies of *FOXP2* in the host genome of (**D**) PLB-985 *NCF1* ΔGT cells and (**E**) hCD34+ cells. PLB-985 *NCF1* ΔGT cells and hCD34+ cells were transduced with IDLV *GFP* or AAV *GFP* at the indicated MOI for 24h or 48h prior to DNA isolation from whole cells or nuclei. Each data point of similar colour represents replicates (n=2). Transduction of PLB-985 *NCF1* ΔGT cells with IDLVs was efficient, with the highest VCN of 11.5 copies detected in the whole cell samples, and 3.4 copies in the nuclei samples. In hCD34+ cells, there were less than 0.6 copies detected in both whole cell and nuclei samples, suggesting that either the IDLVs used in our experiments (D64V) have a low tropism to hCD34+ cells, or reverse transcription of lentiviral RNA to DNA was impaired and/or delayed. This observation was contrary to the previously published data showing that IDLVs outperformed AAVs in template delivery.[10] High VCN in AAV-transduced hCD34+ cells (up to 543 copies in the whole cell samples) indicated that AAV serotype 6 is highly efficient in targeting hCD34+ cells, while it is less active in PLB-985 cells (up to 43 copies in whole cell samples). When comparing nuclear transport of vector DNA, IDLVs were more efficiently transported into the nucleus than AAVs (*e.g.,* 7.8 copies in the whole cell samples, and 4.8 copies in the nuclei samples at 24h post transduction). A higher MOI was needed for AAV transduction as the nuclear transport was poor, suggesting the high particle-to-infectivity ratio.

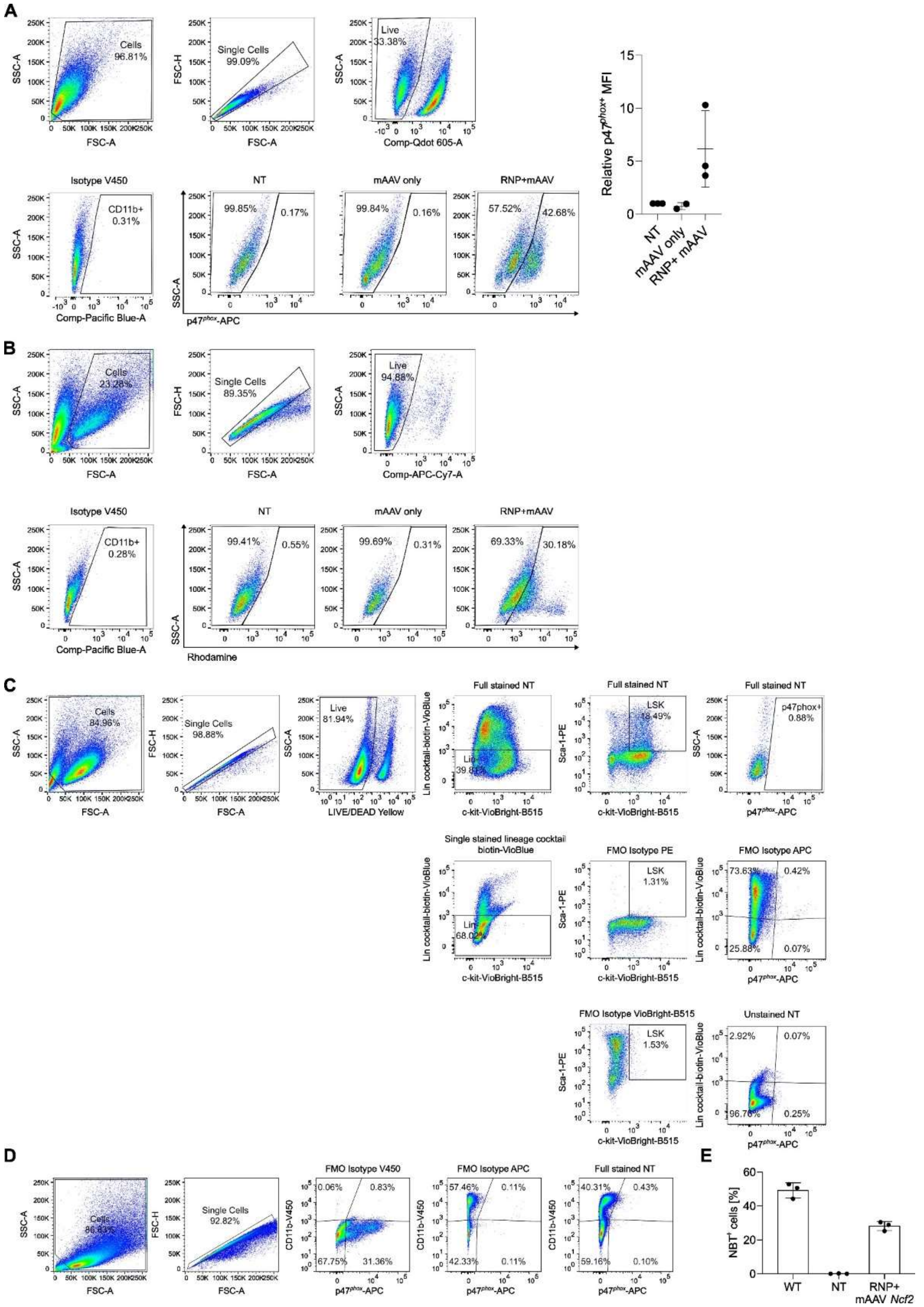

Figure S7. Gating strategy of flow cytometry analyses in lineage-negative mouse hematopoietic stem cells supplementary to Figure 3B, 3C, 3E and 3F.

(**A**) Expression of p47^{phox} was gated on live, CD11b-positive cells. FMO control, stained with isotype control V450 antibody, was used for CD11b gating. Gating of p47^{phox}-positive cells was set on fully stained p47^{phox}-deficient mouse hematopoietic stem cells (mHSPCs) as negative control. The relative p47^{phox}-positive MFI of corresponding samples is shown in **Figure 3B**; n=2-3, data presented as mean

± SD. (**B**) Gating strategy determining Rho-positive cells in the live, CD11b-positive population. CD11b gating was set on FMO control plus isotype control V450 antibody. Rho positive cells were gated on fully stained and PMA-stimulated, non-treated p47*phox*-deficient mHSPCs. (**C**) Gating strategy supplementary to **Figure 3E**, determining p47*phox* expression in LSK (Lin- Sca-1+ c-Kit+) cells in mHSPCs before differentiation. Gating was done on the live and Lin- population, followed by Sca-1 versus c-Kit, gated on FMO controls, stained with isotype control PE and VioBright-B515, respectively. Within the Lin- population, p47*phox* expression was determined on fully stained non-treated p47*phox*-deficient mHSPCs as negative control. Alternative quadrant gating with Lin- versus p47*phox* was performed to check for transgene expression in the undifferentiated population. (**D**) Gating strategy supplementary to **Figure 3F** to determine p67*phox* versus p47*phox* expression on live cells, after myeloid differentiation of p47*phox*-deficient mHSPCs. (**E**) Quantification of formazan-positive cells in NBT test shown in **Figure 3D**.

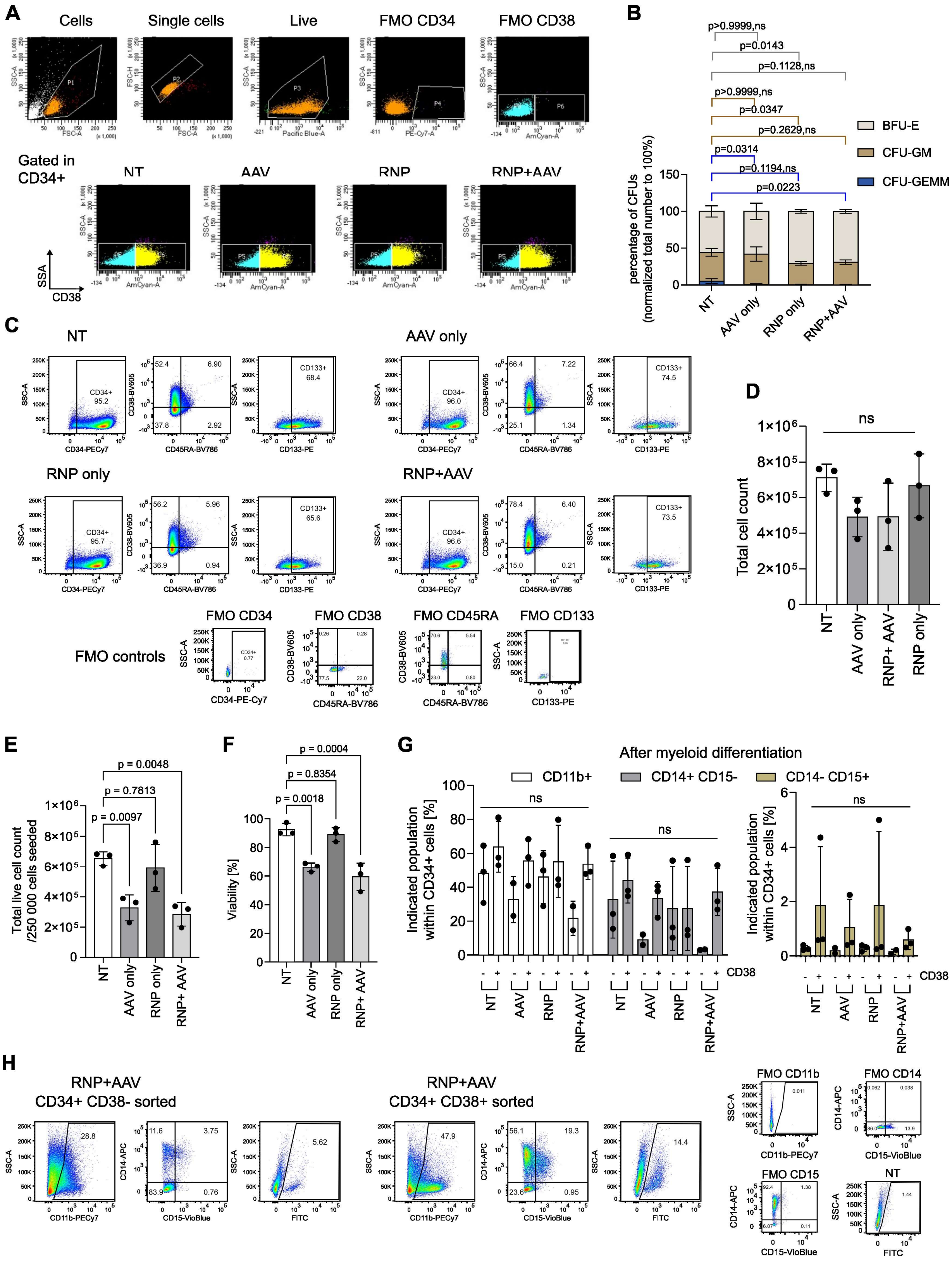

Figure S8. Editing of healthy human CD34+ hematopoietic stem cells shown in Figure 4A-D.

(**A**) Gating strategy supplementary to **Figure 4A and 4C** for FACS sorting of $CD34^+$ $CD38^+$ and $CD34^+$ $CD38^-$ subpopulation 2 days after editing of human HSPCs from healthy donors. CD34 and CD38 gates were set on FMO controls. (**B**) Percentage of CFUs normalized to the total number of colonies per sample shown in **Figure 4B**; n=3 donors, with 2 technical replicates per donor; data are shown as mean ± SD; BFU-E, burst-forming unit-erythroid; CFU-G, colony-forming unit granulocyte; CFU-M, colony-forming unit macrophage; CFU-GEMM, colony-forming unit granulocyte, erythrocyte, monocyte, megakaryocyte; one-way ANOVA with Dunnett's multiple comparisons test for CFU-GM and BFU-E, Kruskal-Wallis test with Dunn's multiple comparisons test for CFU-GEMM. (**C**) Representative flow cytometry plots showing CD34, CD38 versus CD45RA, CD133 expression in samples shown in **Figure 4C** and their respective FMO controls for gating. (**D**) Total cell count of healthy HSPCs 2 days after editing. (**E**) Total live cells and (**F**) percentage of cell viability as counted by trypan blue exclusion test 2 days after editing of 250 000 cells as starting material. (**G**) Percentage of CD11b+ (myeloid cells), CD14+ CD15- (monocyte/macrophages) and CD14- CD15+ (granulocytes) subpopulation in edited and sorted healthy HSPCS 14 days after myeloid differentiation. Myeloid subpopulations were not significantly different between $CD34^+$ $CD38^+$ and $CD34^+$ $CD38^-$ fractions nor between treatments. Results in **D-F** correspond to the state of cells shown in **Figure 4C**. In **D-G**, n=2 healthy donors, 3 technical replicates; data are shown as mean ± SD; one-way ANOVA followed by Dunnett's multiple comparisons test in **D-F** and Brown-Forsythe and Welch ANOVA test followed by Dunnett's T3 multiple comparisons test in **G**. (**H**) Representative flow cytometry plots showing CD11b, CD14 versus CD15, and GFP expression of edited and $CD34^+$ $CD38^+$/$CD34^+$ $CD38^-$ sorted fractions shown in **Figure 4D** 14 days after myeloid differentiation. FMO controls were used as gating reference.

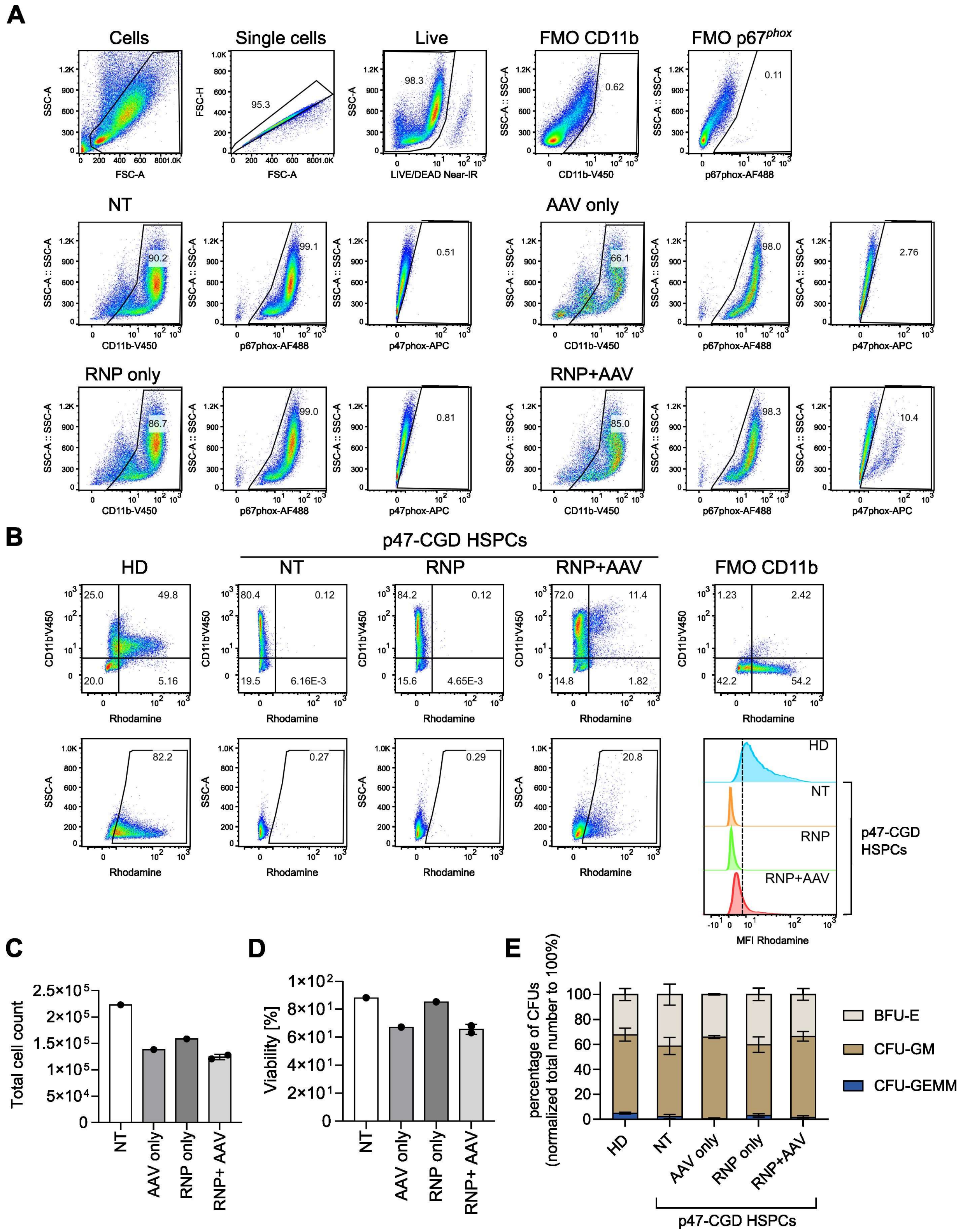

Figure S9. Editing of p47-CGD patient CD34+ hematopoietic stem cells shown in Figure 4E-J.

(**A**) Gating strategy supplementary to **Figure 4F 4H, and 4J** and their representative flow cytometry plots showing CD11b, p67*phox*, and p47*phox* expression of edited p47-CGD patient HSPCs after 14 days

of myeloid differentiation. (**B**) Representative flow cytometry plots of DHR tests performed on samples shown in **Figure 4G and 4I**, showing rhodamine positive cells gated within CD11b positive population. Rhodamine MFI is alternatively represented in a histogram. (**C**) Total cell count of p47-CGD HSPCs 2 days after editing. (**D**) Percentage of cell viability as counted by trypan blue exclusion test 2 days after editing of 250 000 p47-CGD HSPCs as starting material. (**E**) Percentage of CFUs normalized to the total number of colonies per sample shown in **Figure 4E**; n=1 donor, with 3 technical replicates; data are shown as mean ± SD.

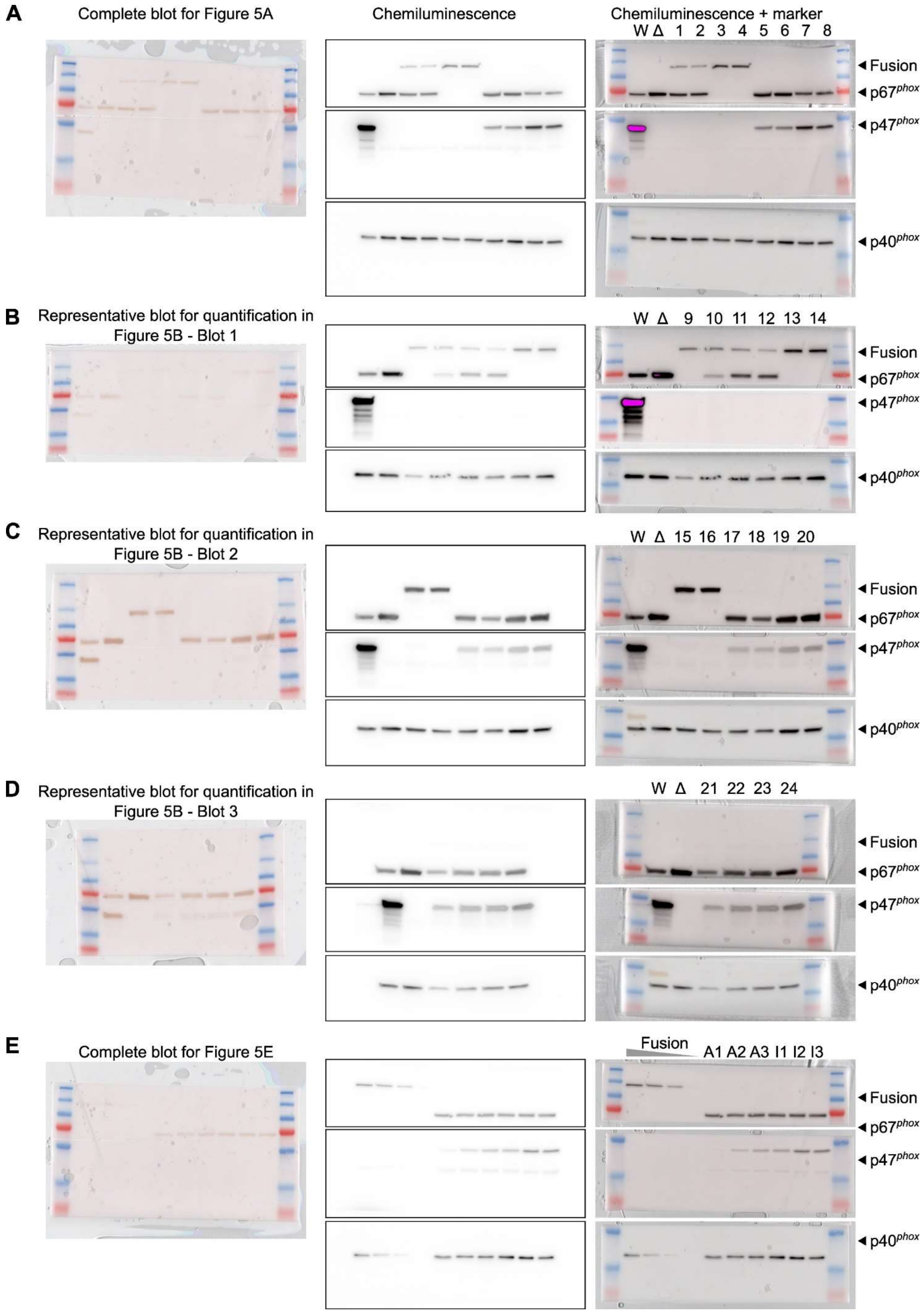
A
Complete blot for Figure 5A
Chemiluminescence
Chemiluminescence + marker
W Δ 1 2 3 4 5 6 7 8
Fusion
p67phox
p47phox
p40phox
B
Representative blot for quantification in Figure 5B - Blot 1
W Δ 9 10 11 12 13 14
Fusion
p67phox
p47phox
p40phox
C
Representative blot for quantification in Figure 5B - Blot 2
W Δ 15 16 17 18 19 20
Fusion
p67phox
p47phox
p40phox
D
Representative blot for quantification in Figure 5B - Blot 3
W Δ 21 22 23 24
Fusion
p67phox
p47phox
p40phox
E
Complete blot for Figure 5E
Fusion
A1 A2 A3 I1 I2 I3
Fusion
p67phox
p47phox
p40phox

Figure S10. Uncropped and unexposed western blot membranes shown in Figure 5A, 5B, and 5E.

(**A**) Complete uncropped blot (left) for **Figure 5A** imaged with protein ladder, PageRuler™ Plus Prestained Protein Ladder 10 to 250 kDa (Thermo Fisher Scientific, USA). The membrane was cut-split at the level of approximately 55 kDa for separate antibody incubations before imaging. Unexposed (middle) western blot membrane, immuno-stained for p67^{phox}-2A*-p47^{phox} fusion (using anti-human p67^{phox} antibody), anti-human p67^{phox}, anti-p47^{phox} antibody, and anti-p40^{phox} antibody conjugated with horse radish peroxidase (HRP) imaged by chemiluminescence detection, alongside the same membrane imaged for protein ladder (right). Protein samples loaded were: W, PLB-985 WT; Δ, PLB-985 *NCF1* ΔGT; 1 and 2, 'Fused hetero' clones; 3 and 4, 'Fused homo' clones; 5 and 6, 'Cleaved hetero' clones; 7 and 8, 'Cleaved homo' clones. (**B-D**) Representative blots of one set of differentiation experiment used for quantification in **Figure 5B**. Same set of clones were differentiated in 3 independent differentiation experiments and quantified for absolute intensities using ImageJ; n=16 clones. Protein samples loaded were: (**B**) 9 to 12, 'Fused hetero' clones; 13 and 14, 'Fused homo' clones; (**C**) 15 and 16, 'Fused homo' clones; 17 to 20, 'Cleaved hetero' clones; (**D**) 21 to 24, 'Cleaved homo' clones. (**E**) Uncropped, unexposed blot used in **Figure 5E**. Protein samples loaded were: A1-A3, RNP+AAV *NCF1* treated PLB-985 cells in bulk culture with 3 different (increasing) MOI; I1-I3, RNP+IDLV *NCF1* treated PLB-985 cells in bulk culture with increasing MOI. Arrowheads indicate the expected location of respective proteins based on the ladder position.

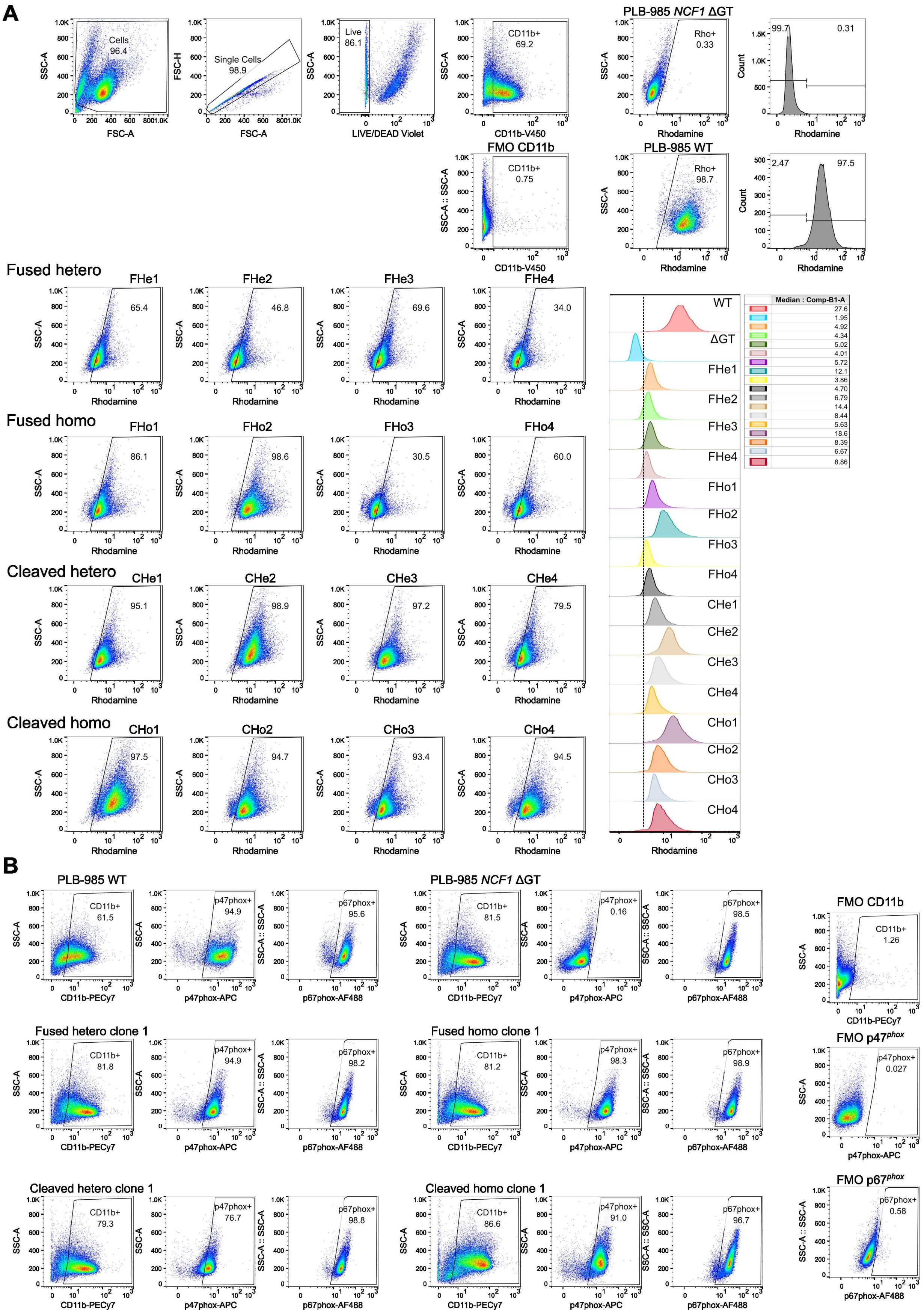
A
PLB-985 NCF1 ΔGT
PLB-985 WT
FMO CD11b
Cells 96.4
Single Cells 98.9
Live 86.1
CD11b+ 69.2
Rho+ 0.33
99.7
0.31
CD11b+ 0.75
Rho+ 98.7
2.47
97.5
SSC-A
FSC-A
FSC-H
LIVE/DEAD Violet
CD11b-V450
Rhodamine
Count
Fused hetero
FHe1 65.4
FHe2 46.8
FHe3 69.6
FHe4 34.0
Fused homo
FHo1 86.1
FHo2 98.6
FHo3 30.5
FHo4 60.0
Cleaved hetero
CHe1 95.1
CHe2 98.9
CHe3 97.2
CHe4 79.5
Cleaved homo
CHo1 97.5
CHo2 94.7
CHo3 93.4
CHo4 94.5
WT
ΔGT
Median : Comp-B1-A
27.6
1.95
4.92
4.34
5.02
4.01
5.72
12.1
3.86
4.70
6.79
14.4
8.44
5.63
18.6
8.39
6.67
8.86
B
PLB-985 WT
CD11b+ 61.5
p47phox+ 94.9
p67phox+ 95.6
PLB-985 NCF1 ΔGT
CD11b+ 81.5
p47phox+ 0.16
p67phox+ 98.5
FMO CD11b
CD11b+ 1.26
Fused hetero clone 1
CD11b+ 81.8
p47phox+ 94.9
p67phox+ 98.2
Fused homo clone 1
CD11b+ 81.2
p47phox+ 98.3
p67phox+ 98.9
FMO p47phox
p47phox+ 0.027
Cleaved hetero clone 1
CD11b+ 79.3
p47phox+ 76.7
p67phox+ 98.8
Cleaved homo clone 1
CD11b+ 86.6
p47phox+ 91.0
p67phox+ 96.7
FMO p67phox
p67phox+ 0.58
CD11b-PECy7
p47phox-APC
p67phox-AF488
SSC-A :: SSC-A

Figure S11. Flow cytometry analysis and DHR tests performed on 'Fused' and 'Cleaved' clones shown in Figure 5D.

(**A**) Gating strategy of a DHR test performed on 'Fused' and 'Cleaved' clones. Rhodamine positive cells were gated within CD11b positive population. FMO controls were used for CD11b gating and NT PLB-985 *NCF1* GT cells were referred as negative for rhodamine signal. Representative flow cytometry plots and histograms of rhodamine MFI of clones shown in **Figure 5D,** gated for rhodamine positive cells and rhodamine MFI values. 'Fused hetero' or 'Fused homo' clones were abbreviated as FHe and FHo, 'Cleaved hetero' or 'Cleaved homo' clones were abbreviated as CHe and CHo. (**B**) Representative flow cytometry plots showing CD11b expression, $p47^{phox}$ expression within CD11b positive cells and $p67^{phox}$ expression within CD11b positive cells in 'Fused' and 'Cleaved' clones. The MFI values of $p67^{phox}$ gated within CD11b positive cells were used for normalization of the clones' differentiation status in **Figure 5D**.

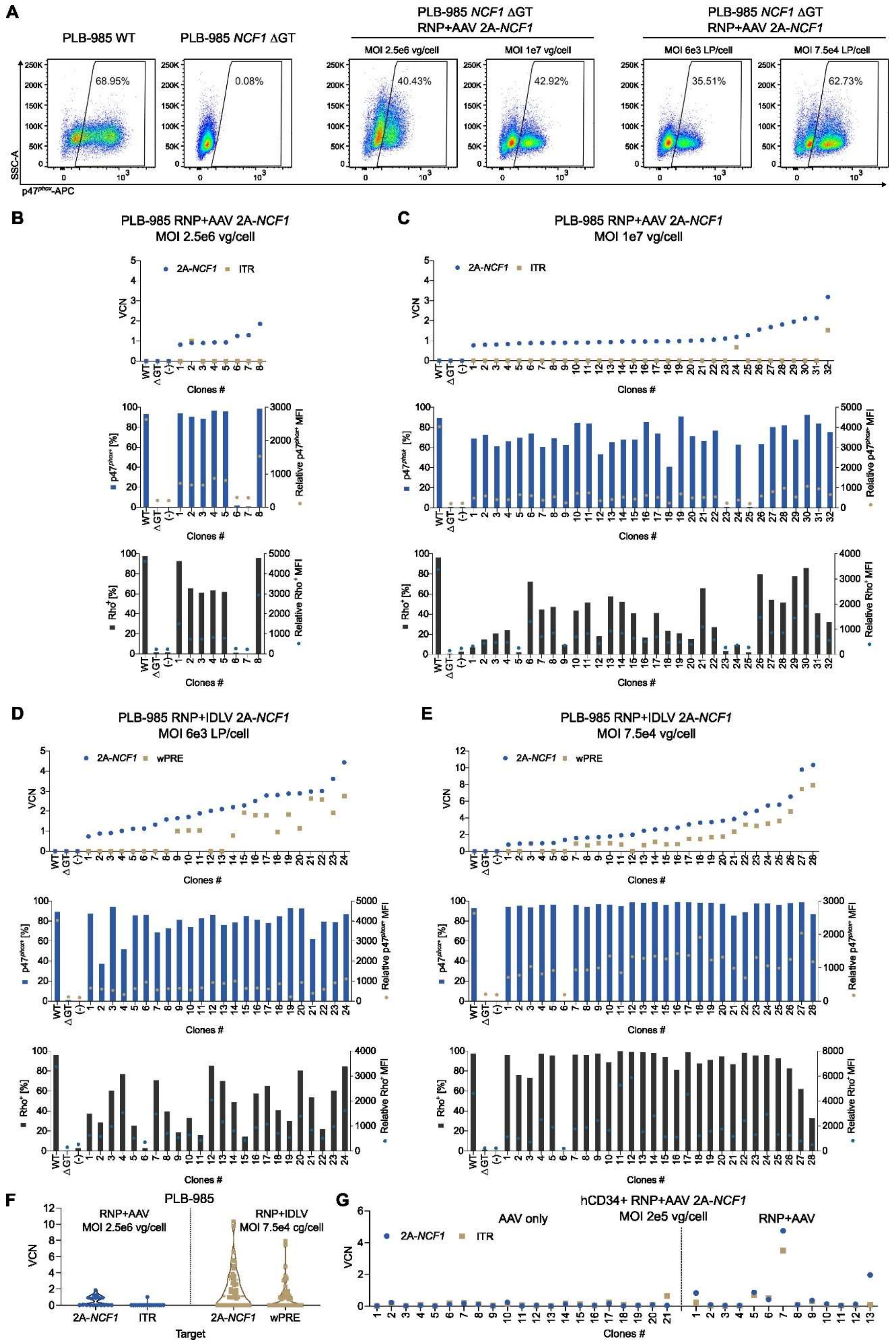

Figure S12. Detailed characterization of individual clones knocked-in with 2A-*NCF1* shown in Figure 6A.

(**A**) Flow cytometry analysis of p47*phox* expression to determine knock-in efficiency prior to single clone generation by fluorescence activated cell sorting. PLB-985 *NCF1* ΔGT cells were treated with RNP+AAV *NCF1* or with IDLV *NCF1* at the indicated MOIs, differentiated intro granulocytic-like cells, and analysed by flow cytometry for p47*phox* expression. (**B, C**) VCN, % p47*phox* expression, and % Rho-positive cells, and the respective absolute MFI of PLB-985 *NCF1* ΔGT cells treated with RNP + AAV *NCF1* at an MOI of 2.5×10^6 vg/cell (**B**, n=8) or 1×10^7 vg/cell (**C**, n=32). ITR, inverted terminal repeat; wPRE, woodchuck hepatitis virus post-transcriptional regulatory element. (**D, E**) VCN, % p47*phox* expression, and % Rho-positive cells, and the respective absolute MFI of PLB-985 *NCF1* ΔGT cells treated with RNP + IDLV *NCF1* at an MOI of 6×10^3 LP/cell (**D**, n=24) or 7.5×10^4 LP/cell (**E**, n=28). (**F**) VCN of 2A-*NCF1* versus ITR or wPRE in PLB-985 *NCF1* ΔGT cells treated with RNP + AAV *NCF1* or with IDLV *NCF1*. Each data point represents an individual clone. The higher the MOI used during gene editing, the higher was the frequency of unintended integration events detected (unintended integrations mean integrations other than the on-target knock-in at *NCF2*). IDLV-transduced samples showed higher frequency of unintended integration events (*e.g.*, at MOI 6×10^3 LP/cell, unintended integration events=14/24, knock-in efficiency of 35.6%) when compared with AAV-transduced samples (*e.g.*, at MOI 1×10^7 vg/cell, unintended integration events=2/32, knock-in efficiency of 43.0%) even with higher knock-in efficiencies. (**G**) VCN of 2A-*NCF1* versus ITR in hCD34+ cells treated with RNP + AAV *NCF1*.

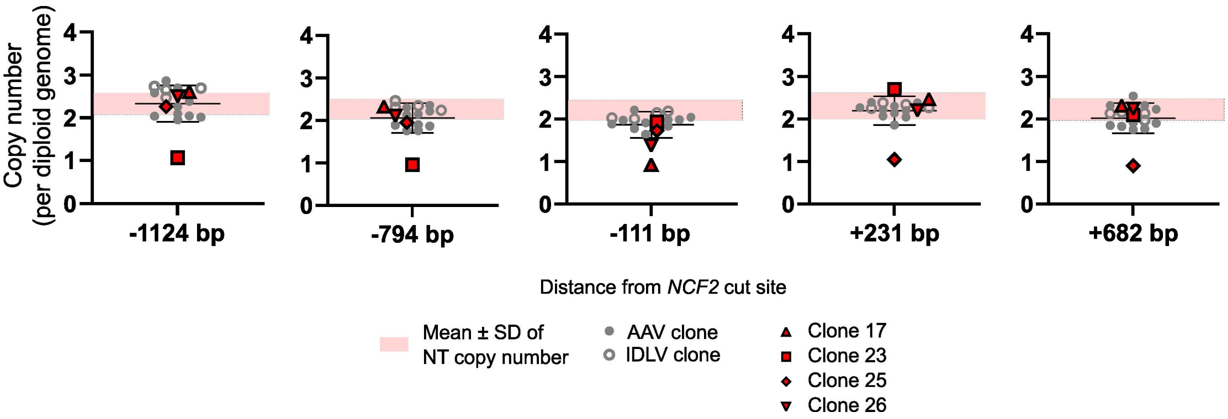

Figure S13. Evaluation of copy number gains or losses surrounding the *NCF2* on-target site.

Copy number detected 5 locations on the *NCF2* gene (in reference to 2 copies of *FOXP2* housekeeping gene) was evaluated in 13 AAV knocked-in clones (grey dots) and 5 IDLV knocked-in clones (unfilled grey circles). T1 to T5 represents primers and probes detecting copy number surrounding the *NCF2* cut site at positions -1124, -794bp, -111, +231 and +682 bp, respectively. A reference bar (pink) was set based on the mean ± SD of copy numbers determined from 15 non-treated PLB-985 WT or ΔGT

cells. Any copy number loss that deviates from the reference bar is considered as a deletion at the indicated location. Clones 17, 23, 25 and 26 were identified to have copy number losses surrounding the five sites. Referring to the flow cytometry data in **Figure S12**, the clones 23 and 25 indeed did not express p47^{phox} and did not produce ROS.

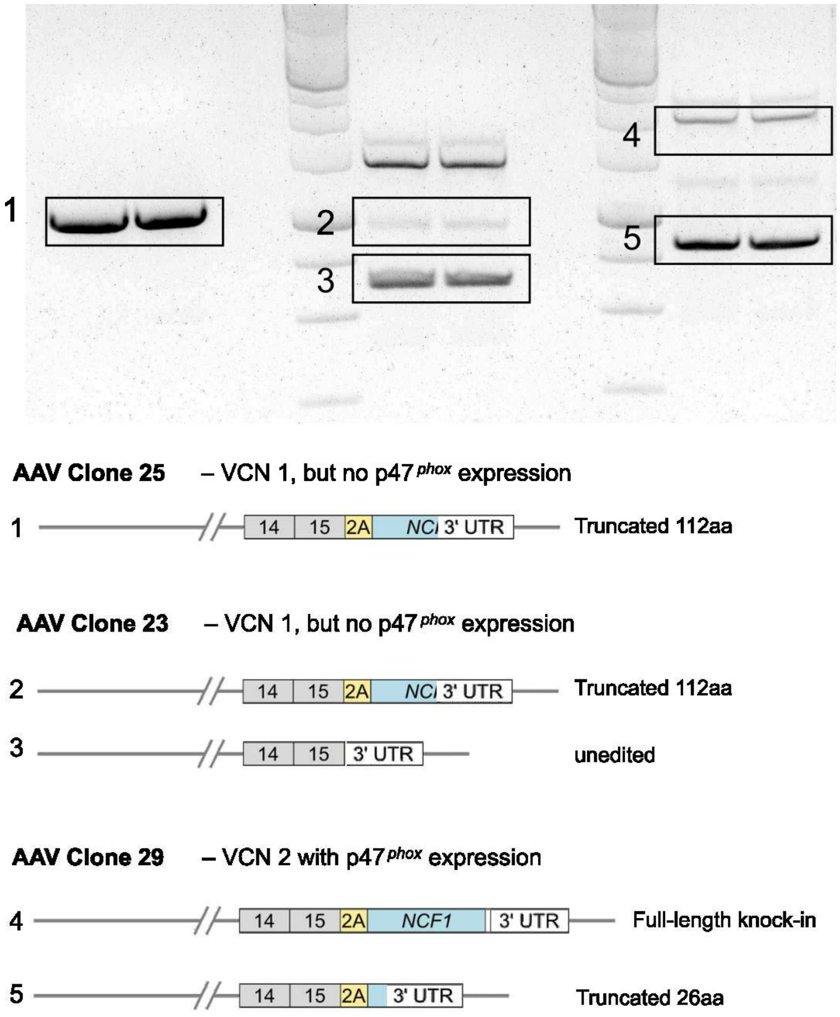

Figure S14. Several knocked-in clones carried truncated *NCF1* cDNA fragment.

(**A**) AAV knocked-in clones 23, 25 and 29 carried truncated *NCF1* cDNA at the *NCF2* target site as confirmed by Sanger sequencing. 2.3kb surrounding *NCF2* cut site was PCR amplified and gel extracted before sending for Sanger sequencing. Clones 23 and 25 were also identified to have on-target deletion by copy number ddPCR and to not express p47^{phox}/produce ROS (**Figure S12 and S13**).
